# Rhodium catalysed C-3/5 methylation of pyridines using temporary dearomatisation†

**DOI:** 10.1039/d0sc02759f

**Published:** 2020-08-10

**Authors:** Alexandru Grozavu, Hamish B. Hepburn, Elliot P. Bailey, Peter J. Lindsay-Scott, Timothy J. Donohoe

**Affiliations:** Chemistry Research Laboratory, University of Oxford Oxford OX1 3TA UK timothy.donohoe@chem.ox.ac.uk; Eli Lilly and Company Erl Wood Manor, Windlesham Surrey GU20 6PH UK

## Abstract

Pyridines are ubiquitous aromatic rings used in organic chemistry and are crucial elements of the drug discovery process. Herein we describe a new catalytic method that directly introduces a methyl group onto the aromatic ring; this new reaction is related to hydrogen borrowing, and is notable for its use of the feedstock chemicals methanol and formaldehyde as the key reagents. Conceptually, the C-3/5 methylation of pyridines was accomplished by exploiting the interface between aromatic and non-aromatic compounds, and this allows an oscillating reactivity pattern to emerge whereby normally electrophilic aromatic compounds become nucleophilic in the reaction after activation by reduction. Thus, a set of C-4 functionalised pyridines can be mono or doubly methylated at the C-3/5 positions.

## Abstract 2

Electron poor pyridines can be activated by reduction and then methylated at C3/5 using formaldehyde.

## Graphical abstract

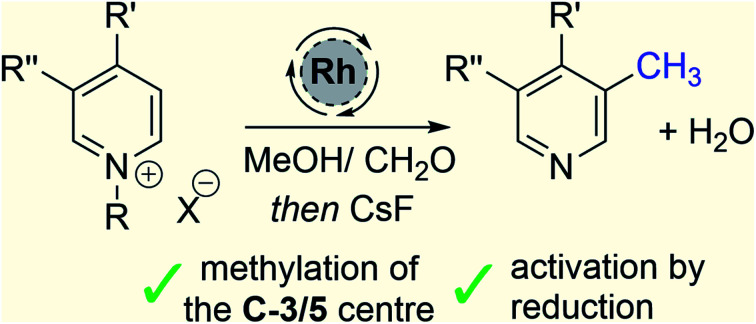

## Introduction

**Scheme 1:**
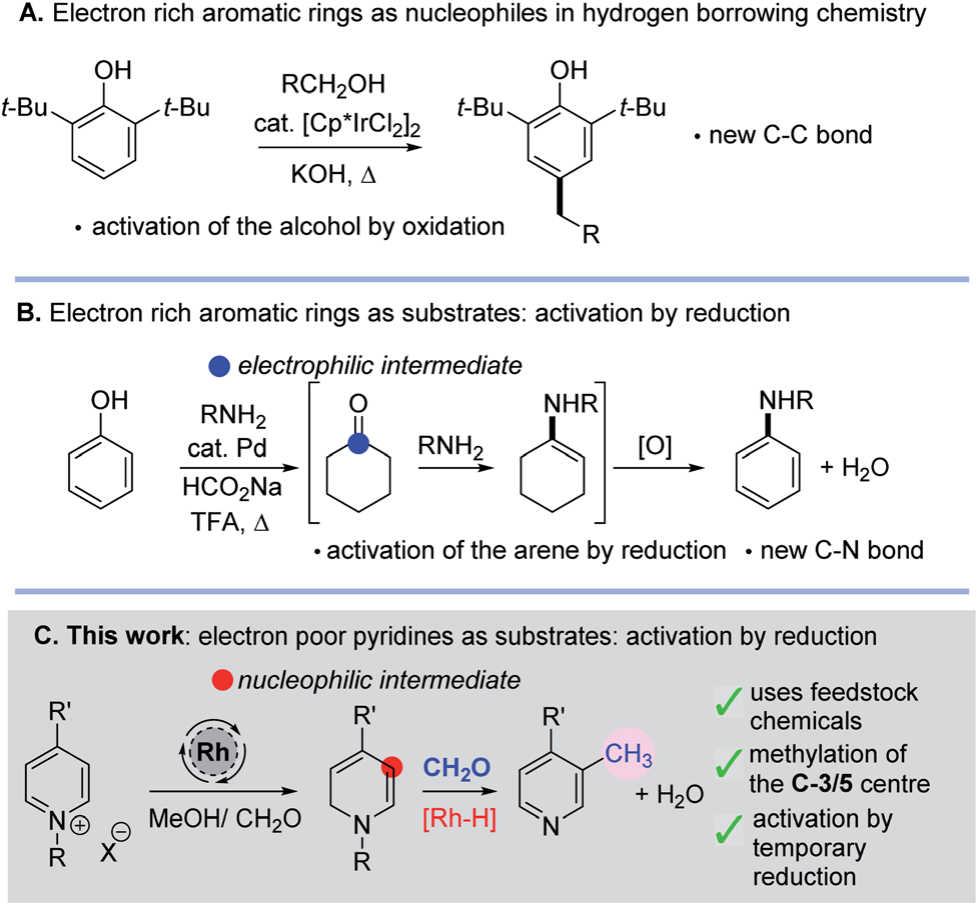
(A) Electron rich arenes as nucleophiles in hydrogen borrowing. (B) *In situ* activation of phenols to allow C–N bond formation. (C) This work; temporary dearomatisation as a tool for the C-3/5 methylation of pyridines.

The idea of reversibly altering the oxidation state of a molecule to enable reactive intermediates to be formed transiently is an attractive one for constructing both C–C and C–N bonds; it is most commonly found in hydrogen borrowing methodology.^1^ Typically new bonds are formed by a sequence that entails selective oxidation of an alcohol, nucleophilic addition to a carbonyl, loss of water and subsequent reduction of the organic substrate. This method has become extremely useful for the alkylation of amines and carbonyl compounds using alcohols.^1^ Intriguingly, aromatic rings have also been shown to participate in hydrogen borrowing chemistry in a number of different ways. For example, electron-rich aromatic rings such as phenols or indoles can be utilized as nucleophiles towards aldehydes, themselves generated *in situ* by oxidation of an alcohol (Scheme 1A).^2^ Intriguingly, work by Li has also shown that phenols could become activated (by an arene reduction reaction), thus turning an electron rich arene into an electrophilic intermediate allowing subsequent C–N bond formation and ultimately reoxidation (Scheme 1B).^3^

In this work, we have found new methodology which shows that electron deficient pyridine rings can also be activated by *in situ* reduction caused by metal hydride addition; in this case nucleophilic enamine intermediates are formed which enable new C–C bond formation. Pleasingly, this sequence leads to derivatisation of the C-3 position of the pyridine and so mono- and di-methylated pyridines are formed using methanol, formaldehyde and a transition metal catalyst (Scheme 1C). Note that the C-5-silylation of pyridines through a dearomatization/rearomatization sequence has been previously reported by Oestreich^4^ as has the alkylation of naphthyridines and quinolines with higher alcohols.^5,6^ In addition, there is a report on pyridine alkylation using methanol with Ni–Co ferrites at high temperatures (*ca.* 400 °C) in a down-flow vapour phase reactor.^7^

Interestingly, the temporary participation of dearomatised intermediates, formed *via* a reduction process involving hydride addition from a metal–H species, makes this approach complementary to the C–H activation and photocatalyst driven approaches that currently exist for pyridine functionalisation.^8,9^ The direct methylation of pyridines at C-3/5 is a valuable new reaction, and it is noted that the methylation of an aromatic ring is a fundamentally important process in areas ranging from DNA manipulation *in vivo*^10^ through to the magic methyl effect discovered in pharmaceutical research.^11^ Pyridine is the most common heteroaromatic motif found in small molecule FDA approved pharmaceutical drugs,^12^ making this methylation reaction all the more valuable.

## Results and discussion

**Scheme 2:**
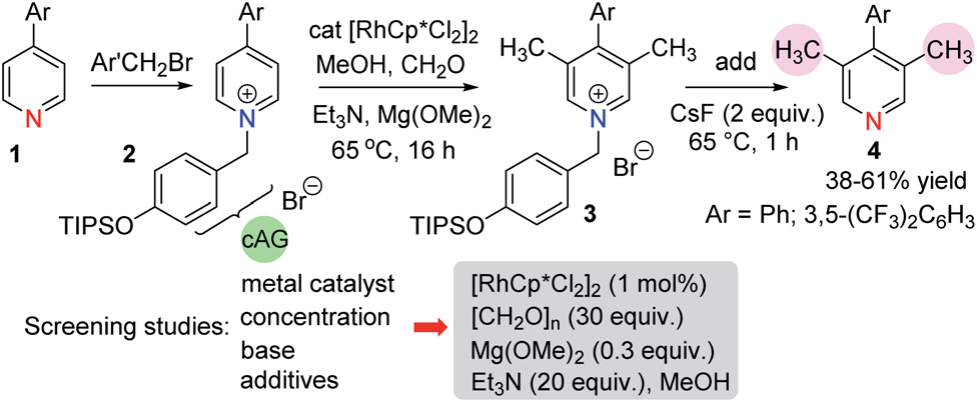
The general reaction sequence.

We began our work with an examination of 4-aryl pyridines because these substrates have previously been shown to participate in related catalytic reductive hydroxymethylation reactions.^13^ In order to provide electrophilic activation for the pyridine towards metal hydride reduction (Scheme 2) it was necessary to quaternarise the nitrogen with a benzyl halide. Therefore, we developed a cleavable activating group (cAG) that can be easily installed before methylation and removed at the end of the reaction simply by the addition of fluoride. This protocol avoids the difficult purification of pyridinium salts and allows isolation of methylated pyridines **4** in one pot. Taking the conditions required for the reductive hydroxymethylation of 4-aryl pyridines as a starting point,^13^ the variables that we examined in an extensive screening exercise (see ESI, Tables S1–S4†) were the following: (i) metal catalyst – here we found that Rh was consistently better at promoting methylation than other metals; (ii) dilution – running the reaction at a dilute concentration (0.1 M) was beneficial; (iii) base – the addition of a magnesium alkoxide base, together with an added amine gave the highest yields of methylation products; (iv) additives – generally, the addition of additives such as iodide^14^ was detrimental to the methylation reaction, although iodide did provide a small increase in yields when very electron deficient pyridines were used.

In these early studies we were not able to optimise the reaction to form the C-3 mono methyl pyridine because the reaction always gave significant amounts of C-3,5-dimethylated derivatives. Therefore, we found it beneficial to run the reaction to full conversion so that the dimethylated arene could be isolated cleanly.

Once a set of suitable conditions were identified, we examined the C-3/5 dimethylation of a set of C-4 substituted pyridine rings (Table 1(A)). 4-Aryl substituted pyridines containing electron neutral or electron withdrawing aryl groups are well tolerated under this methodology (**4a–4j**) and C-4 keto-substituted substrates are also viable (**4k**). In terms of scope, the current methodology allows for a substitution pattern that is complementary to those already established in the literature,^15^ and produces a set of 3,4,5-substituted pyridines. Importantly, it allows for the instalment of a methyl group adjacent to a non-coordinating substituent. Although the yields of this reaction range from 38–68%, this is simply a reflection of the significant number of chemical steps involved, *vide infra*. The remaining mass from these experiments comprised of a complex mixture of unidentified compounds.

**Table 1:** The catalytic C-3 and C-5 methylation of pyridines.^a^^,^^b^ (A) Dimethylation reactions of C-4 substituted pyridines; (B) mono-methylation of C-3 blocked pyridines; (C) selective activation and dimethylation of one heteroarene in the presence of another

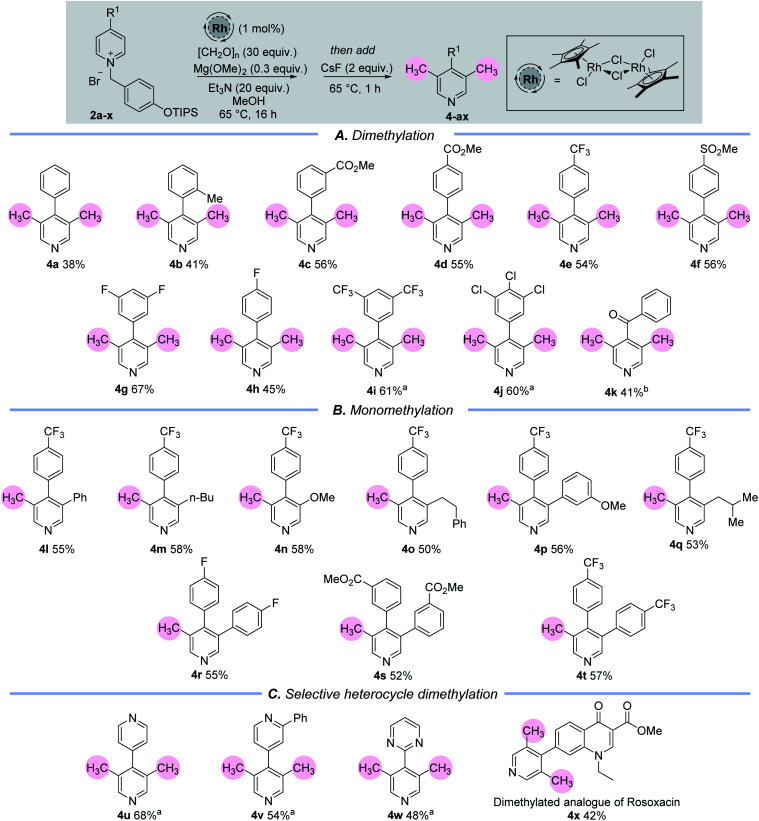

a NaI (1.0 equiv.) added.

b Pyridinium iodide used and NaI (1.0 equiv.) added. Reaction temperature = 40 °C. (see ESI for details).

Experiments showed that the C-4 position of the pyridine must be substituted because otherwise multi-component mixtures were formed, which we attribute to over addition by the metal hydride at the unencumbered C-4 position. Moreover, we examined other C-4 substituents in addition to aryl, keto and heteroaryl groups. However, in each case the reaction was unsuccessful because of presumed deprotonation *in situ* (C-4 alkyl) or S_N_Ar processes (C-4 heteroatom), see ESI.†

Pleasingly, when the 3-position of the starting pyridine is already substituted with electron rich or electron deficient groups, for example with an alkyl, aryl or ether group (**1l–1t**), then mono-methylation takes place at the C-5 position to generate the 3,4,5-substituted pyridines, **4l–4t** (Table 1(B)).

We also sought to utilise the selective cAG activation of a pyridine nitrogen in the presence of other heteroarenes to enable selective catalytic arene methylation (Table 1(C)). In the first instance, this was accomplished through the selective mono-alkylation of symmetrical bis-pyridine, which then delivered product **4u** whereby only one pyridine ring had been doubly methylated in the presence of another. Steric and electronic effects can also be exploited to allow differentiation between different heteroarene N atoms. Note the selective activation and dimethylation sequence leading to the bis-arenes **4v** and **4w** in which the deactivating effects of an *ortho*-Ph group and a pyrimidine nitrogen were used to deter installation of the cAG and subsequent methylation. Finally, a more complex example lay in the selective activation and dimethylation of the ester of rosoxacin,^16^ a prescription antibiotic compound. The selective formation of **4x** shows clearly that this methodology has significant potential for the selective and late-stage introduction of one, or two, methyl groups onto pharmaceutically important molecules.

**Scheme 3:**
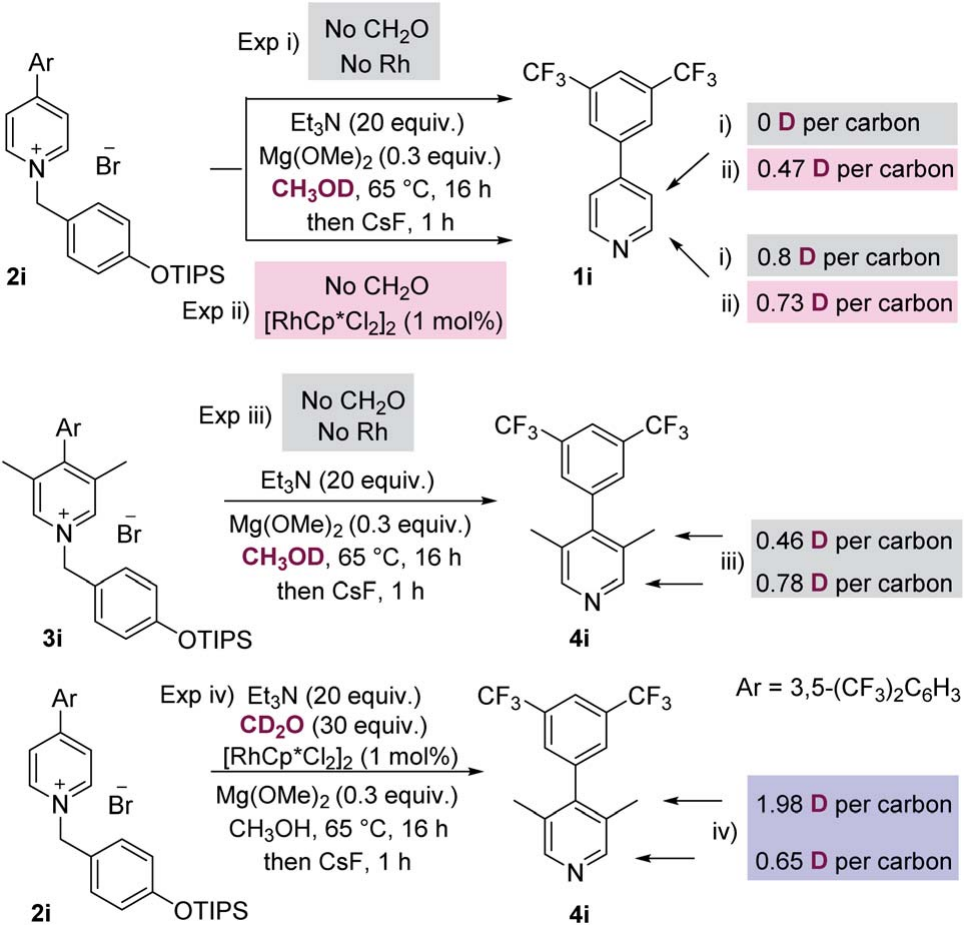
Experiments to probe the reaction mechanism. (i/ii) show exchange at C-2 and metal promoted exchange at C-3; (iii) shows exchange at C-2 and the C-3 methyl groups in the product; (iii) CD_2_O shows this reagent to be the source of the methyl groups.

We then conducted a number of control and labelling experiments in order to probe the reaction mechanism (Scheme 3). As expected, the presence of both rhodium catalyst and formaldehyde were essential for the methylation process (see ESI† for further details). The results given in labelling experiment (i), Scheme 3, show that proton exchange at the C-2 position of starting material **2i** occurs in the presence of base, presumably *via* a benzylic deprotonation/reprotonation process. Interestingly, the presence of rhodium catalyst (see experiment (ii)) also promoted exchange at the C-3 position of the recovered starting material **1i**.

Labelling experiment (iii) was conducted on the initial product of the methylation reaction **3i**, and reveals that the C-3 methyl groups of this product are themselves susceptible to proton exchange with the protic solvent by acid/base reactions, which complicates analysis. Finally, experiment (iv) which isolated and analysed the methylated pyridine product, shows that most of the deuterium present in added CD_2_O is present at the C-3 methyl group, which is consistent with this reagent being the source of the methyl group itself.

**Scheme 4:**
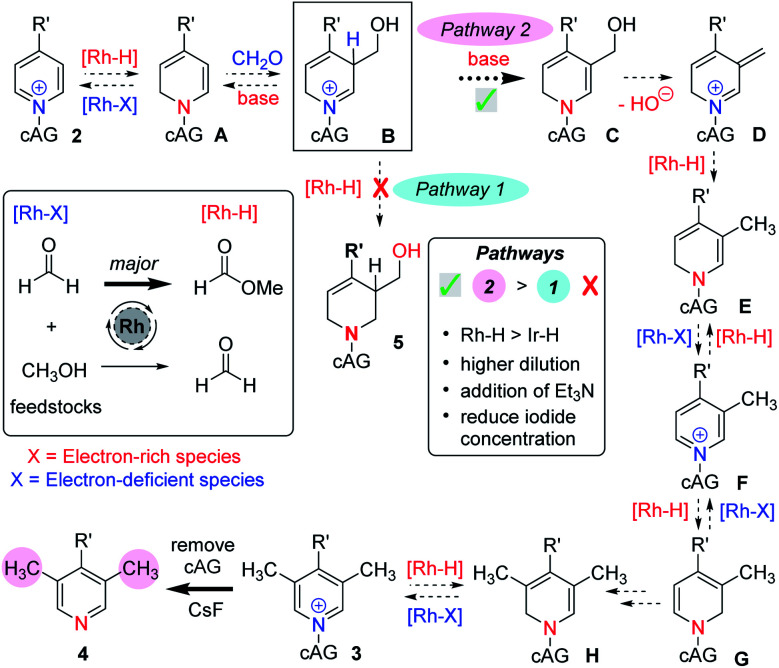
An outline of the proposed mechanism.

Our earlier work on the reductive hydroxymethylation of similar pyridines using iridium catalysis^13^ had investigated the *in situ* formation of metal hydrides *via* oxidation of the feedstocks formaldehyde and methanol,^17^ together with reversible Ir–H addition to the C-2 position of a pyridinium ion. Using these basic steps and the mechanistic experiments described above, we now propose the following mechanism (Scheme 4).

We think that a key intermediate in this sequence is iminium salt **B**, which in our earlier work was reduced to the dearomatised product 5 *in situ* by a metal hydride species (Pathway 1). We reason that by diverting the electrophilic intermediate **B** from this iminium reductive process and favouring a deprotonation instead, we have followed a pathway that regains aromaticity later on (Pathway 2). Starting from pyridinium **2** and following the mechanism (Pathway 2, Scheme 4), it can be seen that partial and temporary transition metal mediated dearomatisation of the pyridine (see **A**), followed by reaction with formaldehyde, which is conceivably activated by the magnesium cation, (**B**), deprotonation (**C**), loss of water (**D**), exocyclic reduction by metal hydride (**E**) and rearomatisation would lead to C-3 methylated pyridine derivative **F**. Note that it is also possible for the transformation of **D**→**F** to involve a transition metal free tautomerisation process.^13*b*^

When both C-3 positions are free on the starting pyridine, we suggest that there is an iterative process that leads to the formation of a C-3,5 dimethylated pyridinium **3** (see **F**→**3**). At this point any Rh–H catalysed dearomatisation of the product (**3**) would lead to sterically hindered enamine H, which does not react with formaldehyde and instead can revert back to pyridinium **3**.

This mechanism is consistent with the labelling experiments shown in Scheme 3. In particular, the exchange at C-3 promoted by the rhodium catalyst (see experiment (ii), Scheme 3) can be rationalised by invoking oxidation of methanol (no CH_2_O is present) by the rhodium catalyst, and the Rh–H species thus generated adding reversibly to the pyridinium C-2 position forming intermediate **A** (see Scheme 4). Intermediate **A**, in the absence of added excess formaldehyde electrophile, can then exchange deuterium at C-3 by reversible deuteration from the CH_3_OD solvent.

Finally, the relatively high amount of D incorporated at C-2 in experiment (iv), Scheme 3, is also consistent with reversible Rh–D (this time originating from the oxidation of CD_2_O) addition at C-2, which competes with the background deprotonation/reprotonation from CH_3_OH.

## Conclusions

In conclusion, we have developed a set of mild catalytic conditions that allow di- and mono-methylation of pyridines at the relatively unreactive C-3 and C-5 positions. This reaction utilises readily available formaldehyde as the source of the methyl group and does not require a directing group. Moreover, the selective activation and methylation of multi-arene ring systems has also been demonstrated and this holds many opportunities for use in medicinal chemistry lead optimisation programmes in both academia and industry.

## Conflicts of interest

There are no conflicts to declare.

## Supplementary Material

SC-011-D0SC02759F-s001
