# Supplementary material for: Rhodium catalysed C-3/5 methylation of pyridines using temporary dearomatisation
Source: Chem Sci. 2020 Aug 10;11(32):8595–9. doi: 10.1039/d0sc02759f (PMC8163342; doi:10.1039/d0sc02759f)

Electronic Supplementary Material (ESI) for Chemical Science.
This journal is © The Royal Society of Chemistry 2020

Rhodium Catalysed C3/5 Methylation of Pyridines using Temporary Dearomatisation

Alexandru Grozavu[1], Hamish B. Hepburn[1], Elliot P. Bailey[1], Peter J. Lindsay-Scott[2], Timothy J. Donohoe[1*]

[1] Chemistry Research Laboratory, University of Oxford, Mansfield Road, Oxford, OX1 3TA, UK.
[2] Eli Lilly and Company, Erl Wood Manor, Windlesham, Surrey, GU20 6PH, UK

Supporting Information

Contents

General Experimental Techniques S2
Screening Studies S4
Preparation of the Alkylating Reagents S10
Preparation of the Pyridine Precursors S13
Preparation of the Pyridinium Salts S29
Preparation of 3-Methylated Pyridines S56
Unproductive Substrates S73
NMR Spectra
- Pyridine Precursors S74
- Starting Materials S84
- Methylated Products S114

Mechanistic Experiments S138
Deuterium Labelling Studies S139

General Experimental Techniques

Chemicals and solvents

Unless stated otherwise, all chemicals were purchased from commercial suppliers (Sigma-Aldrich, Fluorochem, Alfa Aesar) and used without further purification. The magnesium methoxide was purchased from Alfa Aesar as a 6-10% w/w solution in methanol and was titrated using EDTA in the presence of Eriochrome Black T as an indicator.

Glassware and reaction conditions

Reactions were carried out in oven-dried microwave vials under an atmosphere of air unless otherwise stated.

Analytical techniques

^{1}H, ^{19}F and ^{13}C NMR spectra were recorded on a Bruker AVIII400 Spectrometer (400 MHz 376 MHz and 100 MHz respectively) or a Bruker AVII500 (^{1}H: 500 MHz and ^{13}C: 126 MHz) in $CDCl_3$ or DMSO-d_6, and referenced to residual solvent peaks. Chemical shifts δ are quoted in parts per million (ppm) to the nearest 0.01 for ^{1}H and 0.1 for ^{13}C, coupling constants *J* are quoted in Hz to the nearest 0.1 and splitting are recorded as singlet (s), doublet (d), triplet (t), quartet (q), doublet of a doublet (dd), doublet of a doublet of a doublet (ddd), and multiplet (m). Assignments were based upon COSY, HSQC and HMBC experiments. Infrared spectra were recorded on a Bruker Tensor 27 FT-IR spectrometer fitted with an Attenuated Total Reflectance (ATR) sampling accessory. Absorption maxima are quoted in wavenumbers (cm^{-1}). High resolution mass spectra were recorded on a Bruker MicroTof (resolution = 10000 FWHM). Melting points (m.p.) were obtained using a Lecia VMGT heated-stage microscope and are uncorrected.

Chromatography

Analytical thin layer chromatography was performed on pre-coated silica gel aluminium sheets from Merck (TLC Silica Gel 60 F_{254s}). Spots were visualized either by the quenching of UV fluorescence or by staining with phosphomolybdic acid solution. Preparative flash column chromatography (FCC) was carried out using Geduran Silica Gel 60 (40 µm – 63 µm) from Merck.

Screening Studies

Reaction Development

General method for screening reactions In a 10 mL microwave vial equipped with a stirring bar was added the corresponding pyridinium salt **1**, followed by the specified amount of paraformaldehyde and additive. In a 5 mL volumetric flask was added $[RhCp^*Cl_2]_2$ followed by a solution of $Mg(OMe)_2$ in methanol and additional methanol to form a clear violet solution. An aliquot of this solution was added to the microwave vial charged with all the solid components, along with NEt_3 and additional methanol in order to reach the required stoichiometries and concentrations mentioned in **Tables S1-S4.** The reaction was sealed and left stirring at the specified temperature for 16 hours. To the reaction was added trimethoxybenzene (IS) and an aliquot was removed (~0.5 mL), concentrated and dissolved in 0.5 mL MeOD-d_4, which was subsequently subjected to NMR spectroscopy.

Table S1. Selected screening and optimisation entries for the model substrate

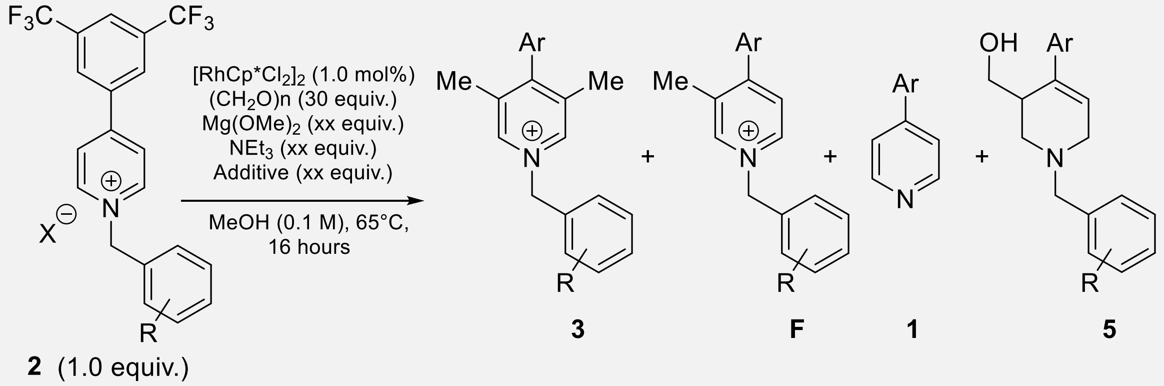

No.	X	R	Iodide conc. (M)	$Mg(OMe)_2$ equiv.	NEt_3 equiv.	Consumption of 2[a]	3[a,b]	F[a]	1[a]	5[a]
1	Br	H	x	0.75	x	>98	**36**	15	8	<2
2	Br	*o*-NO_2	x	0.75	x	>98	**38**	5	6	<2
3	Br	*o*-NO_2	x	0.50	x	>98	**43**	3	6	<2
4	Br	H	x	0.50	3	>98	**36**	13	30	<5
5	I	H	0.1	0.50	3	>98	**59**	<2	x	12
6	I	H	0.1	0.30	20	>98	**62**	x	x	6
7	I	*p*-OTIPS	0.1	0.30	20	>98	**65**	x	x	7
8[c]	Br	*p*-OTIPS	0.1	0.30	20	>98	**65**	x	x	7
9	Br	*p*-OTIPS	x	0.30	20	>98	**62**	<2	<2	2

The screening reactions were run on 0.25 mmol of substrate in 10 mL sealed microwave vials were assessed by NMR. (^{1}H NMR and ^{19}F NMR). [a] NMR Yield. [b] For the *p*-OTIPS salts the NMR yield is calculated as the sum of the protected and deprotected product (some product deprotects under the reaction conditions). [c] To the reaction was added 1.0 equiv. of NaI as additive.

Table S2. Screening and Optimisation

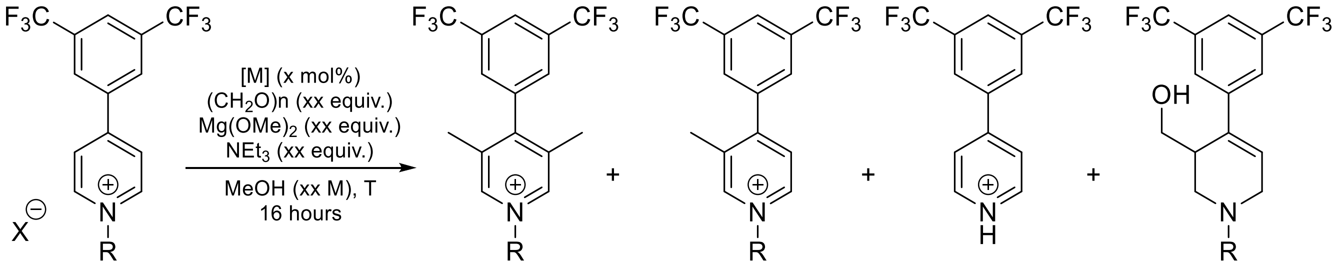

2 (1.0 equiv) **3** **F** **1** **5**

No.	X	R	M (mol%)		Add. (equiv.)	Total iodide conc. (M)	NEt_3 equiv.	CH_2O equiv.	Base (equiv.)		T (°C)	Conc. of 1 (M)	Consumption of 2[a]	3[a,b]	F[a]	1[a]	5[a]	Comments
1	I	Bn	[Ir]	1	KI (4.00)	1.0	x	20	$Mg(OMe)_2$	0.75	65	0.2	40	**trace**	trace	x	32	
2	I	Bn	[Ir]	1	KI (2.00)	0.6	x	20	$Mg(OMe)_2$	0.75	65	0.2	45	**1**	1	x	35	
3	I	Bn	[Ir]	1	x	0.2	x	20	$Mg(OMe)_2$	0.75	65	0.2	61	**2**	3	x	39	
4	Br	Bn	[Ir]	1	x	x	x	20	$Mg(OMe)_2$	0.75	65	0.2	>98	**5**	7	trace	<5	
5	I	Bn	[Rh]	1	x	0.2	x	20	$Mg(OMe)_2$	0.75	65	0.2	>98	**14**	trace	x	60	54 % **5** isolated
6	I	Bn	[Rh]	1	KI (2.00)	0.6	x	20	$Mg(OMe)_2$	0.75	65	0.2	>98	**6**	12	x	45	
7	I	Bn	[Rh]	1	x	0.2	x	20	$Mg(OMe)_2$	0.75	45	0.2	95	**6**	18	x	56	
8	I	Bn	[Rh]	1	KI (2.00)	0.6	x	20	$Mg(OMe)_2$	0.75	45	0.2	80	**3**	10	x	42	
9	Br	Bn	[Rh]	1	x	x	x	20	$Mg(OMe)_2$	0.75	45	0.2	>98	**6**	21	4	4	
10	Br	Bn	[Rh]	1	x	x	x	20	$Mg(OMe)_2$	0.75	65	0.2	>98	**30**	7	3	3	
11	I	Bn	[Ru]	1	x	0.2	x	20	$Mg(OMe)_2$	0.75	65	0.2	>98	**5**	5	x	36	Unidentified mixture of by-products
12	I	Bn	[Rh]	1	x	0.1	x	20	$Mg(OMe)_2$	0.75	65	0.1	>98	**40**	trace	x	21	
13	Br	Bn	[Rh]	1	x	x	x	20	$Mg(OMe)_2$	0.75	65	0.1	90	**9**	9	28	<2	Moderate signs of degradation
14	Br	Bn	[Rh]	1	x	x	x	10	$Mg(OMe)_2$	0.75	65	0.1	80	**6**	7	38	<2	
15	Br	Bn	[Rh]	1	x	x	x	30	$Mg(OMe)_2$	0.75	65	0.1	>98	**36**	15	8	<2	

16	Br	Bn	[Rh]	1	x	x	x	20	$Mg(OMe)_2$	1.5	65	0.1	90	**8**	7	29	<2	
17	Br	Bn	[Rh]	1	x	x	x	30	KOH	1.5	65	0.1	95	**7**	30	2	<2	
18	Br	o-NO_2	[Rh]	1	x	x	x	20	$Mg(OMe)_2$	0.75	65	0.1	>98	**33**	5	8	<2	
19	Br	o-NO_2	[Rh]	1	x	x	x	20	$Mg(OMe)_2$	0.5	65	0.1	>98	**35**	4	6	<2	
20	Br	o-NO_2	[Rh]	1	x	x	x	20	$Mg(OMe)_2$	1.5	65	0.1	>98	**26**	4	10	<2	
21	Br	o-NO_2	[Rh]	1	x	x	x	10	$Mg(OMe)_2$	0.75	65	0.1	>98	**18**	3	13	<2	Debenzylation is still observed (formation of **1**)
22	Br	o-NO_2	[Rh]	1	x	x	x	30	$Mg(OMe)_2$	1.0	65	0.1	>98	**37**	6	6	<2	
23	Br	o-NO_2	[Rh]	1	x	x	x	30	$Mg(OMe)_2$	0.75	65	0.1	>98	**38**	5	6	<2	
24	Br	o-NO_2	[Rh]	1	x	x	x	30	$Mg(OMe)_2$	0.5	65	0.1	>98	**43**	3	6	<2	
25	Br	o-NO_2	[Rh]	1	x	x	x	30	NaOMe	1.0	65	0.1	>98	**x**	x	x	x	Complex degradation mixture.
26	Br	o-NO_2	[Rh]	1	x	x	x	30	KOH	1.0	65	0.1	>98	**x**	x	x	x	Complex degradation mixture
27	I	o-NO_2	[Rh]	1	x	0.1	x	30	$Mg(OMe)_2$	0.5	65	0.1	95	**14**	3	2	21	
28	Br	o-NO_2	[Rh]	1	NaI (0.25)	0.025	x	30	$Mg(OMe)_2$	0.5	65	0.1	>98	**28**	3	3	9	
29	Br	o-NO_2	[Rh]	1	NaI (0.50)	0.050	x	30	$Mg(OMe)_2$	0.5	65	0.1	>98	**19**	3	3	15	
30	I	Bn	[Rh]	1	x	0.1	1	30	$Mg(OMe)_2$	0.5	65	0.1	>98	**46**	trace	x	23	
31	I	Bn	[Rh]	1	x	0.1	2	30	$Mg(OMe)_2$	0.5	65	0.1	>98	**55**	trace	x	14	
32	I	Bn	[Rh]	1	x	0.1	3	30	$Mg(OMe)_2$	0.5	65	0.1	>98	**59**	trace	x	12	
33	I	Bn	[Rh]	1	x	0.1	2	30	$Mg(OMe)_2$	0.3	65	0.1	>98	**56**	trace	x	14	
34	I	o-NO_2	[Rh]	1	x	0.1	2	30	$Mg(OMe)_2$	0.5	65	0.1	>98	**15**	21	3	11	
35	Br	Bn	[Rh]	1	x	x	3	30	$Mg(OMe)_2$	0.3	65	0.1	>98	**36**	13	30	<5	Significant debenzylation
36	I	Bn	[Rh]	1	x	0.1	3	30	$Mg(OMe)_2$	0.3	65	0.1	>98	**61**	x	x	11	
37	I	Bn	[Rh]	1	x	0.1	3	30	x	x	65	0.1	>98	**45**	trace	x	12	Incomplete reaction
38	I	Bn	[Rh]	1	x	0.1	5	30	$Mg(OMe)_2$	0.3	65	0.1	>98	**61**	x	x	8	
39	I	Bn	[Rh]	1	x	0.1	10	30	$Mg(OMe)_2$	0.3	65	0.1	>98	**63**	x	x	7	
40	I	Bn	[Rh]	1	x	0.1	20	30	$Mg(OMe)_2$	0.3	65	0.1	>98	**62**	x	x	6	
41	Br	p-OTIPS	[Rh]	1	x	x	20	30	$Mg(OMe)_2$	0.3	65	0.1	>98	**62**	trace	trace	2	
42	Br	p-OTIPS	[Rh]	1	NaI (0.33)	0.033	20	30	$Mg(OMe)_2$	0.3	65	0.1	>98	**65**	x	x	4	
43	Br	p-OTIPS	[Rh]	1	NaI (0.66)	0.066	20	30	$Mg(OMe)_2$	0.3	65	0.1	>98	**65**	x	x	6	
44	Br	p-OTIPS	[Rh]	1	NaI (1.00)	0.1	20	30	$Mg(OMe)_2$	0.3	65	0.1	>98	**65**	x	x	7	60 % isolated

45	I	p-OTIPS	[Rh]	1	x	0.1	20	30	$Mg(OMe)_2$	0.3	65	0.1	>98	**65**	x	x	7	61 % isolated
46	I	p-OTIPS	[Rh]	0.5	x	0.1	20	30	$Mg(OMe)_2$	0.3	65	0.1	>98	**44**	12	x	8	Incomplete reaction
47	I	p-OTIPS	[Rh]	1	x	0.1	20	30	$Mg(OMe)_2$	0.3	50	0.1	>98	**49**	7	x	9	Incomplete reaction
48	I	p-OTIPS	[Rh]	1	x	0.1	x	30	NaOMe	1.5	65	0.1	>98	**x**	x	x	x	TIPS group not stable to nucleophilic base

The screening reactions were run on 0.25 mmol of substrate in 10 mL sealed microwave vials.

[a] NMR Yield. [b] For the *p*-OTIPS salts the NMR yield is calculated as the sum of the protected and deprotected product (some product deprotects under the reaction conditions).

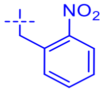

= o-NO_2

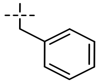

= Bn

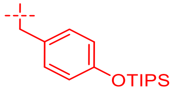

= p-OTIPS

[Ir]= $[IrCp^*Cl_2]_2$
[Rh]= $[RhCp^*Cl_2]_2$
[Ru]= $[Ru(p\text{-}cymene)Cl_2]_2$

In order to extend the methodologies to substrates with different electronic properties we undertook addition screening and optimisation on both less electron deficient pyridines (**Table S3**) and also on more electron deficient pyridines (**Table S4**).

Table S3. Screening and Optimisation

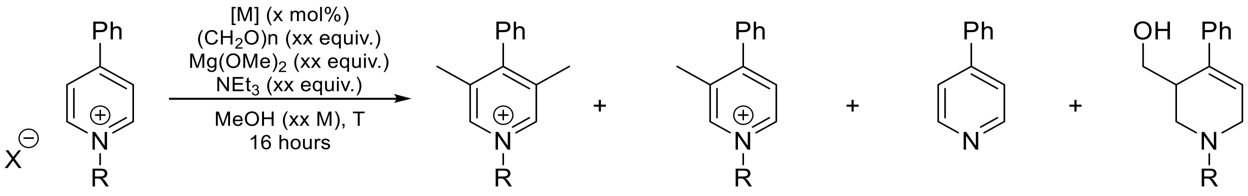

2 (1.0 equiv) **3** **F** **1** **5**

No.	X	R	M (mol%)		Add. (equiv.)	Total iodide conc. (M)	NEt_3 equiv.	CH_2O equiv.	Base (equiv.)		T (°C)	Conc. of 1 (M)	Consumption of 2[a]	3[a,b]	F[a]	1[a]	5[a]	Comments
1	I	Bn	[Rh]	1	x	0.1	10	30	$Mg(OMe)_2$	0.3	65	0.1	>98	**23**	10	x	34	
2	I	o-NO_2	[Rh]	1	x	0.1	10	30	$Mg(OMe)_2$	0.3	65	0.1	>98	**8**	trace	1	37	
3	I	Bn	[Rh]	1	x	0.1	20	30	$Mg(OMe)_2$	0.3	65	0.1	>98	**28**	8	x	30	
4	I	Bn	[Rh]	1	DBU (10.0)	0.1	x	30	$Mg(OMe)_2$	0.3	65	0.1	>98	**x**	x	x	x	Complex mixture
5	Br	Bn	[Rh]	1	x	x	20	30	$Mg(OMe)_2$	0.3	65	0.1	>98	**41**	x	x	17	
6	Br	Bn	[Rh]	1	NaI (0.25)	0.025	20	30	$Mg(OMe)_2$	0.3	65	0.1	>98	**39**	trace	x	25	
7	Br	Bn	[Rh]	1	NaI (0.50)	0.050	20	30	$Mg(OMe)_2$	0.3	65	0.1	>98	**37**	2	x	28	
8	Br	Bn	[Rh]	1	NaI (0.75)	0.075	20	30	$Mg(OMe)_2$	0.3	65	0.1	>98	**33**	7	x	32	
9	Br	Bn	[Rh]	1	NaI (1.00)	0.1	20	30	$Mg(OMe)_2$	0.3	65	0.1	>98	**28**	7	x	32	
10	Br	Bn	[Rh]	1	x	x	30	30	$Mg(OMe)_2$	0.3	65	0.1	>98	**40**	x	x	16	
11	Br	p-OTIPS	[Rh]	1	x	x	x	30	Na_2CO_3	1.0	65	0.1	>98	**<25**	5	x	14	
12	Br	p-OTIPS	[Rh]	1	x	x	x	30	K_2CO_3	1.0	65	0.1	>98	**<15**	6	x	12	
13	Br	p-OTIPS	[Rh]	1	x	x	x	30	K_3PO_4	1.0	65	0.1	>98	**<15**	6	x	13	
14	Br	p-OTIPS	[Rh]	1	x	x	20	30	$Mg(OMe)_2$	0.3	65	0.1	>98	**41**	trace	x	15	38 % isolated
15	I	p-OTIPS	[Rh]	1	x	0.1	20	30	$Mg(OMe)_2$	0.3	65	0.1	>98	**22**	8	x	30	

The screening reactions were run on 0.25 mmol of substrate in 10 mL sealed microwave vials.

[a] NMR Yield. [b] For the *p*-OTIPS salts the NMR yield is calculated as the sum of the protected and deprotected product (some product deprotects under the reaction conditions).

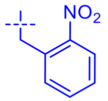

= o-NO_2

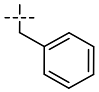

= Bn

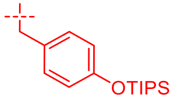

= p-OTIPS

[Ir]= $[IrCp^*Cl_2]_2$
[Rh]= $[RhCp^*Cl_2]_2$
[Ru]= $[Ru(p\text{-}cymene)Cl_2]_2$

Table S4. Screening and Optimisation

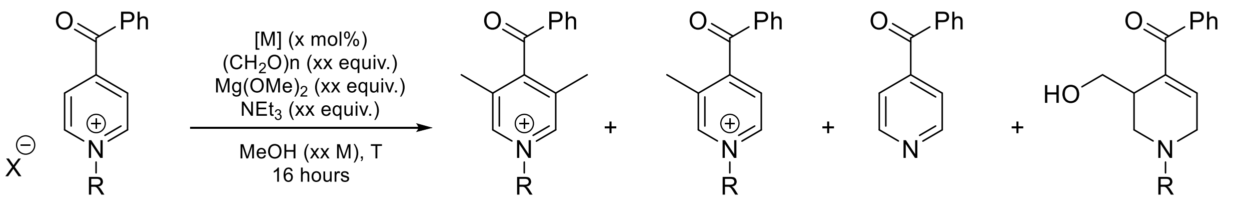

2 (1.0 equiv) → **3** + **F** + **1** + **5**

No.	X	R	M (mol%)		add. (equiv.)	Total iodide conc. (M)	NEt_3 equiv.	CH_2O equiv.	Base (equiv.)		T (°C)	Conc. of 1 (M)	Consumption of 2[a]	3[a,b]	F[a]	1[a]	5[a]	Comments
1	Br	p-OTIPS	[Rh]	1	x	x	20	30	$Mg(OMe)_2$	0.3	65	0.1	>98	**9**	8	5	x	severe degradation
2	I	p-OTIPS	[Rh]	1	x	0.1	20	30	$Mg(OMe)_2$	0.3	65	0.1	>98	**23**	5	x	x	moderate degradation
3	I	p-OTIPS	[Rh]	1	NaI (1.0)	0.2	20	30	$Mg(OMe)_2$	0.3	65	0.1	>98	**26**	3	x	x	
4	Br	p-OTIPS	[Rh]	1	NaI (4.0)	0.4	20	30	$Mg(OMe)_2$	0.3	65	0.1	>98	**33**	3	x	x	
5	Br	p-OTIPS	[Rh]	1	x	x	20	30	$Mg(OMe)_2$	0.3	40	0.1	>98	**12**	4	4	x	Severe degradation
6	Br	p-OTIPS	[Rh]	1	NaI (4.0)	0.4	20	30	$Mg(OMe)_2$	0.3	40	0.1	>98	**39**	trace	x	x	Moderate degradation
7	Br	p-OTIPS	[Rh]	1	NaI (1.0)	0.1	20	30	$Mg(OMe)_2$	0.3	40	0.1	>98	**37**	trace	x	x	Moderate degradation
8	Br	p-OTIPS	[Rh]	1	NaI (2.0)	0.2	20	30	$Mg(OMe)_2$	0.3	40	0.1	>98	**44**	x	x	x	Moderate degradation
9	Br	p-OTIPS	[Rh]	1	NaI (2.0)	0.2	20	30	$Mg(OMe)_2$	0.3	22	0.1	>98	**9**	20	x	x	Reaction is slow at RT
10	I	p-OTIPS	[Rh]	1	x	0.1	20	30	$Mg(OMe)_2$	0.3	40	0.1	>98	**36**	trace	x	x	Moderate degradation
11	I	p-OTIPS	[Rh]	1	NaI (1.0)	0.2	20	30	$Mg(OMe)_2$	0.3	40	0.1	>98	**44**	x	x	x	41 % isolated
12	I	p-OTIPS	[Rh]	1	NaI (1.0)	0.2	20	40	$Mg(OMe)_2$	0.3	40	0.1	>98	**43**	x	x	x	
13	I	p-OTIPS	[Rh]	1	NaI (1.0)	0.2	5	30	$Mg(OMe)_2$	0.3	40	0.1	>98	**36**	trace	x	x	
14	I	p-OTIPS	[Rh]	1	NaI (2.0)	0.3	5	30	$Mg(OMe)_2$	0.3	40	0.1	>98	**34**	trace	x	x	

The screening reactions were run on 0.25 mmol of substrate in 10 mL sealed microwave vials.

[a] NMR Yield. [b] For the *p*-OTIPS salts the NMR yield is calculated as the sum of the protected and deprotected product (some product deprotects under the reaction conditions).

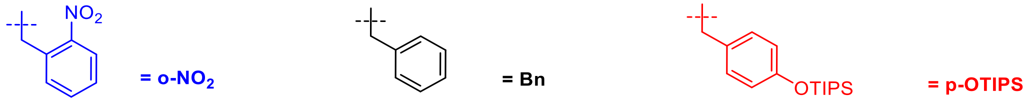

[Rh]= $[RhCp^*Cl_2]_2$

Preparation of the Alkylating Reagents

(4-(Bromomethyl)phenoxy)triisopropylsilane (S1)

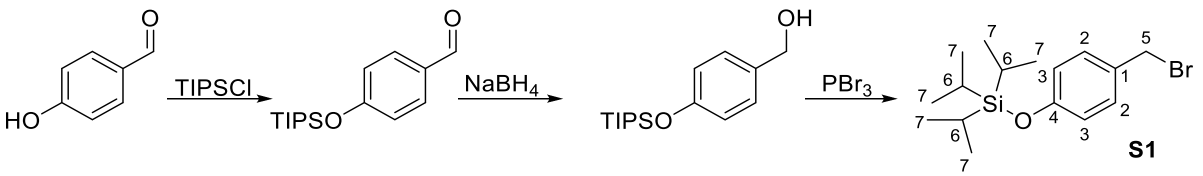

To a flask was added 4-hydroxybenzaldehyde (6.1 g, 50 mmol, 1.0 equiv.), imidazole (5.0 g, 75 mmol, 1.5 equiv.), triisopropylsilyl chloride (13 mL, 60 mmol, 1.2 equiv.) and 150 mL of CH_2Cl_2. The reaction was stirred at 40 °C under an atmosphere of argon for 16 hours. The reaction was allowed to cool to room temperature, then filtered through a pad of silica with CH_2Cl_2. The filtrate was concentrated *in vacuo*, and the crude product was dissolved in 100 mL of ethanol. To the reaction was added sodium borohydride (4.0 g, 100 mmol, 2.0 equiv.) in portions at room temperature. The reaction was left stirring at room temperature for 2-3 hours. To the reaction was added 100 mL of water and then 250 mL of brine and 150 mL of ethyl acetate. The aqueous layer was further extracted twice with ethyl acetate. The combined organic layers were dried over $MgSO_4$ and concentrated *in vacuo*. The crude product was dissolved in 500 mL of dry Et_2O and cooled to 0 °C using an ice bath. To the reaction was added PBr_3 (5.7 mL, 60 mmol, 1.2 equiv.) slowly at 0 °C. The reaction was left to stir for 30 minutes in the ice bath. The reaction was added to a cold saturated solution of $NaHCO_3$ and extracted with Et_2O. The ether layer was dried over $MgSO_4$, concentrated under vacuum, and then filtered through a pad of silica with pentane/Et_2O (95:5) to give the product **S1** as a colourless oil (14.6 g, 42.5 mmol, 85% yield over 3 steps). The spectroscopic data was consistent with previous literature reports.[1]

[1] Otto, N.; Ferenc, D.; Opatz, T. *J. Org. Chem*, **2017**, 82, 1205 - 1217

^{1}H NMR (400 MHz, $CDCl_3$) δ 7.25 (2H, d, *J* = 8.6 Hz, 2 x C^{2}**H**), 6.83 (2H, d, *J* = 8.6 Hz, 2 x C^{3}**H**), 4.49 (2H, s, C^{5}**H_2**), 1.31 – 1.18 (3H, m, 3 x C^{6}**H**), 1.09 (18H, d, *J* = 7.3 Hz, 6 x C^{7}**H_3**);

^{13}C NMR (101 MHz, $CDCl_3$) δ 156.4 (C), 130.5 (2 x C^{2}), 130.3 (C), 120.2 (2 x C^{3}), 34.3 (C^{5}), 18.0 (6 x C^{7}), 12.8 (3 x C^{6}).

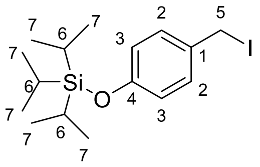

(4-(Iodomethyl)phenoxy)triisopropylsilane (S2)

To a stirred solution of sodium iodide (4.5 g, 30 mmol, 2.0 equiv.) in 40 mL of acetone was slowly added (4-(bromomethyl)phenoxy)triisopropylsilane (5.1 g, 15 mmol, 1.0 equiv.) at room temperature. The reaction was stirred at room temperature in the dark overnight. To the reaction was added 50 mL of brine and then extracted with 2 x Et_2O (100 mL). The organic layer was dried over $MgSO_4$ and concentrated *in vacuo* to give the product **S2** (11.7 g) as a brown liquid in quantitative yield. The spectroscopic data was consistent with previous literature reports.[2]

^{1}H NMR (400 MHz, $CDCl_3$) δ 7.24 (2H, d, *J* = 8.6 Hz, 2 x C^{2}**H**), 6.79 (2H, d, *J* = 8.5 Hz, 2 x C^{3}**H**), 4.46 (2H, s, C^{5}**H_2**), 1.32 – 1.14 (3H, m, 3 x C^{6}**H**), 1.09 (18H, d, *J* = 7.3 Hz, 6 x C^{7}**H_3**);

^{13}C NMR (101 MHz, $CDCl_3$) δ 155.9 (C), 131.7 (C), 130.1 (2 x C^{2}), 120.3 (2 x C^{3}), 18.0 (6 x C^{7}), 12.8 (3 x C^{6}), 7.0 (C^{5}).

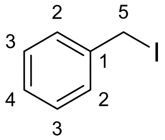

Benzyl iodide (S3)

Sodium iodide (18 g, 120 mmol, 2 equiv.) was dissolved in 80 mL of acetone at 0 °C. To the reaction was slowly added benzyl bromide (7.2 mL, 60 mmol, 1 equiv.) and the reaction was left to stir at room temperature overnight in the dark. To the reaction was added 150 mL of brine and extracted with 2 x 100 mL of diethyl ether. The combined organics were dried over $MgSO_4$ and concentrated *in vacuo*. The crude material was filtered through silica

[2] Lee, H. Y.; Jiang, X.; Lee, D. *Org. Lett.* **2009**, 11, 2065 - 2068

pentane: Et_2O (95:5) to give the product **S3** as a brown oil in quantitative yield. The spectroscopic data was consistent with previous literature reports.[3]

^{1}H NMR (400 MHz, $CDCl_3$) δ 7.44 – 7.21 (5H, m, 5 x Ar-C**H**), 4.47 (2H, s, C^5**H_2**);

^{13}C NMR (101 MHz, $CDCl_3$) δ 139.1 (C^1), 128.6 (2 x C^{Ar}), 128.5 (C^{Ar}), 127.7 (2 x C^{Ar}), 5.58 (C^5).

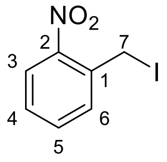

1-(Iodomethyl)-2-nitrobenzene (S4)

To a stirred solution of sodium iodide (9.0 g, 60mmol, 2.0 equiv.) in 40 mL of acetone was slowly added 1-(bromomethyl)-2-nitrobenzene (6.48 g, 30 mmol, 1.0 equiv.) at room temperature. The reaction was stirred at room temperature in the dark for 16 hours. To the reaction was added 50 mL of brine and the solution was extracted with 2 x Et_2O (100 mL). The organic layer was dried over $MgSO_4$ and concentrated under vacuum to give the product **S4** (7.90 g) as a light brown solid in quantitative yield. The spectroscopic data was consistent with previous literature reports.[4]

m.p.: 74-76 °C;

^{1}H NMR (400 MHz, $CDCl_3$) δ 8.01 (1H, d, J = 8.2 Hz, C^3**H**), 7.60 – 7.47 (2H, m, Ar-C**H**), 7.43 (1H, ddd, J = 8.7, 7.1, 1.7 Hz, Ar-C**H**), 4.77 (2H, s, C^7**H_2**);

^{13}C NMR (101 MHz, $CDCl_3$) δ 147.5 (C^2), 135.0 (C^1), 133.9 (C^{Ar}), 132.3 (C^{Ar}), 129.1 (C^{Ar}), 125.8 (C^{Ar}), 0.00 (C^7).

---

[3] Hoang, C. T.; Alezra, V.; Guillot, R.; Kouklovsky, C. *Org. Lett.*, **2007**, 9, 2521 - 2524

[4] Alajarin, M.; Pastor, A.; Orenes, R.-A.; Steed, J. W.; Arakawa, R. *Chem. Eur. J.* **2004,** 10, 1383 - 1397

Preparation of the Pyridine Precursors

4-Phenylpyridine (**1a**), 4-pyidylpyridine (**1u**) and phenyl(pyridin-4-yl)methanone (**1k**) are commercially available. 4-(3,5-Bis(trifluoromethyl)phenylpyridine (**1i**) was prepared as previously reported.[5]

General Procedure A

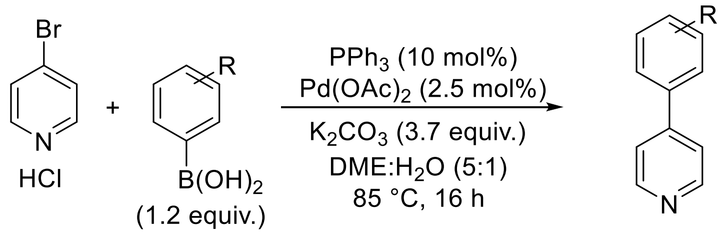

4-Bromopyridine hydrochloride (1.94 g, 10 mmol), triphenylphosphine (262 mg, 1 mmol), boronic acid (1.2 equiv.), and potassium carbonate (5.1 g, 37 mmol) were added to a 3-necked flask fitted with a reflux condenser. The vessel was evacuated and backfilled with argon three times then dimethoxyethane (50 mL) and water (10 mL) were added and solution purged with argon for 10 minutes. Palladium(II) acetate (56 mg, 0.25 mmol) was added and the solution heated at 85 °C for 16 hours. The solution was cooled, diluted with 50 mL of water, and extracted with EtOAc (50 mL) three times. The organic layers were combined, dried ($MgSO_4$), filtered, and concentrated *in vacuo*. The crude material was purified by flash column chromatography to give the product.

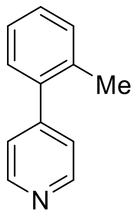

4-(2-Methyphenyl)pyridine (1b)

The title compound was prepared according to General Procedure A using 2-methylphenylboronic acid (1.63 g, 12 mmol) and purified by FCC (10%-20% EtOAc in pentane) to give *pyridine* **1b** (0.94 g, 55% yield) as a colourless oil. The spectroscopic data was consistent with previous literature reports.[6]

[5] Grozavu, A.; Hepburn, H. B.; Smith, P. J.; Potukuchi, H. K.; Lindsay-Scott, P. J.; Donohoe, T. J. *Nat. Chem.* **2019**,
[6] Zhang, X.; McNally, A. *Angew. Chem. Int. Ed.* **2017**, *56*, 9833-9836.

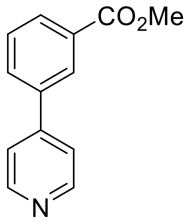

3-Pyridin-4-yl benzoic acid methyl ester (1c)

The title compound was prepared according to General Procedure A using 3-methoxycarbonylphenylboronic acid (2.16 g, 12 mmol) and purified by FCC (30%-50% EtOAc in pentane) to give *pyridine* **1c** (1.53 g, 72% yield) as a white solid. The spectroscopic data was consistent with previous literature reports.[7]

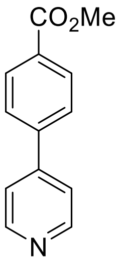

4-Pyridin-4-yl benzoic acid methyl ester (1d)

The title compound was prepared according to General Procedure A using 4-methoxycarbonylphenylboronic acid (2.16 g, 12 mmol) and purified by FCC (20%-40% EtOAc in pentane) to give *pyridine* **1d** (1.60 g, 76% yield) as a cream solid. The spectroscopic data was consistent with previous literature reports.[8]

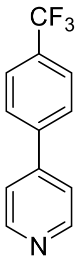

4-(4-Trifluoromethyl)pyridine (1e)

The title compound was prepared according to General Procedure A using 4-trifluoromethylbenzene boronic acid (2.28 g, 12 mmol) and was purified by FCC (30% -50% EtOAc in pentane) to give *pyridine* **X1e** (1.70 g, 76% yield) as a yellow glass. The spectroscopic data was consistent with previous literature reports.[2]

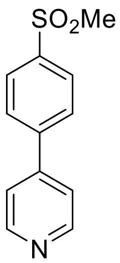

4-(4-Methylsulfonylphenyl)pyridine (1f)

The title compound was prepared according to General Procedure A using 4-methysulfonylphenylboronic acid (2.40 g, 12 mmol) and purified by FCC (10%-

[7] Eisai Co. Ltd. *EP1394147*, **2004**, A1 and Ohkura, K.; Terashima, M.; Kanaoka, Y.; Seki, K. *Chem. Pharm. Bull.* **1993**, *41*, 1920-1924.
[8] Malineni, J.; Jezorek, R. L.; Zhang, N.; Percec, V. *Synthesis*, **2016**, *48*, 2795-2807.

20% acetone in CH_2Cl_2) to give *pyridine* **1f** (1.56 g, 67% yield) as a white solid. The spectroscopic data was consistent with previous literature reports.[9]

arylpyridines **1b-j**.

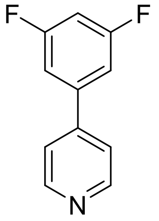

4-(3,5-Difluorophenyl)pyridine (1g)

The title compound was prepared according to General Procedure A using 3,5-difluorobenzne boronic acid (1.9 g, 12 mmol) and was purified by FCC (10% - 20% EtOAc in pentane) to give *pyridine* **1g** (1.67 g, 87% yield) as a cream solid. The spectroscopic data was consistent with previous literature reports.[10]

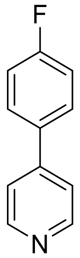

4-(4-Fluorophenyl)pyridine (1h)

The title compound was prepared according to General Procedure A using 4-fluorobenzene boronic acid (1.68 g, 12 mmol) and was purified by FCC (10% - 20% EtOAc in pentane) to give *pyridine* **1h** (1.56 g, 90% yield) as a colourless glass. The spectroscopic data was consistent with previous literature reports.[11]

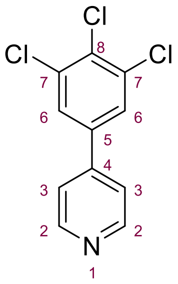

4-(3,4,5-Trichloropheny)pyridine (1j)

The title compound was prepared using General Procedure A on a 2 mmol scale using 3,4,5-trichlorobenzne boronic acid (540 mg, 2.4 mmol) and purified by FCC (30%-50% EtOAc in pentane) to give *pyridine* **1j** (426 mg, 82% yield) as a white solid.

m.p. (Et_2O): 159-161 °C;

[9] Pettersson, F.; Svensson, P.; Waters, S.; Waters, N.; Sonesson, C. *J. Med. Chem.* **2012**, *55*, 3242-3249.
[10] Zhang, X.; McNally, A. *Angew. Chem. Int. Ed.* **2017**, *56*, 9833-9836.
[11] Panda, S.; Coffin, A.; Nguyen, Q. N.; Tantillo, D. J.; Ready, J. M. *Angew. Chem. Int. Ed.* **2016**, *55*, 2245-2249

HRMS (ESI): Exact mass calculated for $C_{11}H_7N^{35}Cl_3$ $[M+H]^+$ calc: 257.96386, found: 257.96405.

^{1}H NMR ($CDCl_3$) δ 8.94-8.48 (2H, m, 2 x C^2**H**), 7.64 (2H, s, 2 x C^6**H**), 7.54-7.28 (2H, m, 2 x C^3**H**);

^{13}C NMR ($CDCl_3$) δ 150.6 (2 x C^2), 144.8 (C^4), 138.1 (C^8), 135.0 (2 x C^7), 132.2 (C^5), 127.0 (2 x C^6), 121.2 (2 x C^3);

IR (neat): 3043, 2161, 1601, 1584, 1536, 1431, 1379, 1328, 1208, 1169, 1034, 994 cm^{-1}.

General Procedure B1

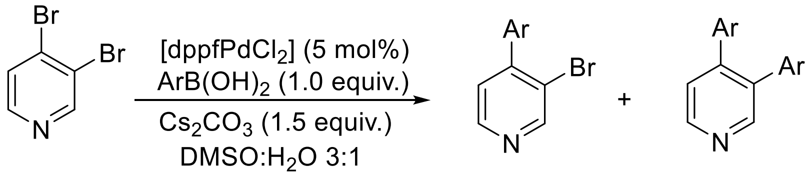

3,4-Dibromopyridine (1.0 equiv.), cesium carbonate (1.5 equiv.), and arylboronic acid (1.0 equiv.) were dissolved in a 3:1 mixture of DMSO;H_2O (5 mL total per mmol of aryl bromide) which was then purged with Ar for 10 minutes. [(dppf)$PdCl_2$] (5 mol%) was added and the solution heated for 16 hours at 80 °C. The solution was cooled, diluted with EtOAc and extracted three times with EtOAc. The combined organic layers were washed with brine, dried ($MgSO_4$), filtered and concentrated *in vacuo*. The crude material was purified by flash column chromatography eluting 4-arylpyridine first followed by 3,4-diarylpyridine.

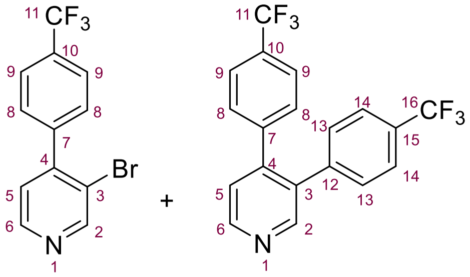

3-Bromo-4-(4-trifluoromethyl)phenylpyridine (S5) and 3,4-bis(4-trifluromethyl)phenylpyridine (1t). The title compound were prepared according to General Procedure B1 using 4-trifluoromethylbenzene boronic acid (1.33 g, 7 mmol) and was purified by FCC (10-30% EtOAc in pentane) to give

arylpyridine **S5** (840 mg, 40%) as a yellow oil followed by *bisarylpyridine* **1t** (297 mg, 23% yield) as a yellow glass.

3-Bromo-4-(4-trifluoromethyl)phenylpyridine (S5):

HRMS (ESI): Exact mass calculated for $C_{12}H_8N^{79}BrF_3$ $[M+H]^+$ calc: 301.97867, found: 301.97873;

^{1}H NMR ($CDCl_3$) δ 8.86 (1H, s, C^2**H**), 8.60 (1H, dd, *J* = 4.9, 0.9 Hz, C^6**H**), 7.75 (2H, d, *J* = 8.0 Hz, 2 x C^9**H**), 7.57 (2H, d, *J* = 8.0 Hz, 2 x C^8**H**), 7.34-7.07 (1H, m, C^5**H**);

^{13}C NMR ($CDCl_3$)) δ 152.7 (C^2), 148.5 (C^6), 148.3 (C), 141.6 (C), 130.9 (q, *J* = 32.6 Hz, C^{10}), 129.3 (2 x C^8), 125.4 (q, *J* = 3.8 Hz, 2 x C^9), 125.3 (C^5), 123.9 (q, *J* = 272.3 Hz, C^{11}), 120.5 (C);

^{19}F NMR ($CDCl_3$) δ –62.7;

IR (neat): 1582, 1395, 1321, 1261, 1165, 1123, 1069, 1021, 828, 741, 696, 608 cm^{-1}.

3,4-bis(4-Triflurometh yl)phenylpyridine (1t):

m.p. (Et_2O): 78-80 °C;

HRMS (ESI): Exact mass calculated for $C_{19}H_{12}NF_6$ $[M+H]^+$ calc: 368.08685, found: 368.08685;

^{1}H NMR ($CDCl_3$) δ 8.73 (1H, d, *J* = 5.1 Hz, C^6**H**), 8.68 (1H, s, C^2**H**), 7.58 (4H, d, *J* = 8.1 Hz, 2 x C^9**H** + 2 x C^{14}**H**), 7.38 (1H, d, *J* = 5.3 Hz, C^5**H**), 7.28 (4H, d, *J* = 8.1 Hz, 2 x C^8**H** + 2 x C^{13}**H**);

^{13}C NMR ($CDCl_3$) δ 151.0 (C^2), 149.6 (C^6), 146.3 (C), 141.7 (C), 140.8 (C), 134.4 (C), 130.3 (q, *J* = 33.1 Hz, C), 130.1 (2 x C^8), 129.7 (q, *J* = 33.0 Hz, C), 129.6 (2 x C^{13}), 125.5 (app p, *J* = 3.7 Hz, 2 x C^9 + 2 x C^{14}), 124.5 (C^5), 123.9 (q, *J* = 272.3 Hz, C^{11}), 123.8 (q, *J* = 232.0 Hz, C^{16});

^{19}F NMR ($CDCl_3$) δ –62.6, –62.7

IR (neat): 1619, 1589, 1493, 1405, 1322, 1280, 1243, 1163, 1106, 1068, 1014, 851 cm^{-1}.

General Procedure B2

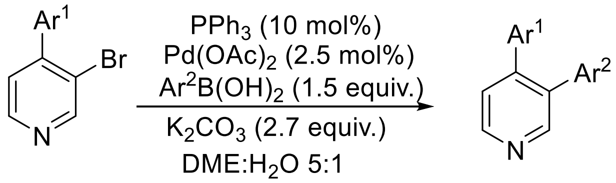

3-Bromo-4-arylpyridine (1.0 equiv.), triphenylphosphine (10 mol%), potassium carbonate (2.7 equiv.), and arylboronic acid (1.5 equiv.) were dissolved in a 5:1 mixture of DME:H_2O (5 mL per mmol of aryl bromide) which was then purged with Ar for 10 minutes. $Pd(OAc)_2$ was added and the solution heated for 16 hours at 85 °C. The solution was cooled, diluted with EtOAC and extracted three times with EtOAC. The combined organic layers were washed with brine, dried ($MgSO_4$), filtered and concentrated *in vacuo*. The crude material was purified by flash column chromatography to give the product.

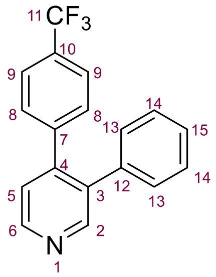

3-Phenyl-4-(4-trifluoromethyl)phenylpyridine (1l)

The title compound was prepared according to General Procedure B2 using 3-Bromo-4-(4-trifluoromethyl)phenylpyridine (755 mg, 2.5 mmol) and phenylboronic acid (458 mg, 3.75 mmol) and was purified by FCC (10-30% EtOAc:pentane) to give pyridine **1l** (673 mg, 90% yield) as a yellow oil which was around 90% pure and taken on without further purification.

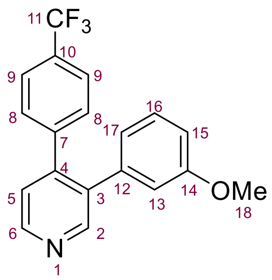

3-(3-Methoxyphenyl)-4-(4-trifluoromethyl)phenylpyridine (1p)

The title compound was prepared according to General Procedure B2 using 3-Bromo-4-(4-trifluoromethyl)phenylpyridine (755 mg, 2.5 mmol) and 3-methoxyphenylboronic acid (570 mg, 3.75 mmol) and was purified by FCC (10-30% EtOAc:pentane) to give pyridine **1p** (737 mg, 90% yield) as a yellow oil.

HRMS (ESI): Exact mass calculated for $C_{19}H_{15}ONF_3$ $[M+H]^+$ calc: 330.11003, found: 330.10995;

^{1}H NMR ($CDCl_3$) δ 8.62 (1H, d, *J* = 0.6 Hz, C^2**H**), 8.60 (1H, d, *J* = 5.1 Hz, C^6**H**), 7.47 (2H, d, *J* = 8.1 Hz, 2 x C^9**H**), 7.26 (1H, dd, *J* = 5.1, 0.7 Hz, C^5**H**), 7.23 (2H, d, *J* = 8.0 Hz, 2 x C^8**H**), 7.13 (1H, t, *J* = 7.9 Hz, C^{16}**H**), 6.77 (1H, ddd, *J* = 8.3, 2.6, 0.9 Hz, C^{15}**H**), 6.64 (1H, dt, *J* = 7.6, 1.2 Hz, C^{17}**H**), 6.59 (1H, dd, *J* = 2.6, 1.6 Hz, C^{13}**H**), 3.59 (3H, s, C^{18}**H_3**);

^{13}C NMR ($CDCl_3$)) δ 159.5 (C), 151.1 (C^2), 149.0 (C^3), 146.2 (C), 142.3 (C), 138.3 (C), 135.6 (C), 129.9 (q, *J* = 32.6 Hz, C^{10}), 129.6 (2 x C^8), 129.5 (C^{16}), 125.3 (q, *J* = 3.7 Hz, 2 x C^9), 124.3 (C^{17}), 124.0 (q, *J* = 272 Hz, C^{11}), 122.2 (C^{13}), 115.2 (C^{15}), 113.4 (C^5) 55.1 (C^{18});

^{19}F NMR ($CDCl_3$) δ –62.6

IR (neat): 1736, 1587, 1473, 1430, 1398, 1322, 1222, 1166, 1122, 1069, 1046, 1029 cm^{-1}.

General Procedure C

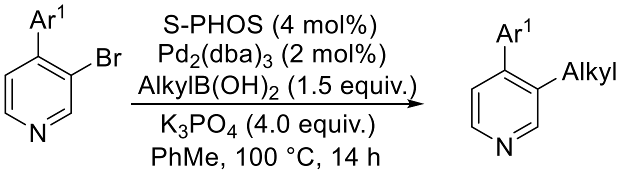

3-Bromo-4-arylpyridine (1.0 equiv.), S-PHOS (4 mol%), potassium phosphate (4.0 equiv.), and alkylboronic acid (2.0 equiv.) were dissolved in PhMe (8 mL per mmol of aryl bromide) which was then purged with Ar for 10 minutes. $Pd_2(dba)_3$ (2 mol%) was added and the solution heated for 16 hours at 100 °C. The solution was cooled, diluted with EtOAC and extracted three times with EtOAC. The combined organic layers were washed with brine, dried ($MgSO_4$), filtered and concentrated *in vacuo*. The crude material was purified by flash column chromatography to give the 3-alkyl-4-arylpyridine.

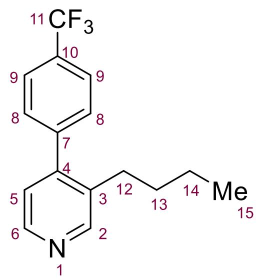

3-(Butyl)-4-(4-trifluoromethyl)phenylpyridine (1m)

The title compound was prepared according to General Procedure C using 3-bromo-4-(4-trifluoromethyl)phenylpyridine **S5** (755 mg, 2.5 mmol) and n-butylboronic acid (510 mg, 5 mmol) and was purified by FCC (10-30% EtOAc:pentane) to give pyridine **1m** (690 mg, 99% yield) as a yellow oil.

HRMS (ESI): Exact mass calculated for $C_{16}H_{17}NF_3$ $[M+H]^+$ calc: 280.13076, found: 280.13062;

^{1}H NMR ($CDCl_3$) δ 8.58 (1H, s, C^2**H**), 8.51 (1H, d, *J* = 5.1 Hz, C^6**H**), 7.73 (2H, d, *J* = 8.0 Hz, 2 x C^9**H**), 7.44 (2H, dt, *J* = 7.8, 0.8 Hz, 2 x C^8**H**), 7.14 (1H, dd, *J* = 5.1, 0.7 Hz, C^5**H**), 2.64-2,57 (2H, m, C^{12}**H**$_2$), 1.50-1.40 (2H, m, C^{13}**H**$_2$), 1.30-1.20 (2H, m, C^{14}**H**$_2$), 0.81 (3H, t, *J* = 7.3 Hz, C^{15}**H**$_3$);

^{13}C NMR ($CDCl_3$)) δ 150.2 (C^2), 148.2 (C), 146.4 (C^6), 142.6 (C), 135.5 (C), 128.9 (2 x C^8), 125.4 (q, *J* = 3.9 Hz, 2 x C^9), 124.2 (C^5), 33.1 (C^{12}), 29.9 (C^{13}), 22.3 (C^{14}), 13.9 (C^{15}) *quartets for C^{10} ≈ 130.0 (J ≈ 30 Hz) and C^{11} ≈ 124.0 (J ≈ 272. Hz) were not readily visible;*

^{19}F NMR ($CDCl_3$) δ –62.6;

IR (neat): 1714, 1490, 1463, 1401, 1355, 1321, 1247, 1168, 1111, 1048, 1043, 1020 cm^{-1}.

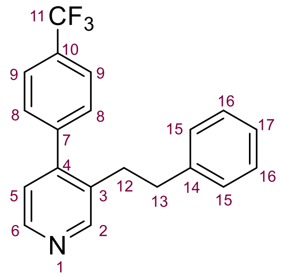

3-(2-Phenylethyl)-4-(4-trifluoromethyl)phenylpyridine 1o

The title compound was prepared according to General Procedure C using 3-bromo-4-(4-trifluoromethyl)phenylpyridine **S5** (755 mg, 2.5 mmol) and 2-phenylethylboronic acid (750 mg, 5 mmol) and was purified by FCC (20-50% EtOAc:pentane) then FCC (0-10% EtOAc:CH_2Cl_2 to give pyridine **1o** (485 mg, 59% yield) as a white solid.

m.p. (Et_2O): 74-76 °C;

HRMS (ESI): Exact mass calculated for $C_{20}H_{17}NF_3$ $[M+H]^+$ calc: 328.13076, found: 328.13065;

^{1}H NMR ($CDCl_3$) δ 8.57 (1H, s, C^2**H**), 8.52 (1H, d, *J* = 5.0 Hz, C^6**H**), 7.69 (2H, d, *J* = 8.0 Hz, 2 x C^9**H**), 7.31 (2H, d, *J* = 8.0 Hz, 2 x C^8**H**), 7.24-7.14 (3H, m, 2 x C^{15}**H** + C^{17}**H**), 7.11 (1H, d, *J* = 5.0 Hz, C^5**H**), 6.90 (2H, dd, *J* = 7.7, 1.7 Hz, 2 x C^{16}**H**), 2.92 (2H, dd, *J* = 9.2, 6.6 Hz, C^{12}**H**$_2$), 2.74 (2H, dd, *J* = 9.2, 6.5 Hz, C^{13}**H**$_2$),

^{13}C NMR ($CDCl_3$)) δ 151.0 (C^2), 148.1 (C), 147.4 (C^6), 142.5 (C), 140.4 (C)¸134.1 (C), 130.2 (q, *J* = 32.6 Hz, C^{10}), 128.9 (2 x C^8), 128.4 (2 x C^{15}), 128.3 (2 x C^{16}), 126.2 (C^{17}), 125.4 (q, *J* = 3.9 Hz, 2 x C^9), 124.0 (C^5), 123.9 (q, *J* = 272.7 Hz, C^{11}), 37.2 (C^{13}), 32.1 (C^{12});

^{19}F NMR ($CDCl_3$) δ –62.6;

IR (neat): 1589, 1494, 1454, 1403, 1324, 1169, 1117, 1070, 1030, 1012, 847, 836 cm^{-1}.

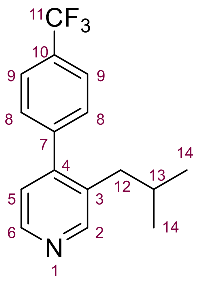

3-(2-Methylpropyl)-4-(4-trifluoromethyl)phenylpyridine (1q)

The title compound was prepared according to General Procedure C using 3-bromo-4-(4-trifluoromethyl)phenylpyridine **S5** (755 mg, 2.5 mmol) and 2-methylpropylboronic acid (510 mg, 5 mmol) and was purified by FCC (10-30% EtOAc:pentane) then FCC (0-7% EtOAc:CH_2Cl_2) to give pyridine **1q** (445 mg, 64% yield) as a yellow oil.

HRMS (ESI): Exact mass calculated for $C_{16}H_{17}NF_3$ $[M+H]^+$ calc: 280.13076, found: 280.13071;

^{1}H NMR ($CDCl_3$) δ 8.53 (1H, s, C^2**H**), 8.50 (1H, d, *J* = 5.0 Hz, C^6**H**), 7.72 (2H, dt, *J* = 7.9, 0.7 Hz, 2 x C^9**H**), 7.42 (2H, dt, *J* = 7.9, 0.8 Hz, 2 x C^8**H**), 7.11 (1H, dd, *J* = 5.0, 0.7 Hz, C^5**H**), 2.51 (2H, d, *J* = 7.3 Hz, C^{12}**H**$_2$), 1.64 (1H, app hept *J* = 6.7 Hz, C^{13}**H**), 0.75 (6H, d, *J* = 6.6 Hz, 2 x C^{14}**H**$_3$);

^{13}C NM0R ($CDCl_3$)) δ 151.2 (C^{2}), 148.2 (C), 147.2 (C^{6}), 143.0 (C), 134.1 (C), 130.1 (q, *J* = 32.7 Hz, C^{10}), 129.0 (2 x C^{8}), 125.4 (q, *J* = 3.9 Hz, 2 x C^{9}), 122.7 (C^{5}), 121.3 (q, *J* = 272.3 Hz, C^{11}), 39.1 (C^{12}), 29.6 (C^{13}), 22.3 (2 x C^{14});

^{19}F NMR ($CDCl_3$) δ –62.5;

IR (neat): 1590, 1403, 1323, 1165, 1124, 1107, 1069, 1030, 1015, 833, 814, 722 cm^{-1}.

3-Fluoro-4-(4-trifluoromethyl)phenylpyridine (S6)

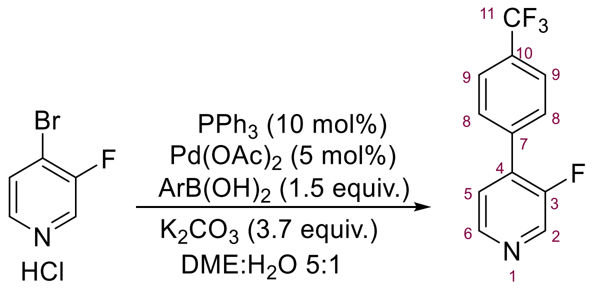

3-Fluoro-4-bromopyridine hydrochloride (2.00 g, 9.4 mmol), 4-trifluorophenylboronic acid (1.79 g, 9.4 mmol), triphenylphosphine (262 mg, 1 mmol), and potassium carbonate (4.8 g, 35 mmol) were added to a three-neck flask fitted with a condenser. DME (50 mL) and water (10 mL) were added the solution was sparged with argon for 15 minutes. Palladium acetate (44 mg, 2.5 mol%) was added and the solution was heated at 85 °C for 16 hours under argon. The solution was cooled, diluted with EtOAc and extracted three times with EtOAc. The combined organic layers were washed with brine, dried ($MgSO_4$), filtered and concentrated *in vacuo*. The crude material was purified by flash column chromatography (10-30% EtOAc:pentane) to give *pyridine* **S6** (1.57 g, 66% yield) as a yellow oil which was 95% pure and used without further purification.

^{1}H NMR ($CDCl_3$) δ 8.60 (1H, d, *J* = 2.4 Hz, C^{6}**H**), 8.53 (1H, d, *J* = 4.9 Hz, C^{2}**H**), 7.77 (2H, d, *J* = 8.6 Hz, 2 x C^{9}**H**), 7.73 (2H, d, *J* = 7.7 Hz, 2 x C^{8}**H**), 7.42 (1H, dd, *J* = 6.6, 5.0 Hz, C^{5}**H**);

^{13}C NMR ($CDCl_3$) δ 156.4 (d, J = 258.8 Hz, C^3), 146.2 (d, J = 5.4 Hz, C^6), 139.2 (d, J = 25.5 Hz, C^2), 136.4 (C), 134.7 (d, J = 10.1 Hz. C^4), 131.3 (q, J = 32.8 Hz, C^{10}), 129.2 (d, J = 3.3 Hz, 2 x C^8), 125.8 9q, J = 3.6 Hz, 2 x C^9), 124.0 (C^5), 123.8 (q, J = 272.6 Hz, C^{11});

^{19}F NMR ($CDCl_3$) δ –62.0 (CF_3), –132.6 (Ar-F);

3-Methoxy-4-(4-trifluoromethyl)phenylpyridine (1n)

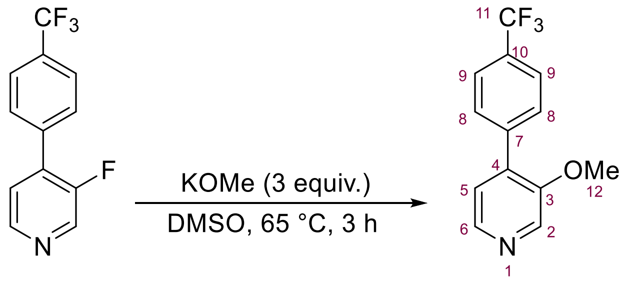

3-Fluoro-4-(4-trifluoromethyl)phenylpyridine **S6** (0.241 g, 1 mmol) and potassium methoxide (210 mg, 3 mmol) were dissolved in DMSO (3 mL) and heated to 65 °C for 3 hours. The solution was cooled and diluted by the addition of EtOAc (20 mL) and water (20 mL). The solution was separated and then the aqueous layer was extracted with EtOAc (2 x 20 mL). The combined organic layers were washed with brine (50 mL), dried ($MgSO_4$), filtered, and concentrated *in vacuo*. The crude material was purified by flash column chromatography (0-20% acetone/CH_2Cl_2) to give *pyridine* **1n** (200 mg, 79% yield) as a cream solid

m.p. (Et_2O): 54-56 °C;

HRMS (ESI): Exact mass calculated for $C_{13}H_{11}ONF_3$ $[M+H]^+$ calc: 254.07873, found: 254.07877;

^{1}H NMR ($CDCl_3$) δ 8.42 (1H, s, C^2**H**), 8.36 (1H, d, J = 4.8 Hz, C^6**H**), 7.71 (2H, d, J = 9.0 Hz, 2 x C^9**H**), 7.68 (2H, d, J = 8.7 Hz, 2 x C^8**H**), 7.25 (1H, d, J = 4.7 Hz, C^5**H**), 3.95 (3H, s, C^{12}**H**$_3$);

^{13}C NMR ($CDCl_3$) δ 152.4 (C), 143.1 (C^6), 139.4 (C)¸136.0 (C)¸134.6 (C^2), 130.3 (q, J = 32.5 Hz, C^{10}), 129.6 (2 x C^8), 125.2 (q, J = 3.9 Hz, 2 x C^9), 124.2 (C^5), 124.1 (q, J = 272.4 Hz, C^{11}), 56.3 (C^{12});

^{19}**F NMR** ($CDCl_3$) δ –62.7;

IR (neat): 1619, 1591, 1493, 1470, 1418, 1405, 1327, 1280, 1243, 1168, 1107, 1069 cm^{-1}.

2-Chloro-4-(4-pyridyl)pyridine (S7)

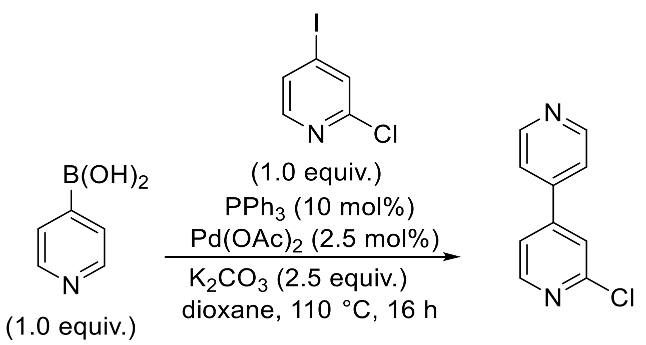

2-Chloro-4-iodopyridine (1.91 g, 8 mmol), 4-pyridylboronic acid (984 mg, 8 mmol), potassium carbonate (2.98 g, 21.6 mmol), and triphenylphosphine (209 mg, 0.8 mmol) were dissolved in dioxane (50 mL) which was then purged with Ar for 10 minutes. $Pd(OAc)_2$ (45 mg, 2.5 mol%) was added and the solution heated for 16 hours at 110 °C. The solution was cooled, diluted with EtOAc and extracted three times with EtOAc. The combined organic layers were washed with brine, dried ($MgSO_4$), filtered and concentrated *in vacuo*. The crude material was purified by flash column chromatography (0-100% ethyl acetate in pentane) eluting 2-chloro-4-(4-pyridyl)pyridine (947 mg, 62% yield) **S7** as a peach solid. The spectroscopic data was consistent with previous literature reports.[12]

2-Phenyl-4-(4-pyridyl)pyridine (1v)

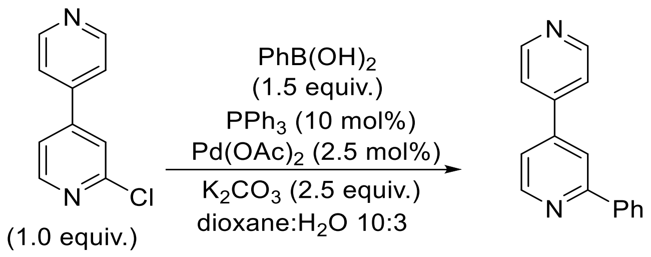

[12] Ohmura, T.; Morimasa, Y.; Suginome, M. *J. Am. Chem. Soc.* **2015**, *137*, 2852-2855.

2-Chloro-4-(4-pyridyl)pyridine (381 mg, 2 mmol), phenylboronic acid (266 mg, 3 mmol), potassium carbonate (690 mg, 5 mmol), and triphenylphosphine (52 mg, 0.2 mmol) were dissolved in a mixture of dioxane/water (10 mL + 3 mL) which was then purged with Ar for 10 minutes. $Pd(OAc)_2$ (11.2 mg, 2.5 mol%) was added and the solution heated for 16 hours at 110 °C. The solution was cooled, diluted with EtOAc and extracted three times with EtOAc. The combined organic layers were washed with brine, dried ($MgSO_4$), filtered and concentrated *in vacuo*. The crude material was purified by flash column chromatography (0-10% acetone in CH_2Cl_2) eluting 2-phenyl-4-(4-pyridyl)pyridine 455 mg, 98% yield) **1v** as a colourless glass. The spectroscopic data was consistent with previous literature reports.[13]

4-(2-Pyrimidine)pyridine (1w)

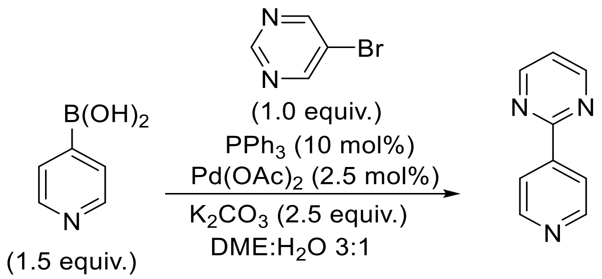

4-Pyridiylboronic acid (922.5 mg, 7.5 mmol) potassium carbonate (1.725 g, 12.5 mmol) triphenylphosphine (131 mg, 0.5 mmol) and 5-bromopyrimdine (790 mg, 5 mmol) were dissolved in a (25:10 mL) mixture of DME:H_2O which was then purged with Ar for 10 minutes. $Pd(OAc)_2$ (28 mg, 2.5 mol%) was added and the solution heated for 16 hours at 80 °C. The solution was cooled, diluted with EtOAc and extracted three times with EtOAc. The combined organic layers were washed with brine, dried ($MgSO_4$), filtered and concentrated *in vacuo*. The crude material was purified by flash column chromatography (0-20% acetone in CH_2Cl_2)

[13] *Zeitschrift fur Naturforschung, B: Chemical Sciences*, **1988**, *43*, 475 – 482.

eluting 4-heteroarylpyridine (403 mg, 51% yield) **1w** as a brown solid. The spectroscopic data was consistent with previous literature reports.[14]

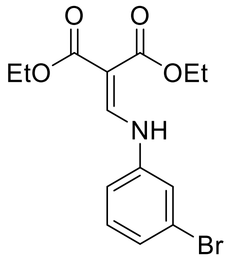

Ethyl α-carbethoxy-β-*m*-bromoanilinoacrylate (S8)

3-Bromoaniline (10 g, 58.1 mmol) and diethyl methoxymethylenemalonate (14 mL, 63.9 mmol) were heated together at 110 °C for 1 hour. The solution was cooled to room temperature which formed a solid, which was broken up and suspended in pentane. The suspension was filtered, washed with pentane, and dried under vacuum to yield *acrylate* **S8** (19.2 g, 97% yield) as a white solid. Spectroscopic data matched those previous reported.[15]

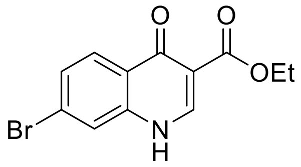

7-Bromo-4-oxoquinoline-3-carboxylic acid ethyl ester (S9)

Dowtherm A© (50 mL) was heated to 250 °C and Compound **S8** (10.0 g, 29 mmol) was added portionwise. The solution was heated at 250 °C for 1 hour before being cooled slowly to room temperature. The resulting suspension was filtered, washed with pentane and dried under vacuum to yield *heterocycle* **S9** (6.99 g, 79% yield) as a colourless solid. Literature NMR spectra in d^6-DMSO are available. However, in our hands, **S9** was insoluble in DMSO and all other deuterated solvents, no NMR spectra could be obtained and the compound was taken onto to the next step.

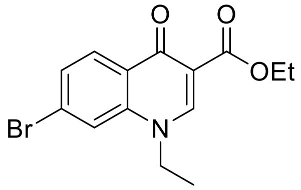

***N*-Ethyl-7-bromo-4-oxoquinoline-3-carboxylic acid ethyl ester (S10)**

[14] Fischer, H.; Summers, L. A. *J. Heterocyclic Chem.* **1980**, 333-336.
[15] De, D.; Krogstad, F. M.; Byers, L. D.; Krogstad, D. J. *J. Med.l Chem.*, **1998**, *41*, 4918 – 4926.

S9 (2.95 g, 10 mmol) and potassium carbonate (4.14 g, 30 mmol) were suspended in DMF (30 mL) and ethyl iodide (4 mL, 50 mmol) was added. The solution was heated at 80 °C for 5 hours, then poured into water (100 mL). The resulting suspension was filtered and solid was dissolved in CH_2Cl_2 (50 mL), dried ($MgSO_4$), filtered, and concentrated *in vacuo*. The crude material was purified by FCC (50-100% EtOAc in pentane) to give *heterocycle* **S10** (2.37 g, 73% yield) as a brown solid. Spectroscopic data matched those previous reported.[16]

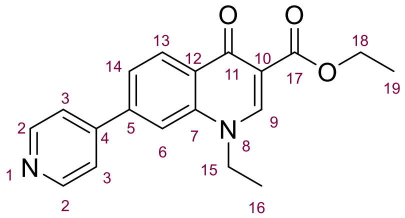

N-Ethyl-7-(4-pyridine)-4-oxoquinoline-3-carboxylic acid ethyl ester (1x)

4-Pyridylboronic acid (553 mg, 4.5 mmol), **S10** (969 mg, 3 mmol), potassium carbonate (1.04 g, 7.5 mmol), and triphenylphosphine (79 mg, 10 mol%) were dissolved in dioxane:water (25 mL:10 mL). The solution was purged with argon for 10 minutes, then palladium acetate (17 mg, 2.5 mol%) was added. The reaction was heated at 80 °C under argon for 14 hours, then cooled, and diluted with EtOAc (50 mL) and water (50 mL). The solution was separated, and the aqueous layer was extracted with EtOAc (2 x 50 mL, the organic layers were combined, washed with brine, dried ($MgSO_4$), filtered, and concentrated *in vacuo*. The crude material was purified by FCC (0-4% MeOH in CH_2Cl_2) to yield *heterocycle* **1x** (572 mg, 59% yield) as a cream solid.

m.p. (acetone): 165-167°C;

HRMS (ESI): Exact mass calculated for $C_{19}H_{19}O_3N_2$ $[M+H]^+$ calc: 323.13902. found: 323.13908;

^{1}H NMR ($CDCl_3$) δ 8.76 (2H, d, J = 6.2 Hz, 2 x C^2**H**), 8.65 (1H, d, J = 8.3 Hz, C^{13}**H**), 8.56 (1H, s, C^9**H**), 7.67 (1H, dd, J = 8.3, 1.5 Hz, C^{14}**H**), 7.64 (1H, d, J = 1.5 Hz, C^6**H**), 7.57 (2H, d,

[16] US Patent ASTRAZENECA AB - US2010/317624, **2010**, A1

J = 6.1 Hz, 2 x C^3**H**), 4.42 (2H, q, *J* = 7.1 Hz, $C^{18}$$\mathbf{H}_2$), 4.35 (2H, q, *J* = 7.3 Hz, $C^{15}$$\mathbf{H}_2$), 1.61 (3H, t, *J* = 7.2 Hz, $C^{16}$$\mathbf{H}_3$), 1.43 (3H, t, *J* = 7.1 Hz, $C^{19}$$\mathbf{H}_3$);

^{13}C NMR ($CDCl_3$) δ 173.8 (C^{17}), 165.7 (C^{11}), 150.4 (2 x C^2), 149.0 (C^{ArH}), 147.3 (C), 142.6 (C), 139.1 (C), 129.3 (C^{ArH}), 123.9 (C^{ArH}), 122.0 (2 x C^3), 121.4 (C), 114.1 (C^{ArH}), 111.7 (C)¸61.0 (C^{18}), 48.9 (C^{15}), 14.6 (C^{16}), 14.4 (C^{19});

IR (neat): 3500, 2981, 2160, 2029, 1672, 1628, 1595, 1558, 1533, 1464, 1385, 1323 cm^{-1}.

Preparation of the Pyridinium Salts

General Procedure D: Preparation of 4-((triisopropylsilyl)oxy)benzyl) pyridinium bromide salts

A mixture of the corresponding pyridine (1.0 equiv.) and (4-(bromomethyl)phenoxy)triisopropylsilane (1.5 equiv.) in dioxane (0.3 M) was stirred at 90 °C for 16 hours. The mixture was allowed to cool to room temperature, then the solvent was removed under reduced pressure, followed by addition of diethyl ether (15 mL/mmol of substrate). The resulting suspension was sonicated (5 min) then filtered. The resultant solid was washed with diethyl ether and dried under vacuum for one hour to give the pyridinium bromide salt.

General Procedure E: Preparation of 4-((triisopropylsilyl)oxy)benzyl) pyridinium bromide salts

A mixture of the corresponding pyridine (1.0 equiv.) and (4-(bromomethyl)phenoxy)triisopropylsilane (1.5 equiv.) in acetone (0.3 M) was stirred at 65 °C for 16 hours. The mixture was allowed to cool to room temperature, then the solvent was removed under reduced pressure, followed by addition diethyl ether (15 mL/mmol of substrate). The resulting suspension was sonicated (5 min) then filtered. The resultant solid was washed with diethyl ether and dried under vacuum for one hour to give the pyridinium bromide salt.

General Procedure F: Preparation of 4-((triisopropylsilyl)oxy)benzyl) pyridinium iodide salts

A mixture of the corresponding pyridine (1.0 equiv.) and (4-(iodomethyl)phenoxy)triisopropylsilane (1.5 equiv.) in acetone (0.3 M) was stirred at room temperature for 16 hours in the dark. The solvent was removed under reduced pressure,

followed by addition of diethyl ether (15 mL/mmol of substrate). The resulting suspension was sonicated (5 min) then filtered. The resultant solid was washed with diethyl ether and dried under vacuum for one hour to give the pyridinium iodide salt.

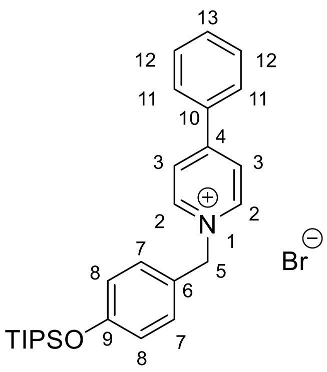

4-Phenyl-*N*-(4-((triisopropylsilyl)oxy)benzyl)pyridinium Bromide (2a)

The title compound was prepared according to **General Procedure D** using 4-phenylpyridine (310.4 mg, 2.0 mmol) to give *salt* **2a** (947.5 mg, 95% yield) as a white solid.

m.p.: 233-235 °C;

HRMS (ESI): Exact mass calculated for $C_{27}H_{36}ON^{28}Si$ $[M]^+$ m/z: 418.25607, found: 418.25641;

^{1}H NMR (400 MHz, DMSO-d_6) δ 9.25 (2H, d, *J* = 6.8 Hz, 2 x C^2**H**), 8.55 (2H, d, *J* = 6.9 Hz, 2 x C^3**H**), 8.07 (2H, dd, *J* = 7.7, 2.0 Hz, 2 x C^{Ar}**H**), 7.69 – 7.60 (3H, m, 3 x C^{Ar}**H**), 7.53 (2H, d, *J* = 8.6 Hz, 2 x C^7**H**), 6.92 (2H, d, *J* = 8.6 Hz, 2 x C^8**H**), 5.80 (2H, s, C^5**H**$_2$), 1.30 – 1.17 (3H, m, 3 x TIPS-C**H**), 1.03 (18H, d, *J* = 7.4 Hz, 6 x TIPS-C**H**$_3$);

^{13}C NMR (101 MHz, DMSO-d_6) δ 156.3 (C), 155.0 (C), 144.6 (C^2), 133.5 (C), 132.2 (C^{Ar}), 130.7 (C^7), 129.7 (2 x C, C^{Ar}), 128.2 (2 x C^{Ar}), 127.1 (C), 124.9 (C^3), 120.1 (C^8), 61.9 (C^5), 17.7 (6 x TIPS-CH_3), 12.0 (3 x TIPS-CH);

IR (neat) (cm^{-1}): 1635, 1608, 1511, 1490, 1281, 1157, 993, 914, 885, 865, 812, 773, 749, 705, 685, 660.

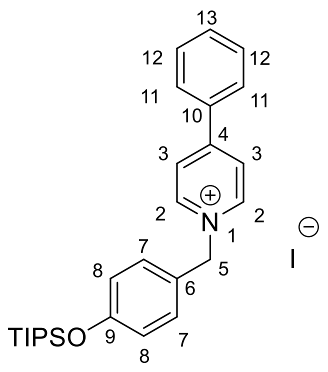

4-Phenyl-*N*-(4-((triisopropylsilyl)oxy)benzyl)pyridinium Iodide (2a')

The title compound was prepared according to **General Procedure F** using 4-phenylpyridine (310.4 mg, 2.0 mmol) to give *salt* **2a'** (1058 mg, 97% yield) as a light-yellow solid.

m.p.: 216-218 °C;

HRMS (ESI): Exact mass calculated for $C_{27}H_{36}ON^{28}Si$ $[M]^+$ m/z: 418.25607, found: 418.25607;

^{1}H NMR (400 MHz, DMSO-d_6) δ 9.24 (2H d, J = 7.1 Hz, 2 x C^2**H**), 8.55 (2H, d, J = 7.1 Hz, 2 x C^3**H**), 8.07 (2H, dd, J = 7.6, 2.0 Hz, 2 x C^{Ar}**H**), 7.68 – 7.61 (3H, m, 3 x C^{Ar}**H**), 7.52 (2H, d, J = 8.6 Hz, 2 x C^7**H**), 6.92 (2H, d, J = 8.6 Hz, 2 x C^8**H**), 5.79 (2H, s, C^5**H**$_2$), 1.30 – 1.17 (3H, m, 3 x TIPS-C**H**), 1.03 (18H, d, J = 7.5 Hz, 6 x TIPS-C**H**$_3$);

^{13}C NMR (101 MHz, DMSO-d_6) δ 156.3 (C), 155.0 (C), 144.6 (2 x C^2), 133.5 (C), 132.2 (C^{Ar}), 130.7 (2 x C^7), 129.7 (2 x C, C^{Ar}), 128.2 (2 x C, C^{Ar}), 127.1 (C), 124.9 (2 x C^3), 120.1 (C^8), 61.9 (C^5), 17.7 (6 x TIPS-CH$_3$), 12.0 (3 x TIPS-CH);

IR (neat) (cm^{-1}): 2943, 1635, 1607, 1511, 1489, 1461, 1285, 1159, 990, 915, 884, 811, 775, 749, 705, 688, 673.

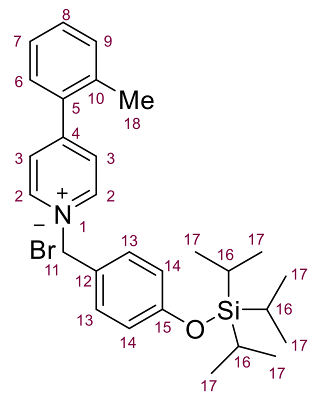

4-(3-Methylphenyl)-1-(4-((triisopropylsilyl)oxy)benzyl)pyridine-1-ium Bromide (2b)

The title compound was prepared using **General Procedure E** with pyridine **1b** (338 mg, 2 mmol) to give *salt* **2b** (1.00 g, 98% yield) as a white solid.

m.p. (acetone): 198-200 °C;

HRMS (ESI): Exact mass calculated for $C_{28}H_{38}ON^{28}Si$ $[M]^+$ calc: 432.27172, found: 432.27130;

^{1}H NMR ((CD_3)$_2$SO) δ 9.21 (2H, d, *J* = 6.9 Hz, 2 x C^2**H**), 8.24 (2H d, *J* = 6.9 Hz, 2 x C^3**H**), 7.53 (2H, d, *J* = 8.6 Hz, 2 x C^{13}**H**), 7.50-7.36 (4H, m, C^6**H** + C^7**H** + C^8**H** + C^9**H**), 6.95 (2H, d, *J* = 8.6 Hz, 2 x C^{14}**H**), 5.80 (2H, s, C^{11}**H**$_2$), 2.34 (3H, s, C^{18}**H**$_3$), 1.31-1.17 (3H, m, 3 x C^{16}**H**), 1.05 (18H, d, *J* = 7.4 Hz, 6 x C^{17}**H**$_3$);

^{13}C NMR ((CD_3)$_2$SO) δ 157.5 (C), 156.4 (C), 144.1 (2 x C^2), 135.6 (C), 135.5 (C), 131.3 (CH), 130.8 (2 x C^{13}), 130.5 (CH), 129.8 (CH), 128.4 (2 x C^3), 126.9 (C), 126.7 (CH), 120.2 (2 x C^{14}), 62.2 (C^{11}), 19.9 (C^{18}), 17.8 (6 x C^{17}), 12.1 (3 x C^{16});

IR (neat): 3505, 2943, 2865, 1636, 1608, 1510, 1461, 1276, 1209, 1178, 1157, 992 cm^{-1}.

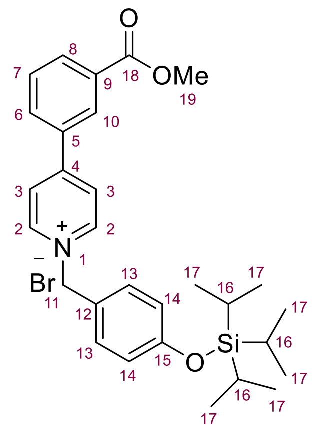

4-(3-Methoxycarbonyl)phenyl)-1-(4-((triisopropylsilyl)oxy)benzyl)pyridine-1-ium Bromide (2c)

The title compound was prepared using **General Procedure E** with pyridine **1c** (426 mg, 2 mmol) to give *salt* **2c** (1.07 g, 96% yield) as a white solid.

m.p. (acetone): 210-212 °C;

HRMS (ESI): Exact mass calculated for $C_{29}H_{38}ON^{28}Si$ $[M]^+$ calc: 476.26155, found: 476.26120;

^{1}H NMR ((CD_3)$_2$SO) δ 9.27 (2H, d, *J* = 6.6 Hz, 2 x C^2**H**), 8.61 (2H, d, *J* = 7.0 Hz, 2 x C^3**H**), 8.51 (1H, s, C^{10}**H**), 8.32 (1H, dd, *J* = 8.1, 1.7 Hz, C^8**H**), 8.20 (1H, dd, *J* = 7.8, 1.5 Hz, C^6**H**), 7.80 (1H, t, *J* = 7.8 Hz, C^7**H**), 7.51 (2H, d, *J* = 8.5 Hz, 2 x C^{13}**H**), 6.93 (2H, d, *J* = 8.5 Hz, 2 x C^{14}**H**), 5.81 (2H, s, C^{11}**H**$_2$), 3.92 (3H, s, C^{19}**H**$_3$), 1.24 (3H, qd, *J* = 6.8, 2.1 Hz, 3 x C^{16}**H**), 1.05 (9H, s, 3 x C^{17}**H**$_3$), 1.03 (9H, s, 3 x C^{17}**H**$_3$);

^{13}**C NMR** ((CD$_3$)$_2$SO) δ 165.6 (C^{18}), 156.3 (C), 154.0 (C), 144.8 (2 x C^{2}), 134.4 (C), 132.9 (C^{8}), 132.2 (C^{6}), 131.0 (C), 130.7 (2 x C^{13}), 130.3 (C^{7}), 128.7 (C^{10}), 127.1 (C), 125.5 (2 x C^{3}), 120.1 (2 x C^{14}), 62.0 (C^{11}), 52.6 (C^{18}), 17.7 (6 x C^{17}), 12.0 (3 x C^{16});

IR (neat): 2980, 2161, 1724, 1636, 1607, 1510, 1462, 1384, 1310, 1255, 1160, 1106.

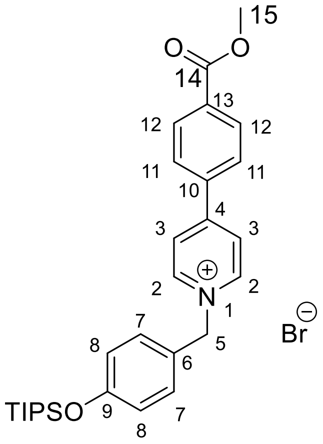

4-(4-(Methoxycarbonyl)phenyl)-*N*-(4-((triisopropylsilyl)oxy)benzyl)pyridinium Bromide (2d)

The title compound was prepared according to **General Procedure D** using methyl 4-(pyridin-4-yl)benzoate (426.5 mg, 2.0 mmol) to give *salt* **2d** (1093 mg, 98% yield) as a white solid.

m.p.: 180-182 °C;

HRMS (ESI): Exact mass calculated for $C_{29}H_{38}O_3N^{28}Si$ [M]$^+$ m/z: 476.26155, found: 476.26208;

^{1}**H NMR** (400 MHz, DMSO-*d*$_6$) δ 9.32 (2H, d, *J* = 7.1 Hz, 2 x C^{2}**H**), 8.61 (2H, d, *J* = 7.1 Hz, 2 x C^{3}**H**), 8.24 – 8.14 (4H, m, 4 x C^{Ar}**H**), 7.54 (2H, d, *J* = 8.6 Hz, 2 x C^{7}**H**), 6.93 (2H, d, *J* = 8.6 Hz, 2 x C^{8}**H**), 5.83 (2H, s, 2 x C^{5}**H**$_2$), 3.91 (3H, s, C^{15}**H**$_3$), 1.31 – 1.18 (3H, m, 3 x TIPS-C**H**), 1.04 (18H, d, *J* = 7.4 Hz, 6 x TIPS-C**H**$_3$);

^{13}**C NMR** (101 MHz, DMSO-*d*$_6$) δ 165.5 (C^{14}), 156.3 (C), 153.7 (C), 144.8 (2 x C^{2}), 137.8 (C), 132.2 (C), 130.7 (2 x C^{7}H), 130.1 (2 x C^{Ar}), 128.7 (2 x C^{Ar}), 127.0 (C), 125.6 (2 x C^{3}), 120.1 (2 x C^{8}), 62.1 (C^{5}), 52.6 (C^{15}), 17.7 (6 x TIPS-CH$_3$), 12.0 (3 x TIPS-CH);

IR (neat) (cm^{-1}): 1727, 1633, 1514, 1458, 1433, 1276, 1179, 1108, 1014, 919, 882, 841, 801, 773, 753, 716, 673.

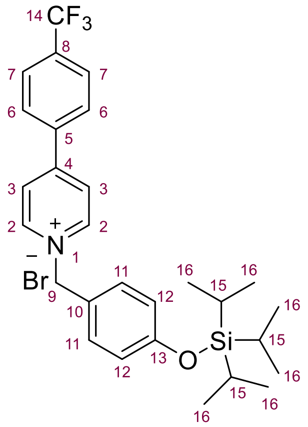

4-(4-(Trifluoromethyl)phenyl)-1-(4-((triisopropylsilyl)oxy)benzyl)pyridin-1-ium Bromide (2e)

The title compound was prepared using **General Procedure E** with pyridine **1e** (446 mg, 2 mmol) to give *salt* **2e** (1.12 g, 99% yield) as a white solid.

m.p. (acetone): 205-207 °C;

HRMS (ESI): Exact mass calculated for $C_{28}H_{35}ONF_3{}^{28}Si$ $[M]^+$ calc: 486.24345, found: 486.24313;

^{1}H NMR (($(CD_3)_2SO$) δ 9.31 (2H, d, *J* = 7.4 Hz, 2 x C^2**H**), 8.61 (2H, d, *J* = 7.1 Hz, 2 x C^3**H**), 8.24 (2H, d, *J* = 8.6 Hz, 2 x C^6**H**), 8.14 (2H, d, *J* = 8.6 Hz, 2 x C^7**H**), 7.52 (2H, d, *J* = 8.6 Hz, 2 x C^{11}**H**), 6.93 (2H, d, *J* = 8.5 Hz, 2 x C^{12}**H**), 5.80 (2H, s, C^9**H**$_2$), 1.25 (3H, ddd, *J* = 14.8, 8.2, 6.9 Hz, 3 x C^{15}**H**), 1.04 (18H, d, *J* = 7.4 Hz, 6 x C^{16}**H**$_3$);

^{13}C NMR ($(CD_3)_2SO$) δ 156.4 (C), 153.5 (C), 144.9 (2 x C^2), 137.7 (C), 131.5 (q, *J* = 32.4 Hz, C^8), 130.8 (2 x C^{11}), 129.3 (2 x C^6), 127.0 (C), 126.4 (q, *J* = 3.6 Hz, 2 x C^7), 125.8 (2 x C^3), 123.8 (q, *J* = 272.5 Hz, C^{14}), 120.1 (2 x C^{12}), 62.2 (C^9), 17.7 (6 x C^{16}), 12.0 (3 x C^{15});

^{19}F NMR ($(CD_3)_2SO$) δ –62.7;

IR (neat): 2981, 2161, 1637, 1607, 1513, 1462, 1408, 1329, 1274, 1207, 1159, 1127 cm^{-1}.

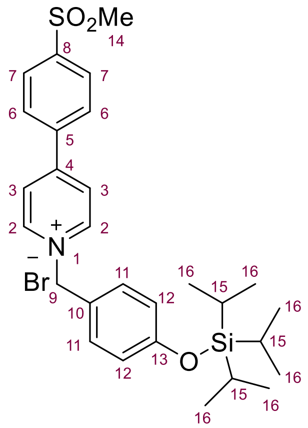

4-(4-Methylsulfonyl)phenyl)-1-(4-((triisopropylsilyl)oxy)benzyl)pyridine-1-ium Bromide (2f)

The title compound was prepared using **General Procedure E** with pyridine **1f** (466 mg, 2 mmol) to give *salt* **2f** (1.06 g, 92% yield) as a white solid.

m.p. (acetone): 198-200 °C;

HRMS (ESI): Exact mass calculated for $C_{28}H_{38}ON^{32}S^{28}Si$ $[M]^+$ calc: 496.23362, found: 496.23343;

^{1}H NMR ($(CD_3)_2SO$) δ 9.37 (2H, d, *J* = 7.0 Hz, 2 x C^2**H**), 8.63 (2H, d, *J* = 7.0 Hz, 2 x C^3**H**), 8.31 (2H, d, *J* = 8.6 Hz, 2 x C^7**H**), 8.16 (2H, d, *J* = 8.6 Hz, 2 x C^6**H**), 7.56 (2H, d, *J* = 8.6 Hz, 2 x C^{11}**H**), 6.93 (2H, d, *J* = 8.6 Hz, 2 x C^{12}**H**), 5.85 (2H, s, C^9**H**$_2$), 3.34 (3H, s, C^{14}**H**$_3$), 1.24 (3H, ddd, *J* = 14.8, 8.2, 6.9 Hz, 3 x C^{15}**H**), 1.04 (9H, s, 3 x C^{16}**H**$_3$), 1.02 (9H, s, 3 x C^{16}**H**$_3$);

^{13}C NMR ($(CD_3)_2SO$) δ 156..4 (C), 153.4 (C), 144.9 (2 x C^2), 143.3 (C), 138.4 (C), 130.8 (2 x C^{11}), 129.4 (2 x C^7), 128.0 (2 x C^6), 127.0 (C), 125.9 (2 x C^3), 120.1 (2 x C^{12}), 62.3 (C^9), 43.2 (C^{14}), 17.7 (6 x C^{16}), 12.0 (3 x C^{15});

IR (neat): 3425, 2941, 2865, 2161, 2029, 1637, 1606, 1511, 1469, 1306, 1273, 1206 cm^{-1}.

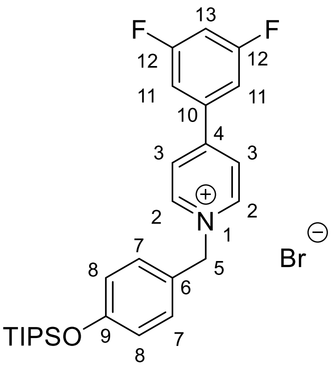

4-(3,5-Difluorophenyl)-*N*-(4-((triisopropylsilyl)oxy)benzyl)pyridinium Bromide (2g)

The title compound was prepared according to **General Procedure D** using 4-(3,5-difluorophenyl)pyridine (382.4 mg, 2.0 mmol) to give *salt* **2g** (1030 mg, 96% yield) as a white solid.

m.p.: 220-222 °C;

HRMS (ESI): Exact mass calculated for $C_{27}H_{34}OF_2N^{28}Si$ $[M]^+$ m/z: 454.23722, found: 454.23724;

^{1}H NMR (400 MHz, DMSO-d_6) δ 9.34 (2H, d, *J* = 7.0 Hz, 2 x C^2**H**), 8.62 (2H, d, *J* = 7.1 Hz, 2 x C^3**H**), 7.92 (2H, dt, *J* = 7.0, 2.1 Hz, 2 x C^{11}**H**), 7.59 (1H, tt, *J* = 9.0, 2.1 Hz, C^{13}**H**), 7.53 (2H, d, *J* = 8.6 Hz, 2 x C^7**H**), 6.92 (2H, d, *J* = 8.6 Hz, 2 x C^8**H**), 5.81 (2H, s, C^5**H**$_2$), 1.28 – 1.18 (3H, m, 3 x TIPS-C**H**), 1.04 (18H, d, *J* = 7.4 Hz, 6 x TIPS-C**H**$_3$);

^{13}C NMR (101 MHz, DMSO-d_6) δ 163.4 (dd, *J* = 247.2, 13.4 Hz, 2 x C^{12}), 156.8 (C), 152.9 (C), 145.4 (2 x C^2), 137.40 (t, *J* = 10.2 Hz, C^{10}), 131.2 (2 x C^7), 127.4 (C), 126.0 (2 x C^3), 120.6

(2 x C^{8}), 112.3 (m, 2 x C^{11}), 107.8 (t, *J* = 26.2 Hz, C^{13}), 62.7 (C^{5}), 18.2 (6 x TIPS-CH_3), 12.5 (3 x TIPS-CH);

^{19}F NMR (377 MHz, DMSO-d_6) δ –107.9;

IR (neat) (cm^{-1}): 2942, 1638, 1606, 1510, 1473, 1426, 1337, 1280, 1176, 1154, 1125, 991, 913, 869, 800, 740, 675, 658.

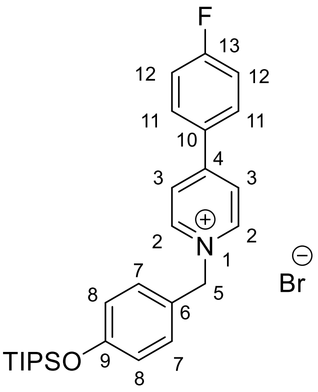

4-(4-Fluorophenyl)-*N*-(4-((triisopropylsilyl)oxy)benzyl)pyridinium Bromide (2h)

The title compound was prepared according to **General Procedure D** using 4-(4-fluorophenyl)pyridine (346.4 mg, 2.0 mmol) to give *salt* **2h** (981.5 mg, 95% yield) as a white solid.

m.p.: 223-225 °C;

HRMS (ESI): Exact mass calculated for $C_{27}H_{35}OFN^{28}Si$ $[M]^+$ m/z: 436.24665, found: 436.24670;

^{1}H NMR (400 MHz, DMSO-d_6) δ 9.25 (2H, d, *J* = 7.1 Hz, 2 x C^{2}**H**), 8.55 (2H, d, *J* = 7.1 Hz, 2 x C^{3}**H**), 8.21 – 8.15 (2H, m, 2 x C^{11}**H**), 7.55 – 7.48 (4H, m, 2 x C^{12}**H** + 2 x C^{7}**H**), 6.93 (2H, d, *J* = 8.6 Hz, 2 x C^{8}**H**), 5.79 (2H, s, C^{5}**H_2**), 1.30 – 1.18 (3H, m, 3 x TIPS-C**H**), 1.04 (18H, d, *J* = 7.5 Hz, 6 x TIPS-C**H_3**);

^{13}C NMR (101 MHz, DMSO-d_6) δ 165.0 (d, *J* = 251.4 Hz, C^{13}), 156.8 (C), 154.3 (C), 145.0 (2 x C^{2}), 131.5 (d, *J* = 9.3 Hz, 2 x C^{11}), 131.1 (2 x C^{7}), 130.5 (d, *J* = 2.9 Hz, C^{10}), 127.6 (C), 125.2 (2 x C^{3}), 120.6 (2 x C^{8}), 117.24 (d, *J* = 21.9 Hz, 2 x C^{12}), 62.4 (C^{5}), 18.2 (6 x TIPS-CH_3), 12.5(3 x TIPS-CH);

^{19}F NMR (377 MHz, DMSO-d_6) δ –107.8;

IR (neat) (cm^{-1}): 1638, 1594, 1495, 1271, 1229, 1149, 990, 907, 838, 746, 675, 647, 633.

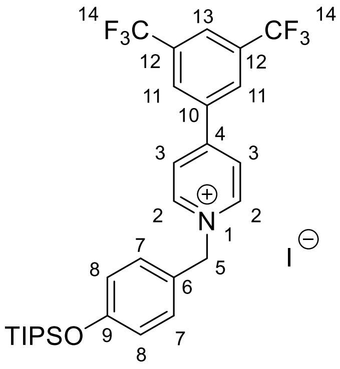

4-(3,5-Bis(trifluoromethyl)phenyl)-*N*-(4-((triisopropylsilyl)oxy)benzyl)pyridinium Iodide (2i'-)

The title compound was prepared according to **General Procedure F** using 4-(3,5-bis(trifluoromethyl)phenyl)pyridine (873.6 mg, 3.0 mmol) to give *salt* **2i'** (1871 mg, 92% yield) as a yellow solid.

m.p.: 191-193 °C;

HRMS (ESI): Exact mass calculated for $C_{29}H_{34}OF_6N^{28}Si$ $[M]^+$ m/z: 554.23084, found: 554.23041;

^{1}H NMR (400 MHz, DMSO-*d*$_6$) δ 9.39 (2H, d, *J* = 6.4 Hz, 2 x C^2**H**), 8.79 (2H, d, *J* = 6.5 Hz, 2 x C^3**H**), 8.74 (2H, s, 2 x C^{11}**H**), 8.40 (1H, s, C^{13}**H**), 7.55 (2H, d, *J* = 8.1 Hz, 2 x C^7**H**), 6.93 (2H, d, *J* = 8.2 Hz, 2 x C^8**H**), 1.31 – 1.18 (3H, m, 3 x TIPS-C**H**), 1.04 (18H, d, *J* = 7.5 Hz, 6 x TIPS-C**H**$_3$);

^{13}C NMR (101 MHz, DMSO-*d*$_6$) δ 156.4 (C), 152.1 (C), 144.8 (2 x C^2), 136.5 (C), 131.3 (q, *J* = 33.5 Hz, 2 x C^{12}), 130.6 (2 x C^7), 129.4 (2 x C^{11}), 127.0 (C), 126.4 (2 x C^3), 125.0 (C^{13}), 123.0 (q, *J* = 273.3 Hz, 2 x C^{14}), 120.1 (2 x C^8), 62.4 (C^5), 17.7 (6 x TIPS-CH$_3$), 12.0 (3 x TIPS-CH);

^{19}F NMR (377 MHz, DMSO-*d*$_6$) δ –61.2;

IR (neat) (cm^{-1}): 2946, 2869, 1508, 1382, 1275, 1174, 1141, 1110, 1061, 904, 882, 841, 682.

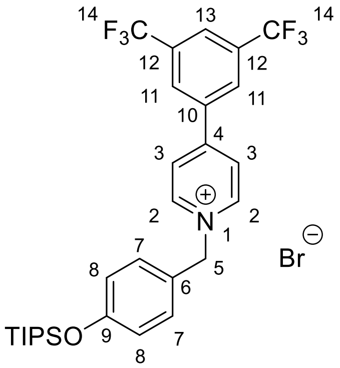

4-(3,5-Bis(trifluoromethyl)phenyl)-*N*-(4-((triisopropylsilyl)oxy)benzyl)pyridinium Bromide (2i)

The title compound was prepared according to **General Procedure E** using 4-(3,5-bis(trifluoromethyl)phenyl)pyridine

(582.4 mg, 2.0 mmol) to give *salt* **2i** (1850 mg, 97% yield) as a white solid.

m.p.: 252-254 °C;

HRMS (ESI): Exact mass calculated for $C_{29}H_{34}OF_6N^{28}Si$ $[M]^+$ m/z: 554.23084, found: 554.23114;

^{1}H NMR (400 MHz, DMSO-d_6) δ 9.42 (2H, d, *J* = 7.0 Hz, 2 x C^2**H**), 8.79 (2H, d, *J* = 7.1 Hz, 2 x C^3**H**), 8.73 (2H, s, 2 x C^{11}**H**), 8.41 (1H, s, C^{13}**H**), 7.56 (2H, d, *J* = 8.6 Hz, 2 x C^7**H**), 6.93 (2H, d, *J* = 8.6 Hz, 2 x C^8**H**), 5.87 (2H, s, C^5**H**$_2$), 1.30 – 1.19 (3H, m, 3 x TIPS-C**H**), 1.04 (18H, d, *J* = 7.4 Hz, 6 x TIPS-C**H**$_3$);

^{13}C NMR (101 MHz, DMSO-d_6) δ 156.3 (C), 152.1 (C), 144.8 (2 xC^2), 136.5 (C), 131.3 (q, *J* = 33.5 Hz, 2 x C^{12}), 130.6 (2 x C^7), 129.4 (2 x C^{11}), 127.0 (C), 126.4 (2 x C^3), 125.0 (C^{13}), 123.0 (d, *J* = 273.1 Hz, 2 x C^{14}), 120.0 (2 x C^8), 62.3 (C^5), 17.7 (6 x TIPS-CH$_3$), 12.0 (3 x TIPS-CH);

^{19}F NMR (377 MHz, DMSO-d_6) δ –61.1;

IR (neat) (cm^{-1}): 1509, 1383, 1276, 1173, 1143, 1110, 914, 904, 882, 837, 701, 682.

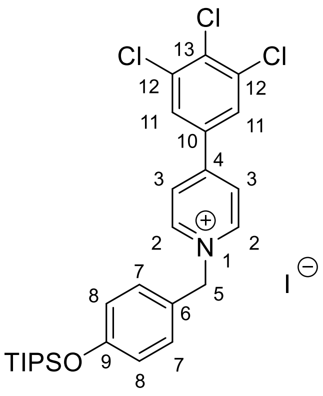

4-(3,4,5-Trichlorophenyl)-*N*-(4-((triisopropylsilyl)oxy)benzyl)pyridinium Iodide (2j')

The title compound was prepared according to **General Procedure F** using 4-(3,4,5-trichlorophenyl)pyridine (387.8 mg, 1.5 mmol) to give *salt* **2j'** (973 mg, 97% yield) as a yellow solid.

m.p.: 222-224 °C;

HRMS (ESI): Exact mass calculated for $C_{27}H_{33}ON^{35}Cl_3{}^{28}Si$ $[M]^+$ m/z: 520.13915, found: 520.13971;

^{1}H NMR (400 MHz, DMSO-d_6) δ 9.31 (2H, d, *J* = 7.1 Hz, 2 x C^2**H**), 8.64 (2H, d, *J* = 7.1 Hz, 2 x C^3**H**), 8.40 (2H, s, 2 x C^{11}**H**), 7.51 (2H, d, *J* = 8.6 Hz, 2 x C^7**H**), 6.92 (2H, d, *J* = 8.6 Hz, 2

x C^8**H**), 5.78 (2H, s, C^5**H**$_2$), 1.30 – 1.17 (3H, m, 3 x TIPS-C**H**), 1.03 (18H, d, *J* = 7.5 Hz, 6 x , TIPS-C**H**$_3$);

^{13}C NMR (101 MHz, DMSO-*d*$_6$) δ 156.4 (C), 151.5 (C), 144.8 (2 x C^2), 134.4 (C), 134.2 (2 x C^{12}), 133.4 (C), 130.7 (2 x C^7), 128.8 (2 x C^{11}), 126.9 (C), 125.7 (2 x C^3), 120.1 (2 x C^8), 62.4 (C^5), 17.7 (6 x TIPS-CH$_3$), 12.0 (3 x TIPS-CH);

IR (neat) (cm^{-1}): 2945, 1643, 1541, 1511, 1461, 1431, 1278, 1172, 993, 915, 733, 679.

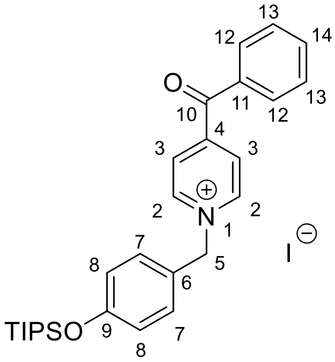

4-Benzoyl-*N*-(4-((triisopropylsilyl)oxy)benzyl)pyridinium Iodide (2k')

The title compound was prepared according to **General Procedure F** using phenyl(pyridin-4-yl)methanone (916.1 mg, 5.0 mmol) to give *salt* **2k'** (2.81 g , 98% yield) as a yellow solid.

m.p.: 125-127 °C;

HRMS (ESI): Exact mass calculated for $C_{28}H_{36}O_2N^{28}Si$ $[M]^+$ m/z: 446.25098, found: 446.25128;

^{1}H NMR (400 MHz, DMSO-*d*$_6$) δ 9.36 (2H, d, *J* = 6.8 Hz, 2 x C^2**H**), 8.38 (2H, d, *J* = 6.7 Hz, 2 x C^3**H**), 7.88 – 7.84 (2H, m, 2 x C^{12}**H**), 7.83 – 7.78 (1H, m, C^{14}**H**), 7.67 – 7.59 (2H, m, 2 x C^{13}**H**), 7.54 (2H, d, *J* = 8.6 Hz, 2 x C^7**H**), 6.95 (2H, d, *J* = 8.5 Hz, 2 x C^8**H**), 5.88 (2H, s, C^5**H**$_2$), 1.34 – 1.20 (3H, m, 3 x TIPS-C**H**), 1.05 (18H, d, *J* = 7.5 Hz, 6 x TIPS-C**H**$_3$);

^{13}C NMR (101 MHz, DMSO-*d*$_6$) δ 192.1 (C^{10}), 156.5 (C), 151.7 (C), 145.6 (2 x C^2), 134.9 (C^{14}), 134.1 (C), 131.0 (2 x C^7H), 130.4 (2 x C^{12}), 129.1 (2 x C^{13}), 127.4 (2 x C^3), 126.4 (C), 120.1 (2 x C^8), 63.1 (C^5), 17.8 (6 x TIPS-CH$_3$), 12.1 (3 x TIPS-CH);

IR (neat) (cm^{-1}): 1669, 1513, 1458, 1288, 1268, 907, 886, 844, 807, 737, 686, 645, 634.

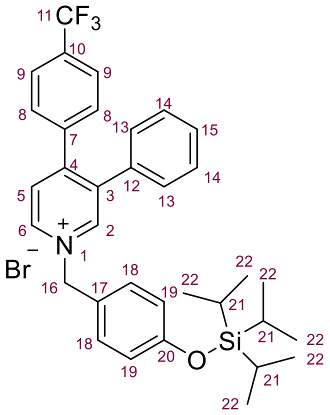

3-Phenyl-4-(4-trifluoromethyl)phenyl)-1-(4-((triisopropylsilyl)oxy)benzyl)pyridine-1-ium Bromide (2l)

The title compound was prepared using **General Procedure E** with pyridine **1l** (598 mg, 2.0 mmol) to give, after FCC (0-10% MeOH in CH_2Cl_2), *salt* **2l** (1.24 g, 96% yield) as a yellow solid.

mp (acetone): 120-122 °C;

HRMS (ESI): Exact mass calculated for $C_{34}H_{39}ONF_3{}^{28}Si$ $[M]^+$ calc: 562.27475, found: 562.27441;

^{1}H NMR ($(CD_3)_2SO$) δ 9.62 (1H, dd, *J* = 6.4, 1.5 Hz, C^6**H**), 9.00 (1H, d, *J* = 1.4 Hz, C^2**H**), 7.87 (1H, d, *J* = 6.3 Hz, C^5**H**), 7.54 (2H, d, *J* = 8.6 Hz, 2 x C^8**H**), 7.47 (2H, 8.2 Hz, 2 x C^9**H**), 7.32-7.21 (3H, m, 2 x C^{13}**H** + C^{15}**H**), 7.21-7.15 (4H, m, 2 x C^{14}**H** + 2 x C^{18}**H**), 6.81 (2H, d, *J* = 8.6 Hz, 2 x C^{19}**H**), 6.25 (2H, s, C^{16}**H**$_2$), 1.24-1.08 (3H, m, 3 x C^{21}**H**), 0.99 (18H, d, *J* = 7.3 Hz, 6 x C^{22}**H**$_3$);

^{13}C NMR ($(CD_3)_2SO$) δ 157.7 (C), 154.6 (C), 144.4 (C^2), 143.3 (C^6), 140.1 (C), 138.4 (C), 132.7 (C), 132.0 (q, *J* = 32.8 Hz, C^{10}), 131.5 (2 x C^8), 129.72 (2 x C^{14}), 129.67 (C^{15}), 129.6 (2 x C^{18}), 129.2 (2 x C^{13}), 128.8 (C^5), 125.8 (q, *J* = 3.5 Hz, 2 x C^9), 124.8 (C), 123.4 (q, *J* = 272.5 Hz, C^{11}), 121.0 (2 x C^{19}), 63.8 (C^{16}), 17.8 (6 x C^{22}), 12.5 (3 x C^{21});

^{19}F NMR ($(CD_3)_2SO$) δ –63.0;

IR (neat): 2980, 2161, 1607, 1511, 1464, 1406, 1323, 1269, 1168, 1127, 1070, 1014 cm^{-1}.

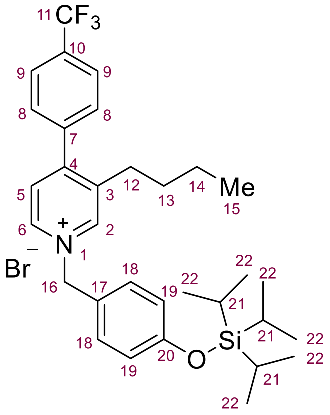

3-(*n*-Butyl)-4-(4-trifluoromethyl)phenyl)-1-(4-((triisopropylsilyl)oxy)benzyl)pyridine-1-ium Bromide (2m)

The title compound was prepared using **General Procedure E** with pyridine **1m** (558 mg, 2.0 mmol) to give, after FCC (0-5% MeOH in CH_2Cl_2), *salt* **2m** (0.91 g, 73% yield) as a white solid.

m.p. (acetone): 139-141 °C;

HRMS (ESI): Exact mass calculated for $C_{32}H_{43}ONF_3{}^{28}Si$ $[M]^+$ calc: 542.30605, found: 542.30597;

^{1}H NMR ($(CD_3)_2SO$) δ 9.34 (1H, s, C^2**H**), 9.16 (1H, dd, *J* = 6.3, 1.4 Hz, C^6**H**), 8.08 (1H, d, *J* = 6.3 Hz, C^5**H**), 7.96 (2H, d, *J* = 7.9 Hz, 2 x C^9**H**), 7.76 (2H, d, *J* = 7.9 Hz, 2 x C^8**H**), 7.56 (2H, d, *J* = 8.6 Hz, 2 x C^{18}**H**), 6.94 (2H, d, *J* = 8.6 Hz, 2 x C^{19}**H**), 5.83 (2H, s, $C^{16}H_2$), 2.74 (2H, dd, *J* = 8.9, 6.8 Hz, $C^{12}H_2$), 1.47-1.39 (2H, m, $C^{13}H_2$), 1.25 (3H, dq, *J* = 8.6, 7.5 Hz, 3 x C^{21}**H**), 1.18-1.10 (2H, m, $C^{14}H_2$), 1.05 (18H, d, *J* = 7.4 Hz, 6 x $C^{22}H_3$), 0.72 (3H, t, *J* = 7.3 Hz, $C^{15}H_3$);

^{13}C NMR ($(CD_3)_2SO$) δ 156.3 (C), 155.5 (C), 144.7 (C^2), 141.7 (C^6), 140.7 (C), 139.6 (C), 130.7 (2 x C^{18}), 130.0 (q, *J* = 31.7 Hz, C^{10}), 129.5 (2 x C^8), 128.7 (C^5), 126.9 (C), 125.8 (q, *J* = 4.0 Hz, 2 x C^9), 123.9 (q, *J* = 272.6 Hz, C^{11}), 120.1 (2 x C^{19}), 62.5 (C^{16}), 31.2 (C^{13}), 29.4 (C^{12}), 21.4 (C^{14}), 17.7 (6 x C^{22}), 13.3 (C^{15}), 12.0 (3 x C^{21});

^{19}F NMR ($(CD_3)_2SO$) δ –61.3;

IR (neat): 2965, 1607, 1512, 1463, 1321, 1268, 1169, 1127, 1069, 1014, 905, 882 cm^{-1}.

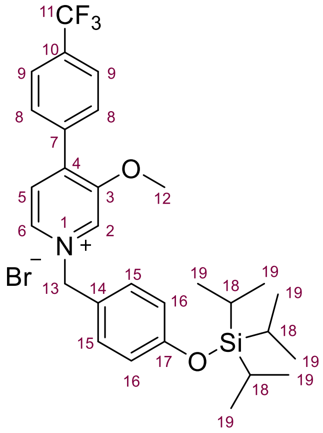

3-(Methoxy)-4-(4-trifluoromethyl)phenyl)-1-(4-((triisopropylsilyl)oxy)benzyl)pyridine-1-ium Bromide (2n)

The title compound was prepared using **General Procedure E** with pyridine **1n** (180 mg, 0.7 mmol) to give *salt* **2n** (404 mg, 97% yield) as a white solid.

m.p. (acetone): 214-216 °C;

HRMS (ESI): Exact mass calculated for $C_{29}H_{37}O_2NF_3{}^{28}Si$ $[M]^+$ calc: 516.25402, found: 516.25391;

^{1}H NMR ($(CD_3)_2SO$) δ 9.13 (1H, d, *J* = 1.3 Hz, C^2**H**), 8.82 (1H, dd, *J* = 6.2, 1.3 Hz, C^6**H**), 8.11 (1H, d, *J* = 6.2 Hz, C^5**H**), 7.91 (2H, d, *J* = 8.4 Hz, 2 x C^9**H**), 7.87 (2H, d, *J* = 8.5 Hz, 2 x C^8**H**), 7.53 (2H, d, *J* = 8.6 Hz, 2 x C^{15}**H**), 6.92 (2H, d, *J* = 8.5 Hz, 2 x C^{16}**H**), 5.79 (2H, s, C^{13}**H**$_2$), 4.05 (3H, s, C^{12}**H**$_3$), 1.23 (3H, ddt, *J* = 14.0, 9.7, 6.7 Hz, 3 x C^{18}**H**), 1.04 (18H, d, *J* = 7.4 Hz, 6 x C^{19}**H**$_3$);

^{13}C NMR ($(CD_3)_2SO$) δ 157.1 (C)¸155.8 (C)¸143.9 (C), 137.9 (C^6), 136.8 (C), 131.2 (q, *J* = 29.3 Hz, C^{10}), 131.1 (2 x C^{15}), 130.8 (2 x C^8), 130.4 (C^2), 128.7 (C^5), 127.1 (C), 125.8 (q, *J* = 4.0 Hz, 2 x C^9), 124.4 (q, *J* = 272.4 Hz, C^{11}), 120.6 (2 x C^{16}), 63.7 (C^{13}), 58.3 (C^{12}), 17.9 (6 x C^{19}), 12.6 (3 x C^{18});

^{19}F NMR ($(CD_3)_2SO$) δ –61.4;

IR (neat): 2981, 2349, 2161, 1510, 1462, 1406, 1323, 1271, 1163, 1121, 1074, 1039 cm^{-1}.

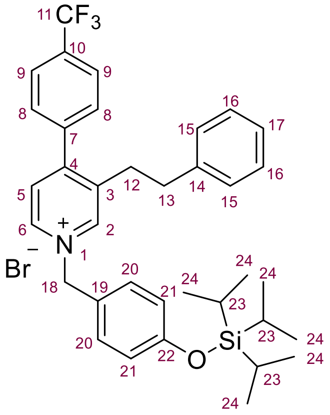

3-(2-Phenylethyl)-4-(4-trifluoromethyl)phenyl)-1-(4-((triisopropylsilyl)oxy)benzyl)pyridine-1-ium Bromide (2o)

The title compound was prepared using **General Procedure E** with pyridine **1o** (360 mg, 1.1 mmol) to give, after FCC (0-4% MeOH in CH_2Cl_2), *salt* **2o** (602 mg, 90% yield) as a white solid.

m.p. (acetone): 106-108 °C;

HRMS (ESI): Exact mass calculated for $C_{36}H_{43}ONF_3{}^{28}Si$ $[M]^+$ calc: 590.30605, found: 590.30575;

^{1}H NMR ($(CD_3)_2SO$) δ 9.38 (1H, d, *J* = 1.5 Hz, C^2**H**), 9.15 (1H, dd, *J* = 6.4, 1.5 Hz, C^6**H**), 8.07 (1H, d, *J* = 6.3 Hz, C^5**H**), 7.94 (2H, d, *J* = 7.7 Hz, 2 x C^9**H**), 7.66 (2H, d, *J* = 7.9 Hz, 2 x C^8**H**), 7.49 (2H, d, *J* = 8.6 Hz, 2 x C^{20}**H**), 7.19-7.13 (3H, m, 2 x C^{15}**H** + C^{17}**H**), 6.96-6.91 (4H, m, 2 x C^{16}**H** + 2 x C^{21}**H**), 5.80 (2H, s, C^{18}**H**$_2$), 3.06 (2H, dd, *J* = 8.8, 6.6 Hz, C^{12}**H**$_2$), 2.78 (2H, dd, *J* = 8.7, 6.7 Hz, C^{13}**H**$_2$), 1.27 (3H, app tt, *J* = 8.6, 6.9 Hz, 3 x C^{23}**H**), 1.06 (18H, d, *J* = 7.4 Hz, 6 x C^{24}**H**$_3$);

^{13}C NMR ($(CD_3)_2SO$) δ 156.4 (C), 155.7 (C), 145.1 (C^2), 141,9 (C^6), 139.7 (C), 139.6 (C), 139.4 (C), 130.7 (2 x C^{20}), 130.0 (q, *J* = 32.5 Hz, C^{10}), 129.5 (2 x C^8), 128.8 (C^5), 128.4 (2 x C^{15}), 128.2 (2 x C^{16}), 126.9 (C), 126.3 (C^{17}), 125.8 (q, *J* = 4.1 Hz, 2 x C^9), 124.0 (q, *J* = 272.5 Hz, C^{11}), 120.1 (2 x C^{21}), 62.6 (C^{18}), 34.8 (C^{13}), 31.6 (C^{12}), 17.8 (6 x C^{24}), 12.1 (3 x C^{23});

^{19}F NMR ($(CD_3)_2SO$) δ –61.3;

IR (neat): 3408, 2944, 2867, 2161, 1637, 1606, 1512, 1464, 1323, 1270, 1169, 1128 cm^{-1}.

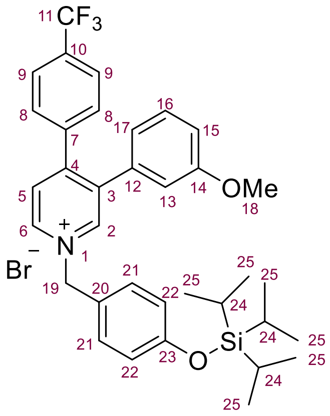

3-(3-Methoxyphenyl)-4-(4-trifluoromethyl)phenyl)-1-(4-((triisopropylsilyl)oxy)benzyl)pyridine-1-ium Bromide (2p)

The title compound was prepared using **General Procedure E** with pyridine **1p** (658 mg, 2.0 mmol) to give *salt* **2p** (1.07 g, 80% yield) as a white solid.

m.p. (acetone): 195-197 °C;

HRMS (ESI): Exact mass calculated for $C_{35}H_{41}O_2NF_3{}^{28}Si$ $[M]^+$ calc: 592.28532, found: 592.28485;

^{1}H NMR ($(CD_3)_2SO$) δ 9.48 (1H, d, *J* = 1.3 Hz, C^2**H**), 9.26 (1H, dd, *J* = 6.4, 1.4 Hz, C^6**H**), 8.29 (1H, d, *J* = 6.3 Hz, C^5**H**), 7.80 (2H, d, *J* = 8.2 Hz, 2 x C^9**H**), 7.61 (2H, d, *J* = 8.3 Hz, 2 x C^{21}**H**), 7.53 (2H, d, *J* = 8.1 Hz, 2 x C^8**H**), 7.31 (1H, t, *J* = 8.0 Hz, C^{16}**H**), 7.00 (1H, dd, *J* = 8.4, 2.6 Hz, C^{15}**H**), 6.95 (2H, d, *J* = 8.3 Hz, 2 x C^{22}**H**), 6.91 (1H, t, *J* = 2.0 Hz, C^{13}**H**), 6.78 (1H, d, *J* = 7.5 Hz, C^{17}**H**), 5.87 (2H, s, C^{19}**H**$_2$), 3.66 (3H, s, C^{18}**H**$_3$), 1.26 (3H, hept, *J* = 7.3 Hz, 3 x C^{24}**H**), 1.05 (18H, d, *J* = 7.4 Hz, 6 x C^{25}**H**$_3$);

^{13}C NMR ($(CD_3)_2SO$) δ 159.3 (C), 156.5 (C), 154.0 (C), 145.1 (C^2), 142.8 (C^6), 139.6 (C), 139.3 (C), 134.6 (C), 131.0 (2 x C^{21}), 130.3 (2 x C^8), 130.1 (C^{16}), 129.6 (q, *J* = 32.0 Hz, C^{10}), 129.2 (C^5), 126.8 (C), 125.6 (q, *J* = 4.2 Hz, 2 x C^9), 123.9 (q, *J* = 272.7 Hz, C^{11}), 122.1 (C^{17}), 120.1 (2 x C^{22}), 115.6 (C^{13}), 114.8 (C^{15}), 62.5 (C^{19}), 55.2 (C^{18}), 17.7 (6 x C^{25}), 12.1 (3 x C^{24});

^{19}F NMR ($(CD_3)_2SO$) δ –61.3;

IR (neat): 2950, 2869, 2161, 1633, 1607, 1580, 1511, 1469, 1427, 1404, 1320, 1268 cm^{-1}.

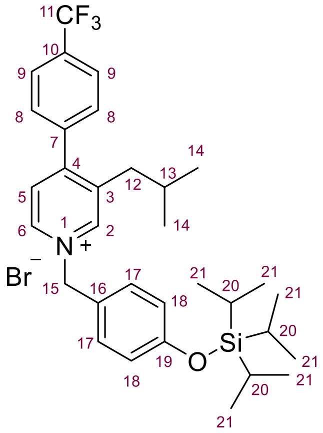

3-(2-Methylpropyl)-4-(4-trifluoromethyl)phenyl)-1-(4-((triisopropylsilyl)oxy)benzyl)pyridine-1-ium Bromide (2q)

The title compound was prepared using **General Procedure E** with pyridine **1q** (418 mg, 1.5 mmol) to give, after FCC (0-5% MeOH in CH_2Cl_2), *salt* **2q** (726 mg, 78% yield) as a white solid.

m.p. (acetone): 110-112 °C;

HRMS (ESI): Exact mass calculated for $C_{32}H_{43}ONF_3{}^{28}Si$ $[M]^+$ calc: 542.30605, found: 542.30585;

^{1}H NMR ($(CD_3)_2SO$) δ 9.14 (1H, d, J = 1.5 Hz, C^2**H**), 9.03 (1H, dd, J = 6.4, 1.4 Hz, C^6**H**), 7.93 (1H, d, J = 6.3 Hz, C^5**H**), 7.79 (2H, d, J = 8.4 Hz, 2 x C^9**H**), 7.59 (2H, d, J = 8.0 Hz, 2 x C^8**H**), 7.39 (2H, d, J = 8.6 Hz, 2 x C^{17}**H**), 6.77 (2H, d, J = 8.6 Hz, 2 x C^{18}**H**), 5.66 (2H, s, C^{19}**H**$_2$), 2.51 (2H, d, J = 7.3 Hz, C^{12}**H**$_2$), 1.47 (1H, hept, J = 6.8 Hz, C^{13}**H**), 1.14-1.02 (3H, m, 3 x C^{20}**H**), 0.88 (18H, d, J = 7.4 Hz, 6 x C^{21}**H**$_3$), 0.50 (6H, d, J = 6.6 Hz, 2 x C^{14}**H**$_3$);

^{13}C NMR ($(CD_3)_2SO$) δ 156.8 (C), 156.3 (C), 145.5 (C^2), 142.3 (C^6), 140.3 (C), 140.1 (C), 131.2 (2 x C^{17}), 130.4 (q, J = 32.1 Hz, C^{10}), 130.0 (2 x C^8), 129.4 (C^5), 127.5 (C), 126.2 (q, J = 4.1 Hz, 2 x C^9), 122.0 (q, J = 269.4 Hz, C^{11}), 120.6 (2 x C^{18}), 63.0 (C^{15}), 38.8 (C^{12}), 28.9 (C^{13}), 22.2 (2 x C^{14}), 18.2 (6 x C^{21}), 12.5 (3 x C^{20});

^{19}F NMR ($(CD_3)_2SO$) δ –61.3;

IR (neat): 3415, 2946, 2868, 2161, 1636, 1607, 1512, 1465, 1407, 1323, 1269, 1168 cm^{-1}.

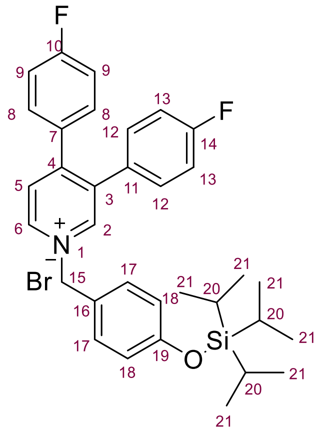

4-(3,4 Bis (4-fluorophenyl)pyridine)-1-(4-((triisopropylsilyl)oxy)benzyl)pyridin-1-ium Bromide (2r)

The title compound was prepared using **General Procedure E** with pyridine **1r** (400 mg, 1.5 mmol) to give *salt* **2r** (743 mg, 80% yield) as a white solid which was purified by FCC (0-5% MeOH in CH_2Cl_2).

m.p. (acetone): 156-158 °C;

HRMS (ESI): Exact mass calculated for $C_{33}H_{38}ONF_2{}^{28}Si$ $[M]^+$ calc: 530.26852, found: 530.26807;

^{1}H NMR ($(CD_3)_2SO$) δ 9.39 (1H, d, J = 1.5 Hz, C^2**H**), 9.19 (1H, dd, J = 6.5, 1.5 Hz, C^6**H**), 8.23 (1H, d, J = 6.4 Hz, C^5**H**), 7.59 (2H, d, J = 8.6 Hz, 2 x C^{17}**H**), 7.37-7.24 (8H, m, 2 x C^8**H** + 2 x C^9**H** + 2 x C^{12}**H** + 2 x C^{13}**H**), 6.94 (2H, d, J = 8.6 Hz, 2 x C^{18}**H**), 5.83 (2H, s, C^{15}**H_2**), 1.31-1.20 (3H, m, 3 x C^{20}**H**), 1.05 (18H, d, J = 7,4 Hz, 6 x C^{21}**H_3**);

^{13}C NMR ($(CD_3)_2SO$) δ 162.9 (d, J = 248.7 Hz, $C^{10/14}$), 162.5 (d, J = 246.8 Hz, $C^{10/14}$), 156.4 (C), 154.5 (C), 145.0 (C^2), 142.6 (C^6), 138.3 (C), 132.1 (d, J = 15.2 Hz, 2 x $C^{9/13}$), 132.0 (d, J = 15.3 Hz, 2 x $C^{9/13}$), 131.5 (C), 130.9 (2 x C^{17}), 130.2 (C), 129.0 (C^5), 126.8 (C), 120.1 (2 x C^{18}), 116.0 (2 x $C^{8/12}$), 115.8 (2 x $C^{8/12}$), 62.3 (C^{15}), 17.7 (6 x C^{21}), 12.0 (3 x C^{20});

^{19}F NMR ($CDCl_3$) δ –110.5, –112.1;

IR (neat): 2940, 2867, 2161, 1632, 1600, 1510, 1490, 1471, 1261, 1238, 1163, 1149 cm^{-1}.

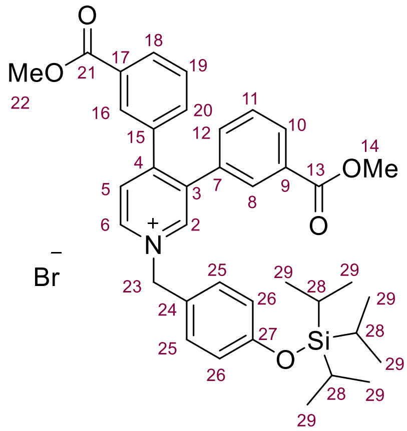

4-(3,4 bis (3- Methoxycarbonylphenyl)pyridine)-1-(4-((triisopropylsilyl)oxy)benzyl)pyridin-1-ium Bromide (2s)

The title compound was prepared using **General Procedure E** with pyridine **1s** (576 mg, 1.66 mmol) to give *salt* **2s** (1.04 g, 91% yield) as a white solid.

m.p. (acetone): 209–211 °C;

HRMS (ESI): Exact mass calculated for $C_{37}H_{44}O_5N^{28}Si$ $[M]^+$ calc: 610.29833, found: 610.29709;

^{1}H NMR ($(CD_3)_2SO$) δ : 9.50 (1H, d, *J* = 1.5 Hz C^2**H**), 9.25 (1H, dd, *J* = 6.4, 1.5 Hz, C^6**H**), 8.34 (1H, d, *J* = 6.4 Hz, 2 x C^5**H**), 8.03–7.97 (3H, m 3 x C^{Ar}**H**), 7.93–7.91 (1H, m C^{Ar}**H**), 7.58 (2H, d, *J* = 8.6 Hz, 2 x C^{25}**H**), 7.55–7.48 (3H, m 3 x C^{Ar}**H**), 7.46 (1H, ddd, *J* = 7.8, 1.6, 1.6 Hz C^{Ar}**H**), 6.94 (2H, d, J = 8.6 Hz, 2 x C^{26}**H**), 5.88 (2H, s, C^{23}**H$_2$**), 3.85 (3H, s, C^{22}**H$_3$**), 3.81 (3H, s, C^{14}**H$_3$**), 1.25 (3H, sep, *J* = 7.4 Hz, 3 x C^{28}**H**), 1.05 (18H, d, *J* = 7.4 Hz, 6 x C^{29}**H$_3$**);

^{13}C NMR ($(CD_3)_2SO$) δ 165.6 (C), 165.4 (C), 156.4 (C), 154.5 (C), 145.3 (C^2), 143.1 (C^6), 138.4, 135.5, 134.7, 134.2, 134.0 , 130.8, 130.5, 130.4, 130.2, 130.1, 130.0, 129.7, 129.3, 129.2, 129.1, 126.9, 120.0 (C^{26}), 62.4 (C^{23}), 52.4 (C^{14} and C^{22} confirmed by HSQC), 17.7 (C^{29}), 12.0 (C^{28});

IR (neat): 2947, 1722, 1510, 1251, 1172, 1112, 908, 883, 754, 677 cm^{-1}.

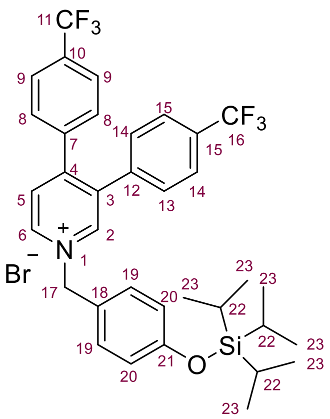

3-(3-Methoxyphenyl)-4-(4-trifluoromethyl)phenyl)-1-(4-((triisopropylsilyl)oxy)benzyl)pyridine-1-ium Bromide (2t)

The title compound was prepared using **General Procedure E** with pyridine **1t** (734 mg, 2.0 mmol) to give *salt* **2t** (1.32 g, 93% yield) as a white solid.

m.p. (acetone): 174-176 °C;

HRMS (ESI): Exact mass calculated for $C_{35}H_{38}ONF_6{}^{28}Si$ $[M]^+$ calc: 630.26214, found: 630.26166;

^{1}H NMR (($CD_3)_2SO$) δ 9.56 (1H, d, *J* = 1.4 Hz, C^2**H**), 9.34 (1H, dd, *J* = 6.5, 1.4 Hz, C^6**H**), 8.35 (1H, d, *J* = 6.4 Hz, C^5**H**), 7.83 (4H, app t, *J* = 8.7 Hz, 2 x C^9**H** + 2 x C^{14}**H**), 7.63 (2H, d, *J* = 8.6 Hz, 2 x C^{19}**H**), 7.55 (4H, app dd, *J* = 7.8, 5.4 Hz, 2 x C^8**H** + 2 x C^{13}**H**), 6.95 (2H, d, *J* = 8.6 Hz, 2 x C^{20}**H**), 5.90 (2H, s, 2 x C^{17}**H**$_2$), 1.26 (3H, dq, *J* = 14.1, 7.4 Hz, 3 x C^{22}**H**), 1.06 (18H, d, *J* = 7.4 Hz, 6 x C^{23}**H**$_3$);

^{13}C NMR (($CD_3)_2SO$) δ 156.9 (C), 154.2, (C), 145.9 (C^2), 154.5 (C^6), 139.5 (C), 138.5 (C), 138.2 (C), 131.5 (2 x C^8), 131.4 (2 x C^{19}), 130.9 (2 x C^{13}), 130.5 (q, *J* = 32.0 Hz, C), 130.3 (q, *J* = 32.4 Hz, C), 129.8 (C^5), 127.2 (C), 126.1 (app m, 2 x C^9 + 2 x C^{15}), 124.4 (q, *J* = 272.3 Hz, C^{11}), 124.3 (q, *J* = 272.8 Hz, C^{16}), 120.5 (2 x C^{20}), 63.0 (C^{17}), 18.2 (6 x C^{23}), 12.5 (3 x C^{22});

^{19}F NMR (($CD_3)_2SO$) δ –61.2, –61.3;

IR (neat): 2981, 2161, 1607, 1512, 1464, 1407, 1322, 1276, 1167, 1128, 1071, 1017 cm^{-1}.

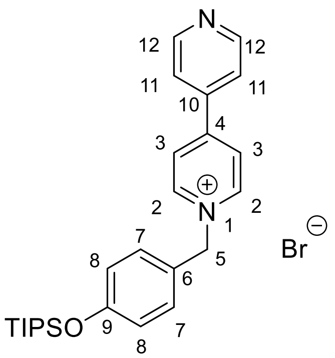

***N*-(4-((Triisopropylsilyl)oxy)benzyl)-[4,4'-bipyridin]ium Bromide (2u)**

To a stirred solution of 4,4'-bipyridine (937.1 mg, 6.0 mmol, 1.5 equiv.) in 50 mL of dioxane was added (4-(bromomethyl)phenoxy)triisopropylsilane (1372 mg, 4.0 mmol,

1.0 equiv.) at room temperature. The reaction was left stirring at room temperature for 3 days. The solvent was removed under vacuum, and then 100 mL of ether was added. The resulting suspension was sonicated (5 min) then filtered. The resultant solid was washed with diethyl ether and dried under vacuum for one hour to give the pyridinium bromide salt **2u** (1764 mg, 88% yield) as a white solid.

m.p.: 203-205 °C;

HRMS (ESI): Exact mass calculated for $C_{26}H_{35}ON_2{}^{28}Si$ $[M]^+$ m/z: 419.25132, found: 419.25140;

^{1}H NMR (400 MHz, DMSO-d_6) δ 9.34 (2H, d, J = 6.9 Hz, 2 x C^2**H**), 8.86 (2H, d, J = 6.2 Hz, 2 x C^{12}**H**), 8.64 (2H, d, J = 7.0 Hz, 2 x C^3**H**), 8.02 (2H, d, J = 6.2 Hz, 2 x C^{11}**H**), 7.52 (2H, d, J = 8.6 Hz, 2 x C^7**H**), 6.93 (2H, d, J = 8.6 Hz, 2 x C^8**H**), 5.82 (2H, s, C^5**H_2**), 1.31 – 1.19 (3H, m, 3 x TIPS-C**H**), 1.04 (18H, d, J = 7.5 Hz, 6 x TIPS-C**H_3**);

^{13}C NMR (101 MHz, DMSO-d_6) δ 156.4 (C), 152.7 (C), 151.0 (2 x C^{12}), 145.1 (2 x C^2), 140.8 (C), 130.7 (2 x C^7), 126.8 (C), 125.8 (2 x C^3), 122.0 (2 x C^{11}), 120.1 (2 x C^8), 62.5 (C^5), 17.7 (6 x TIPS-CH_3), 12.0 (3 x TIPS-CH);

IR (neat) (cm^{-1}): 1636, 1605, 1509, 1459, 1269, 1154, 909, 882, 820, 806, 757, 709, 677, 636.

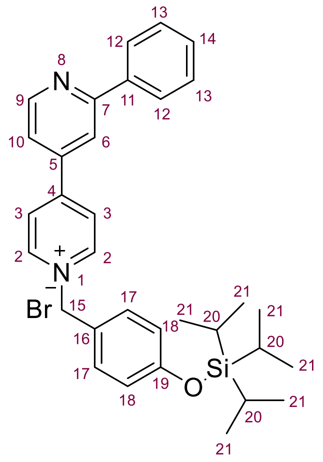

4-(4-(4-(2-Phenyl)-pyridine)-1-(4-((triisopropylsilyl)oxy)benzyl)pyridin-1-ium Bromide (2v)

The title compound was prepared using **General Procedure E** with pyridine **1v** (232 mg, 1 mmol) to give *salt* **2v** (459 mg, 80% yield) as a cream solid.

m.p. (acetone): greater than 300 °C;

HRMS (ESI): Exact mass calculated for $C_{32}H_{39}ON_2{}^{28}Si$ $[M]^+$ calc: 495.2826, found: 495.2822;

^{1}H NMR ($(CD_3)_2SO$) δ 9.40 (2H, d, *J* = 7.0 Hz, 2 x C^2**H**), 8.93 (1H, dd, *J* = 5.2, 0.8 Hz, C^9**H**), 8.79 (2H, d, *J* = 7.1 Hz, 2 x C^3**H**), 8.54 (1H, dd, *J* = 1.7, 0.9 Hz, C^6**H**), 8.27-8.23 (2H, m, 2 x C^{13}**H**), 7.97 (1H, dd, *J* = 5.2, 1.7 Hz, C^{10}**H**), 7.57-7.48 (5H, m, 2 x C^{17}**H** + 2 x C^{12}**H** + C^{14}**H**), 6.94 (2H, d, *J* = 8.6 Hz, 2 x C^{18}**H**), 5.85 (2H, s, C^{15}**H**$_2$), 1.30-1.20 (3H, m, 3 x C^{20}**H**), 1.04 (18H, d, *J* = 7.4 Hz, 6 x C^{21}**H**$_3$);

^{13}C NMR ($(CD_3)_2SO$) δ 157.5 (C), 156.4 (C), 152.9 (C), 150.9 (C^9), 145.0 (C + 2 x C^2), 142.1 (C), 137.9 (C), 130.7 (2 x C^{17}), 129.7 (C^{14}), 128.8 (2 x C^{12}), 126.9 (2 x C^{13}), 126.1 (2 x C^3), 120.6 (C^{10}), 120.1 (2 x C^{18}), 118.6 (C^6), 62.4 (C^{15}), 17.7 (6 x C^{21}), 12.0 (3 x C^{20});

IR (neat): 3011, 2942, 2867, 2161, 1641, 1605, 1545, 1507, 1460, 1396, 1354, 1272 cm^{-1}.

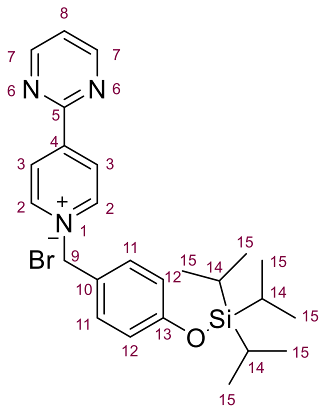

4-(4-(2-Pyrimidine)-1-(4-((triisopropylsilyl)oxy)benzyl)pyridin-1-ium Bromide (2w)

The title compound was prepared using **General Procedure E** with pyridine **1w** (134 mg, 0.85 mmol) to give *salt* **2w** (325 mg, 76% yield) as a white solid.

m.p. (acetone): 200-202 °C;

HRMS (ESI): Exact mass calculated for $C_{25}H_{34}ON_3{}^{28}Si$ $[M]^+$ calc: 420.24657 found: 420.24606;

^{1}H NMR ($(CD_3)_2SO$) δ 9.31 (2H, d, *J* = 6.9 Hz, 2 x C^2**H**), 9.14 (2H, d, *J* = 4.9 Hz, 2 x C^7**H**), 8.92 (2H, d, *J* = 6.9 Hz, 2 x C^3**H**), 7.76 (1H, t, *J* = 4.9 Hz, C^8**H**), 7.50 (2H, d, *J* = 8.6 Hz, 2 x C^{11}**H**), 6.93 (2H, d, *J* = 8.6 Hz, 2 x C^{12}**H**), 5.88 (2H, s, C^9**H**$_2$), 1.30-1.20 (3H, m, 3 x C^{14}**H**), 1.04 (18H, d, *J* = 7.4 Hz, 6 x C^{15}**H**$_3$);

^{13}C NMR ($(CD_3)_2SO$) δ 158.60 (2 x C^7), 158.57 (C)¸156.4 (C), 151.7 (C)¸145.6 (2 x C^2), 130.8 (2 x C^{11}), 126.7 (C), 125.8 (2 x C^3), 123.0 (C^8), 120.2 (2 x C^{12}), 62.7 (C^9), 17.7 (6 x C^{15}), 12.0 (3 x C^{14});

IR (neat): 2944, 2864, 2161, 1639, 1607, 1556, 1513, 1467, 1401, 1285, 1209, 1176 cm^{-1}.

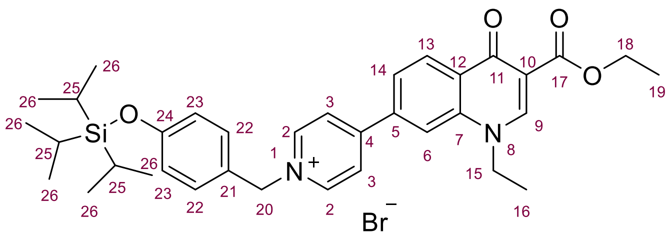

Rosoxacin Methyl Ester Salt (2x)

The title compound was prepared using **General Procedure E** with pyridine **1x** (322 mg, 1.0 mmol) to give *salt* **2x** (565 mg, 85% yield) as a yellow solid.

m.p. (acetone): 190-192 °C;

HRMS (ESI): Exact mass calculated for $C_{35}H_{45}O_4N_2{}^{28}Si$ $[M]^+$ calc: 585.31431, found: 585.31421;

^{1}**H NMR** ($(CD_3)_2SO$) δ 9.32 (2H, d, *J* = 6.5 Hz, 2 x C^{2}**H**), 8.76 (1H, s, C^{9}**H**), 8.69 (2H, d, *J* = 6.4 Hz, 2 x C^{3}**H**), 8.40 (1H, d, *J* = 8.5 Hz, C^{14}**H**), 8.30 (1H, s, C^{6}**H**), 8.03 (1H, d, *J* = 8.5 Hz, C^{13}**H**), 7.52 (2H, d, *J* = 7.9 Hz, 2 x C^{22}**H**), 6.92 (2H, d, *J* = 8.3 Hz, 2 x C^{23}**H**), 5.82 (2H, s, C^{20}**H**$_2$), 4.56 (2H, q, *J* = 7.1 Hz, C^{18}**H**$_2$), 4.22 (2H, q, *J* = 7.1 Hz, C^{15}**H**$_2$), 1.39 (3H, t, *J* = 7.0 Hz, C^{19}**H**$_3$), 1.30-1.18 (6H, m, C^{16}**H**$_3$ + 3 x C^{25}**H**), 1.03 (18H, d, *J* = 7.5 Hz, 6 x C^{26}**H**$_3$);

^{13}**C NMR** ($(CD_3)_2SO$) δ 172.2 (C^{17}), 164.4 (C^{11}), 156.4 (C), 154.0 (C), 149.9 (C^{9}), 144.7 (2 x C^{2}), 139.1 (C), 137.7 (C), 130.7 (2 x C^{22}), 129.8 (C), 127.9 (C^{14}), 127.0 (C), 126.3 (2 x C^{3}), 124.0 (C^{13}), 120.1 (2 x C^{23}), 117.5 (C^{6}), 111.0 (C), 62.3 (C^{20}), 59.9 (C^{15}), 48.1 (C^{18}), 17.7 (6 x C^{26}), 14.6 (C^{19}), 14.3 (C^{16}), 12.0 (3 x C^{25});

IR (neat): 3411, 2943, 2866, 2161, 1714, 1635, 1608, 1565, 1542, 1511, 1458, 1365 cm^{-1}.

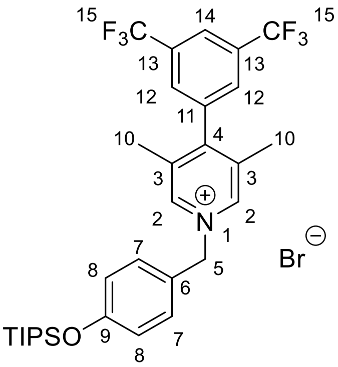

4-(3,5-bis(Trifluoromethyl)phenyl)-3,5-dimethyl-*N*-(4-((triisopropylsilyl)oxy)benzyl)pyridinium Iodide (3i)

The title compound was prepared according to **General Procedure E** using 4-(3,5-bis(trifluoromethyl)phenyl)-3,5-dimethylpyridine (319.3 mg, 1.0 mmol) to give *salt* **3i** (589.7 mg, 89% yield) as a white solid.

m.p.: 249-251 °C;

HRMS (ESI): Exact mass calculated for $C_{31}H_{38}OF_6N^{28}Si$ $[M]^+$ m/z: 582.26214, found: 582.26190;

^{1}H NMR (400 MHz, $CDCl_3$) δ 9.53 (2H, s, 2 x C^2**H**), 8.01 (1H, s, C^{14}**H**), 7.69 (2H, d, J = 8.4 Hz, 2 x C^7**H**), 7.64 (2H, s, 2 x C^{12}**H**), 6.88 (2H, d, J = 7.5 Hz, 2 x C^8**H**), 6.21 (2H, s, 2 x C^5**H_2**), 2.22 (6H, s, 2 x C^{10}**H_3**), 1.26 – 1.14 (3H, m, 3 x TIPS-C**H**), 1.04 (18H, d, J = 7.4 Hz, 6 x TIPS-C**H_3**);

^{13}C NMR (101 MHz, $CDCl_3$) δ 157.8 (C), 154.8 (C), 142.9 (2 x C^2), 137.4 (2 x C^3), 136.6 (C), 133.3 (q, J = 34.0 Hz, 2 x C^{13}), 131.6 (2 x C^7), 127.7 (2 x C^{12}), 125.3 (C), 123.6 (C^{14}), 122.8 (d, J = 273.4 Hz, 2 x C^{15}), 121.0 (2 x C^8), 63.4 (C^5), 18.2 (2 x C^{10}), 17.9 (6 x TIPS-CH_3), 12.7 (3 x TIPS-CH);

^{19}F NMR (377 MHz, $CDCl_3$) δ –62.9;

IR (neat) (cm^{-1}): 1509, 1375, 1277, 1258, 1165, 1153, 1139, 1108, 902, 885, 845, 714, 700, 681, 647.

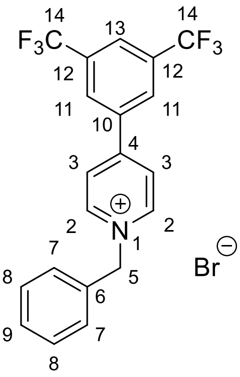

N-Benzyl-4-(3,5-bis(trifluoromethyl)phenyl)pyridinium Bromide (S11)

A mixture of 4-(3,5-bis(trifluoromethyl)phenyl)pyridine (582.4 mg, 2.0 mmol 1.0 equiv.) and benzyl bromide (0.36 mL, 3.0 mmol, 1.5 equiv.) in 5 mL of dioxane was stirred at 90 °C for 16 hours. The mixture was allowed to cool to room temperature, then the solvent was removed under reduced pressure and 25 mL of diethyl ether was added. The resulting suspension was sonicated (5 min) then filtered. The resultant solid was washed with diethyl ether and dried under vacuum for one hour to give the pyridinium bromide salt **S11** (869 mg, 94 % yield) as a white solid.

m.p.: 272-275 °C;

HRMS (ESI): Exact mass calculated for $C_{20}H_{14}NF_6$ $[M]^+$ m/z: 382.10250, found: 382.10266;

^{1}H NMR (400 MHz, DMSO-d_6) δ 9.50 (2H, d, J = 7.0 Hz, 2 x C^2**H**), 8.80 (2H, d, J = 7.0 Hz, 2 x C^3**H**), 8.73 (2H, s, 2 x C^{11}**H**), 8.37 (1H, s, C^{13}**H**), 7.65 (2H, dd, J = 7.8, 1.7 Hz, 2 x C^{Ar}**H**), 7.49 – 7.40 (3H, m, 3 x C^{Ar}**H**), 6.00 (2H, s, 2 x C^5**H_2**);

^{13}C NMR (101 MHz, DMSO-d_6) δ 152.3 (C^4), 145.0 (2 x C^2), 136.6 (2 x C^{13}), 134.5 (C), 131.33 (q, J = 33.4 Hz, 2 x C^{12}), 129.4 (m, 2 x C^{11}), 129.3 (C^{Ar}), 129.2 (2 x C^{Ar}), 128.8 (2 x C^{Ar}), 126.5 (2 x C^3), 125.0 (m, C^{13}), 123.01 (d, J = 273.1 Hz, 2 x C^{14}). 62.6 (C^5);

^{19}F NMR (376 MHz, DMSO-d_6) δ –61.2;

IR (neat) (cm^{-1}): 1634, 1378, 1285, 1275, 1192, 1171, 1128, 1058, 911, 868, 847, 819, 746, 735, 713, 695, 684, 633.

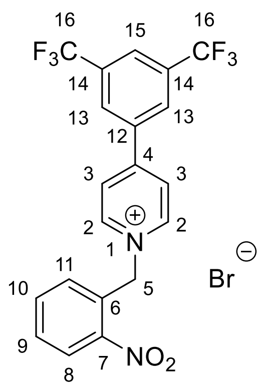

4-(3,5-Bis(trifluoromethyl)phenyl)-*N*-(2-nitrobenzyl)pyridinium Bromide (S12)

A mixture of 4-(3,5-bis(trifluoromethyl)phenyl)pyridine (582.4 mg, 2.0 mmol 1.0 equiv.) and 1-(bromomethyl)-2-nitrobenzene (648.1 mg, 3.0 mmol, 1.5 equiv.) in 5 mL of dioxane was stirred at 90 °C for 16 hours. The mixture was allowed to cool to room temperature, then the solvent was removed under reduced pressure and 25 mL of diethyl ether was added. The resulting suspension was sonicated (5 min) then filtered. The resultant solid was washed with diethyl ether and dried under vacuum for one hour to give the pyridinium bromide salt **S12** (943 mg, 93 % yield) as a white solid.

m.p.: 266-268 °C;

HRMS (ESI): Exact mass calculated for $C_{20}H_{13}O_2N_2F_6$ $[M]^+$ m/z: 427.08757, found: 427.08738;

^{1}H NMR (400 MHz, DMSO-d_6) δ 9.31 (2H, d, J = 6.9 Hz, 2 x C^2**H**), 8.88 (2H, d, J = 6.9 Hz, 2 x C^3**H**), 8.79 (1H, s, C^{13}**H**), 8.44 (1H, s, C^{15}**H**), 8.29 (1H, dd, J = 8.0, 1.5 Hz, C^8**H**), 7.78 (2H, dtd, J = 26.3, 7.7, 1.5 Hz, 2 x C^{Ar}**H**), 7.18 (1H, dd, J = 7.7, 1.5 Hz, C^{11}**H**), 6.34 (2H, s, C^5**H**$_2$);

^{13}C NMR (101 MHz, DMSO-d_6) δ 152.7 (C), 147.5 (C), 145.9 (2 x C^2), 136.5 (C), 135.0 (C^{Ar}), 131.4 (q, J = 33.3 Hz, 2 x C^{14}), 130.5 (C^{Ar}), 129.9 (C^{11}), 129.6 (C^{Ar}), 129.5 (2 x C^{13}), 126.4 (2 x C^3), 125.7 (C^8), 125.3 (C^{15}), 123.1 (q, J = 273.2 Hz, 2 x C^{16}), 60.2 (C^5);

^{19}F NMR (377 MHz, DMSO-d_6) δ –61.1;

IR (neat) (cm^{-1}): 1638, 1531, 1382, 1344, 1276, 1189, 1166, 1127, 1063, 906, 858, 838, 796, 731, 719, 702, 682.

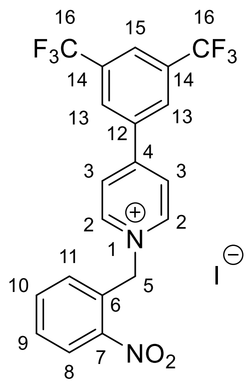

4-(3,5-Bis(trifluoromethyl)phenyl)-*N*-(2-nitrobenzyl)pyridinium Iodide (S13)

A mixture of 4-(3,5-bis(trifluoromethyl)phenyl)pyridine (582.4 mg, 2.0 mmol 1.0 equiv.) and 1-(iodomethyl)-2-nitrobenzene (789.1 mg, 3.0 mmol, 1.5 equiv.) in 5 mL of acetone was stirred at room temperature for 16 hours in the dark. The solvent was removed under reduced pressure, followed by addition of 30 mL of diethyl ether. The resulting suspension was sonicated (5 min) then filtered. The resultant solid was washed with diethyl ether and dried under vacuum for one hour to give the pyridinium iodide salt **S13** (1064 mg, 96% yield) as a yellow solid.

m.p.: 233-235 °C;

HRMS (ESI): Exact mass calculated for $C_{20}H_{13}O_2N_2F_6$ $[M]^+$ m/z: 427.08757, found: 427.08731;

^{1}H NMR (400 MHz, DMSO-d_6) δ 9.30 (2H, d, J = 6.3 Hz, 2 x C^2**H**), 8.88 (2H, d, J = 6.3 Hz, 2 x C^3**H**), 8.78 (2H, s, 2 x C^{13}**H**), 8.41 (1H, s, C^{15}**H**), 8.29 (1H, d, J = 8.0 Hz, C^8**H**), 7.80 (2H, dt, J = 28.1, 7.6 Hz, 2 x C^{Ar}**H**), 7.22 (1H, d, J = 7.7 Hz, C^{11}**H**), 6.34 (2H, s, C^5**H**$_2$);

^{13}C NMR (101 MHz, DMSO-d_6) δ 152.6 (C), 147.4 (C), 145.8 (2 x C^2), 136.4 (C), 134.9 (C^{Ar}), 131.4 (q, J = 33.5 Hz, 2 x C^{14}), 130.4 (C^{Ar}), 130.0 (C^{11}), 129.4 (C), 129.4 (2 x C^{13}), 126.4 (2 x C^3), 125.5 (C^8), 125.1 (C^{15}), 123.0 (q, J = 273.3 Hz, 2 x C^{16}), 60.2 (C^5);

^{19}F NMR (377 MHz, DMSO-d_6) δ –61.2;

IR (neat) (cm^{-1}): 1643, 1534, 1384, 1339, 1280, 1176, 1164, 1123, 1061, 907, 870, 852, 837, 827, 811, 790, 728, 704, 682, 637, 611.

Preparation of 3-Methylated Pyridines

General Procedure G: The methylation of pyridinium salts

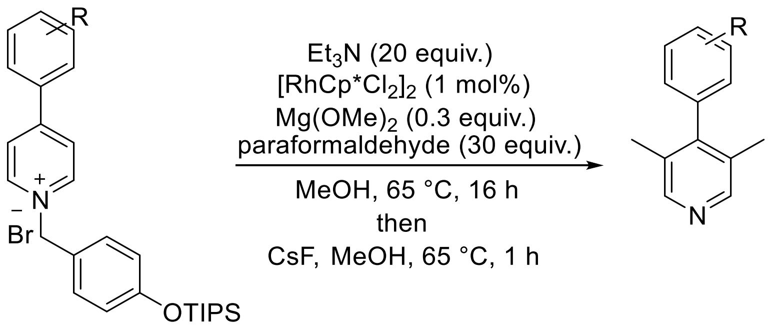

Pyridinium salt **3** (0.5 mmol), $[RhCp^*Cl_2]_2$ (3.1 mg, 0.005 mmol, 1 mol%), and paraformaldehyde (450 mg, 15 mmol, 30 equiv.) were added to a microwave vial. MeOH (3.43 mL), Et_3N (1.4 mL, 10 mmol, 20 equiv.) and magnesium methoxide solution (0.166 mL, 0.15 mmol, 0.9M solution in MeOH) were added and the vial was capped and heated at 65 °C for 16 hours. The solution was then removed from the oil bath, opened, and 150 mg of CsF (1.0 mmol) and 100 mg of thiourea (1.3 mmol) were added along with an addition 5 mL of MeOH. The open vial was returned to the oil bath and heated at 65 °C for a further 1 hour. The solution was concentrated, diluted with water (50 mL) and extracted with CH_2Cl_2 (3 x 50 mL). The combined organic layers were dried ($MgSO_4$), filtered and concentrated *in vacuo*. The crude material was purified by flash column chromatography to give *dimethylpyridine* **4**.

**If, after FCC, the dimethylpyridine product is still contaminated by non-pyridine impurities, additional purification can be achieved by dissolving the material in Et_2O (10 mL) and extracting with 2M HCl solution (2 x 10 mL). The acidic layers were combined, basified, then extracted with CH_2Cl_2 (2 x 10 mL). The organic layers were combined, dried ($MgSO_4$), filtered and concentrated to give pure *pyridine*.

3,5-Dimethyl-4-phenylpyridine (4a)

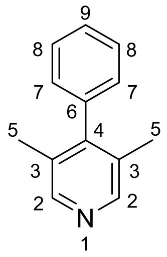

The title compound was prepared according to **General Procedure G** using *pyridinium* **2a** (249.3 mg, 0.5 mmol). The crude material was purified by FCC (CH_2Cl_2:EtOAc - 95:5 to 85:15) to give *pyridine* **4a** (36 mg, 38 % yield) as a white solid.

m.p.: 70-72 °C;

HRMS (ESI): Exact mass calculated for $C_{13}H_{14}N$ $[M+H]^+$ m/z: 184.11208, found: 184.11211;

^{1}H NMR (400 MHz, $CDCl_3$) δ 8.34 (2H, s, 2 x C^2**H**), 7.48 – 7.34 (3H, m, 3 x Ar-C**H**), 7.13 – 7.09 (2H, m, 2 x Ar-C**H**), 2.02 (6H, s, 2 x C^5**H**$_3$);

^{13}C NMR (101 MHz, $CDCl_3$) δ 149.4 (C), 148.5 (2 x C^2H), 138.2 (C^{Ar}), 130.9 (2 x C^3), 128.8 (2 x C^{Ar}), 128.1 (2 x C^{Ar}), 127.6 (C^{Ar}), 17.4 (2 x C^5);

IR (neat) (cm^{-1}): 1584, 1472, 1441, 1409, 1159, 877, 774, 755, 710, 666.

3,5-Dimethyl-4-(2-methylphenyl)pyridine (4b)

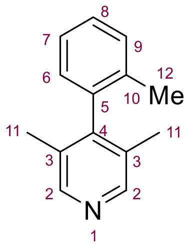

The title compound was prepared by **General Procedure G** using *pyridinium salt* **2b** (256 mg, 0.5 mmol) and was purified using FCC (0% - 5% EtOAc in CH_2Cl_2) and an acidic wash to give *pyridine* **4b** (40 mg, 41% yield) as a light yellow solid.

m.p. (Et_2O): 65-67 °C;

HRMS (ESI): Exact mass calculated for $C_{14}H_{16}N$ $[M+H]^+$ calc: 198.12773 found: 198.12782;

^{1}H NMR ($CDCl_3$) δ 8.37 (2H, s, 2 x C^2**H**), 7.45-7.19 (3H, m, C^6 + C^7 + C^8), 6.97 (1H, d, J = 6.3 Hz, C^9**H**), 1.97 (3H, s, C^{12}**H**$_3$), 1.95 (6H, s, 2 x C^{11}**H**$_3$);

^{13}C NMR ($CDCl_3$) δ 148.9 (C), 148.3 (2 x C^2), 137.5 (C), 134.6 (C), 130.3 (CH), 127.8 (CH), 127.6 (C^9), 126.3 (CH), 126.1 (C), 19.2 (C^{12}), 16.9 (2 x C^{11});

IR (neat): 1584, 1513, 1453, 1410, 1379, 1241, 1156, 1118, 1037, 987, 881, 810 cm^{-1}.

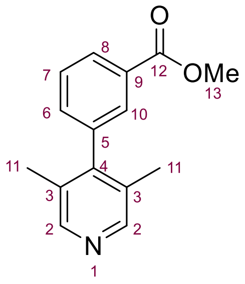

3,5-Dimethyl-4-(3-methoxycarbonyl)pyridine (4c)

The title compound was prepared by **General Procedure G** using *pyridinium salt* **2c** (278 mg, 0.5 mmol) and was purified using FCC (0% - 10% EtOAc in CH_2Cl_2) and an acidic wash to give *pyridine* **4c** (67 mg, 56% yield) as a white solid.

m.p. (Et_2O): 57-59 °C;

HRMS (ESI): Exact mass calculated for $C_{15}H_{16}O_2N$ $[M+H]^+$ calc: 242.11756 found: 242.11754;

^{1}H NMR ($CDCl_3$) δ 8.36 (2H, s, 2 x C^2**H**), 8.08 (1H, ddd, *J* = 7.8, 1.7, 1.2 Hz, C^8**H**), 7.82 (1H, td, *J* = 1.7, 0.6 Hz, C^{10}**H**), 7.55 (1H, td, *J* = 7.7, 0.6 Hz, C^7**H**), 7.33 (1H, ddd, *J* = 7.6, 1.8, 1.2 Hz, C^6**H**), 3.93 (3H, s, C^{13}**H**$_3$), 2.01 (6H, s, 2 x C^{11}**H**$_3$);

^{13}C NMR ($CDCl_3$) δ 166.8 (C^{12}), 148.5 (2 x C^2), 148.1 (C^4), 138.4 (C^9), 132.6 (C^6), 130.7 (C^5), 130.6 (2 x C^3), 129.2 (C^{10}), 128.9 (C^7), 128.8 (C^8), 52.3 (C^{13}), 17.3 (2 x C^{11});

IR (neat): 3353, 2955, 2161, 1713, 1591, 1443, 1383, 1303, 1284, 1273, 1242, 1199 cm^{-1}.

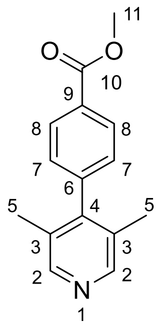

Methyl 4-(3,5-dimethylpyridin-4-yl)benzoate (4d)

The title compound was prepared according to **General Procedure G** using *pyridinium* **2d** (278.3 mg, 0.5 mmol). The crude material was purified by FCC (CH_2Cl_2:EtOAc - 95:5 to 85:15) to give *pyridine* **4d** (66 mg, 55 % yield) as a white solid.

m.p.: 100-102 °C;

HRMS (ESI): Exact mass calculated for $C_{15}H_{16}O_2N$ $[M+H]^+$ m/z: 242.11756 , found: 224.11742;

^{1}H NMR (400 MHz, $CDCl_3$) δ 8.34 (2H, s, 2 x C^2**H**), 8.14 – 8.10 (2H, m, 2 xC^8**H**), 7.22 – 7.17 (2H, m, 2 x C^7**H**), 3.93 (3H, s, C^{11}**H**$_3$), 1.99 (6H, s, 6 x C^5**H**$_3$);

^{13}C NMR (101 MHz, $CDCl_3$) δ 166.8 (C^{10}), 148.6 (2 x C^2), 148.4 (C^4), 143.0 (C^6), 130.4 (2 x C^3), 130.2 (2 x C^8), 129.6 (C^9), 128.3 (2 x C^7), 52.3 (C^{11}), 17.3 (2 x C^5);

IR (neat) (cm^{-1}): 1717, 1584, 1438, 1315, 1288, 1181, 1160, 1115, 1103, 862, 774, 760, 710.

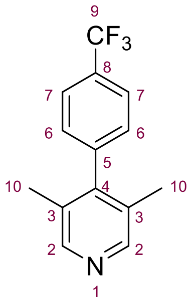

3,5-Dimethyl-4-(4-trifluoromethylphenyl)pyridine (4e)

The title compound was prepared by **General Procedure G** using *pyridinium salt* **2e** (283 mg, 0.5 mmol) and was purified using FCC (0% - 10% EtOAc in CH_2Cl_2) and an acidic wash to give *pyridine* **4e** (68 mg, 54% yield) as a white solid.

m.p. (Et_2O): 78-80 °C;

HRMS (ESI): Exact mass calculated for $C_{14}H_{13}NF_3$ [M+H] calc: 252.09946 found: 252.09958;

^{1}H NMR ($CDCl_3$) δ 8.38 (2H, s, 2 x C^2**H**), 7.75 (2H, dt, J = 7.9, 0.8 Hz, 2 x C^6**H**), 7.36-7.13 (2H, m, 2 x C^7**H**), 2.03 (6H, s, 2 x C^{10}**H**$_3$);

^{13}C NMR ($CDCl_3$) δ 148.6 (2 x C^2), 147.8 (C^4), 141.9 (C^5), 130.4 (2 x C^3), 130.1 (q, J = 30.2 Hz, C^8), 128.5 (2 x C^6), 125.8 (q, J = 3.7 Hz, 2 x C^7), 124.0 (q, J = 272.3 Hz, C^9), 17.2 (2 x C^{10});

^{19}F NMR ($CDCl_3$) δ –62.6;

IR (neat): 1617, 1581, 1445, 1404, 1319, 1160, 1121, 1068, 1030, 889, 839, 781 cm^{-1}.

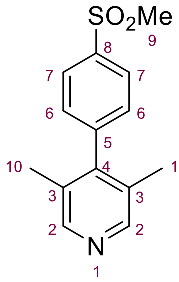

3,5-Dimethyl-4-(4-methylsulfonylphenyl)pyridine (4f)

The title compound was prepared by **General Procedure G** using *pyridinium salt* **2f** (288 mg, 0.5 mmol) and was purified using FCC (0% -

20% EtOAc in CH_2Cl_2) and an acidic wash to give *pyridine* **4f** (73 mg, 56% yield) as a white solid.

m.p. (Et_2O): 220-224 °C;

HRMS (ESI): Exact mass calculated for $C_{14}H_{16}O_2NS$ [M+H] calc: 262.08963 found: 262.08960;

^{1}H NMR ($CDCl_3$) δ 8.39 (2H, s, 2 x C^2**H**), 8.07 (2H, d, *J* = 8.4 Hz, 2 x C^7**H**), 7.37 (2H, d, *J* = 8.6 Hz, 2 x C^6**H**), 3.16 (3H, s, C^9**H**$_3$), 2.02 (6H, s, 2 x C^{10}**H**$_3$);

^{13}C NMR ($CDCl_3$) δ 148.6 (2 x C^2), 147.2 (C^8), 144.0 (C^4), 139.9 (C^5), 130.2 (2 x C^3), 129.3 (2 x C^6), 128.0 (2 x C^7), 44.5 (C^9), 17.3 (2 x C^{10});

IR (neat): 2918, 2161, 1584, 1300, 1149, 1089, 979, 879, 839, 793, 768, 731 cm^{-1}.

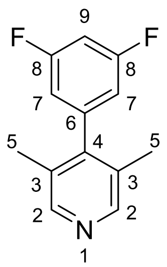

4-(3,5-Difluorophenyl)-3,5-dimethylpyridine (4g)

The title compound was prepared according to **General Procedure G** using *pyridinium* **2g** (267.3 mg, 0.5 mmol). The crude material was purified by FCC (CH_2Cl_2:EtOAc - 95:5 to 85:15) and acid wash to give *pyridine* **4g** (73 mg, 67 % yield) as a white solid.

m.p.: 84-87 °C;

HRMS (ESI): Exact mass calculated for $C_{13}H_{12}NF_2$ $[M+H]^+$ m/z: 220.09323, found: 220.09331;

^{1}H NMR (400 MHz, $CDCl_3$) δ 8.35 (2H, s, 2 x C^2**H**), 6.85 (1H, tt, *J* = 9.0, 2.3 Hz, C^9**H**), 6.70 – 6.63 (2H, m, 2 x C^7**H**), 2.04 (6H, s, 3 x C^5**H**$_3$);

^{13}C NMR (101 MHz, $CDCl_3$) δ 163.4 (dd, *J* = 250.2, 12.8 Hz, 2 x C^8), 148,7 (2 x C^2), 147.1 (m, C^4), 141.46 (m, C^6), 130.5 (2 x C^3) 111.3 (m, 2 x C^7), 103.34 (t, *J* = 25.1 Hz, C^9) 17.2 (2 x C^5);

^{19}F NMR (377 MHz, $CDCl_3$) δ –108.7;

IR (neat) (cm^{-1}): 1625, 1586, 1462, 1429, 1409, 1380, 1330, 1119, 979, 876, 854, 759, 725, 698, 608.

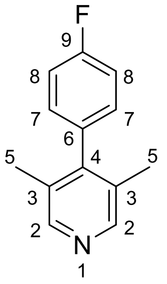

4-(4-Fluorophenyl)-3,5-dimethylpyridine (4h)

The title compound was prepared according to **General Procedure G** using *pyridinium* **2h** (258.3 mg, 0.5 mmol). The crude material was purified by FCC (CH_2Cl_2:EtOAc - 95:5 to 85:15) to give *pyridine* **4h** (45 mg, 45 % yield) as a white solid.

m.p.: 47-49 °C;

HRMS (ESI): Exact mass calculated for $C_{13}H_{13}NF$ $[M+H]^+$ m/z: 202.10265, found: 202.10269;

^{1}H NMR (400 MHz, $CDCl_3$) δ 8.33 (2H, s, 2 x C^2**H**), 7.17 – 7.05 (4H, m, 4 x Ar-C**H**), 2.01 (6H, s, 3 x C^5**H**$_3$);

^{13}C NMR (101 MHz, $CDCl_3$) δ 162.2 (d, *J* = 246.7 Hz, C^9), 148.5 (2 x C^2), 148.4 (C^4), 134.0 (d, *J* = 3.6 Hz, C^6), 131.1 (2 x C^3), 129.9 (d, *J* = 8.0 Hz, 2 x C^7), 115.9 (d, *J* = 21.4 Hz, 2 x C^8), 17.4 (2 x C^5);

^{19}F NMR (377 MHz, $CDCl_3$) δ –114.6;

IR (neat) (cm^{-1}): 1601, 1510, 1474, 1379, 1218, 1161, 837, 817, 761, 619.

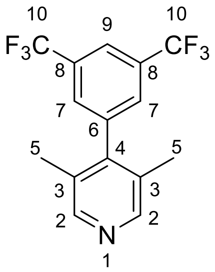

4-(3,5-Bis(trifluoromethyl)phenyl)-3,5-dimethylpyridine (4i)

The title compound was prepared according to **General Procedure G** using using *pyridinium* **2i** (317.3 mg, 0.5 mmol) with the addition of 1 equiv. of NaI. The crude material was purified by FCC (CH_2Cl_2:EtOAc - 100:0 to 90:10) and acid wash to give *pyridine* **4i** (97 mg, 61 % yield) as a white solid.

m.p.: 75-78 °C;

HRMS (ESI): Exact mass calculated for $C_{15}H_{12}NF_6$ $[M+H]^+$ m/z: 320.08685, found: 320.08710;

^{1}H NMR (400 MHz, $CDCl_3$) δ 8.40 (2H, s, 2 x C^{2}**H**), 7.93 (1H, s, C^{9}**H**), 7.63 (2H, s, 2 x C^{7}**H**), 2.02 (6H, s, 3 x C^{5}**H**$_3$);

^{13}C NMR (101 MHz, $CDCl_3$) δ 149.0 (2 x C^{2}), 146.1 (C^{4}), 140.3 (C^{6}), 132.50 (q, *J* = 33.6 Hz, 2 x C^{8}), 130.4 (2 x C^{3}), 128.70 (q, *J* = 4.0 Hz, 2 x C^{7}), 123.25 (q, *J* = 272.8 Hz, 2 x C^{10}), 121.9 (h, *J* = 3.8 Hz, C^{9}), 17.4 (2 x C^{5});

^{19}F NMR (377 MHz, $CDCl_3$) δ –62.9;

IR (neat) (cm^{-1}): 1375, 1278, 1181, 1164, 1112, 1062, 1030, 906, 844, 716, 693, 680.

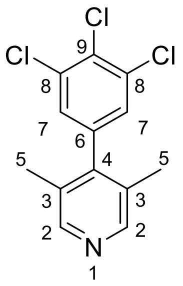

3,5-Dimethyl-4-(3,4,5-trichlorophenyl)pyridine (4j)

The title compound was prepared according to **General Procedure G** using *pyridinium iodide salt* **2j’** (324.5 mg, 0.5 mmol). The crude material was purified by FCC (CH_2Cl_2:EtOAc - 100:0 to 90:10) to give *pyridine* **4j** (86 mg, 60 % yield) as a white solid.

m.p.: 146-148 °C;

HRMS (ESI): Exact mass calculated for $C_{13}H_{11}N^{35}Cl_3$ $[M+H]^+$ m/z: 285.99516, found: 285.99509;

^{1}H NMR (400 MHz, $CDCl_3$) δ 8.35 (2H, s, 2 x C^{2}**H**), 7.17 (2H, s, 2 xC^{7}**H**), 2.04 (6H, s, 2 x C^{5}**H**$_3$);

^{13}C NMR (101 MHz, $CDCl_3$) δ 148.8 (2 x C^{2}), 145.8 (C), 138.2 (C), 134.9 (C), 131.1 (C), 130.5 (2 x C^{3}), 128.5 (2 x C^{7}), 17.3 (2 x C^{5});

IR (neat) (cm^{-1}): 1578, 1538, 1436, 1420, 1377, 1203, 1163, 877, 814, 756, 727, 705, 606.

(3,5-Dimethylpyridin-4-yl)(phenyl)methanone (4k)

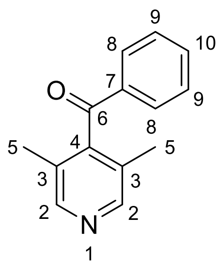

The title compound was prepared according to **General Procedure G** using *pyridinium* **2k'** (286.8 mg, 0.5 mmol) with addition of 1 equiv. of NaI. The crude material was purified by FCC (DCM:Acetone - 95:5 to 90:10) to give *pyridine* **4k** (43 mg, 41 % yield) as a white solid.

m.p.: 54-56 °C;

HRMS (ESI): Exact mass calculated for $C_{14}H_{14}ON$ $[M+H]^+$ m/z: 212.10699 , found: 212.10715;

^{1}H NMR (400 MHz, $CDCl_3$) δ 8.37 (2H, s, 2 x C^2**H**), 7.80 – 7.74 (2H, m, 2 x C^8**H**), 7.66 – 7.61 (1H, m, C^{10}**H**), 7.53 – 7.45 (2H, m, 2 x C^9**H**), 2.11 (6H, s, 2 x C^5**H**$_3$);

^{13}C NMR (101 MHz, $CDCl_3$) δ 197.7 (C^6), 148.8 (2 x C^2), 146.9 (C), 135.8 (C), 134.6 (C^{10}), 129.4 (2 x C^8), 129.3 (2 x C^9), 128.7 (2 x C^3), 16.3 (2 x C^5);

IR (neat) (cm^{-1}): 1666, 1593, 1579, 1452, 1283, 1261, 929, 874, 802, 774, 709, 685, 676, 617.

5-Methyl-3-phenyl-4-(4-trifluoromethylphenyl)pyridine (4l)

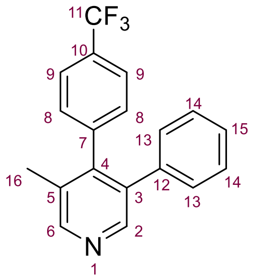

The title compound was prepared by **General Procedure G** using *pyridinium salt* **2l** (321 mg, 0.5 mmol) and was purified using FCC (0% - 4% EtOAc in CH_2Cl_2) to give *pyridine* **4l** (86 mg, 55% yield) as a white solid.

m.p. (Et_2O): 95-97 °C;

HRMS (ESI): Exact mass calculated for $C_{19}H_{15}NF_3$ $[M+H]^+$ calc: 314.11511 found: 314.11496;

^{1}H NMR ($CDCl_3$) δ 8.55 (1H, s, C^2**H**), 8.53 (1H, s, C^6**H**), 7.55 (2H, d, J = 8.5 Hz, 2 x C^9**H**), 7.23-7.20 (3H, m, 2 x C^{13}**H** + C^{15}**H**), 7.18 (2H, d, J = 8.0 Hz, 2 x C^8**H**), 7.06-7.02 (2H, m, 2 x C^{14}**H**), 2.17 (3H, s, C^{16}**H**$_3$);

^{13}C NMR ($CDCl_3$) δ 150.0 (C^{2}), 148.4 (C^{6}), 146,2 (C), 141.3 (C), 137.3 (C), 130.8 (C), 129.8 (2 x C^{8}), 129.7 (2 x C^{14}), 129.5 (q, *J* = 32.7 Hz, C^{10}); 129.4 (C), 128.1 (2 x C^{13}), 127.2 (C^{15}), 125.1 (q, *J* = 3.7 Hz, 2 x C^{9}), 124.0 (q, *J* = 272.1 Hz, C^{11}), 17.6 (C^{16});

^{19}F NMR ($CDCl_3$) δ –62.6;

IR (neat): 1617, 1578, 1446, 1403, 1319, 1160, 1123, 1110, 1069, 1028, 1016, 889 cm^{-1}.

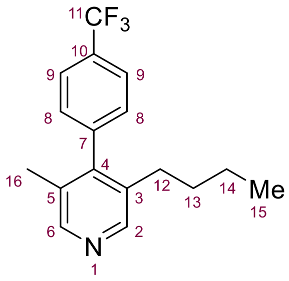

3-*n*-Butyl-5-methyl-4-(4-trifluoromethylphenyl)pyridine (4m)

The title compound was prepared by **General Procedure G** using *pyridinium salt* **2m** (311 mg, 0.5 mmol) and was purified using FCC (0% - 3% EtOAc in CH_2Cl_2) to give *pyridine* **4m** (85 mg, 58% yield) as a colourless oil.

HRMS (ESI): Exact mass calculated for $C_{17}H_{19}NF_3$ $[M+H]^+$ calc: 294.14641, found: 294.14621

^{1}H NMR ($CDCl_3$) δ 8.39 (1H, s, C^{2}**H**), 8.36 (1H, s, C^{6}**H**), 7.73 (2H, dt, *J* = 7.9, 0.8 Hz, 2 x C^{8}**H**), 7.29-7.25 (2H, m, 2 x C^{8}**H**), 2.36-2,30 (2H, m, $C^{12}H_2$), 1.99 (3H, s, $C^{16}H_3$), 1.40-1.31 (2H, m, $C^{13}H_2$), 1.22-1.12 (2H, m, $C^{14}H_2$), 0.76 (3H, t, *J* = 7.3 Hz, $C^{15}H_3$);

^{13}C NMR ($CDCl_3$) δ 148.2 (C^{2}), 148.0 (C^{6}), 147.6 (C), 141.6 (C), 135.1 (C), 130.7 (C), 129.9 (q, *J* = 32.6 Hz, C^{10}), 128.8 (2 x C^{8}), 125.6 (q, *J* = 3.7 Hz, 2 x C^{9}), 124.0 (q, *J* = 272.1 Hz, C^{11}), 33.1 (C^{13}), 30.2 (C^{12}), 22.3 (C^{14}), 17.4 (C^{16}), 13.6 (C^{15});

^{19}F NMR ($CDCl_3$) δ –62.6

IR (neat): 1584, 1404, 1322, 1164, 1124, 1106, 1068, 1031, 1015, 842, 756, 614 cm^{-1}.

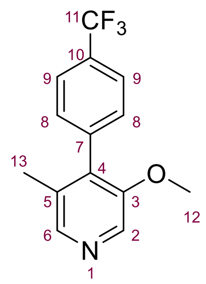

3-Methoxy-5-methyl-4-(4-trifluoromethylphenyl)pyridine (4n)

The title compound was prepared by **General Procedure G** using *pyridinium salt* **2n** (297.5 mg, 0.5 mmol) and was purified using FCC (0% - 2% EtOAc in CH_2Cl_2) to give *pyridine* **4n** (77 mg, 58% yield) as a white solid.

m.p. (Et_2O): 89-91 °C;

HRMS (ESI): Exact mass calculated for $C_{14}H_{13}ONF_3$ $[M+H]^+$ calc: 268.09438, found: 268.09435;

^{1}H NMR ($CDCl_3$) δ 8.24 (2H, app s, C^2**H** + C^6**H**), 7.72 (2H, d, *J* = 7.9 Hz, 2 x C^9**H**), 7.35 (2H, dt, *J* = 7.8, 0.8 Hz, 2 x C^8**H**), 3.82 (3H, s, C^{12}**H**$_3$), 2.09 (3H, d, *J* = 0.6 Hz, C^{13}**H**$_2$);

^{13}C NMR ($CDCl_3$) δ 152.4 (C), 148.6 (C), 144.3 ($C^{2/6}$), 138.6 (C), 136.6 (C), 131.5 ($C^{2/6}$), 129.9 (q, *J* = 32.3 Hz, C^{10}), 129.7 (2 x C^8), 125.3 (q, *J* = 3.8 Hz, 2 x C^9), 124.1 (q, *J* = 272.3 Hz, C^{11}), 56.3 (C^{12}), 16.9 (C^{13});

^{19}F NMR ($CDCl_3$) δ –62.6;

IR (neat): 1617, 1580, 1462, 1422, 1404, 1320, 1289, 1275, 1146, 1105, 1089, 1065 cm^{-1}.

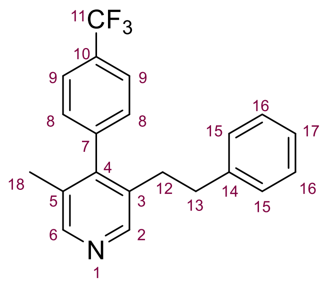

5-Methyl-3-(2-methylpropyl)-4-(4-trifluoromethylphenyl)pyridine (4o)

The title compound was prepared by **General Procedure G** using *pyridinium salt* **2o** (335 mg, 0.5 mmol) and was purified using FCC (0% - 2% EtOAc in CH_2Cl_2) to give *pyridine* **4o** (85 mg, 50% yield) as a cream solid.

m.p. (Et_2O): 58-60 °C;

HRMS (ESI): Exact mass calculated for $C_{21}H_{19}NF_3$ $[M+H]^+$ calc: 342.14641, found: 342.14627;

^{1}H NMR (CDCl$_3$) δ 8.41 (1H, s, C^{2}**H**), 8.40 (1H, s, C^{6}**H**), 7.72 (2H, d, *J* = 7.6 Hz, 2 x C^{9}**H**), 7.23-7.13 (5H, m, 2 x C^{8}**H** + 2 x C^{15}**H** + C^{17}**H**), 6.87-6.83 (2H, m, 2 x C^{16}**H**), 2.72-2.64 (4H, m, C^{12}**H**$_2$ + C^{13}**H**$_2$), 2.00 (3H, s, C^{18}**H**$_3$);

^{13}C NMR (CDCl$_3$) δ 148.4 (C^{2}), 148.3 (C^{6}), 147.9 (C), 141.3 (C), 140.7 (C), 134.0 (C), 130.7 (C), 130.0 (q, *J* = 32.7 HZ, C^{10}), 128.8 (2 x C^{8}), 128.4 (2 x C^{15}), 128.3 (2 x C^{16}), 126.1 (C^{17}), 125.6 (q, *J* = 3.7 Hz, 2 x C^{9}), 124.0 (q, *J* = 272.3 Hz, C^{11}), 37.3 (C^{12}), 32.7 (C^{13}), 17.4 (C^{18});

^{19}F NMR (CDCl$_3$) δ –62.6;

IR (neat): 1584, 1495, 1455, 1408, 1326, 1177, 1119, 1069, 1032, 887, 862, 850 cm^{-1}.

Structure Confirmation by 2D NOESY:

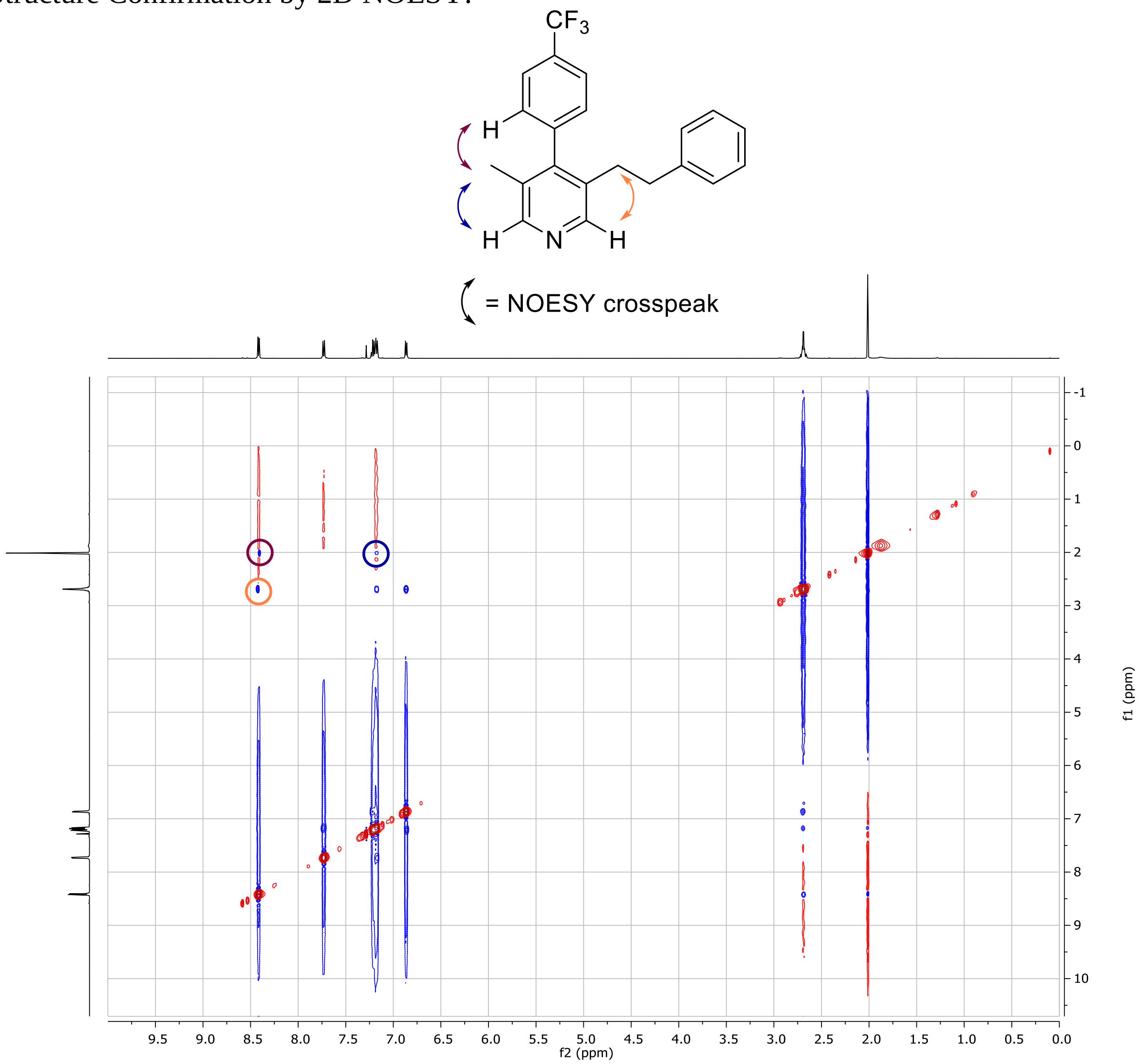

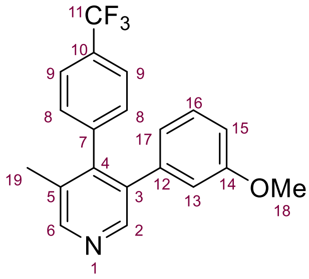

5-Methyl-3-(3-methoxyphenyl)-4-(4-trifluoromethylphenyl)pyridine (4p)

The title compound was prepared by **General Procedure G** using *pyridinium salt* **2p** (336 mg, 0.5 mmol) and was purified using FCC (0% - 3% EtOAc in CH_2Cl_2) to give *pyridine* **4p** (96 mg, 56% yield) as a colourless oil.

HRMS (ESI): Exact mass calculated for $C_{20}H_{17}ONF_3$ $[M+H]^+$ calc: 344.12568, found: 344.12558;

^{1}H NMR ($CDCl_3$) δ 8.54 (1H, d, *J* = 0.6 Hz, C^2**H**), 8.53 (1H, d, *J* = 0.7 Hz, C^6**H**), 7.57 (2H, dt, *J* = 7.9, 0.7 Hz, 2 x C^8**H**), 7.20 (2H, d, *J* = 7.9 Hz, 2 x C^9**H**), 7.17-7.08 (1H, m, C^{16}**H**), 6.75 (1H, ddd, *J* = 8.3, 2.6, 1.0 Hz, C^{17}**H**), 6.64 (1H, ddd, *J* = 7.6, 1.7, 1.0 Hz, C^{15}**H**), 6.55 (1H, dd, *J* = 2.6, 1.6 Hz, C^{13}**H**), 3.62 (3H, s, C^{18}**H**$_3$), 2.17 (3H, s, C^{19}**H**$_3$);

^{13}C NMR ($CDCl_3$) δ 159.1 (C), 150.1 (C^2), 148.3 (C^6), 146.1 (C), 141.4 (C), 138.6 (C), 135.8 (C), 130.8 (C), 129.8 (2 x C^8), 129.6 (q, *J* = 32.2 Hz, C^{10}), 129.1 (C^{16}), 125.2 (q, *J* = 3.8 Hz, 2 x C^9), 124.0 (q, *J* = 272.4 Hz, C^{11}), 122.3 (C^{15}), 115.2 (C^{13}), 113.2 (C^{17}), 55.1 (C^{18}), 17.6 (C^{19});

^{19}F NMR ($CDCl_3$) δ –62.6;

IR (neat): 1580, 1404, 1321, 1233, 1162, 1123, 1069, 1030, 1016, 841, 815, 782 cm^{-1}.

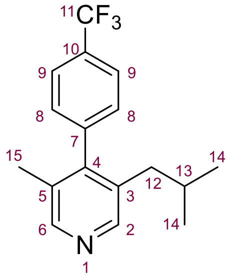

3-*iso*Butyl-5-methyl-4-(4-trifluoromethylphenyl)pyridine (4q)

The title compound was prepared by **General Procedure G** using *pyridinium salt* **2q** (311 mg, 0.5 mmol) and was purified using FCC (0% - 2% EtOAc in CH_2Cl_2) to give *pyridine* **4q** (77 mg, 53% yield) as a colourless oil.

HRMS (ESI): Exact mass calculated for $C_{17}H_{19}NF_3$ $[M+H]^+$ calc: 294.14641, found: 294.146236;

^{1}H NMR ($CDCl_3$) δ 8.37 (2H, app d, *J* = 3.1 Hz, C^{2}**H** + C^{6}**H**), 7.73 (2H, d, *J* = 8.6 Hz, 2 x C^{9}**H**), 7.26 (2H, d, *J* = 7.7 Hz, 2 x C^{8}**H**), 2.25 (2H, d, *J* = 7.3 Hz, C^{12}**H_2**), 1.99 (3H, s, C^{15}**H_3**), 1.61 (1H, dt, *J* = 13.6, 6.8 Hz, C^{13}**H**), 0.75 (6H, d, *J* = 6.6 Hz, 2 x C^{14}**H_3**);

^{13}C NMR ($CDCl_3$) δ 148.8 (C^{2}), 148.1 (C^{6}), 148.0 (C), 141.7 (C), 133.9 (C), 130.7 (C), 129.9 (q, *J* = 32.5 Hz, C^{10}), 129.0 (2 x C^{8}), 125.5 (q, *J* = 3.9 Hz, 2 x C^{9}), 124.1 (q, *J* = 272.2 Hz, C^{11}), 39.5 (C^{12}), 29.4 (C^{13}), 22.3 (2 x C^{14}), 17.5 (C^{15});

^{19}F NMR ($CDCl_3$) δ –62.6

IR (neat): 1583, 1466, 1404, 1322, 1164, 1124, 1106, 1068, 1031, 1013, 842, 755 cm^{-1}.

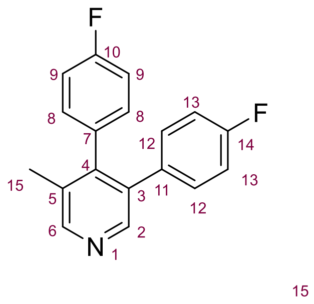

5-Methyl-3,4-(4-fluorophenyl)pyridine (4r)

The title compound was prepared by **General Procedure G** using *pyridinium salt* **2r** (305 mg, 0.5 mmol) and was purified using FCC (0% - 2% EtOAc in CH_2Cl_2) and an acidic wash to give *pyridine* **4r** (77 mg, 55% yield) as a rose coloured solid.

m.p. (Et_2O): 71-73 °C;

HRMS (ESI): Exact mass calculated for $C_{18}H_{14}N_2F$ $[M+H]^+$ calc: 282.1089 found: 282.1088;

^{1}H NMR ($CDCl_3$) δ 8.52 (1H, d, *J* = 0.8 Hz, $C^{2/6}$**H**), 8.47 (1H, d, *J* = 0.6 Hz, $C^{2/6}$**H**), 7.03-6.97 (6H, m, 6 x C^{Ar}**H**), 6.92-6.88 (2H, m, 2 x C^{Ar}), 2.17 (3H, s, C^{15}**H_3**);

^{13}C NMR ($CDCl_3$) δ 162.0 (1H, d, *J* = 247.8 Hz, $C^{10/14}$), 161.9 (d, *J* = 247.2 Hz, $C^{10/14}$), 150.1 ($C^{2/6}$), 148.2 ($C^{2/6}$), 146.8 (C), 135.4 (C), 133.7 (d, *J* = 3.7 Hz, $C^{7/11}$), 133.2 (d, *J* = 3.3 Hz, $C^{7/11}$), 131.2 (d, *J* = 33.4 Hz, 2 x C^{Ar}), 131.2 (d, *J* = 33.4 Hz, 2 x C^{Ar}), 115.4 (d, *J* = 21.6 Hz, 2 x C^{Ar}), 115.0 (d, *J* = 21.5 Hz, 2 x C^{Ar}), 17.6 (C^{15}), *the quaternary carbon for C^5 is not observed;*

^{19}F NMR ($CDCl_3$) δ –114.2, –115.1;

IR (neat): 1617, 1580, 1462, 1422, 1404, 1320, 1289, 1275, 1146, 1105, 1089, 1065 cm^{-1}.

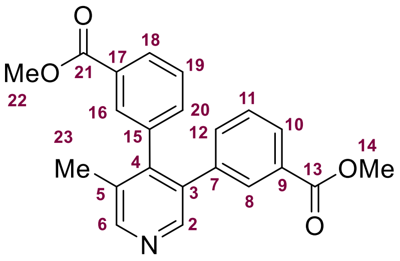

5-Methyl-3,4-(3-methoxycarbonylphenyl)pyridine (4s)

The title compound was prepared by **General Procedure G** using *pyridinium salt* **2s** (345 mg, 0.5 mmol) and was purified using FCC (0% - 5% EtOAc in CH_2Cl_2) and an acidic wash to give *pyridine* **4s** (94 mg, 52% yield) as a colourless oil.

HRMS (ESI): Exact mass calculated for $C_{22}H_{19}NO_4$ $[M+H]^+$ calc: 362.13868, found: 362.12812;

^{1}H NMR ($CDCl_3$) δ 8.56 (1H, s, C^2**H**), 8.52 (1H, s, C^6**H**), 7.93 (1H, dt, *J* = 7.8, 1.6 Hz, C^{18}**H**), 7.85 (1H, *J* = 7.7, 1.6 Hz, C^{10}**H**), 7.82 (1H, dd, *J* = 1.8, 1.8 Hz, C^{16}**H**), 7.79 (1H, dd, *J* = 1.6, 1.6 Hz, C^8**H**), 7.33 (1H, dd, *J* = 7.8, 7.8 Hz, C^{19}**H**), 7.22 (1H, dd, *J* = 7.7, 7.7 Hz, C^{11}**H**), 7.19–7.15 (2H, m, C^{12}**H** + C^{20}**H**), 3.88 (3H, s, C^{22}**H**$_3$), 3.86 (3H, s, C^{14}**H**$_3$), 2.17 (3H, s, C^{23}**H**$_3$);

^{13}C NMR ($CDCl_3$) δ 166.8 (C^{21}), 166.7 (C^{13}), 150.5 (C^2), 148.3 (C^6), 146.9, 138.0, 137.6, 135.4, 134.4, 133.9, 131.4, 131.0 (C^{16}), 130.5 (C^8), 130.4, 130.2, 128.9 (C^{18}), 128.7 (C^{19}), 128.5 (C^{10}), 128.2 (C^{11}), 52.4 (C^{22}), 52.3 (C^{14}), 17.8 (C^{23});

IR (neat): 2952, 1722, 1438, 1303, 1257, 1239, 1168, 1114, 781, 719 cm^{-1}.

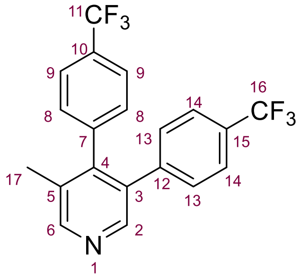

5-Methyl-3-4-bis(4-trifluoromethylphenyl)pyridine (4t)

The title compound was prepared by **General Procedure G** using *pyridinium salt* **2t** (355 mg, 0.5 mmol) and was purified using FCC (0% - 4% EtOAc in CH_2Cl_2) to give *pyridine* **4t** (109 mg, 57% yield) as a white solid.

m.p. (Et_2O): 115-117 °C;

HRMS (ESI): Exact mass calculated for $C_{20}H_{14}NF_6$ $[M+H]^+$ calc: 382.10250, found: 382.10242;

^{1}H NMR ($CDCl_3$) δ 8.59 (1H, s, C^{2}**H**), 8.51 (1H, s,C^{6}**H**), 7.58 (2H, d, *J* = 7.8 Hz, 2 x C^{9}**H**), 7.48 (2H, d, *J* = 8.1 Hz, 2 x C^{14}**H**), 7.20-7.15 (4H, m, 2 x C^{8}**H** + 2 x C^{13}**H**), 2.18 (3H, s, C^{17}**H$_3$**);

^{13}C NMR ($CDCl_3$) δ 150.7 (C^{2}), 148.2 (C^{6}), 146.2 (C), 141.1 (C), 140.7 (C), 134.7 (C), 131.2 (C), 130.0 (2 x C^{8}), 130.0 (q, *J* = 32.9 Hz, C^{10}), 129.7 (2 x C^{13}), 129.5 (q, *J* = 32.6 Hz,), 125.4 (q, *J* = 3.8 Hz, 2 x C^{9}), 125.1 (q, *J* = 3.9 Hz, 2 x C^{14}), 123.9 (q, *J* = 272.4 Hz, C^{11}), 123.8 (q, *J* = 272.1 Hz, C^{16}), 17.6 (C^{17});

^{19}F NMR ($CDCl_3$) δ –62.6;

IR (neat): 1618, 1400, 1324, 1154, 1108, 1066, 1030, 1017, 888, 840, 745, 689 cm^{-1}.

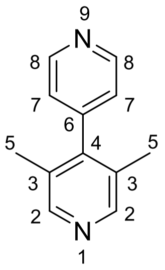

3,5-Dimethyl-4,4'-bipyridine (4u)

The title compound was prepared according to **General Procedure G** using *pyridinium* **2u** (249.8. mg, 0.5 mmol) with the addition of 1 equiv. of NaI. The crude material was purified by FCC (EtOAc:Pyridine - 100:0 to 95:5) and acid wash to give *pyridine* **4u** (63 mg, 68 % yield) as a white solid.

m.p.: 133-135 °C;

HRMS (ESI): Exact mass calculated for $C_{12}H_{13}N_2$ $[M+H]^+$ m/z: 185.10732, found: 185.10744;

^{1}H NMR (400 MHz, $CDCl_3$) δ 8.70 – 8.67 (2H, m, 2 x C^{8}**H**), 8.33 (2H, s, 2 x C^{2}**H**), 7.07 – 7.05 (2H, m, 2 x C^{7}**H**), 1.99 (6H, s, 2 x C^{5}**H$_3$**);

^{13}C NMR (101 MHz, $CDCl_3$) δ 150.4 (2 x C^{8}), 148.6 (2 x C^{2}), 146.4 (C), 146.3 (C), 129.9 (2 x C^{3}), 123.3 (2 x C^{7}), 17.2 (2 x C^{5});

IR (neat) (cm^{-1}): 1585, 1411, 1379, 987, 878, 838, 759, 670.

3,5-Dimethyl-4-(4-((2-phenyl)pyridyl)pyridine (4v)

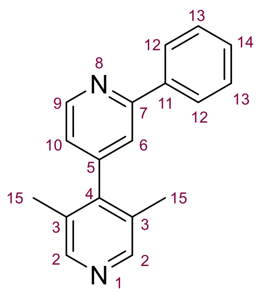

The title compound was prepared by **General Procedure G** using *pyridinium salt* **2v** (288 mg, 0.5 mmol) with the addition of 1 equiv. of NaI and was purified using FCC (0% - 20% EtOAc in CH_2Cl_2) and an acidic wash to give *pyridine* **4v** (70 mg, 54% yield) as a white solid.

m.p. (Et_2O): 132-134 °C;

HRMS (ESI): Exact mass calculated for $C_{18}H_{17}N_2$ $[M+H]^+$ calc: 261.1386 found: 261.1386;

^{1}H NMR ($CDCl_3$) δ 8.81 (1H, dd, *J* = 4.9, 0.9 Hz, C^9**H**), 8.41 (2H, s, 2 x C^2**H**), 8.05-8.02 (2H, m, 2 x C^{13}**H**), 7.54 (1H, dd, *J* = 1.6, 0.9 Hz, C^6**H**), 7.52-7.44 (3H, m, 2 x C^{12}**H** + C^{14}**H**), 7.04 (1H, dd, *J* = 4.9, 1.5 Hz, C^{10}**H**), 2.09 (6H, s, 2 x C^{15}**H**$_3$);

^{13}C NMR ($CDCl_3$) δ 158.1 (C), 150.3 (C^9), 148.7 (2 x C^2), 147.1 (C), 146.7 (C), 138.9 (C), 129.9 (2 x C^3), 129.3 (C^{14}), 128.8 (2 x C^{12}), 126.9 (2 x C^{13}), 121.5 (C^{10}), 119.9 (C^6), 17.2 (2 x C^{15});

IR (neat): 1606, 1584, 1539, 1469, 1443, 1382, 1282, 1162, 1025, 885, 847, 776 cm^{-1}.

3,5-Dimethyl-4-(4-(2-pyrimidine)pyridine (4w)

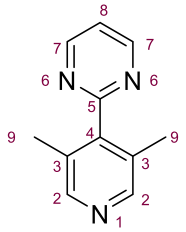

The title compound was prepared by **General Procedure G** using *pyridinium salt* **2w** (125 mg, 0.25 mmol) with the addition of 1 equiv. of NaI and was purified using FCC (20% - 60% EtOAc in CH_2Cl_2) to give *pyridine* **4w** (22 mg, 48% yield) as a white solid.

m.p. (Et_2O): 90-92 °C;

HRMS (ESI): Exact mass calculated for $C_{11}H_{12}N_3$ $[M+H]^+$ calc: 186.10257 found: 186.10270;

^{1}H NMR ($CDCl_3$) δ 8.92 (2H, d, *J* = 4.9 Hz, 2 x C^7**H**), 8.43 (2H, s, 2 x C^2**H**), 7.36 (1H, t, *J* = 4.9 Hz, C^8**H**), 2.13 (6H, s, 2 x C^9**H**$_3$);

^{13}C NMR ($CDCl_3$) δ 165.7 (C), 157.4 (2 x C^7), 148.1 (2 x C^2), 146.5 (C), 130.6 (C), 119.7 (C^8), 16.6 (2 x C^9);

IR (neat): 1563, 1514, 1409, 1261, 1163, 1090, 992, 873, 818, 803, 753, 700 cm^{-1}.

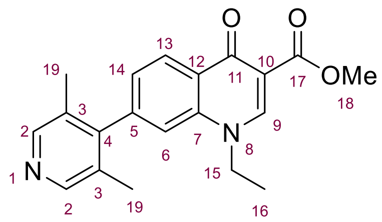

***N*-Methyl-7-(4-(3,3-dimethylpyridine)-4-oxoquinoline-3-carboxylic acid ethyl ester (4x)**

The title compound was prepared by **General Procedure G** using *pyridinium salt* **2x** (166.25 mg, 0.25 mmol) and was purified using FCC (10% EtOAc in CH_2Cl_2 then 2-4% MeOH in CH_2Cl_2) to give *pyridine* **4x** (35 mg, 42% yield) as a white solid.

m.p. (Et_2O): 115-117 °C;

HRMS (ESI): Exact mass calculated for $C_{20}H_{21}O_3N_2$ $[M+H]^+$ calc: 337.15467, found: 337.15469;

^{1}H NMR ($CDCl_3$) δ 8.65 (1H, d, *J* = 8.5 Hz, C^{13}**H**), 8.59 (1H, s, C^9**H**), 8.41 (2H, s, 2 x C^2**H**), 7.24-7.20 (2H, m, C^6**H** + C^{14}**H**), 4.26 (2H, q, *J* = 7.2 Hz, C^{15}**H**$_2$), 3.95 (3H, s, C^{18}**H**$_3$), 2.05 (6H, s, 2 x C^{19}**H**$_3$), 1.54 (3H, t, *J* = 7.2 Hz, C^{16}**H**$_3$);

^{13}C NMR ($CDCl_3$) δ 173.9 (C^{17}), 166.4 (C^{11}), 149.1 (C^9), 148.5 (2 x C^2), 147.9 (C), 142.8 (C)¸138.9 (C), 130.4 (2 x C^3) 129.1 (C^{13}), 128.6 (C), 125.1 ($C^{6/14}$), 115.0 ($C^{6/14}$), 111.2 (C)¸ 52.2 (C^{18}), 48.9 (C^{15}), 17.3 (2 x C^{19}), 14.6 (C^{16});

IR (neat): 2952, 2162, 1723, 1610, 1537, 1464, 1379, 1312, 1258, 1211, 1160, 1130 cm^{-1}.

Unproductive Substrates

A) The following C-4 unsubstituted pyridinium substrate led to a very complex reaction mixture with no methylated product observable. No other structure could be isolated and characterised from the reaction mixture.

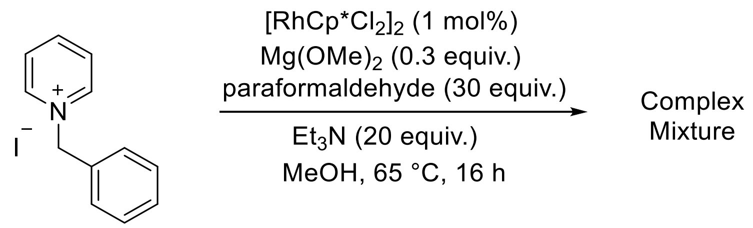

B) The following structural motifs with substitution at C-4 were found to be unstable to the basic methanol-methoxide-paraformaldehyde reaction conditions. Each case led to the formation of complex mixtures and these C-4 substituents are incompatible with the reaction.

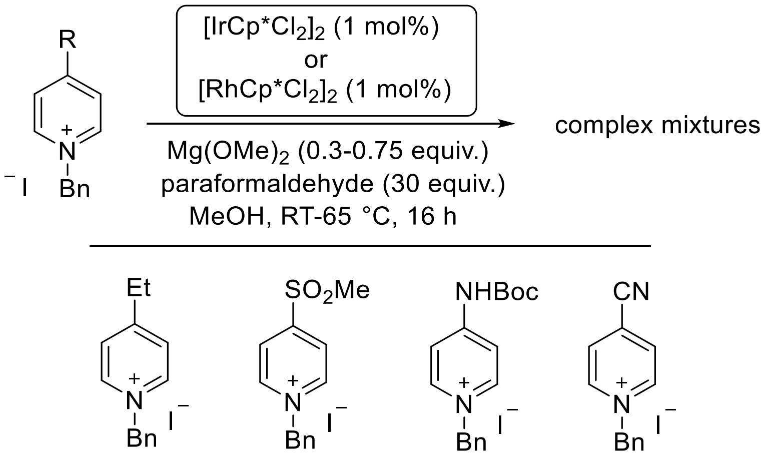

NMR Spectra

Pyridine Precursors

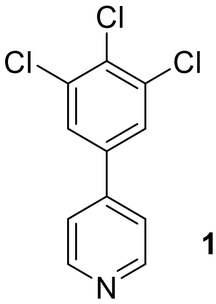

1j

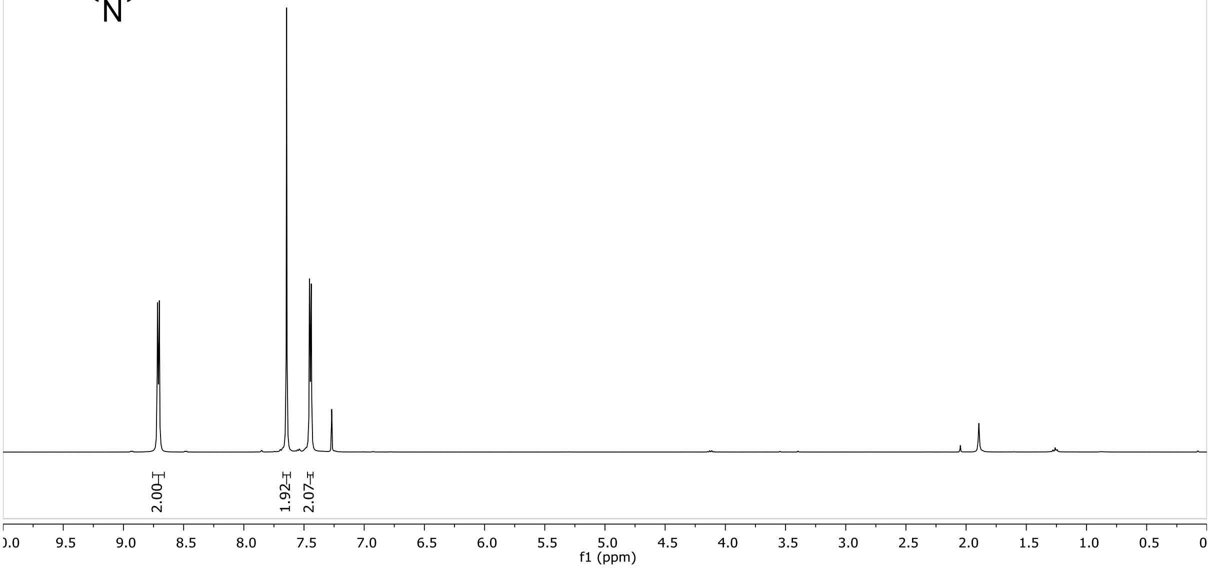

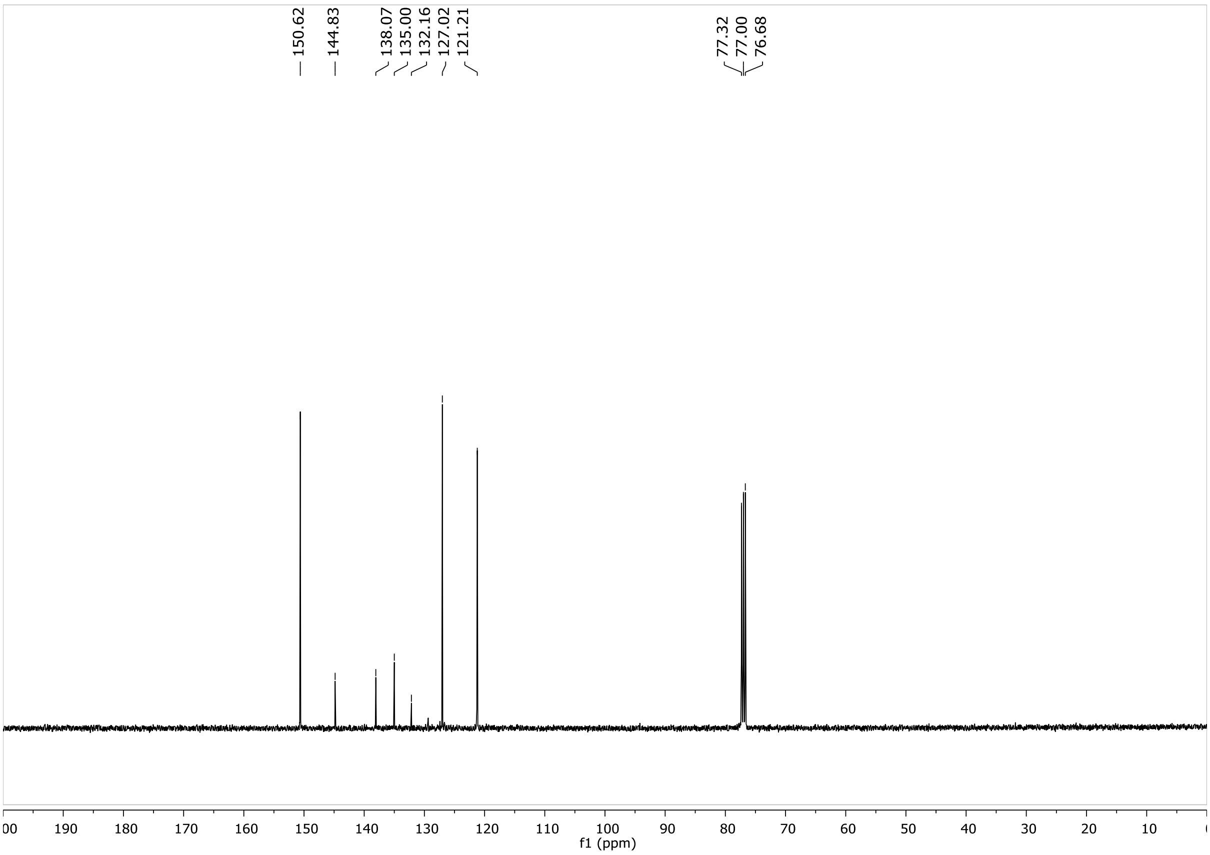

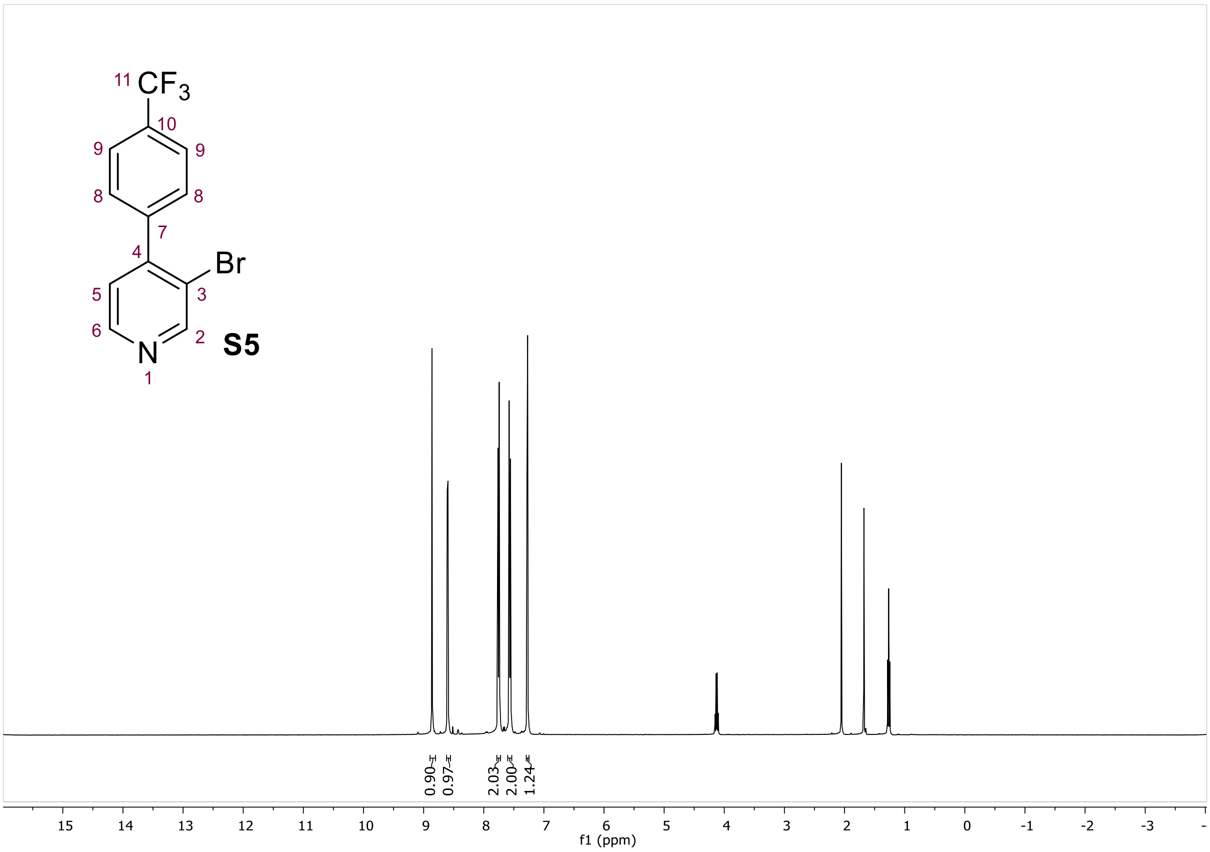
11 CF3
10
9
9
8
8
7
4
Br
3
5
6
2
N
1
S5
0.90
0.97
2.03
2.00
1.24
15 14 13 12 11 10 9 8 7 6 5 4 3 2 1 0 -1 -2 -3
f1 (ppm)

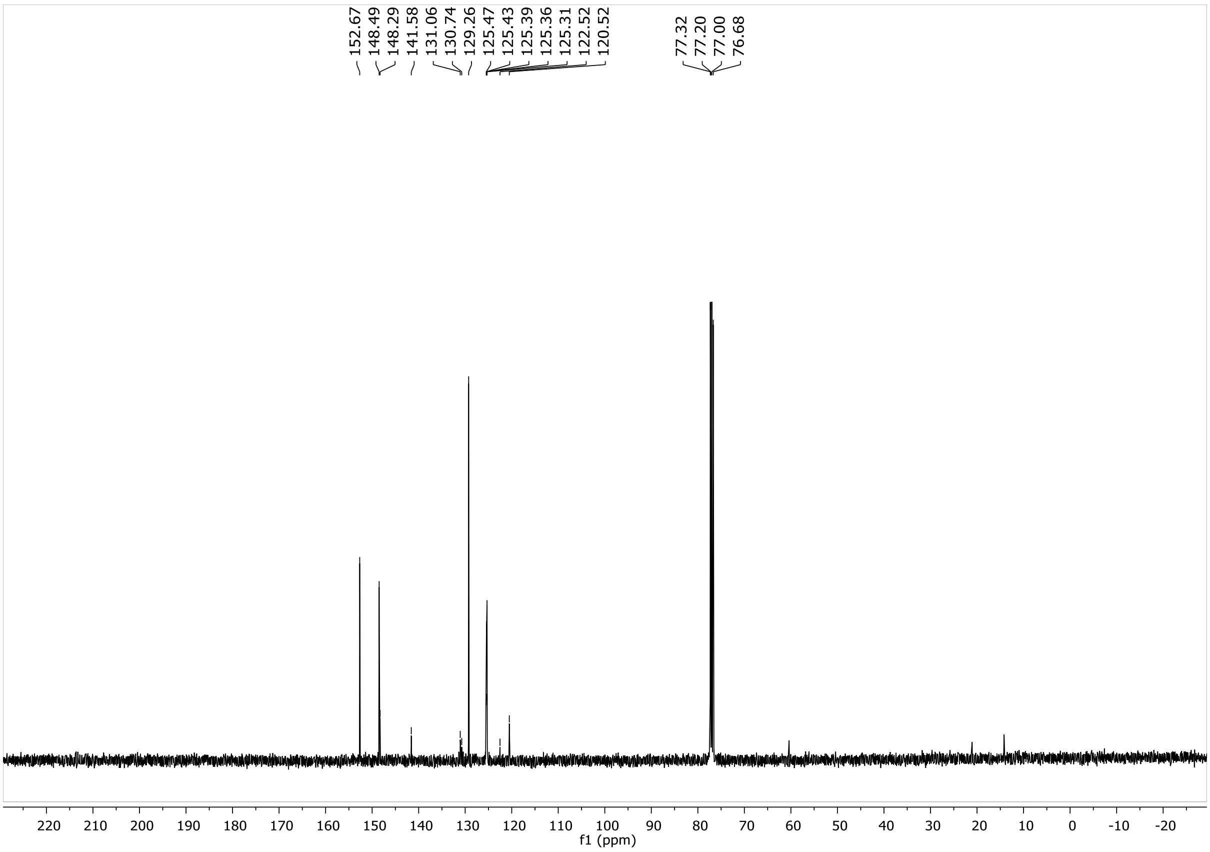
152.67
148.49
148.29
141.58
131.06
130.74
129.26
125.47
125.43
125.39
125.36
125.31
122.52
120.52
77.32
77.20
77.00
76.68
220 210 200 190 180 170 160 150 140 130 120 110 100 90 80 70 60 50 40 30 20 10 0 -10 -20
f1 (ppm)

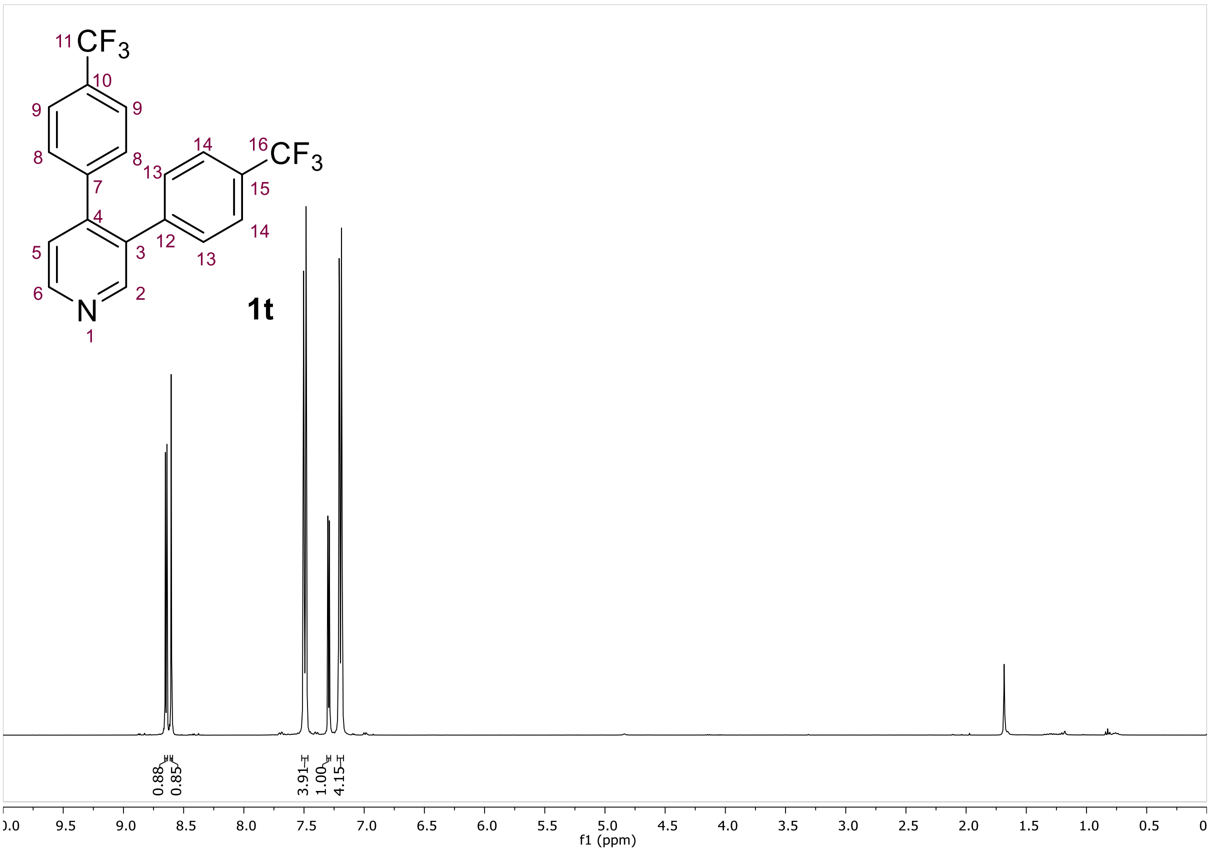
11 CF3
10
9
9
8
8
7
4
14
16 CF3
13
15
12
14
13
5
3
6
N
2
1
1t
0.88
0.85
3.91
1.00
4.15
f1 (ppm)

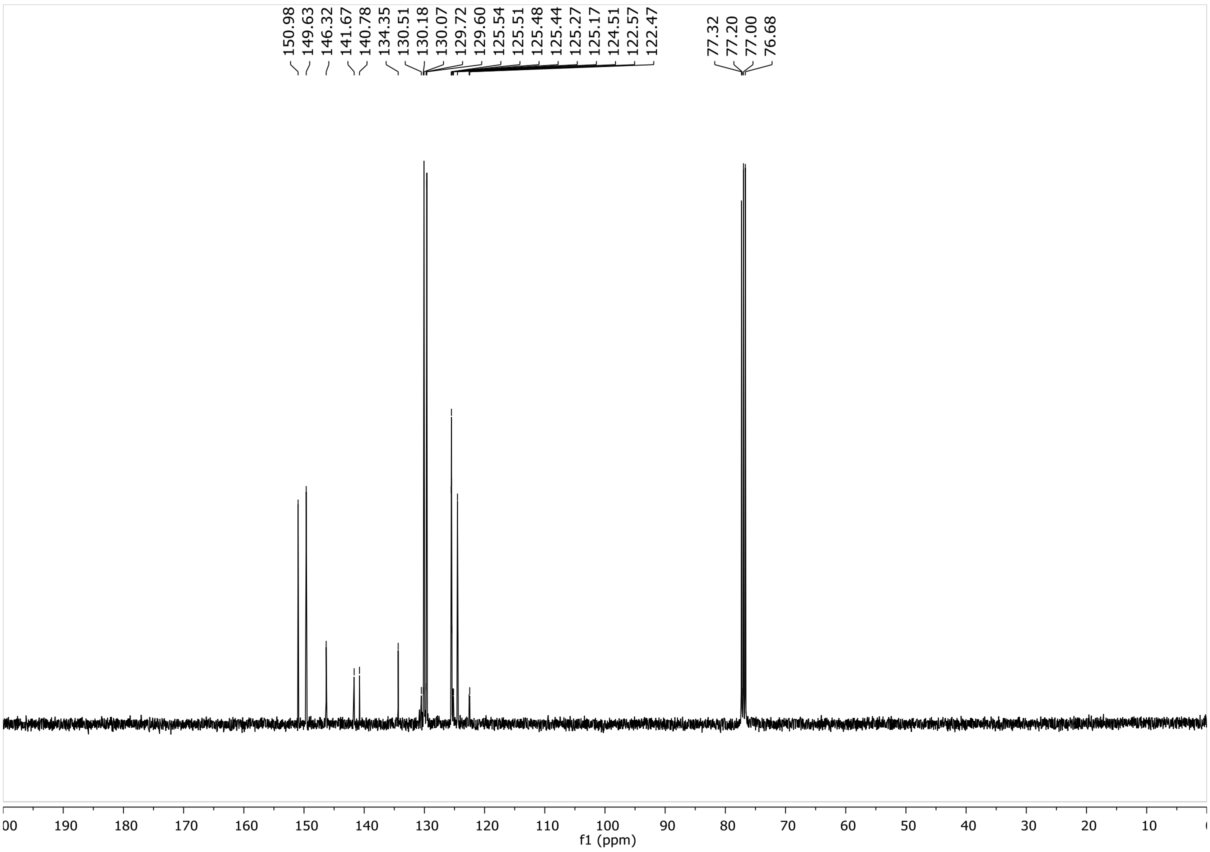
150.98
149.63
146.32
141.67
140.78
134.35
130.51
130.18
130.07
129.72
129.60
125.54
125.51
125.48
125.44
125.27
125.17
124.51
122.57
122.47
77.32
77.20
77.00
76.68
f1 (ppm)

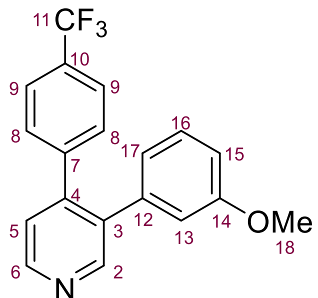

CF3
OMe
N

1p

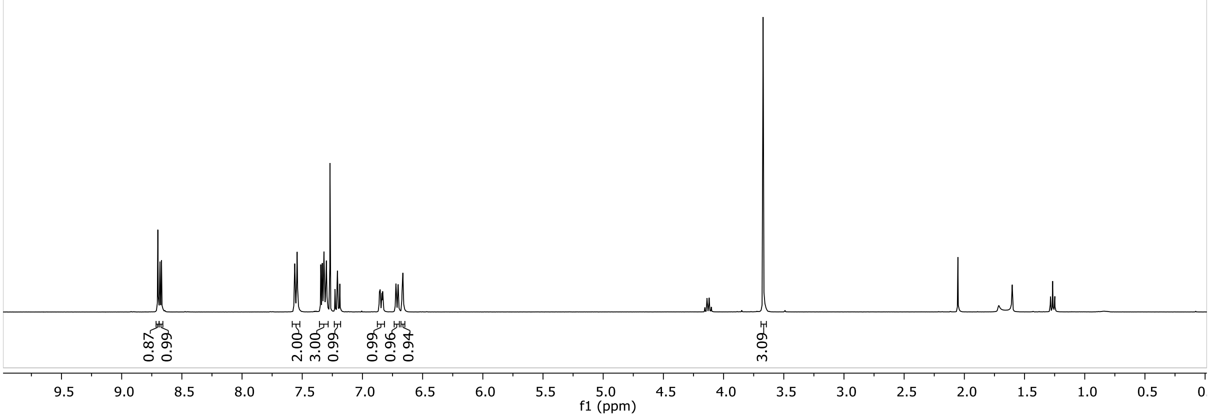

0.87
0.99
2.00
3.00
0.99
0.99
0.96
0.94
3.09
f1 (ppm)

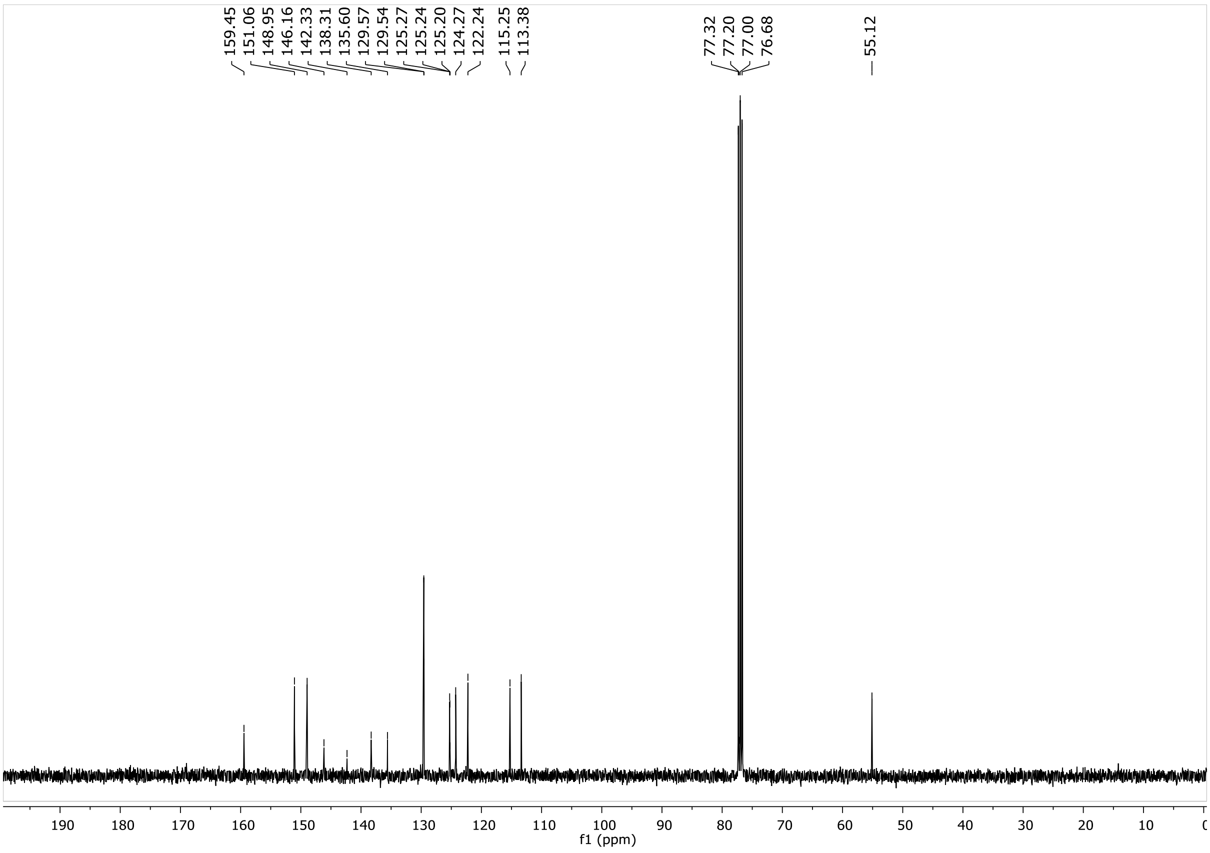

159.45
151.06
148.95
146.16
142.33
138.31
135.60
129.57
129.54
125.27
125.24
125.20
124.27
122.24
115.25
113.38
77.32
77.20
77.00
76.68
55.12
f1 (ppm)

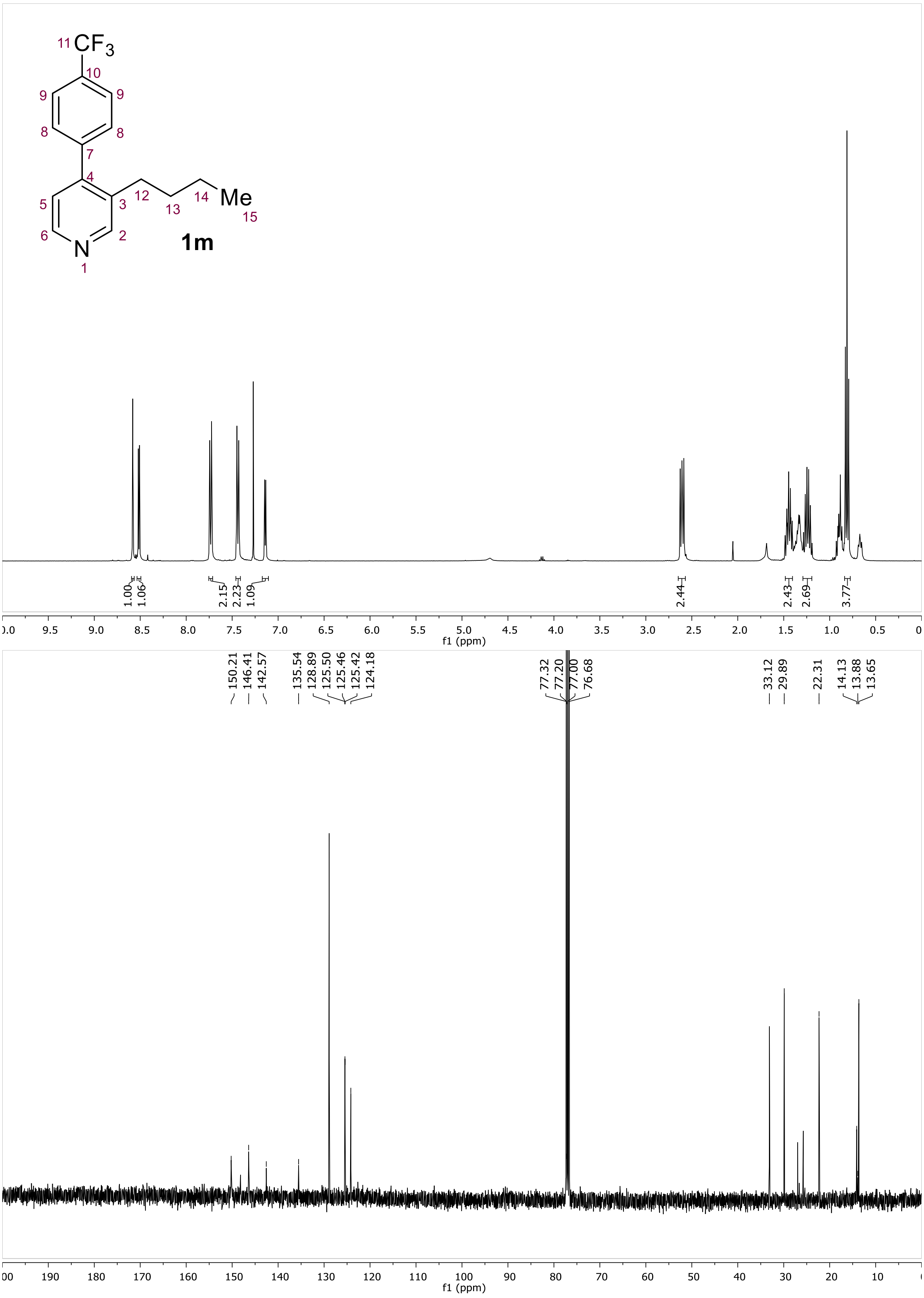

1m
1.00
1.06
2.15
2.23
1.09
2.44
2.43
2.69
3.77
f1 (ppm)
150.21
146.41
142.57
135.54
128.89
125.50
125.46
125.42
124.18
77.32
77.20
77.00
76.68
33.12
29.89
22.31
14.13
13.88
13.65
f1 (ppm)

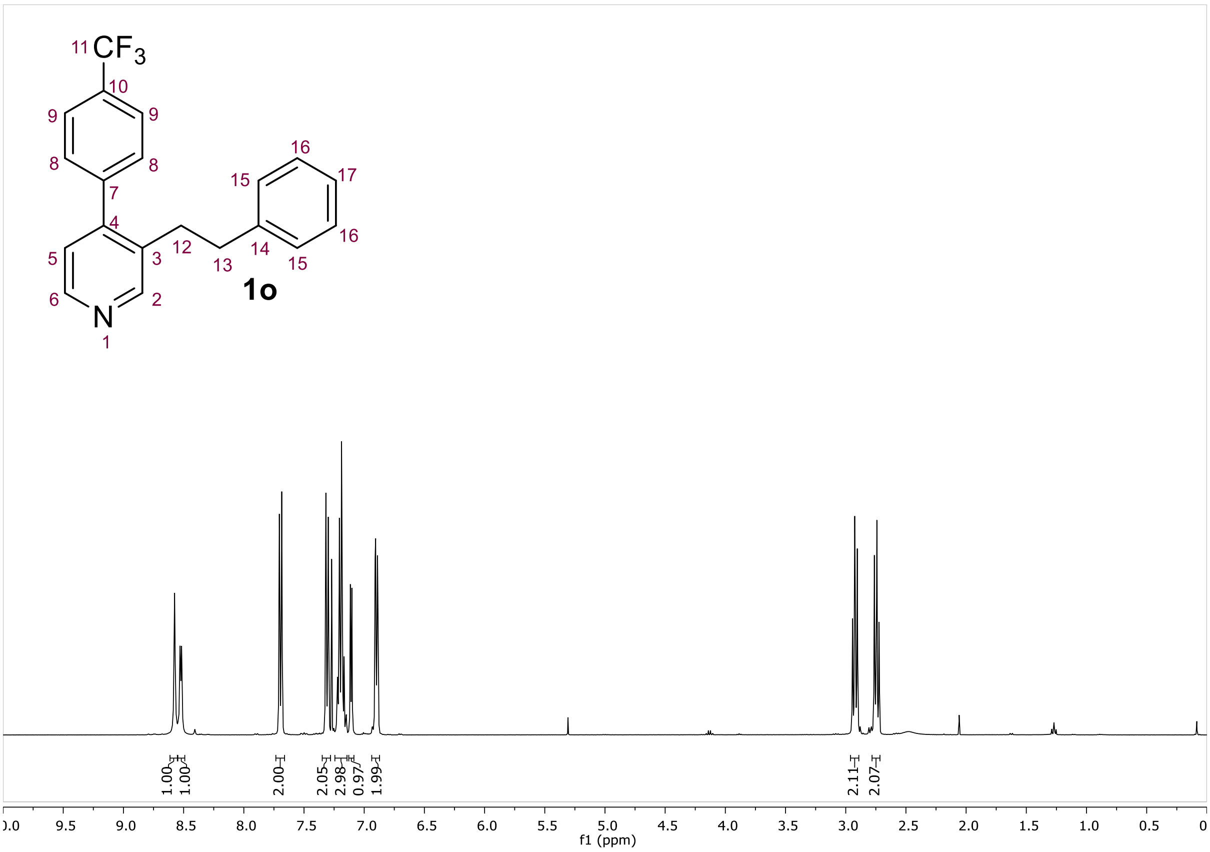
CF3
1o
1.00
1.00
2.00
2.05
2.98
0.97
1.99
2.11
2.07
f1 (ppm)

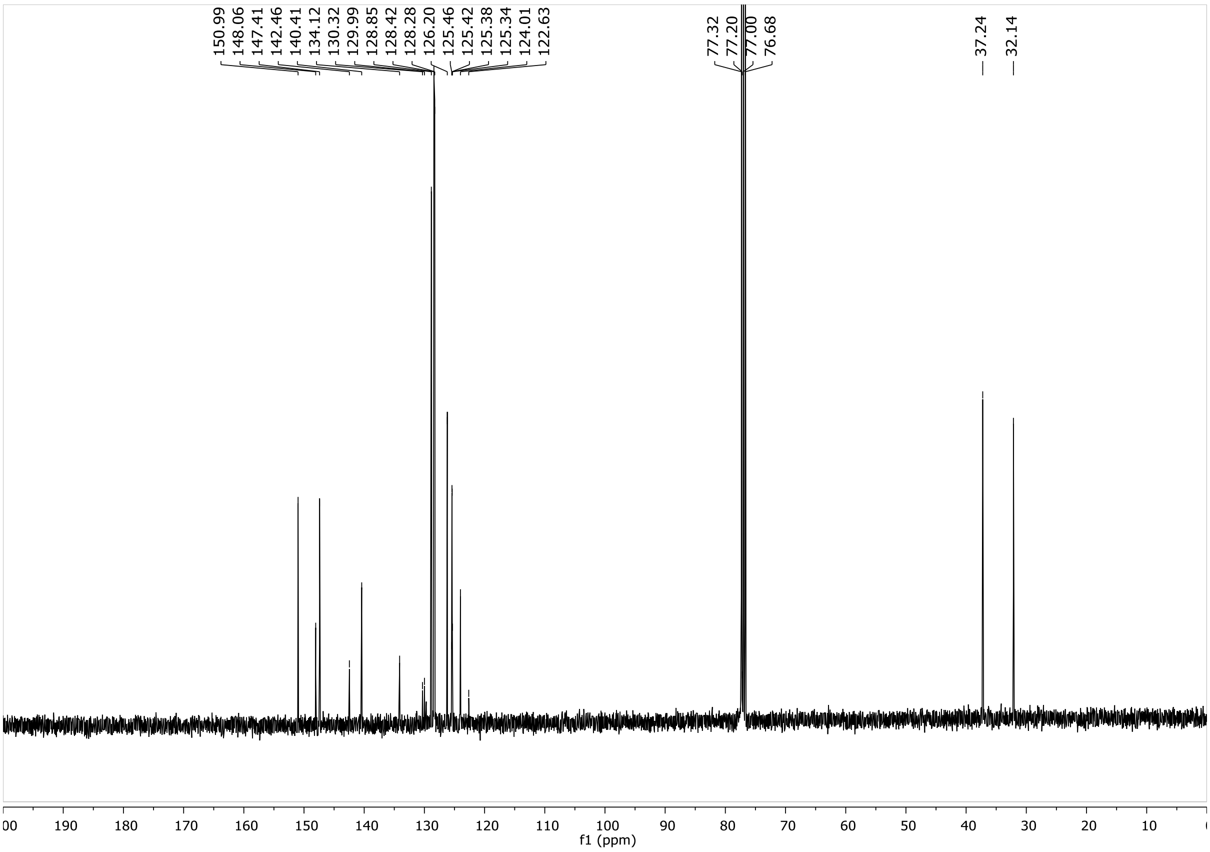
150.99
148.06
147.41
142.46
140.41
134.12
130.32
129.99
128.85
128.42
128.28
126.20
125.46
125.42
125.38
125.34
124.01
122.63
77.32
77.20
77.00
76.68
37.24
32.14
f1 (ppm)

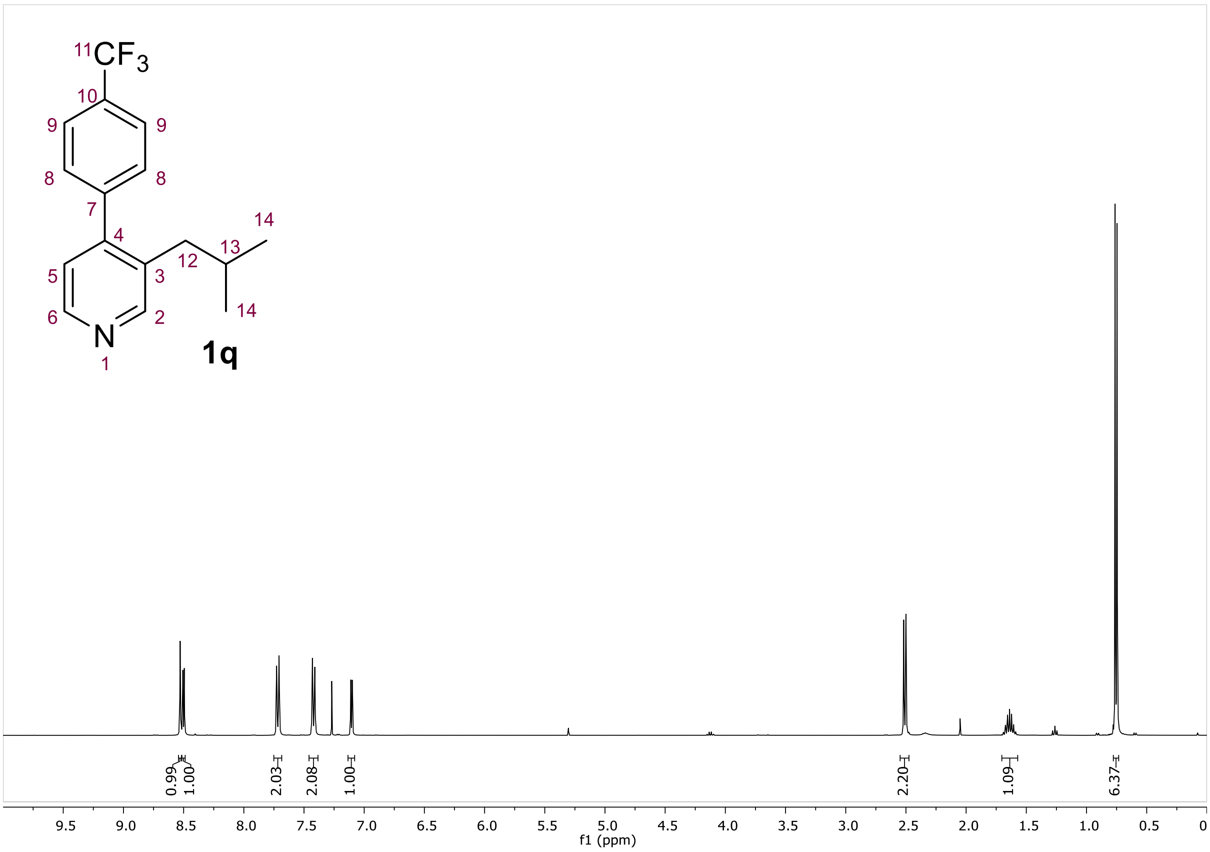
11CF3
10
9
9
8
8
7
4
14
13
12
3
5
6
2
14
N
1
1q
0.99
1.00
2.03
2.08
1.00
2.20
1.09
6.37
9.5 9.0 8.5 8.0 7.5 7.0 6.5 6.0 5.5 5.0 4.5 4.0 3.5 3.0 2.5 2.0 1.5 1.0 0.5 0
f1 (ppm)

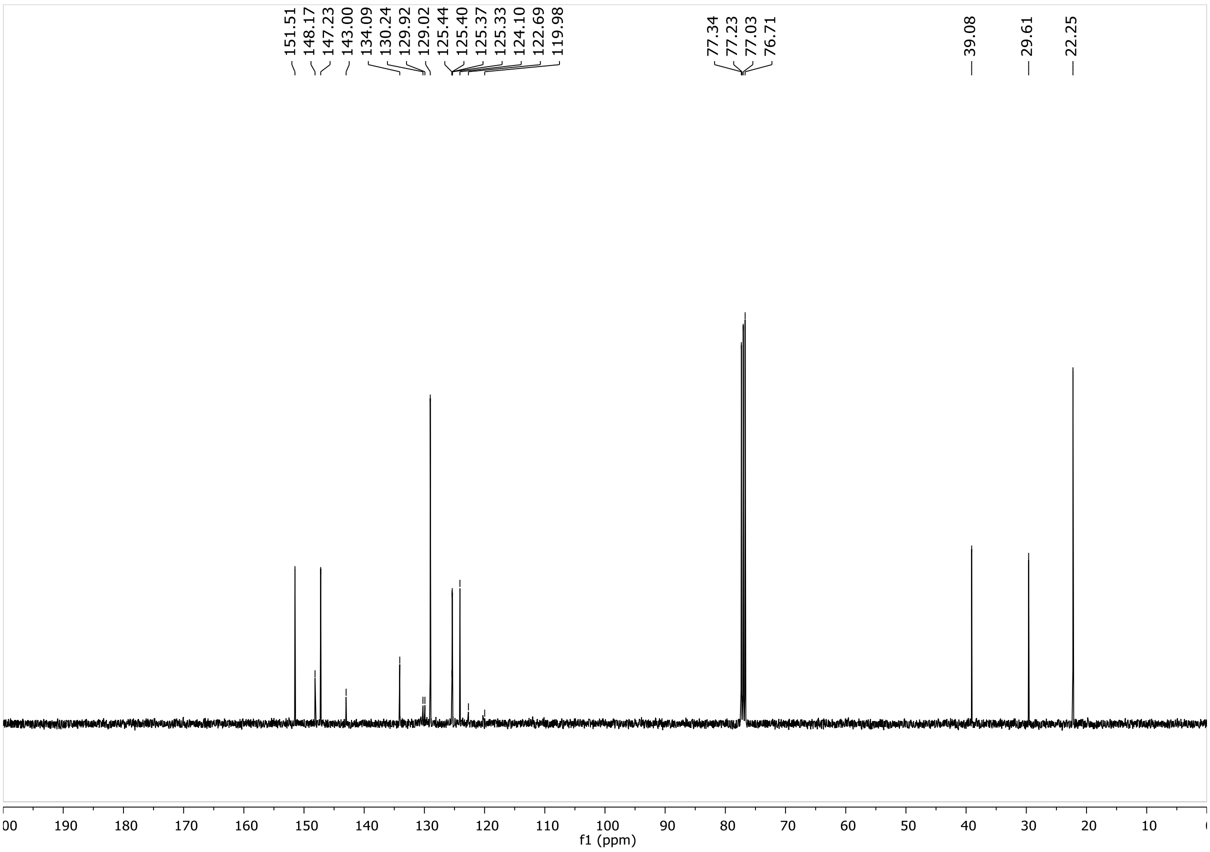
151.51
148.17
147.23
143.00
134.09
130.24
129.92
129.02
125.44
125.40
125.37
125.33
124.10
122.69
119.98
77.34
77.23
77.03
76.71
39.08
29.61
22.25
190 180 170 160 150 140 130 120 110 100 90 80 70 60 50 40 30 20 10
f1 (ppm)

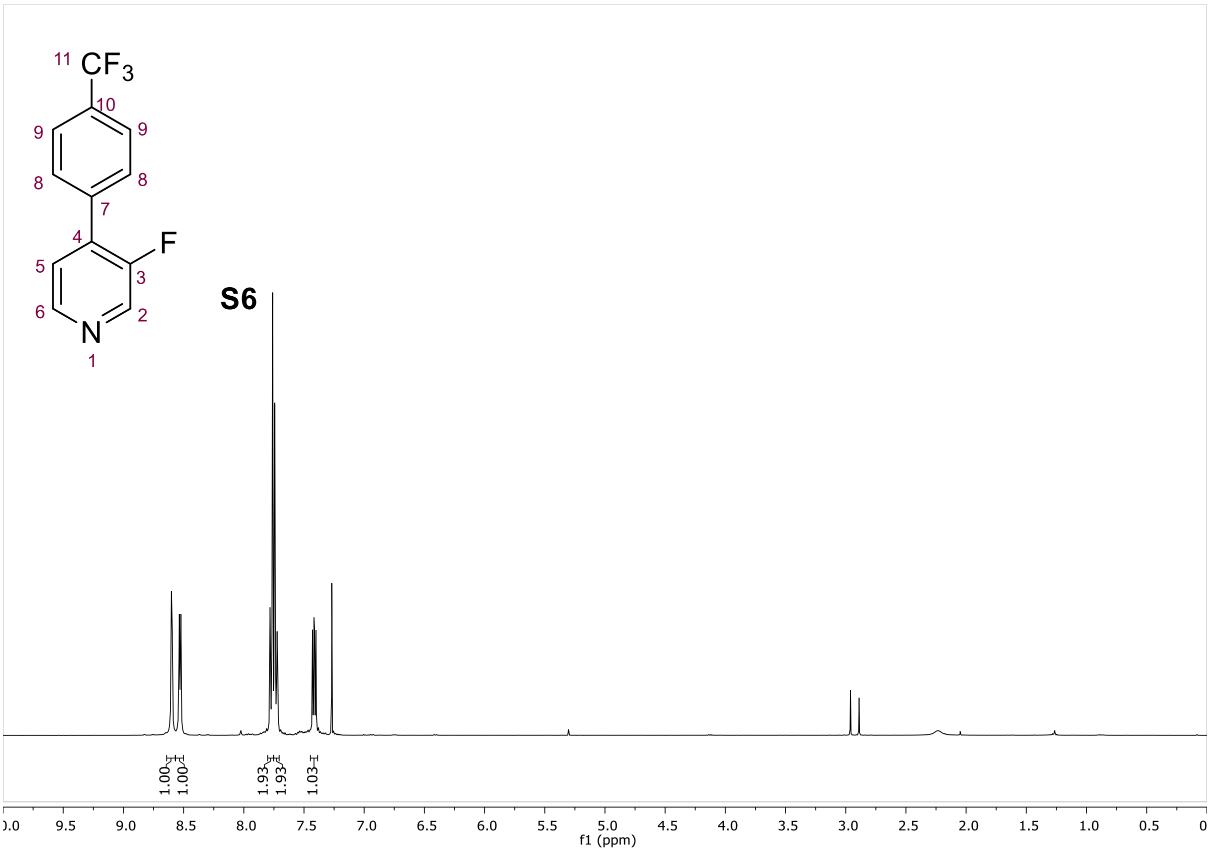

S6
CF3
F
N
1.00
1.00
1.93
1.93
1.03
f1 (ppm)

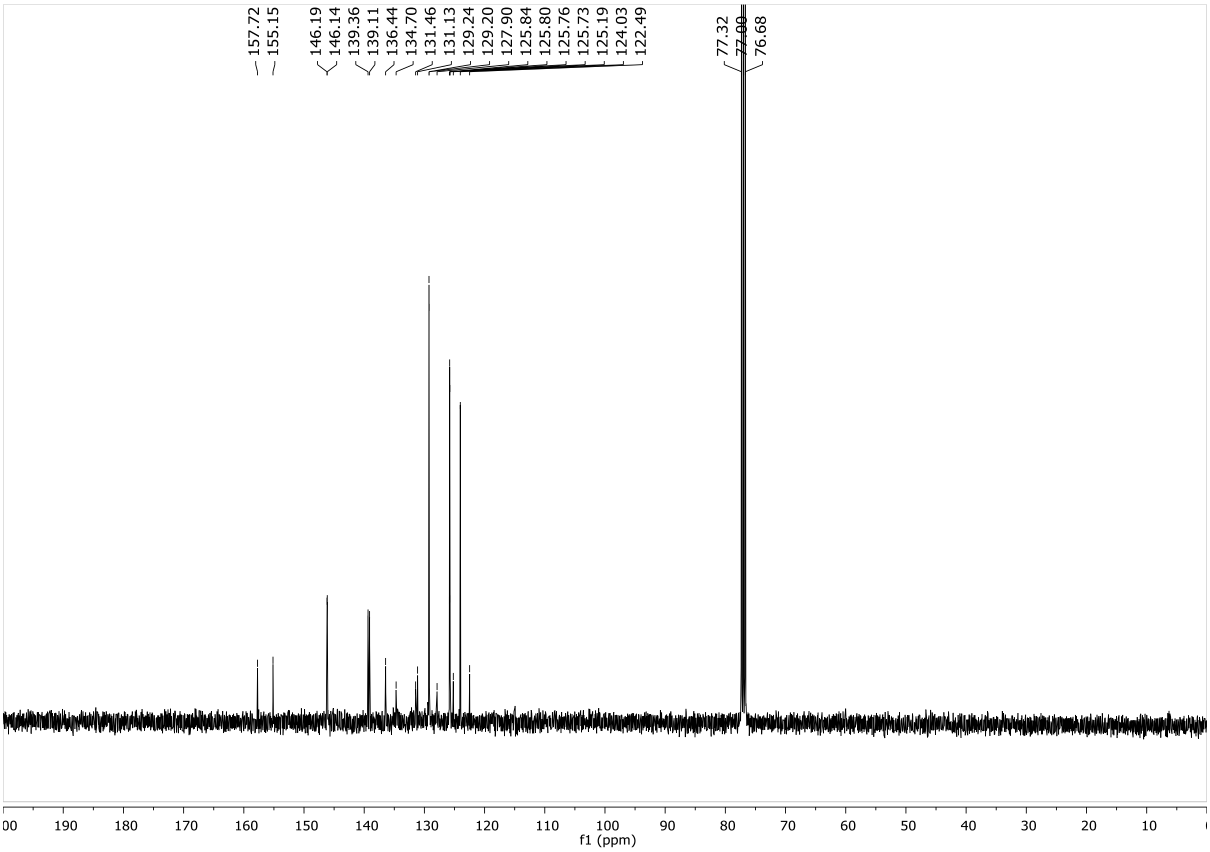

157.72
155.15
146.19
146.14
139.36
139.11
136.44
134.70
131.46
131.13
129.24
129.20
127.90
125.84
125.80
125.76
125.73
125.19
124.03
122.49
77.32
77.00
76.68
f1 (ppm)

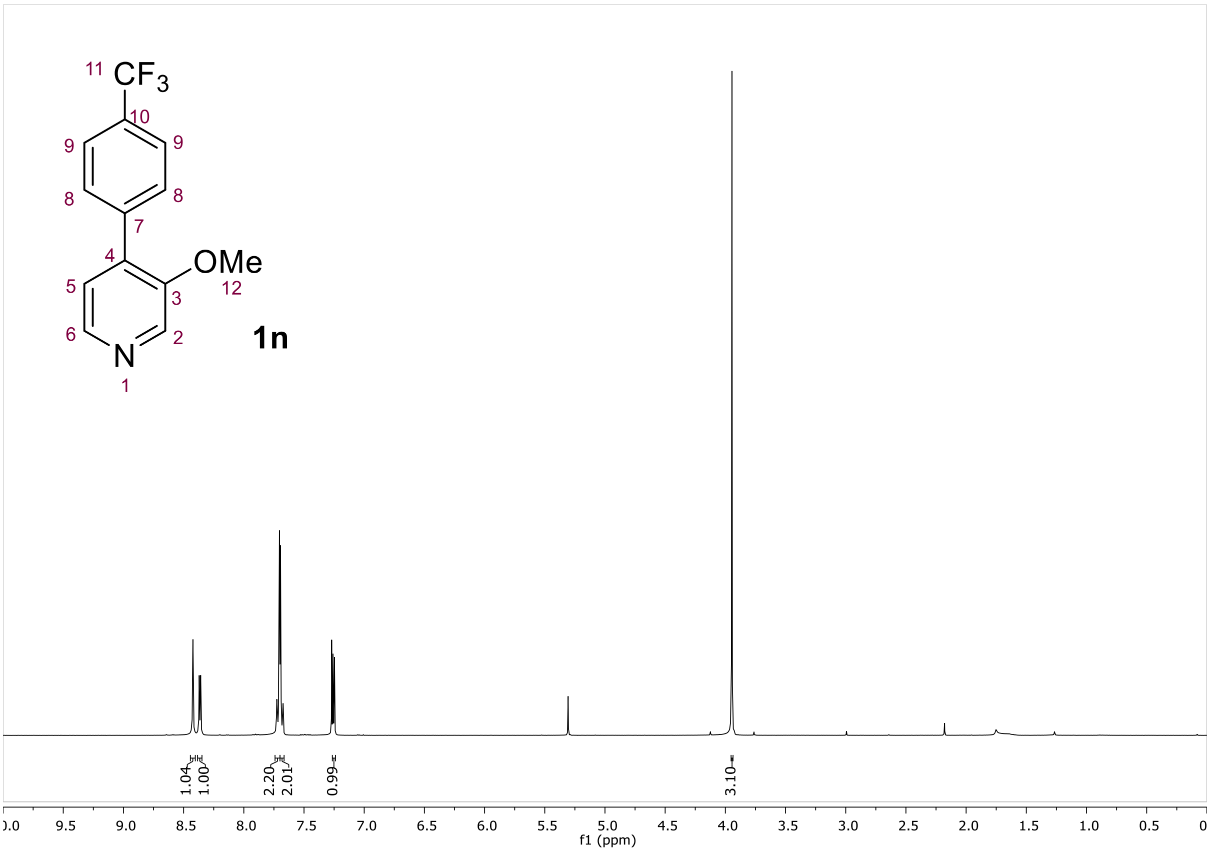
CF3
OMe
1n
1.04
1.00
2.20
2.01
0.99
3.10
f1 (ppm)

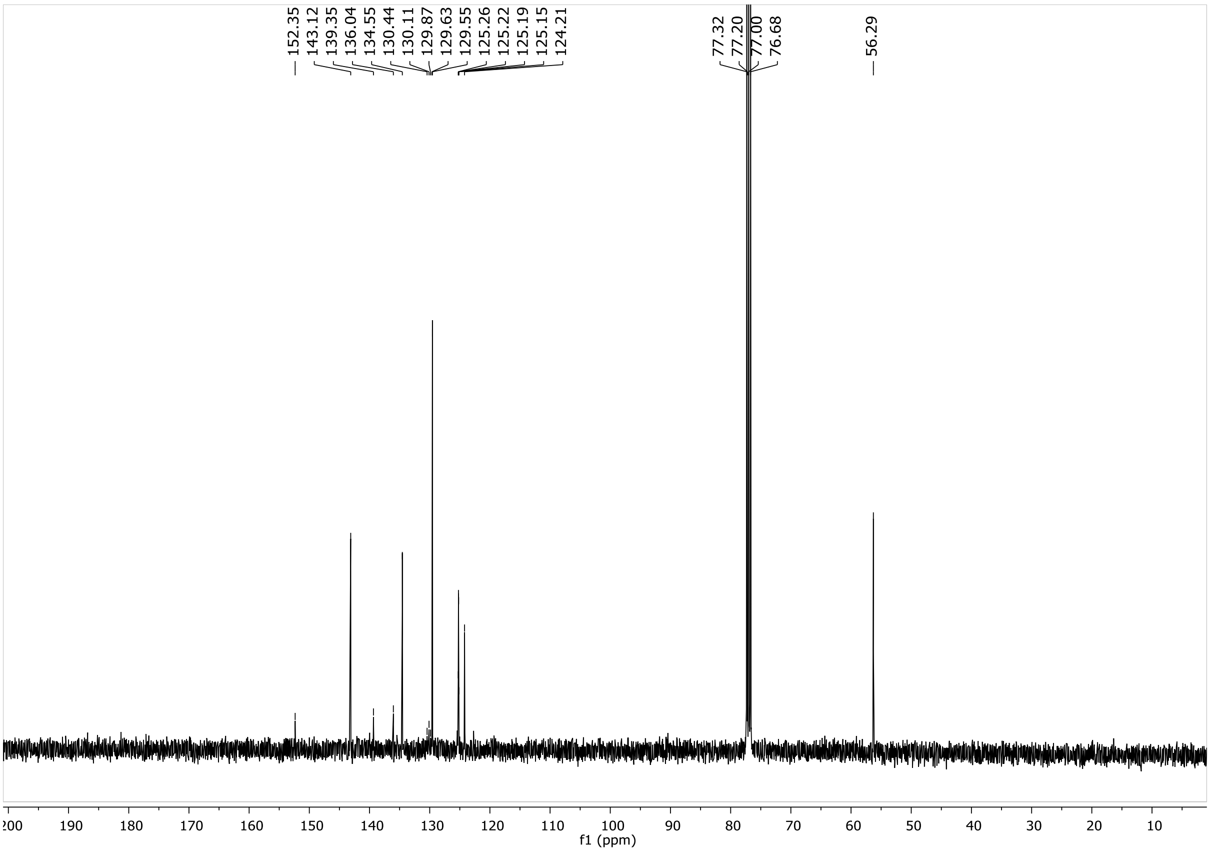
152.35
143.12
139.35
136.04
134.55
130.44
130.11
129.87
129.63
129.55
125.26
125.22
125.19
125.15
124.21
77.32
77.20
77.00
76.68
56.29
f1 (ppm)

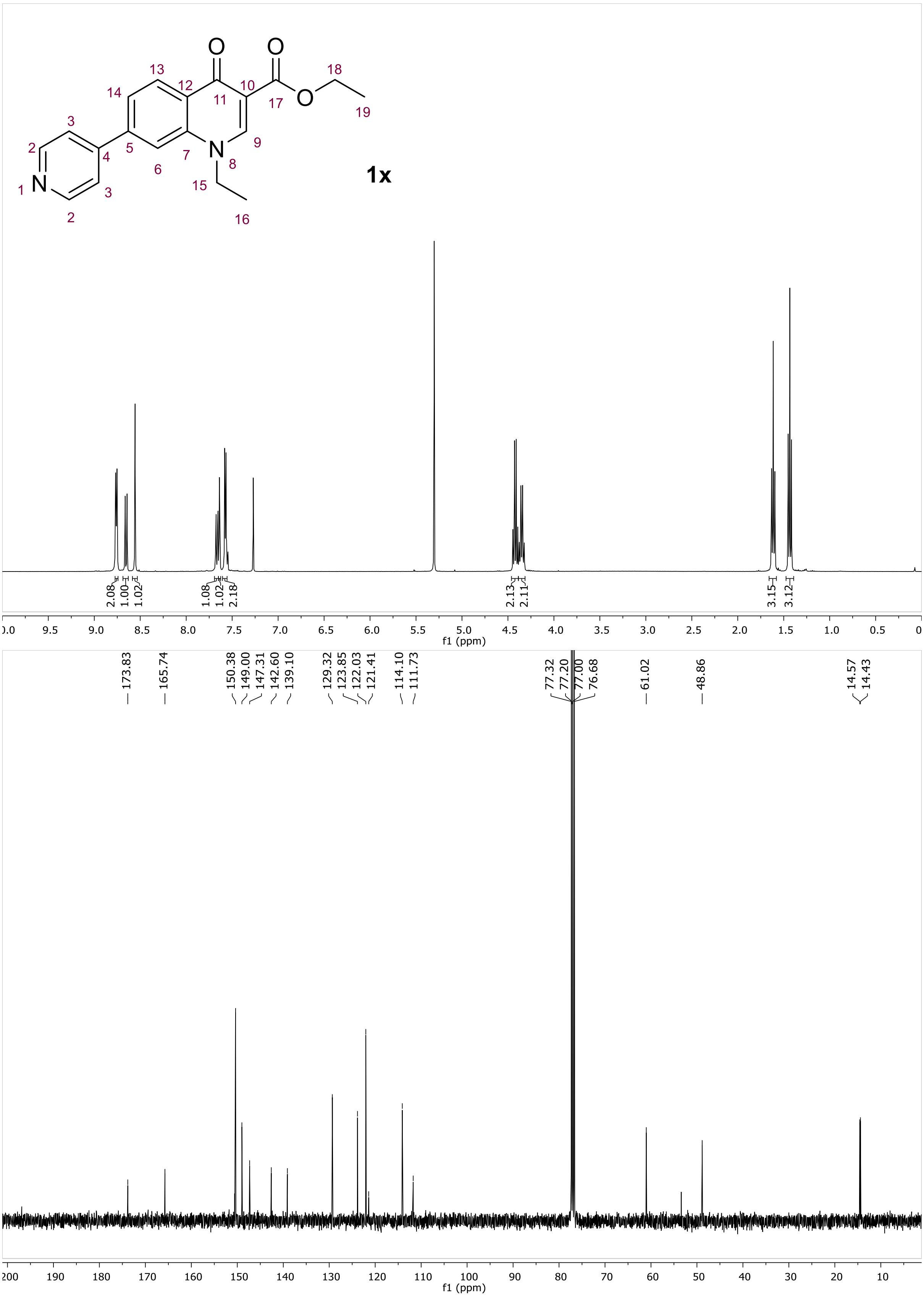
1x
f1 (ppm)
f1 (ppm)

Starting Materials

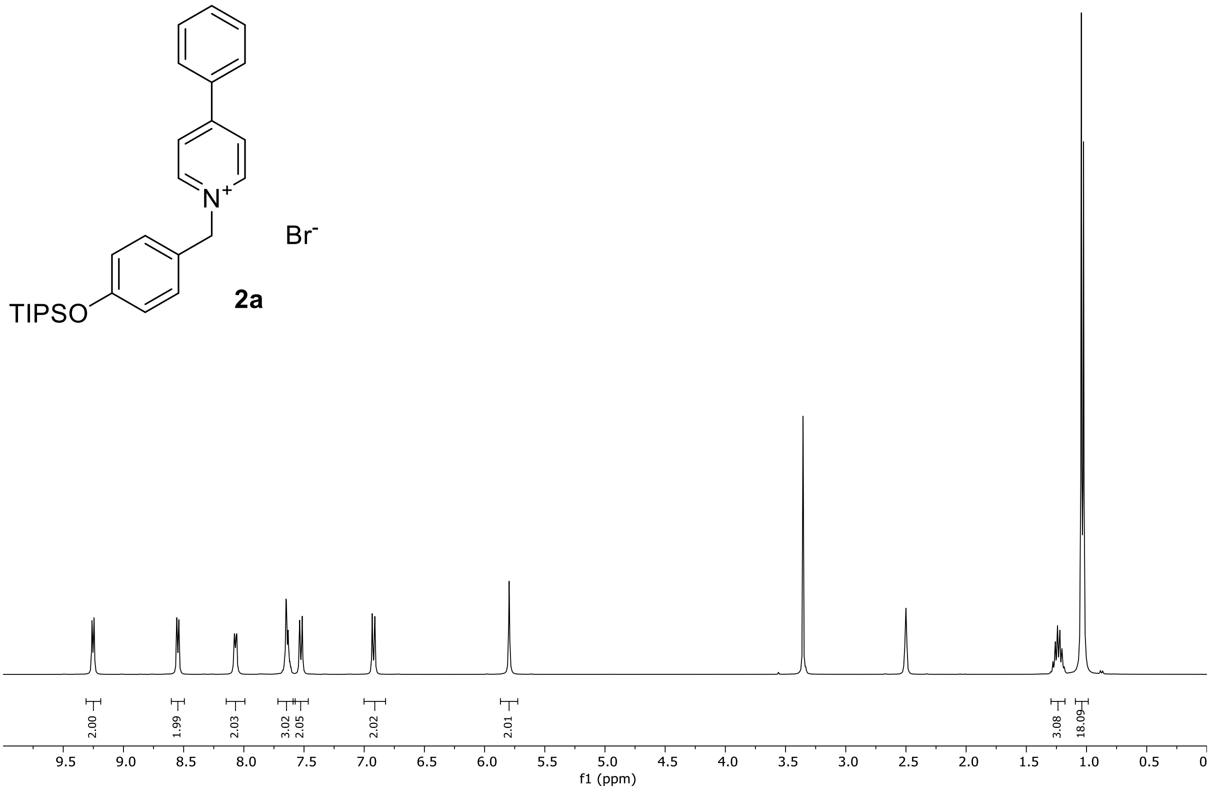

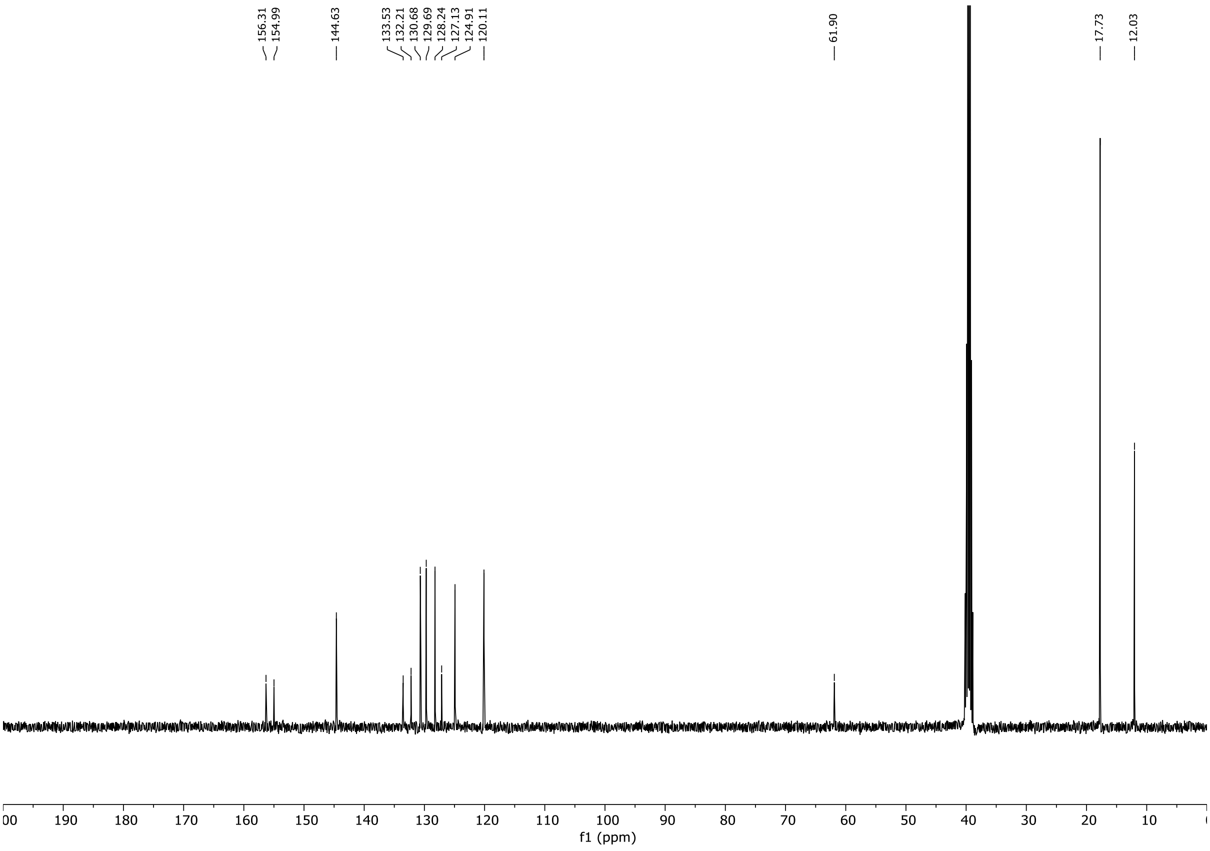

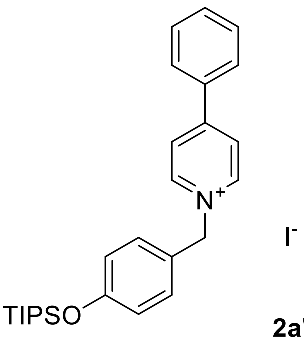
TIPSO
N+
I-

2a'

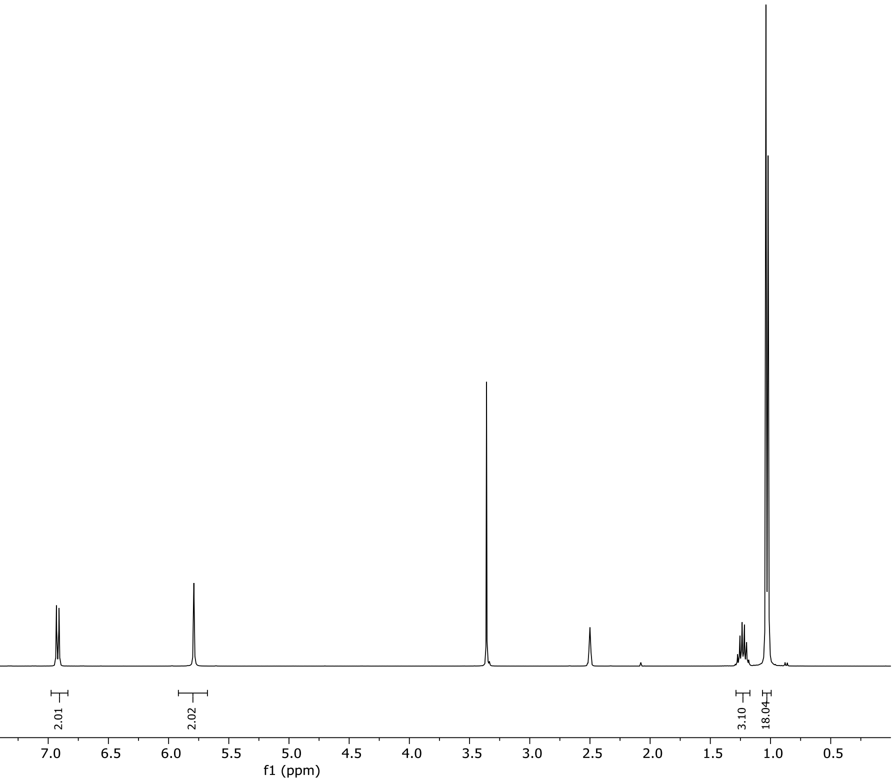
2.00
1.99
2.02
2.99
2.03
2.01
2.02
3.10
18.04
9.5 9.0 8.5 8.0 7.5 7.0 6.5 6.0 5.5 5.0 4.5 4.0 3.5 3.0 2.5 2.0 1.5 1.0 0.5
f1 (ppm)

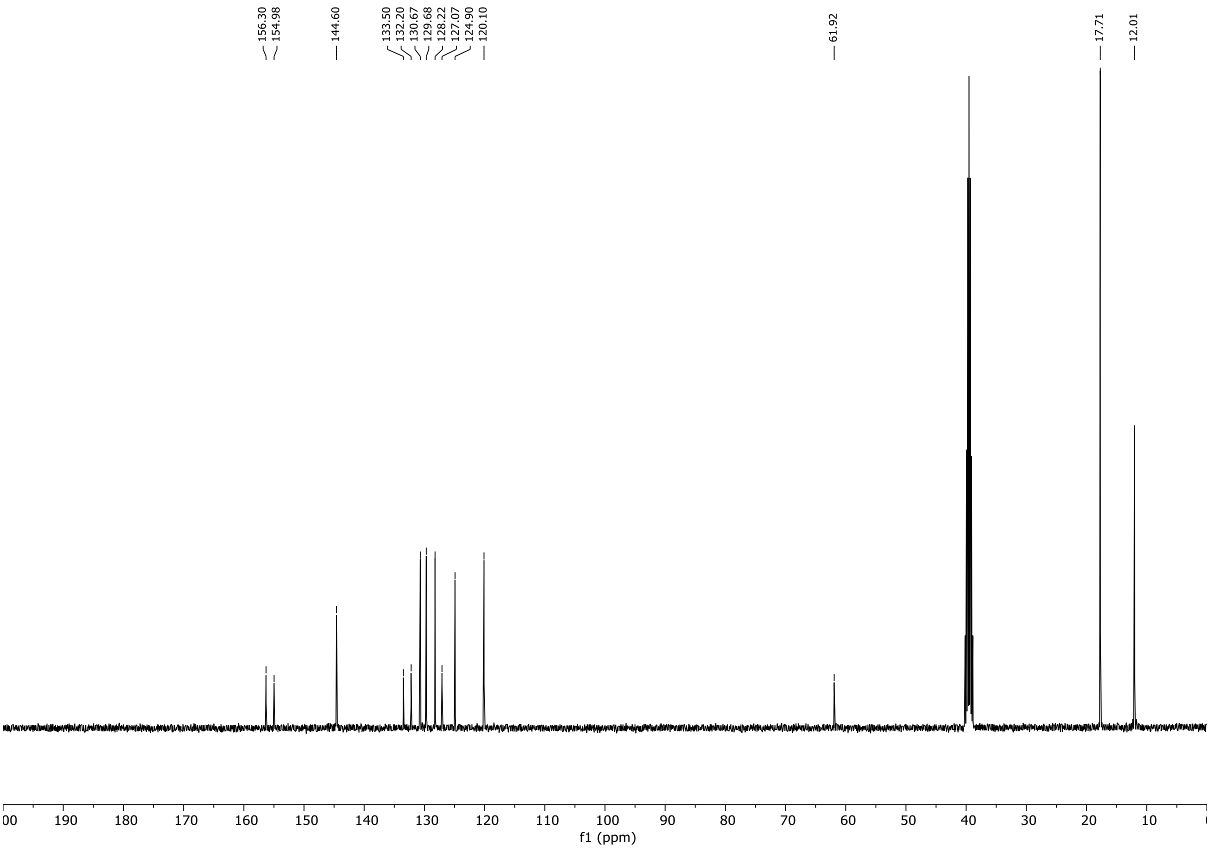
156.30
154.98
144.60
133.50
132.20
130.67
129.68
128.22
127.07
124.90
120.10
61.92
17.71
12.01
00 190 180 170 160 150 140 130 120 110 100 90 80 70 60 50 40 30 20 10
f1 (ppm)

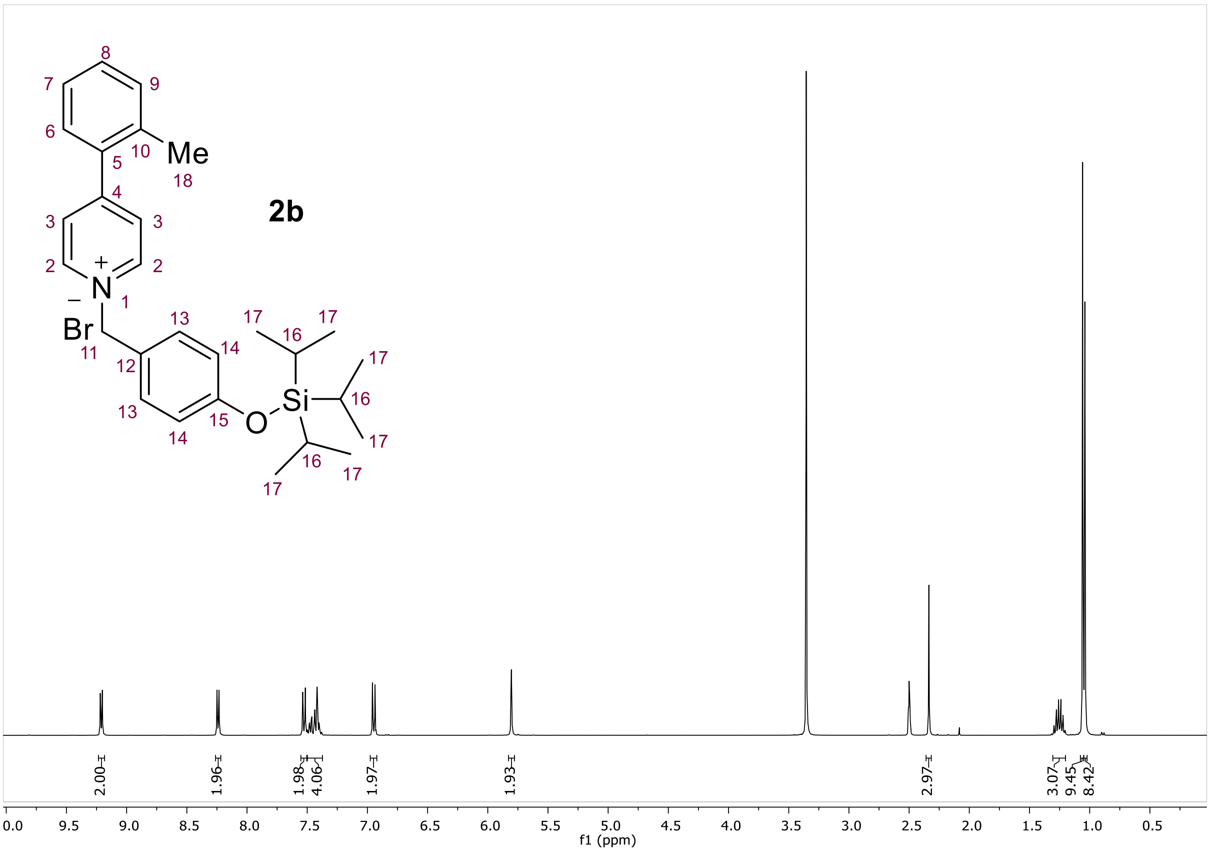
2b
Me
Br
N
O
Si
2.00
1.96
1.98
4.06
1.97
1.93
2.97
3.07
9.45
8.42
f1 (ppm)

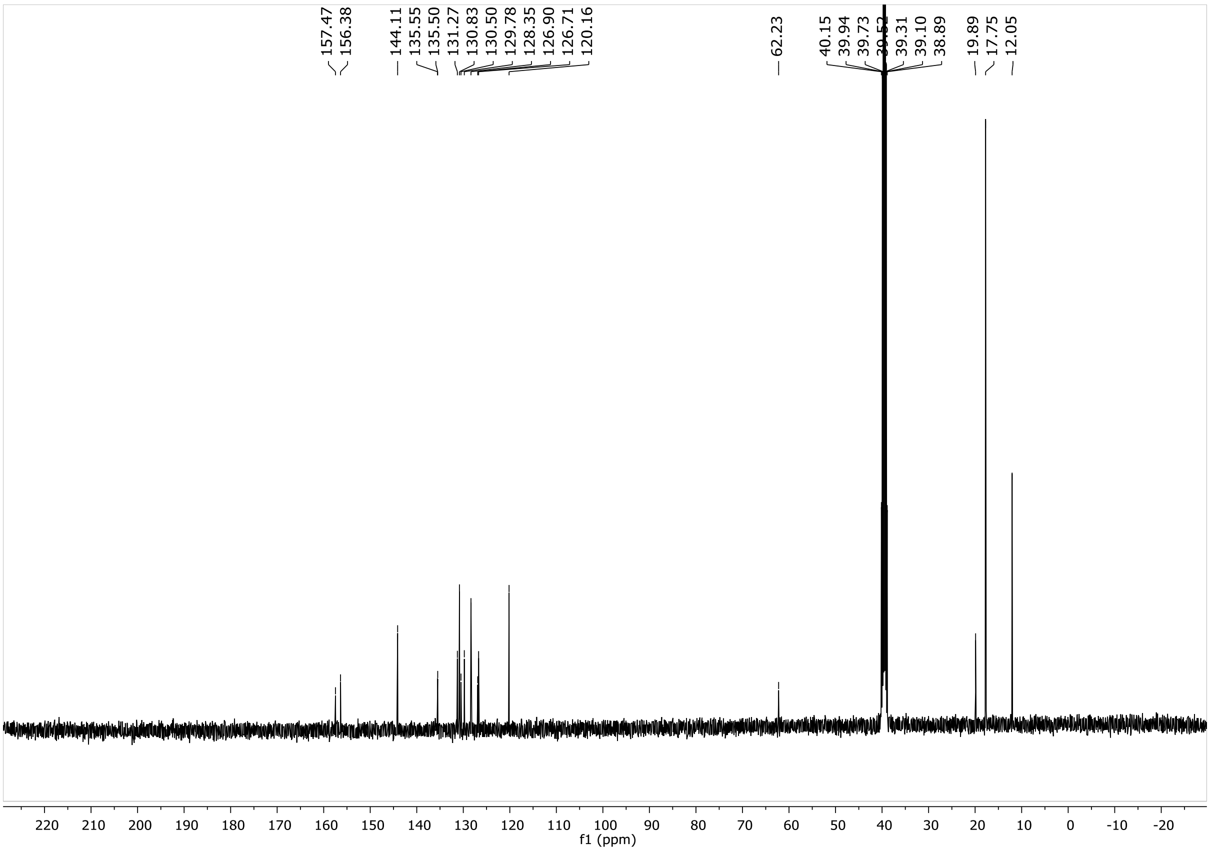
157.47
156.38
144.11
135.55
135.50
131.27
130.83
130.50
129.78
128.35
126.90
126.71
120.16
62.23
40.15
39.94
39.73
39.31
39.10
38.89
19.89
17.75
12.05
f1 (ppm)

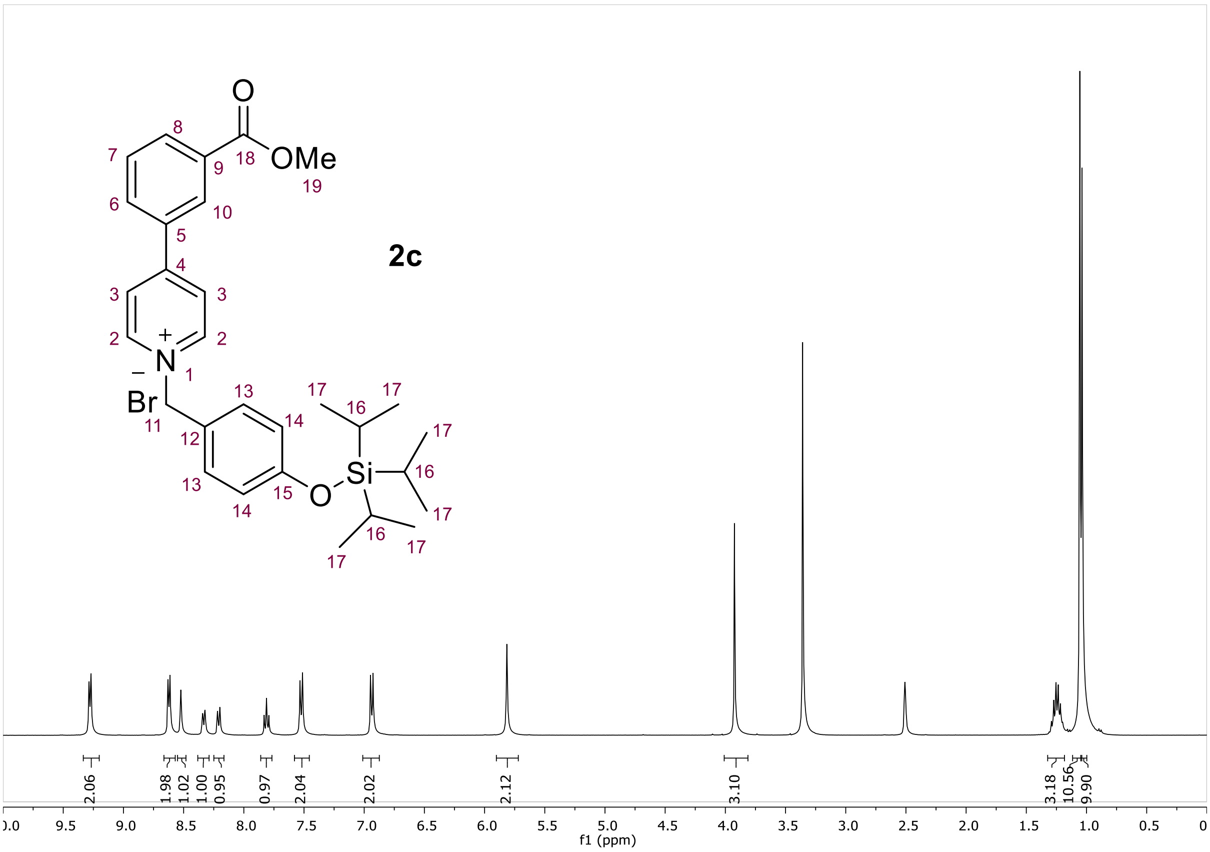

2c
O
OMe
18
19
Br
N
Si
O
2.06
1.98
1.02
1.00
0.95
0.97
2.04
2.02
2.12
3.10
3.18
10.56
9.90
f1 (ppm)

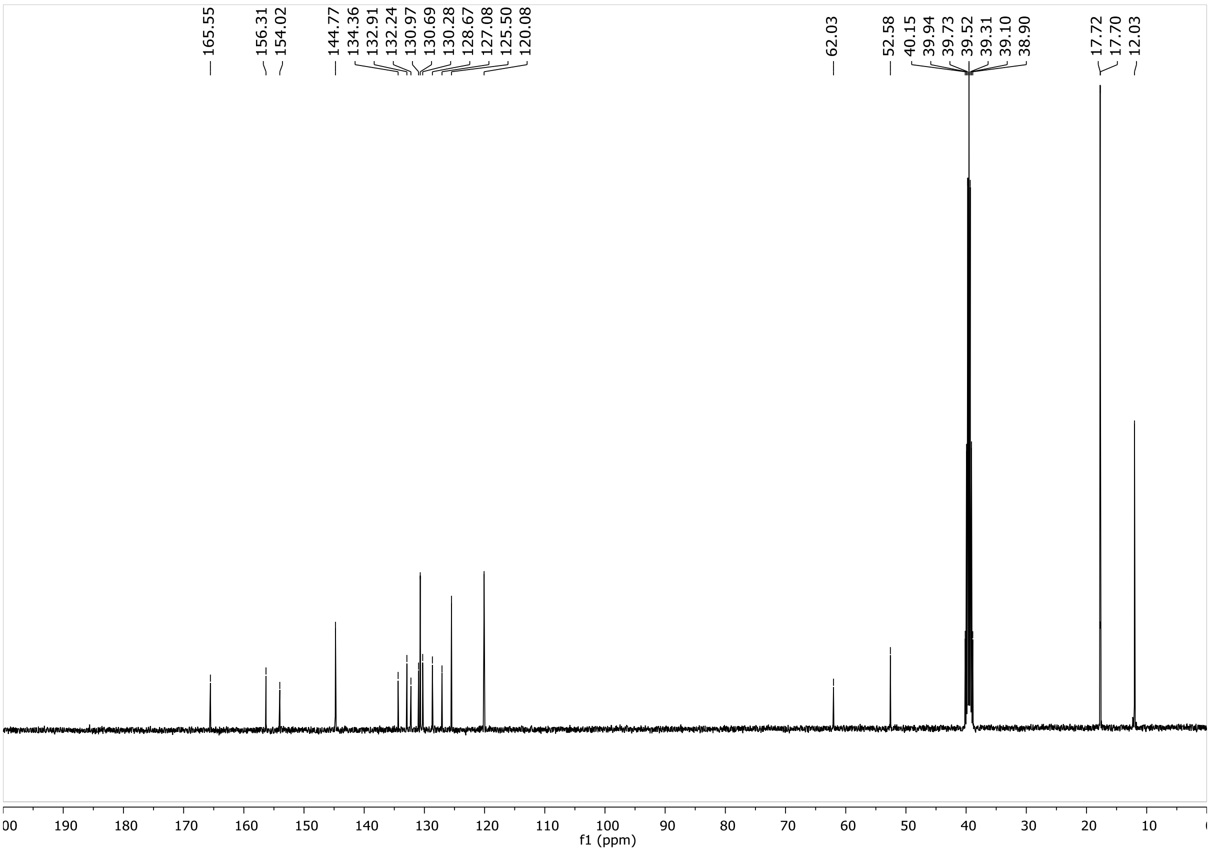

165.55
156.31
154.02
144.77
134.36
132.91
132.24
130.97
130.69
130.28
128.67
127.08
125.50
120.08
62.03
52.58
40.15
39.94
39.73
39.52
39.31
39.10
38.90
17.72
17.70
12.03
f1 (ppm)

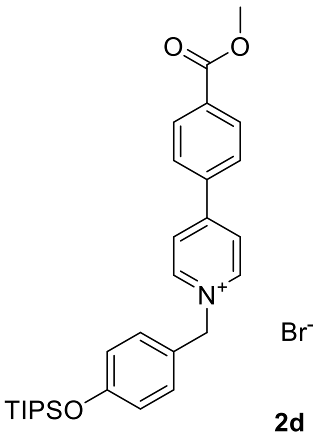
TIPSO
N+
Br-

2d

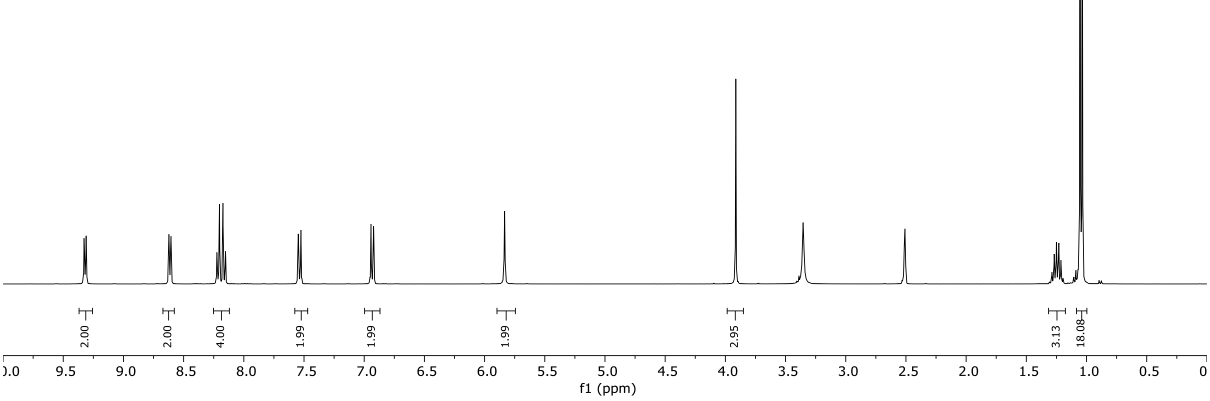
2.00
2.00
4.00
1.99
1.99
1.99
2.95
3.13
18.08
f1 (ppm)

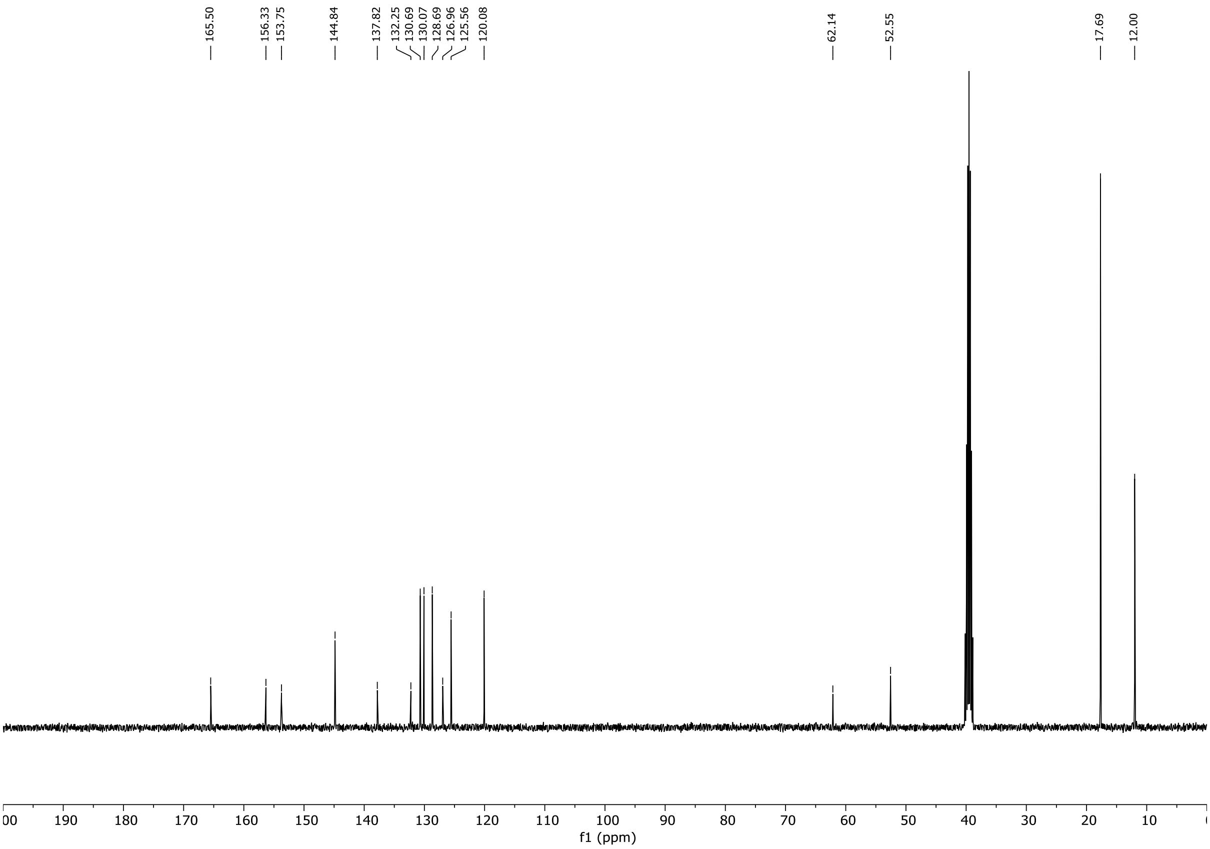
165.50
156.33
153.75
144.84
137.82
132.25
130.69
130.07
128.69
126.96
125.56
120.08
62.14
52.55
17.69
12.00
f1 (ppm)

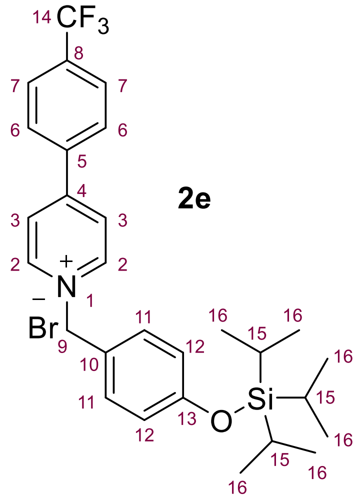

14 CF3
8
7
7
6
6
5
4
3
3
2
2
N
1
Br
9
10
11
11
12
12
13
O
Si
15
16

2e

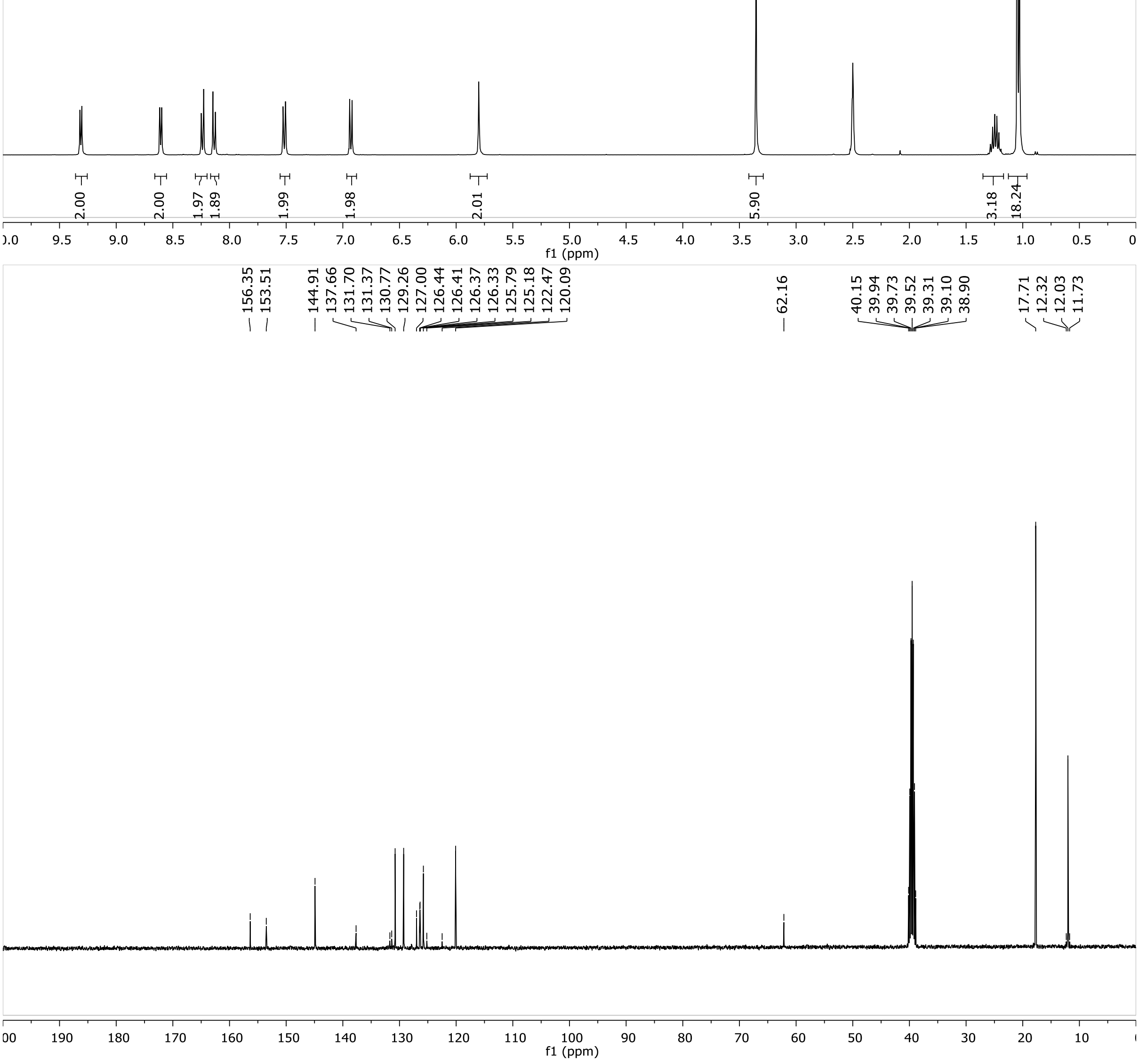

2.00
2.00
1.97
1.89
1.99
1.98
2.01
5.90
3.18
18.24
f1 (ppm)
156.35
153.51
144.91
137.66
131.70
131.37
130.77
129.26
127.00
126.44
126.41
126.37
126.33
125.79
125.18
122.47
120.09
62.16
40.15
39.94
39.73
39.52
39.31
39.10
38.90
17.71
12.32
12.03
11.73
f1 (ppm)

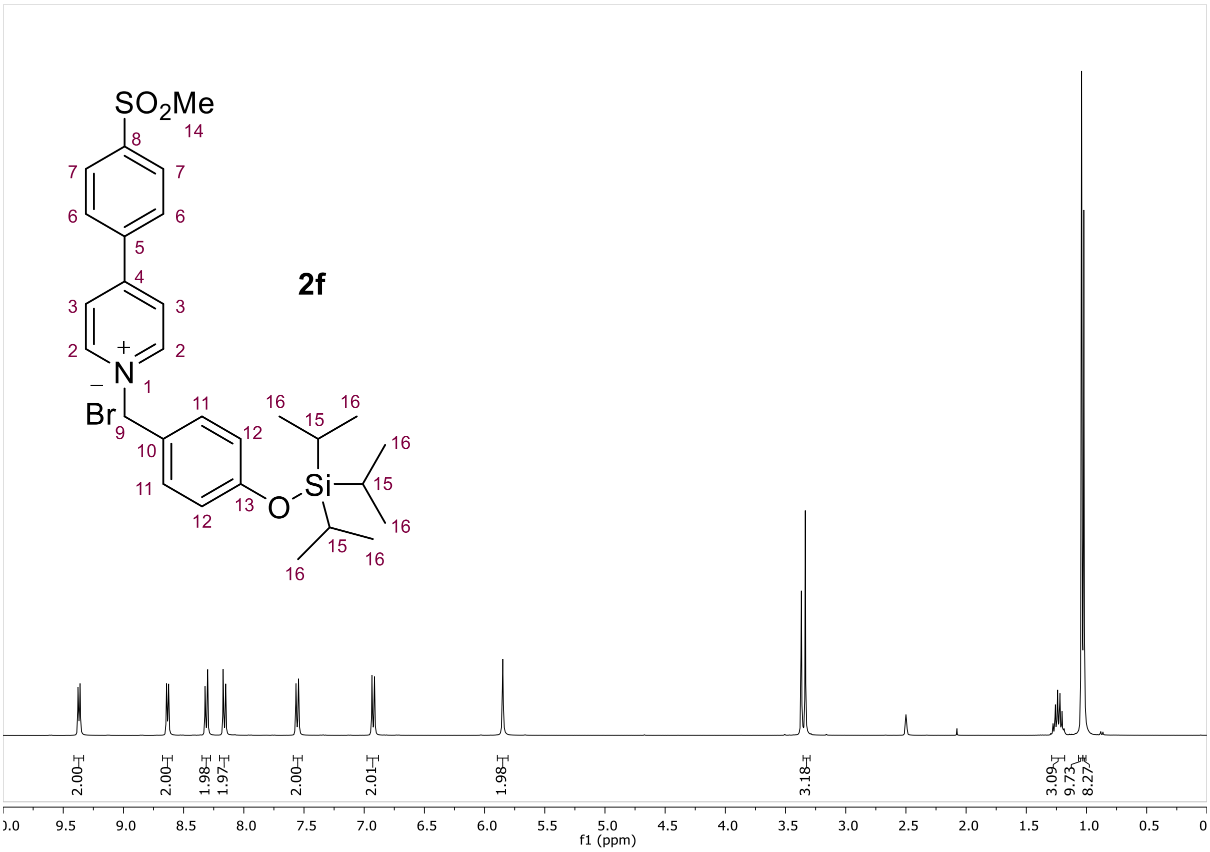
SO2Me
Br
N
O
Si
2f
2.00
2.00
1.98
1.97
2.00
2.01
1.98
3.18
3.09
9.73
8.27
f1 (ppm)

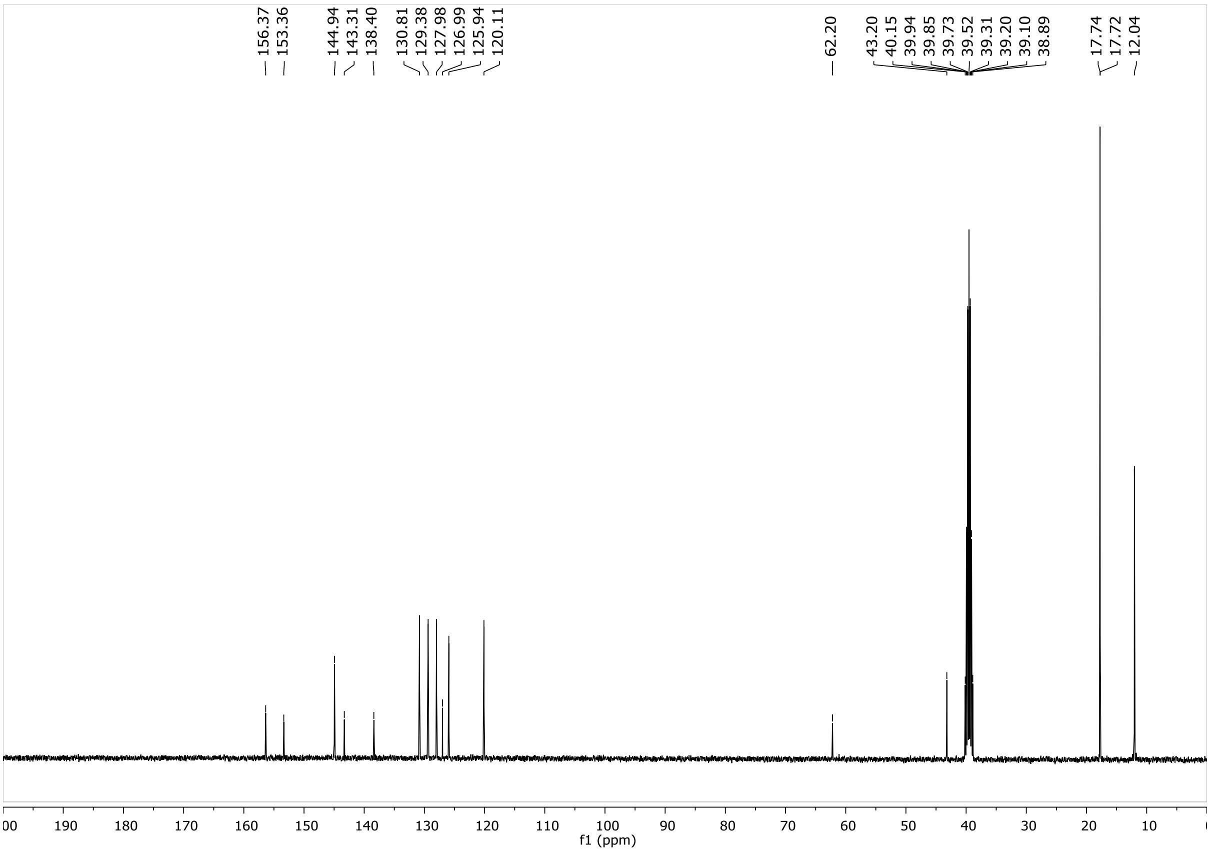
156.37
153.36
144.94
143.31
138.40
130.81
129.38
127.98
126.99
125.94
120.11
62.20
43.20
40.15
39.94
39.85
39.73
39.52
39.31
39.20
39.10
38.89
17.74
17.72
12.04
f1 (ppm)

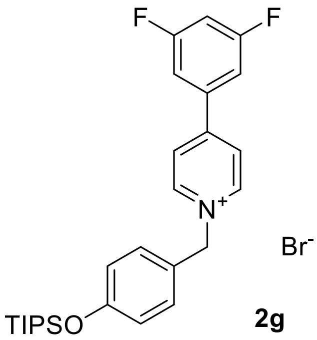
F
F
N+
Br-
TIPSO

2g

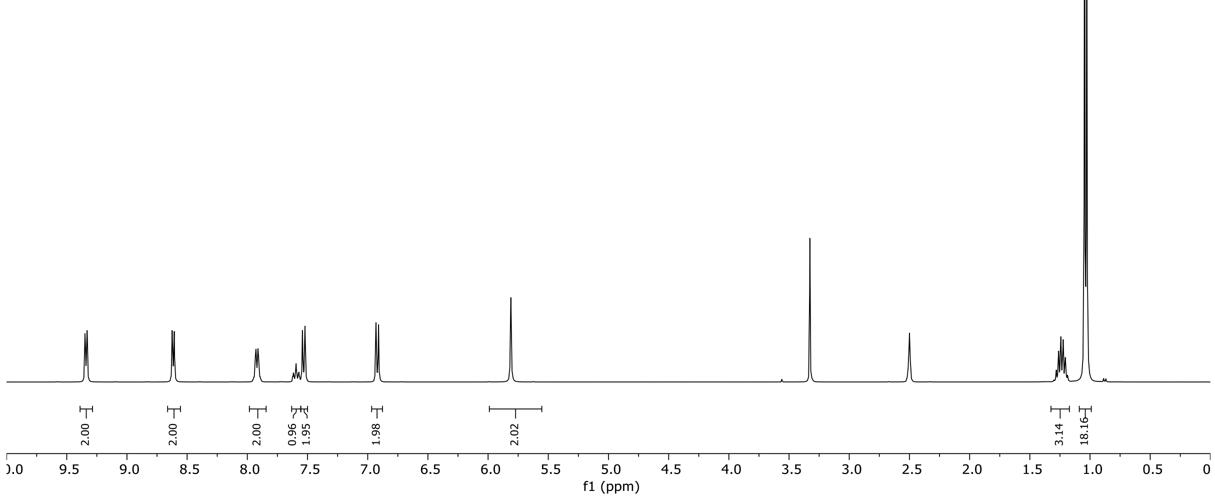
2.00
2.00
2.00
0.96
1.95
1.98
2.02
3.14
18.16
f1 (ppm)

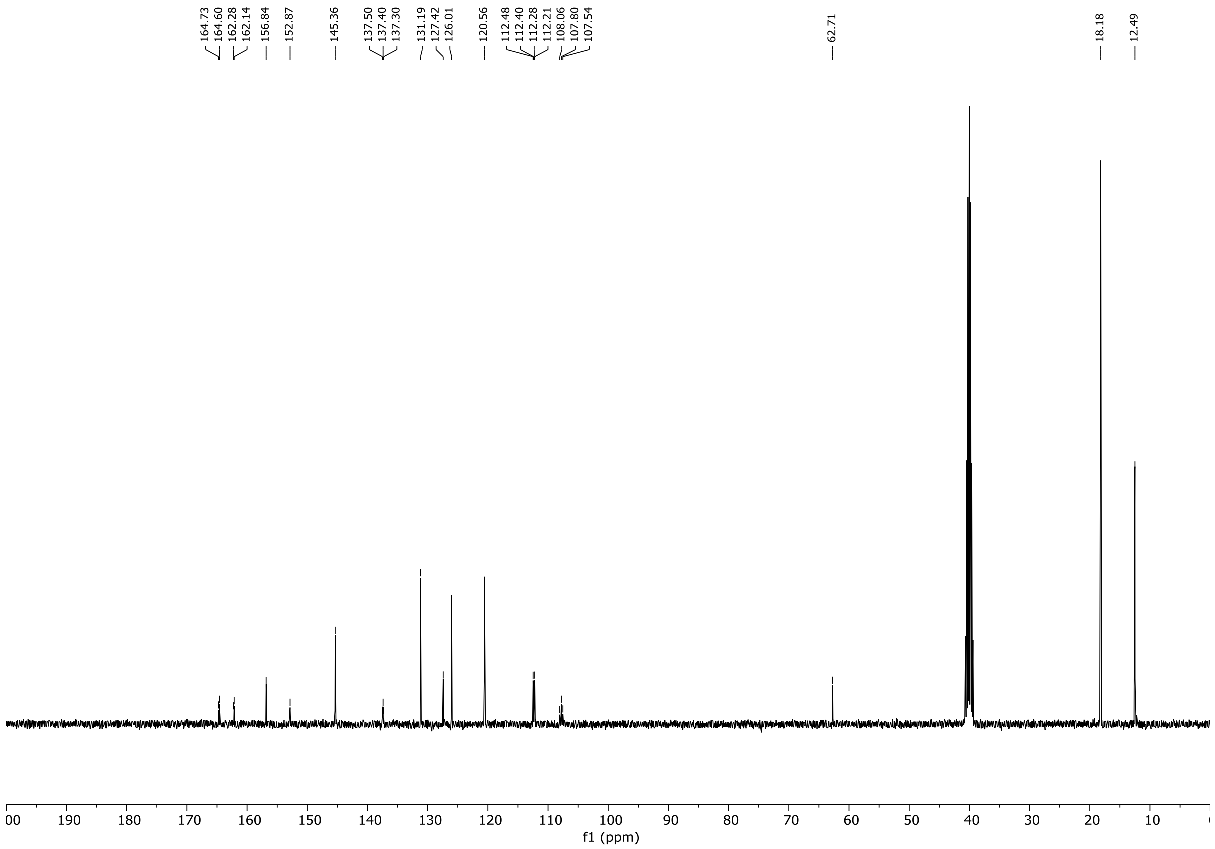
164.73
164.60
162.28
162.14
156.84
152.87
145.36
137.50
137.40
137.30
131.19
127.42
126.01
120.56
112.48
112.40
112.28
112.21
108.06
107.80
107.54
62.71
18.18
12.49
f1 (ppm)

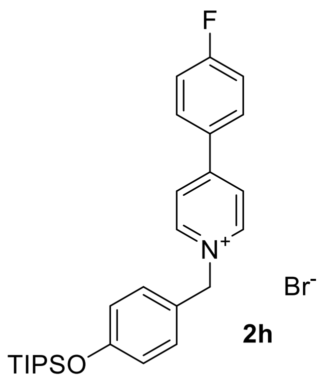
F
N+
Br-
TIPSO

2h

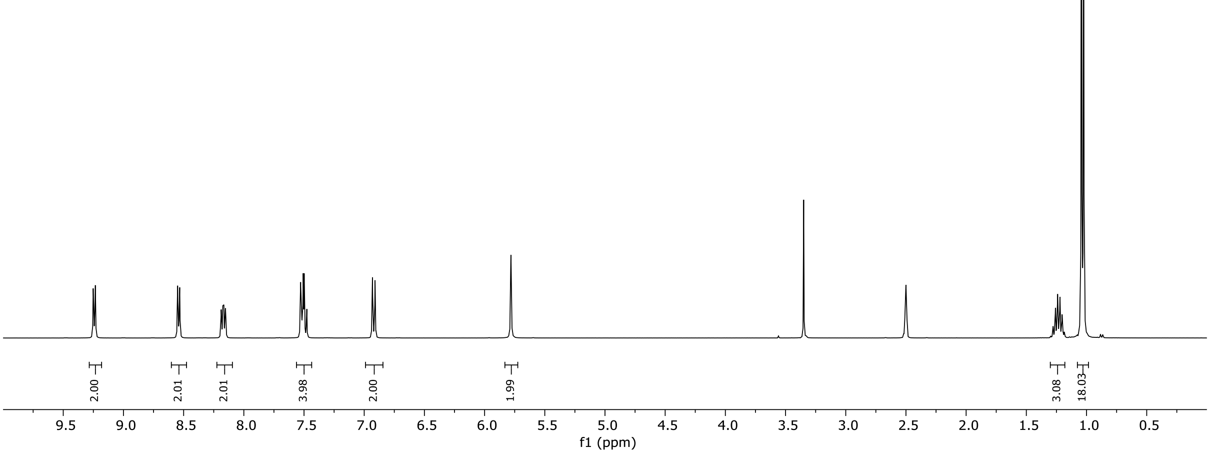
2.00
2.01
2.01
3.98
2.00
1.99
3.08
18.03
9.5 9.0 8.5 8.0 7.5 7.0 6.5 6.0 5.5 5.0 4.5 4.0 3.5 3.0 2.5 2.0 1.5 1.0 0.5
f1 (ppm)

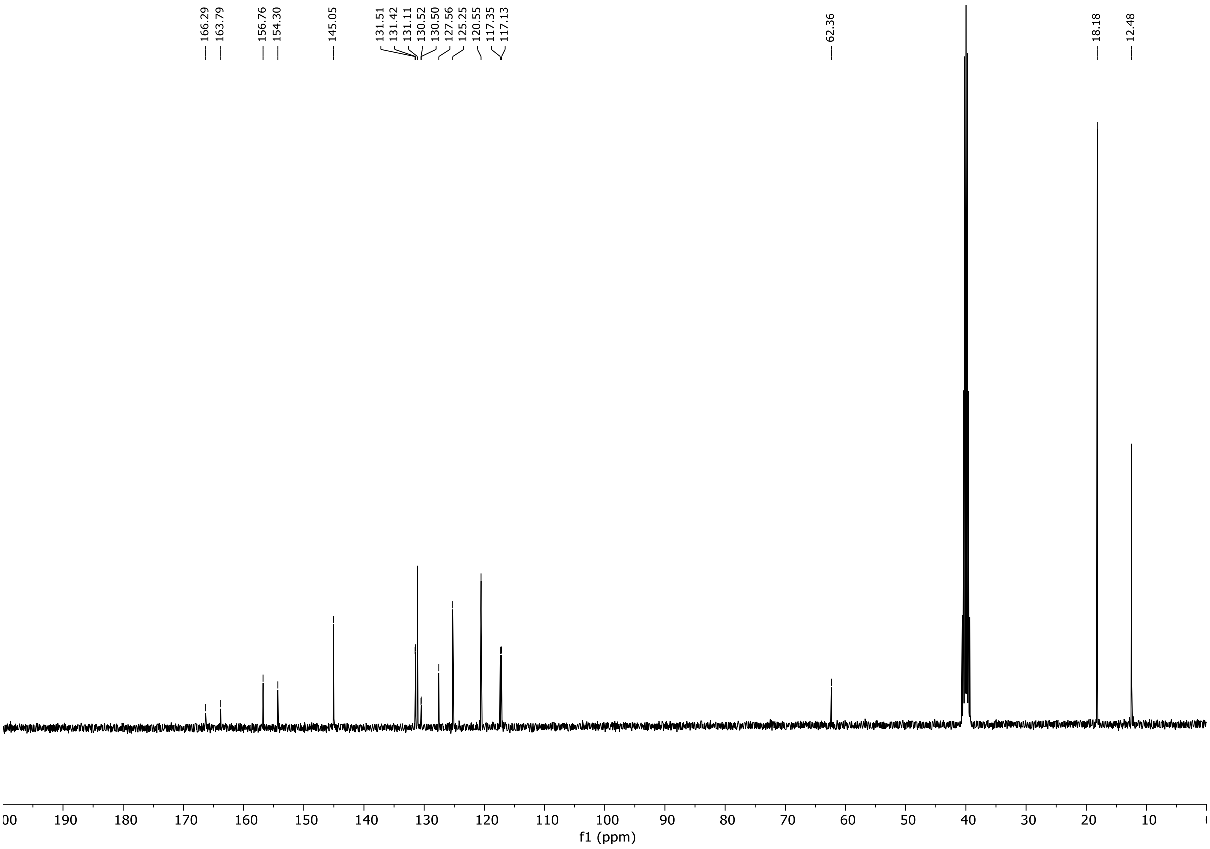
166.29
163.79
156.76
154.30
145.05
131.51
131.42
131.11
130.52
130.50
127.56
125.25
120.55
117.35
117.13
62.36
18.18
12.48
190 180 170 160 150 140 130 120 110 100 90 80 70 60 50 40 30 20 10
f1 (ppm)

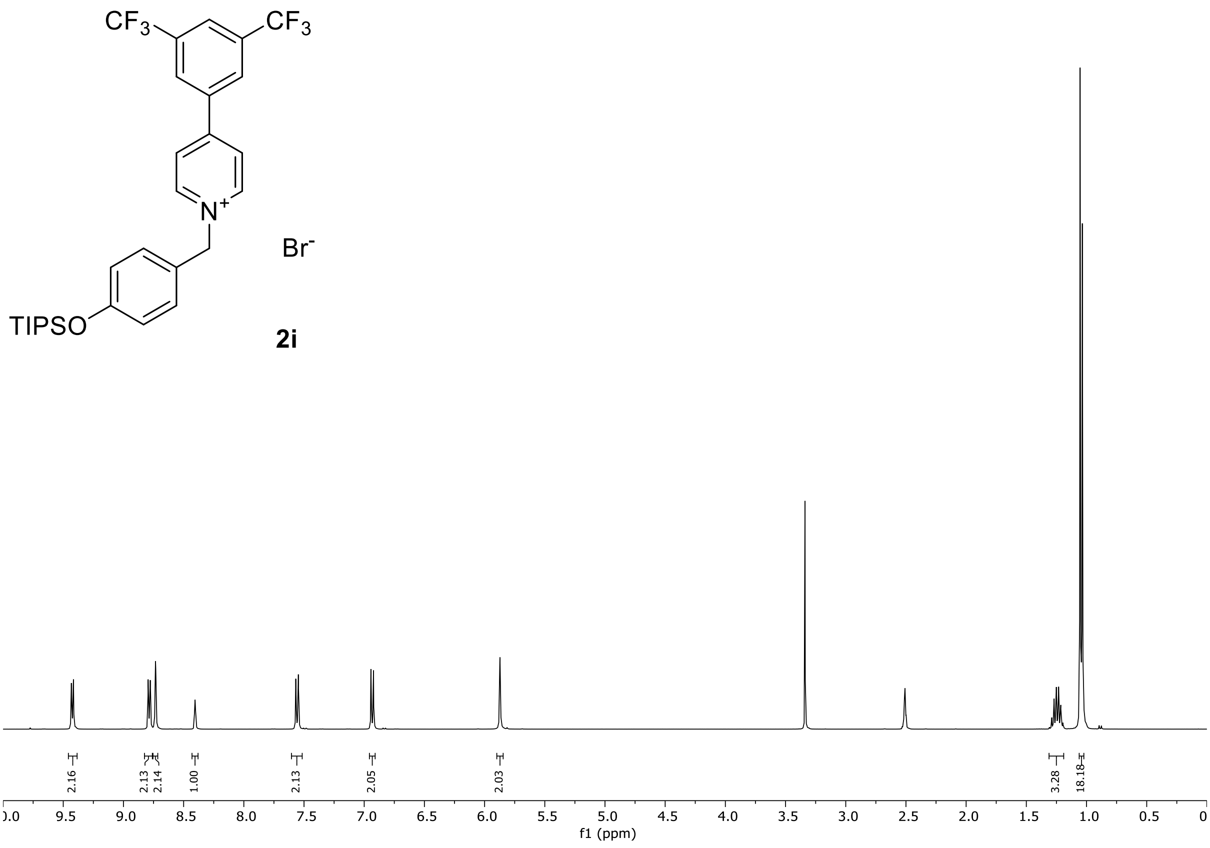
CF3
CF3
N+
Br-
TIPSO
2i
2.16
2.13
2.14
1.00
2.13
2.05
2.03
3.28
18.18
f1 (ppm)

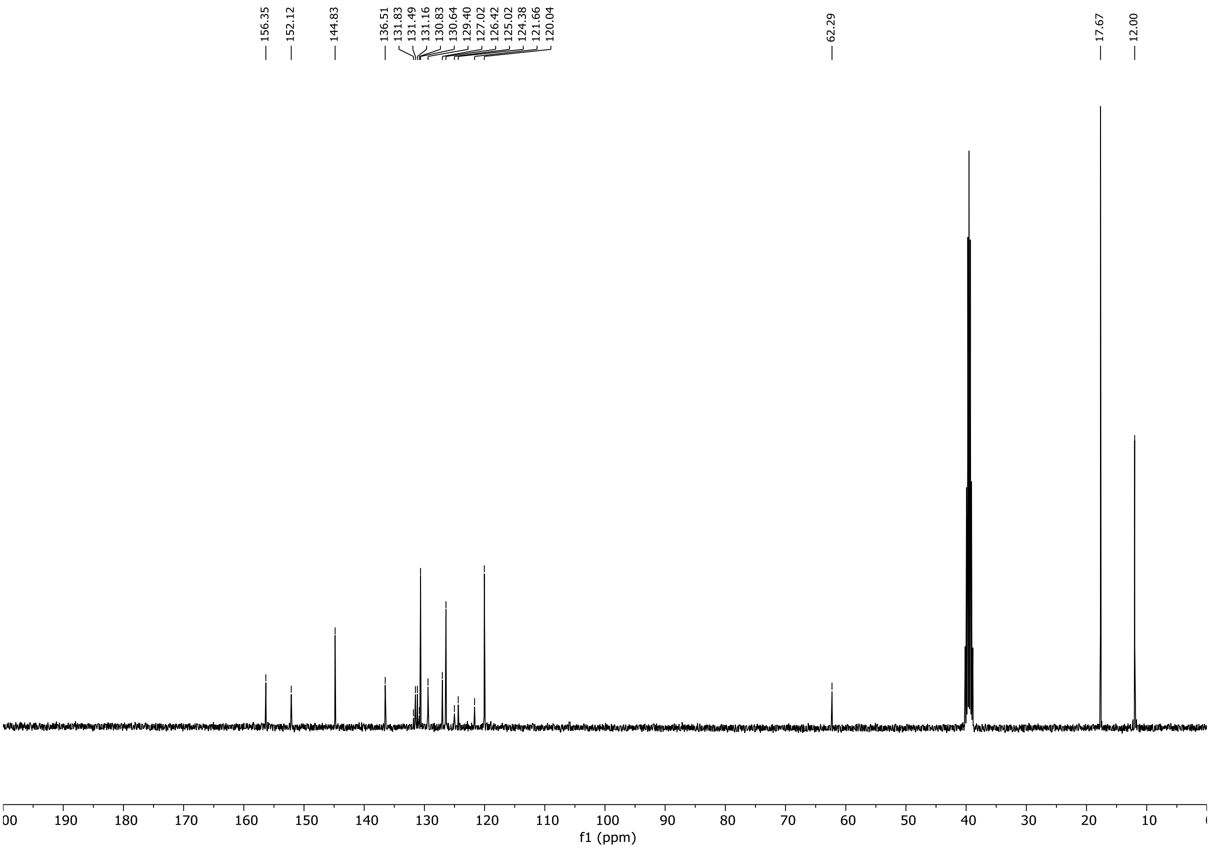
156.35
152.12
144.83
136.51
131.83
131.49
131.16
130.83
130.64
129.40
127.02
126.42
125.02
124.38
121.66
120.04
62.29
17.67
12.00
f1 (ppm)

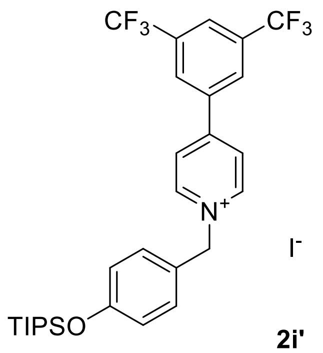

CF3
CF3
N+
TIPSO

I-

2i'

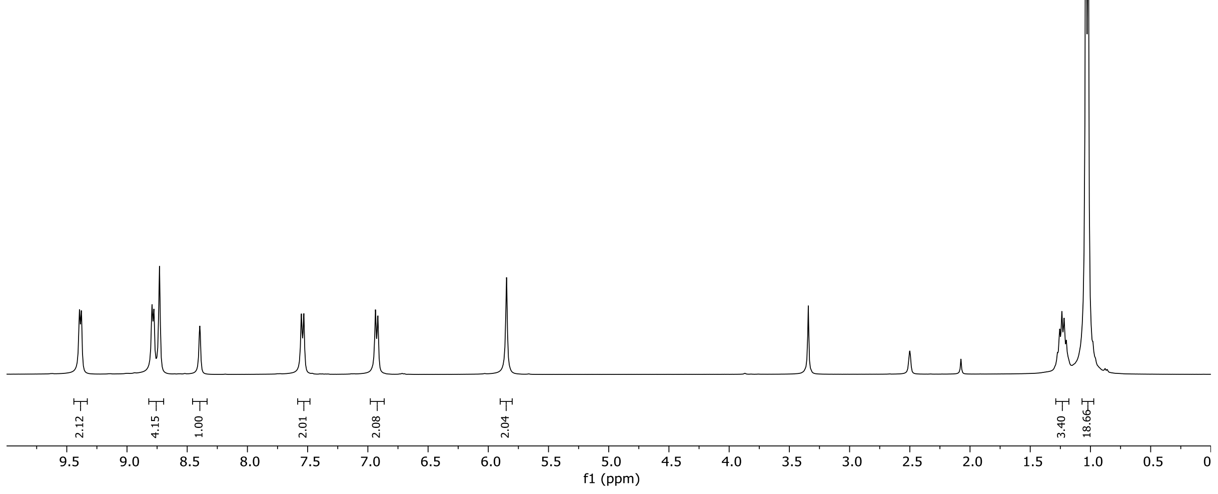

2.12
4.15
1.00
2.01
2.08
2.04
3.40
18.66
f1 (ppm)

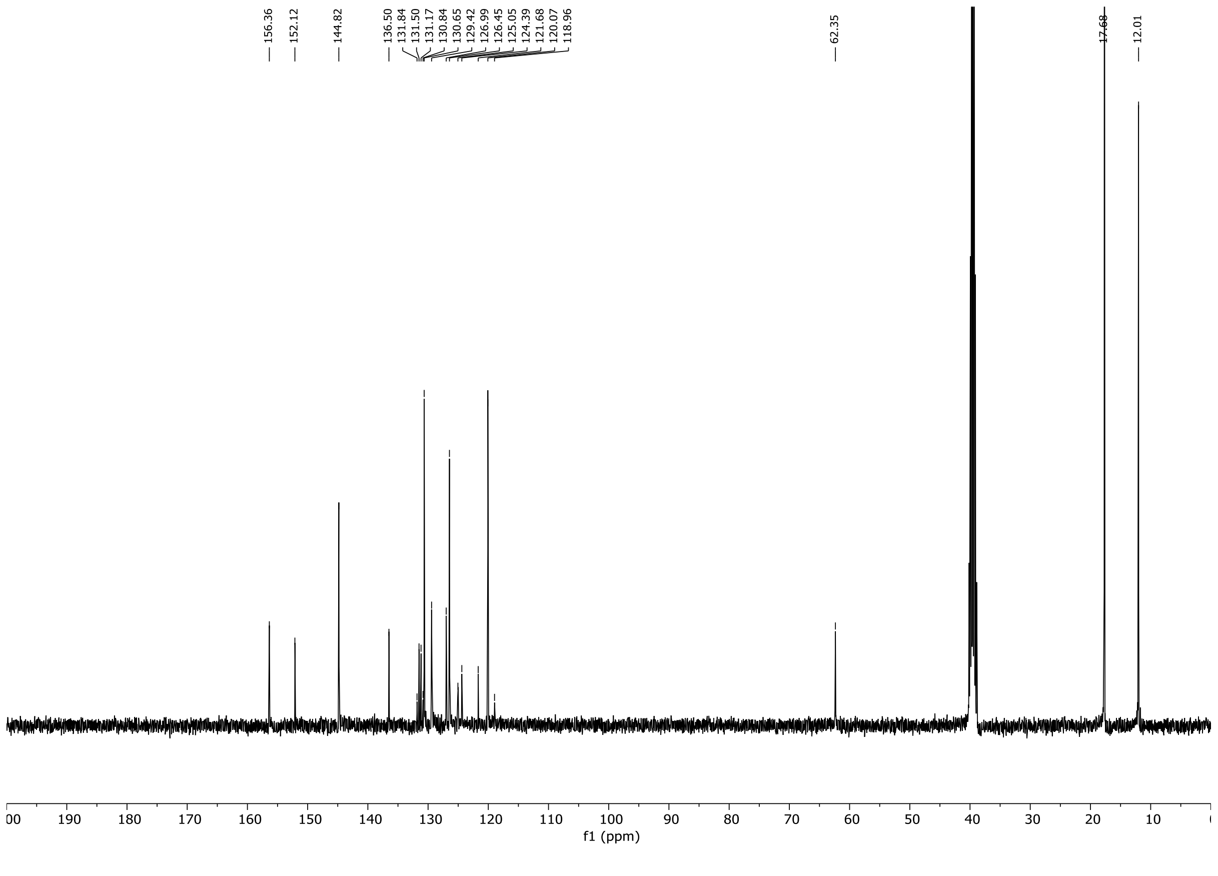

156.36
152.12
144.82
136.50
131.84
131.50
131.17
130.84
130.65
129.42
126.99
126.45
125.05
124.39
121.68
120.07
118.96
62.35
17.68
12.01
f1 (ppm)

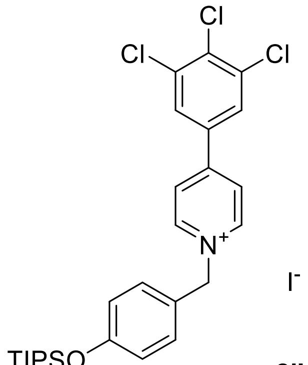

Cl
Cl
Cl
N+
I-
TIPSO

2j'

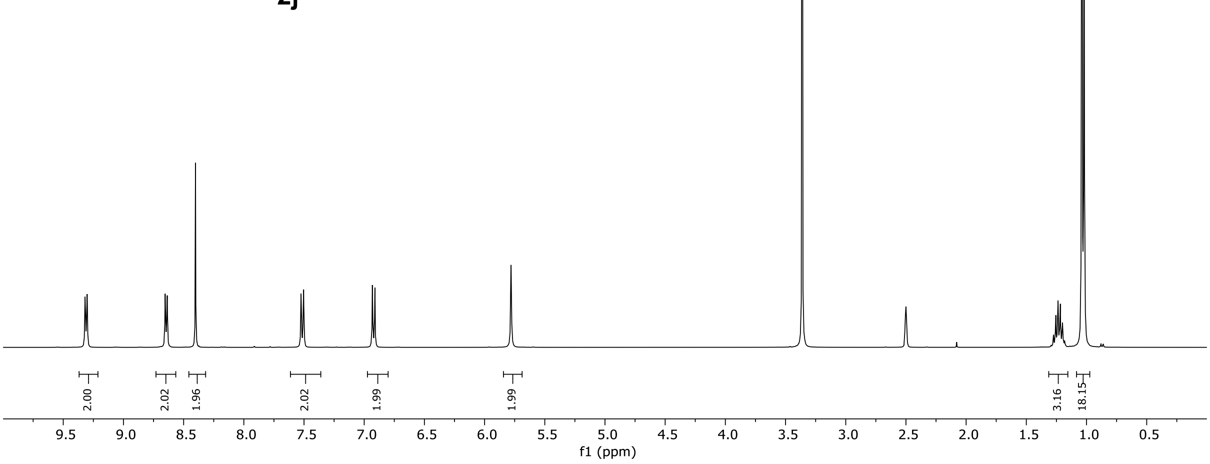

2.00
2.02
1.96
2.02
1.99
1.99
3.16
18.15
9.5 9.0 8.5 8.0 7.5 7.0 6.5 6.0 5.5 5.0 4.5 4.0 3.5 3.0 2.5 2.0 1.5 1.0 0.5
f1 (ppm)

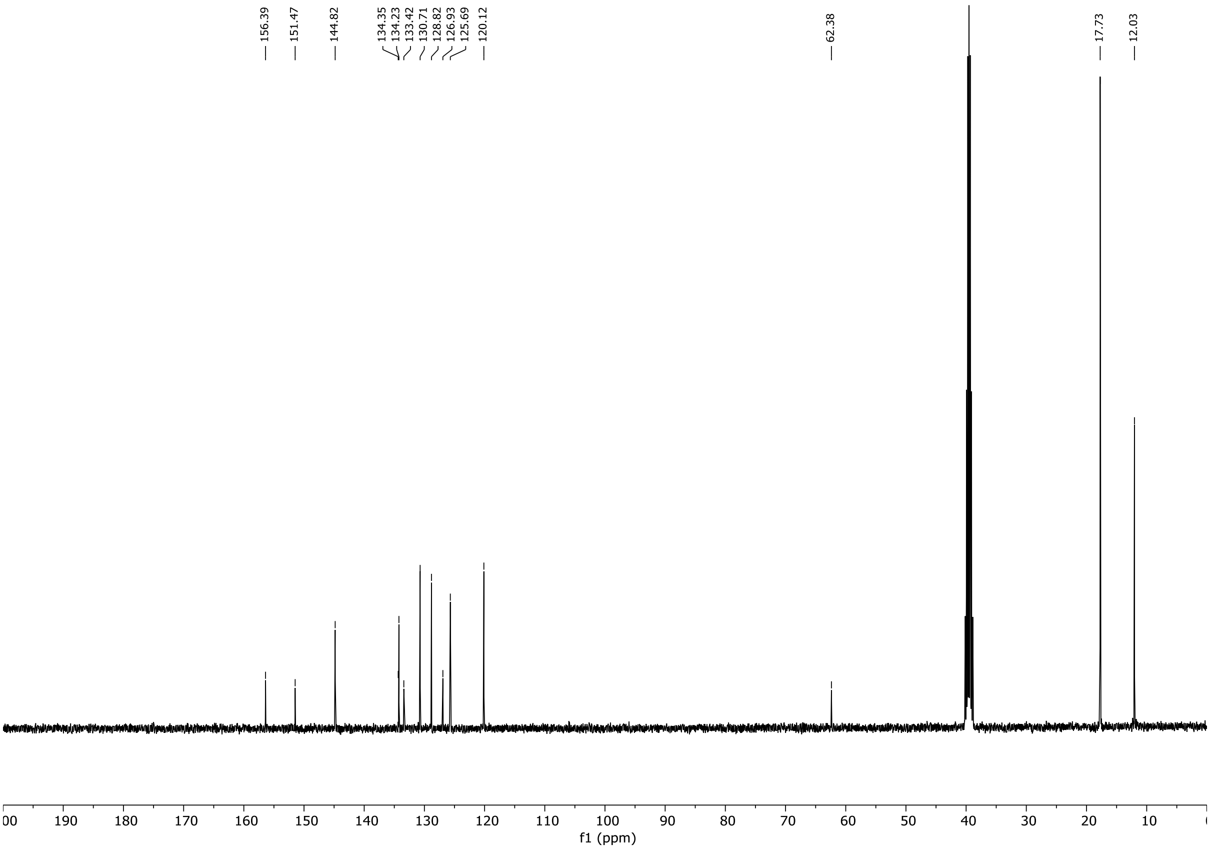

156.39
151.47
144.82
134.35
134.23
133.42
130.71
128.82
126.93
125.69
120.12
62.38
17.73
12.03
190 180 170 160 150 140 130 120 110 100 90 80 70 60 50 40 30 20 10
f1 (ppm)

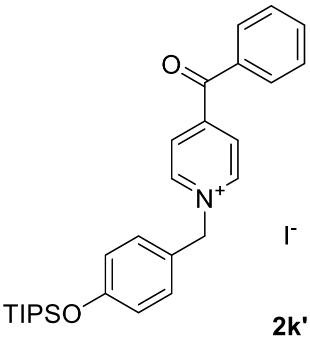
O
N+
I-
TIPSO

2k'

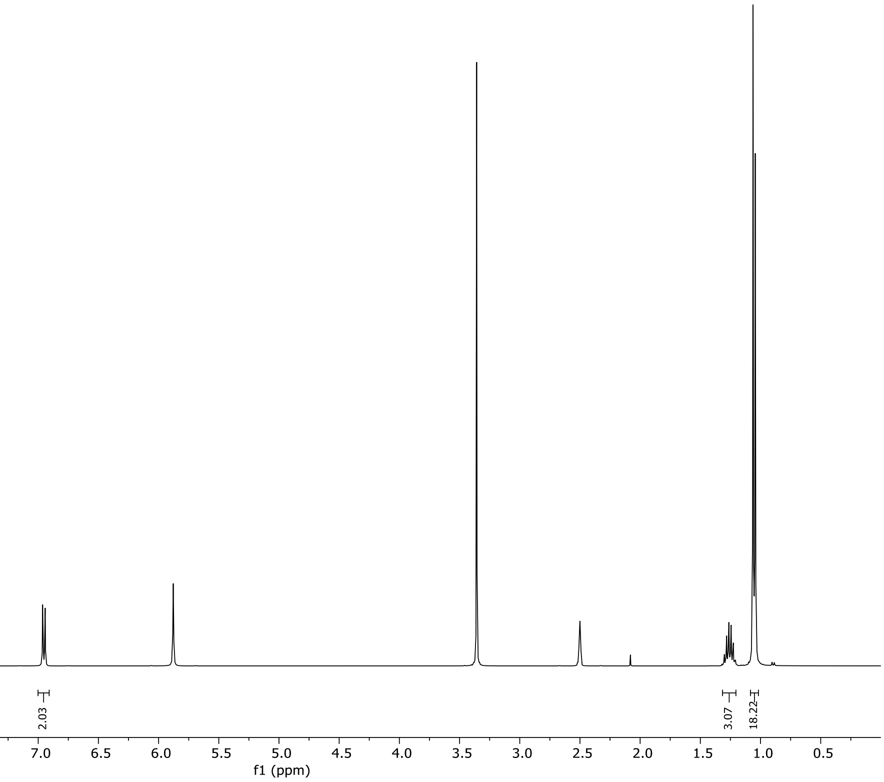
2.01
1.95
2.00
1.04
2.07
2.03
2.03
3.07
18.22
9.5
9.0
8.5
8.0
7.5
7.0
6.5
6.0
5.5
5.0
4.5
4.0
3.5
3.0
2.5
2.0
1.5
1.0
0.5
f1 (ppm)

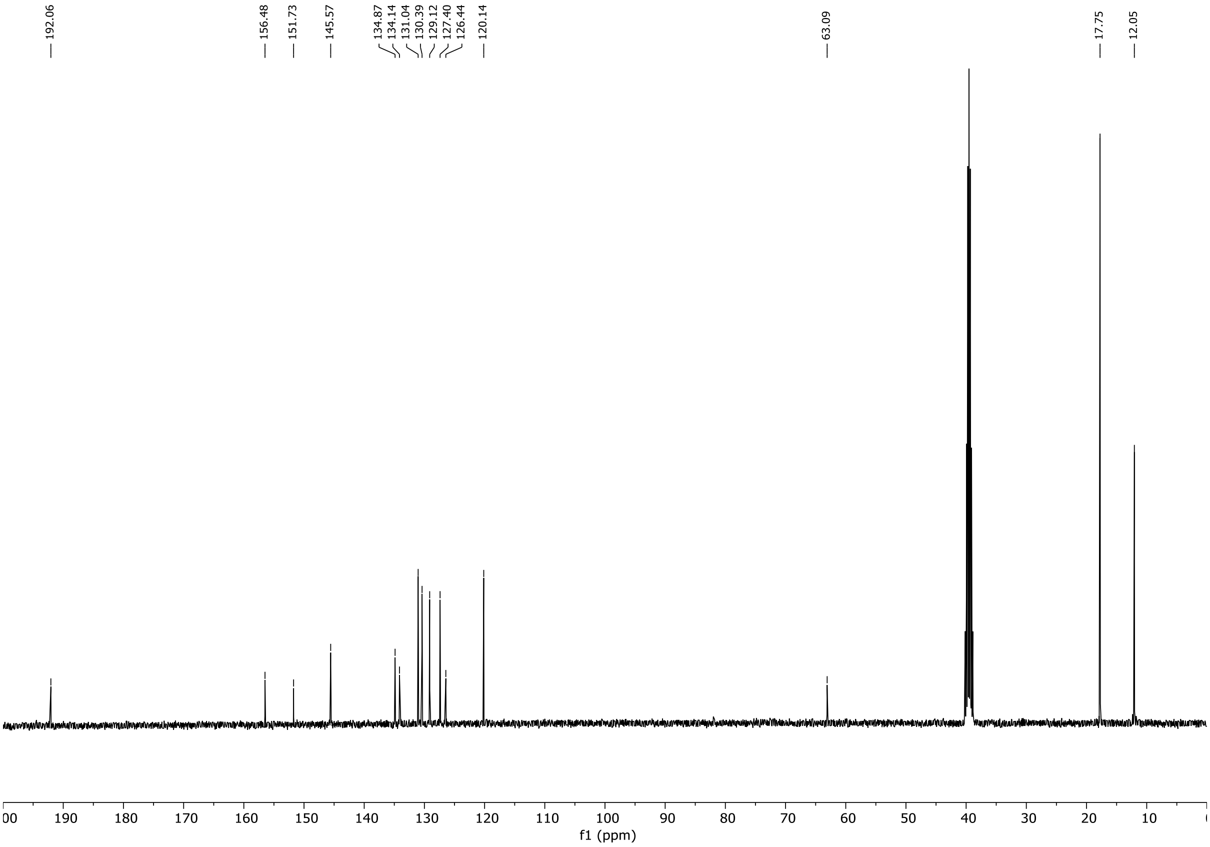
192.06
156.48
151.73
145.57
134.87
134.14
131.04
130.39
129.12
127.40
126.44
120.14
63.09
17.75
12.05
190
180
170
160
150
140
130
120
110
100
90
80
70
60
50
40
30
20
10
f1 (ppm)

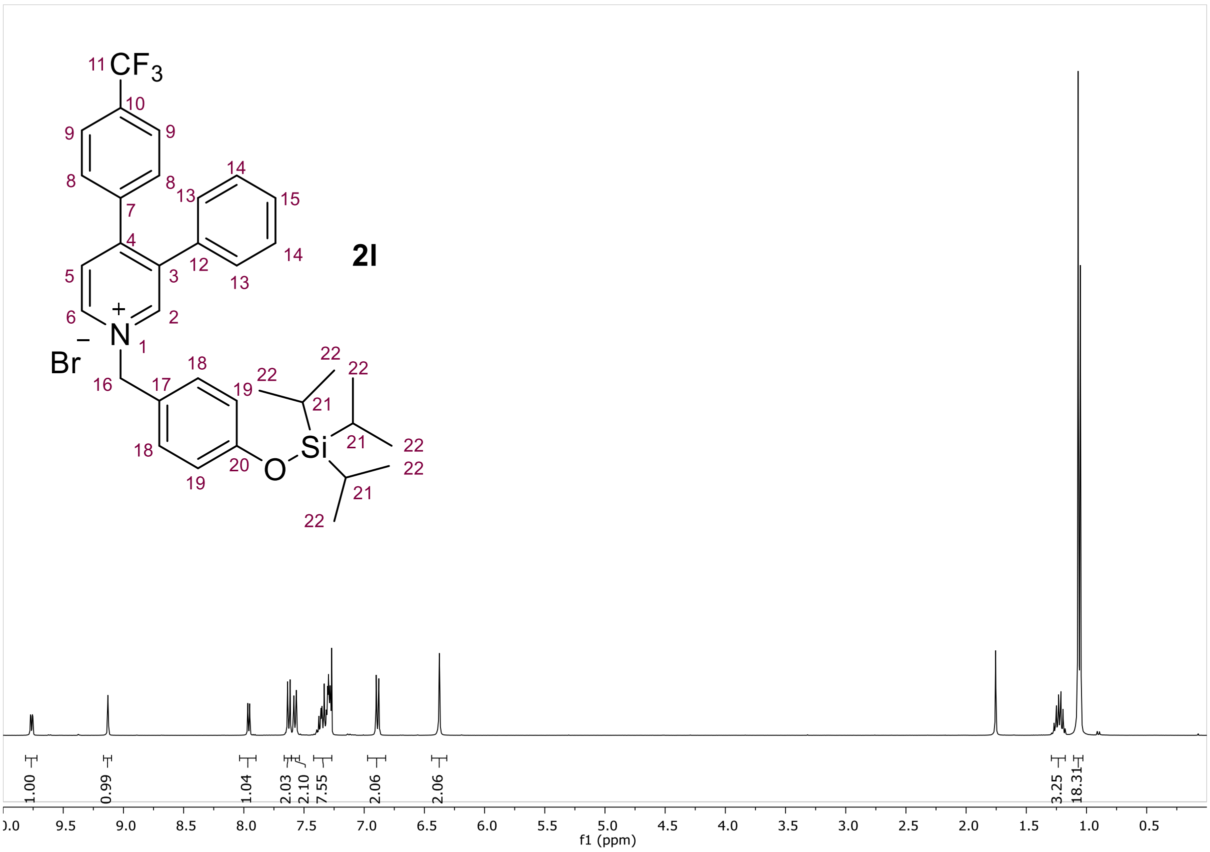

2l
1.00
0.99
1.04
2.03
2.10
7.55
2.06
2.06
3.25
18.31
f1 (ppm)

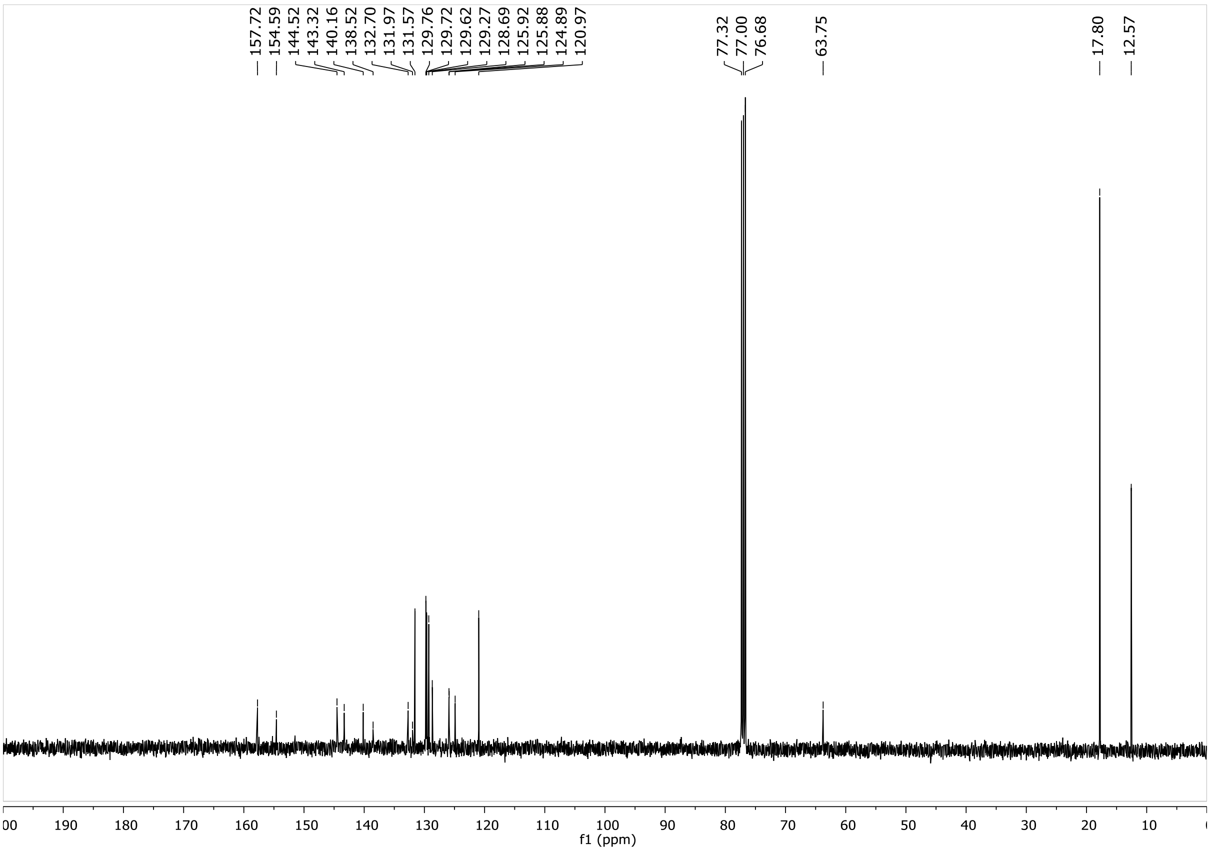

157.72
154.59
144.52
143.32
140.16
138.52
132.70
131.97
131.57
129.76
129.72
129.62
129.27
128.69
125.92
125.88
124.89
120.97
77.32
77.00
76.68
63.75
17.80
12.57
f1 (ppm)

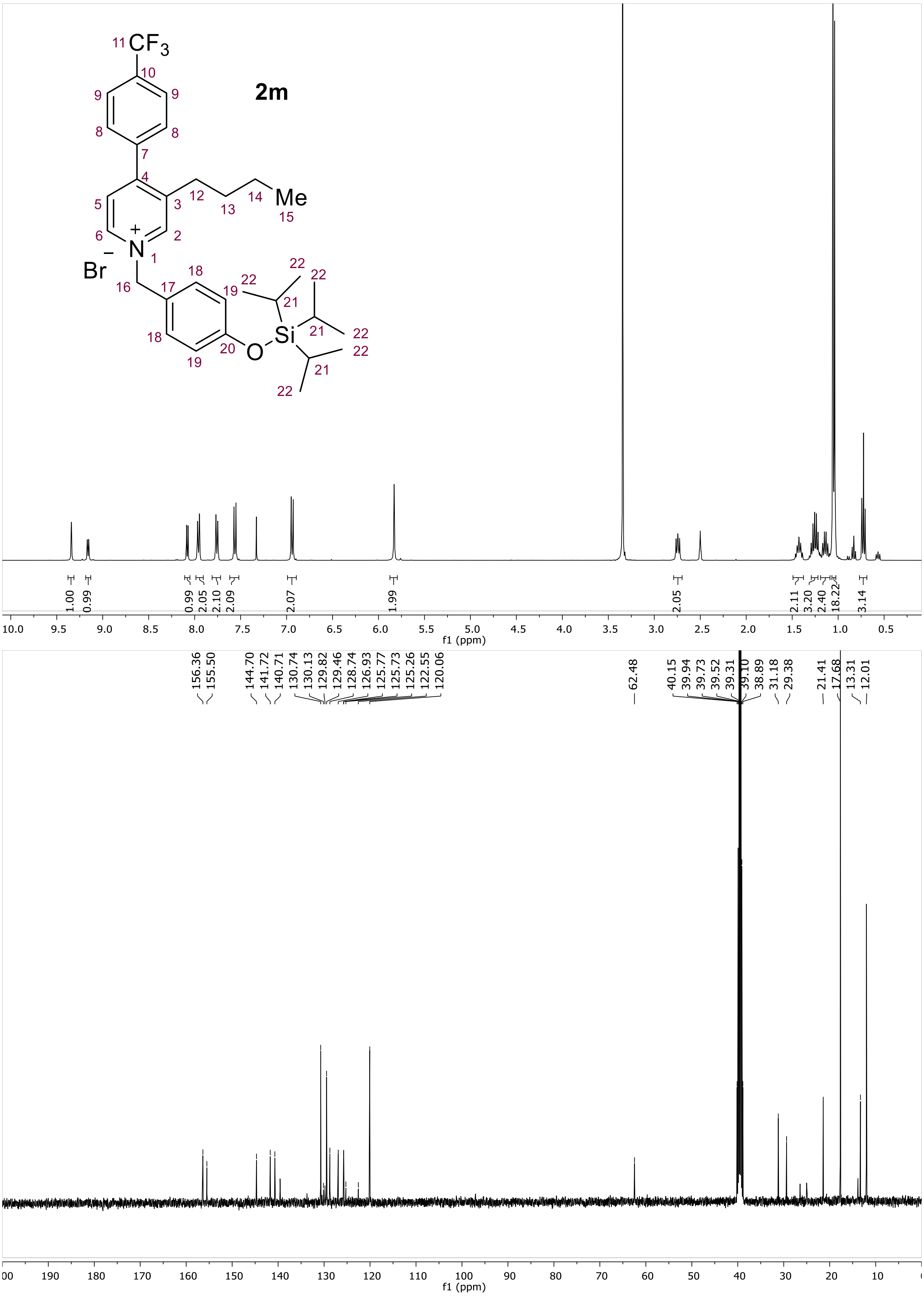

2m
f1 (ppm)
f1 (ppm)

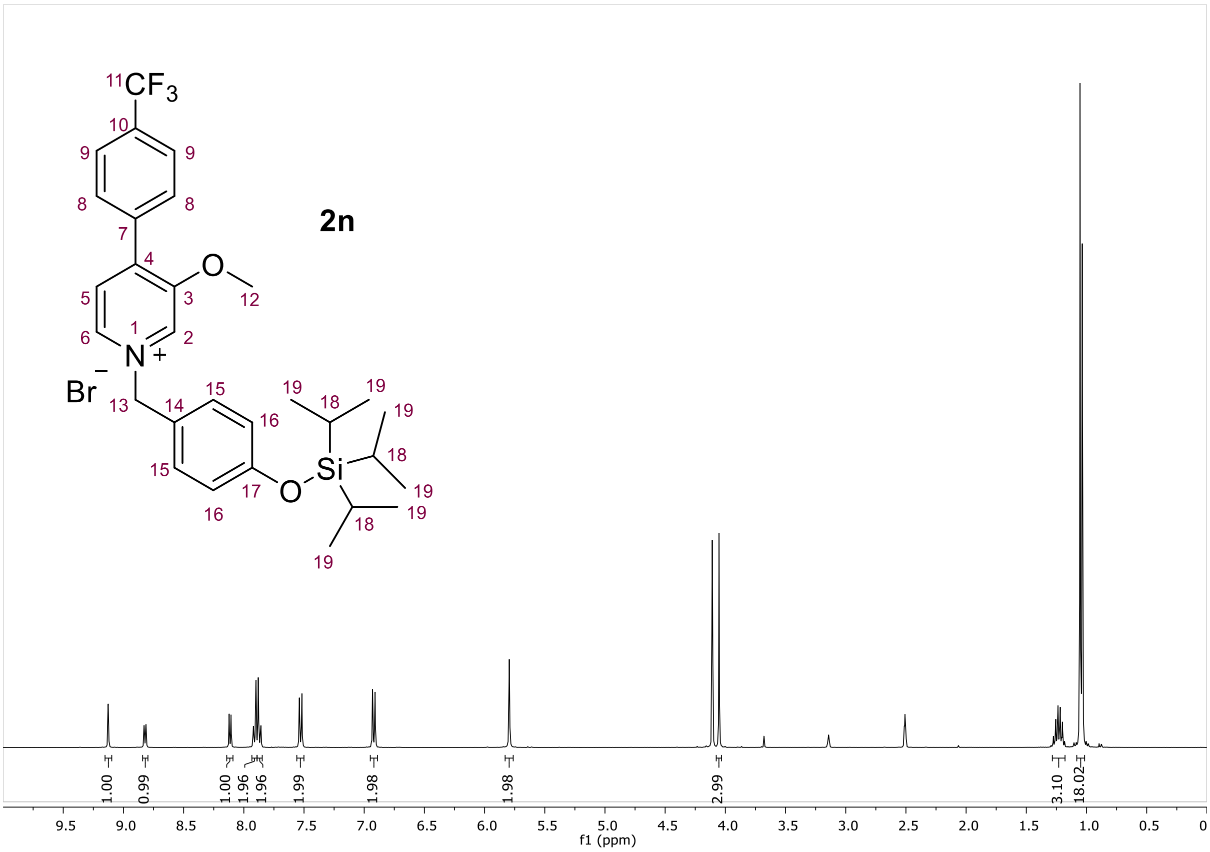
2n
CF3
Br
N
O
Si
1.00
0.99
1.00
1.96
1.96
1.99
1.98
1.98
2.99
3.10
18.02
f1 (ppm)

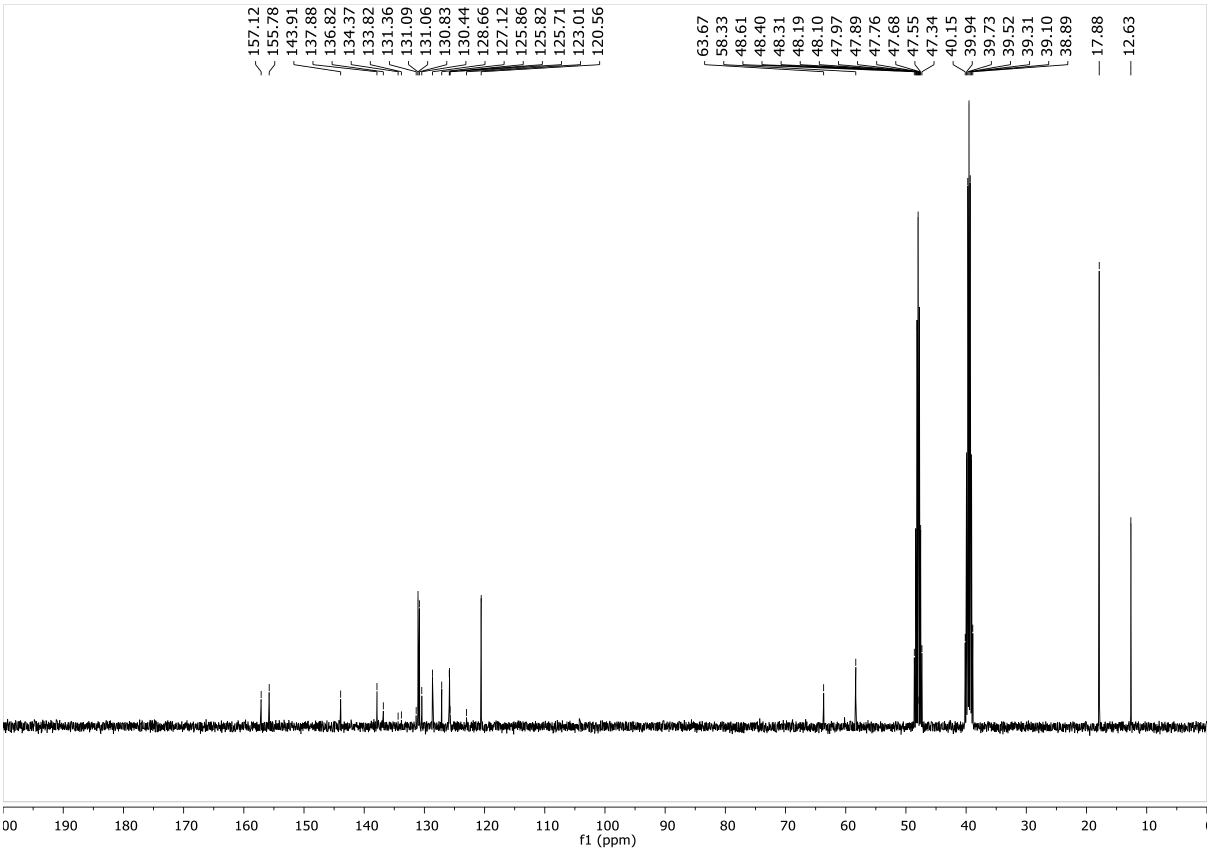
157.12
155.78
143.91
137.88
136.82
134.37
133.82
131.36
131.09
131.06
130.83
130.44
128.66
127.12
125.86
125.82
125.71
123.01
120.56
63.67
58.33
48.61
48.40
48.31
48.19
48.10
47.97
47.89
47.76
47.68
47.55
47.34
40.15
39.94
39.73
39.52
39.31
39.10
38.89
17.88
12.63
f1 (ppm)

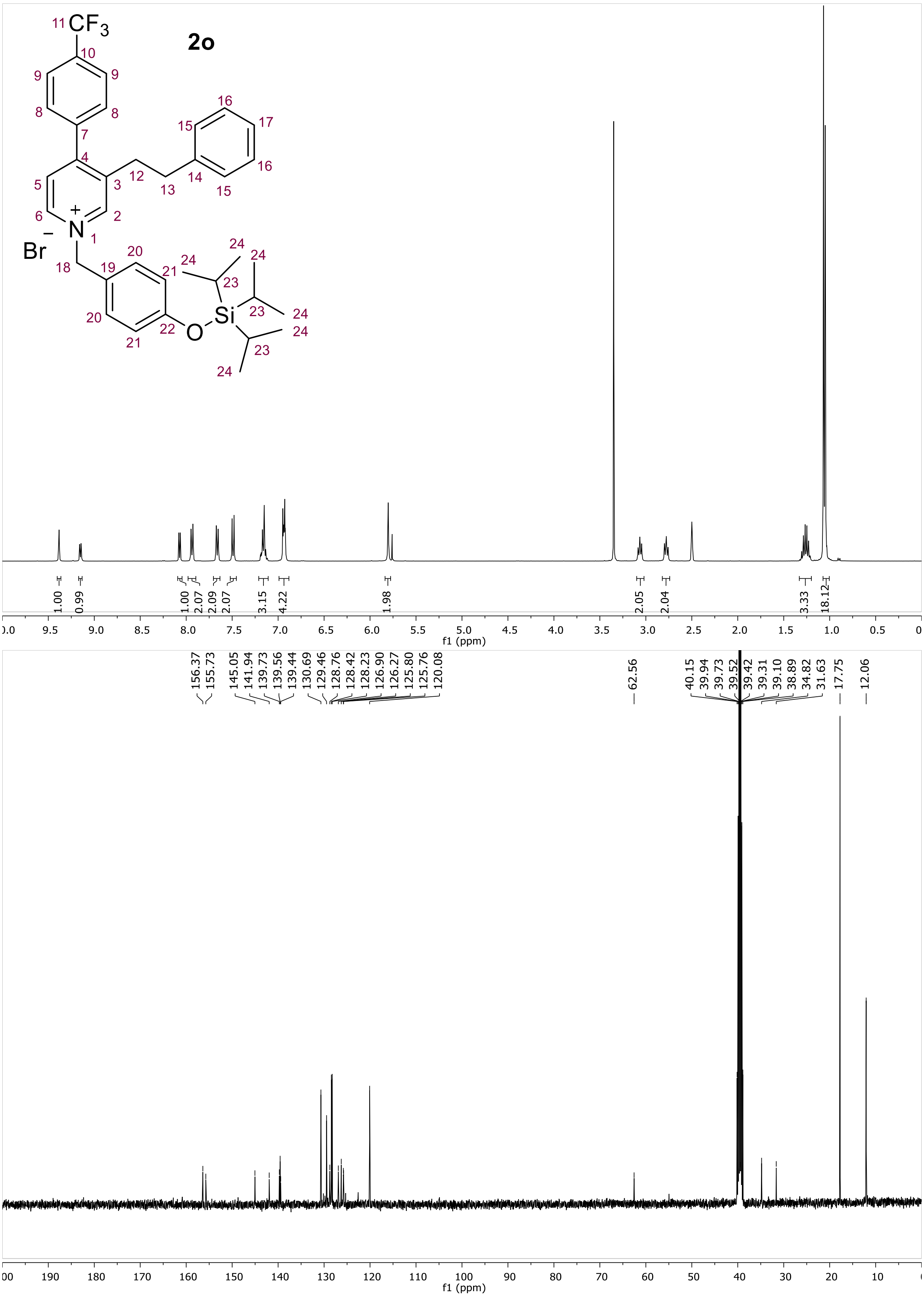
2o
CF3
Br
N
O
Si
1.00
0.99
1.00
2.07
2.09
2.07
3.15
4.22
1.98
2.05
2.04
3.33
18.12
f1 (ppm)
156.37
155.73
145.05
141.94
139.73
139.56
139.44
130.69
129.46
128.76
128.42
128.23
126.90
126.27
125.80
125.76
120.08
62.56
40.15
39.94
39.73
39.52
39.42
39.31
39.10
38.89
34.82
31.63
17.75
12.06
f1 (ppm)

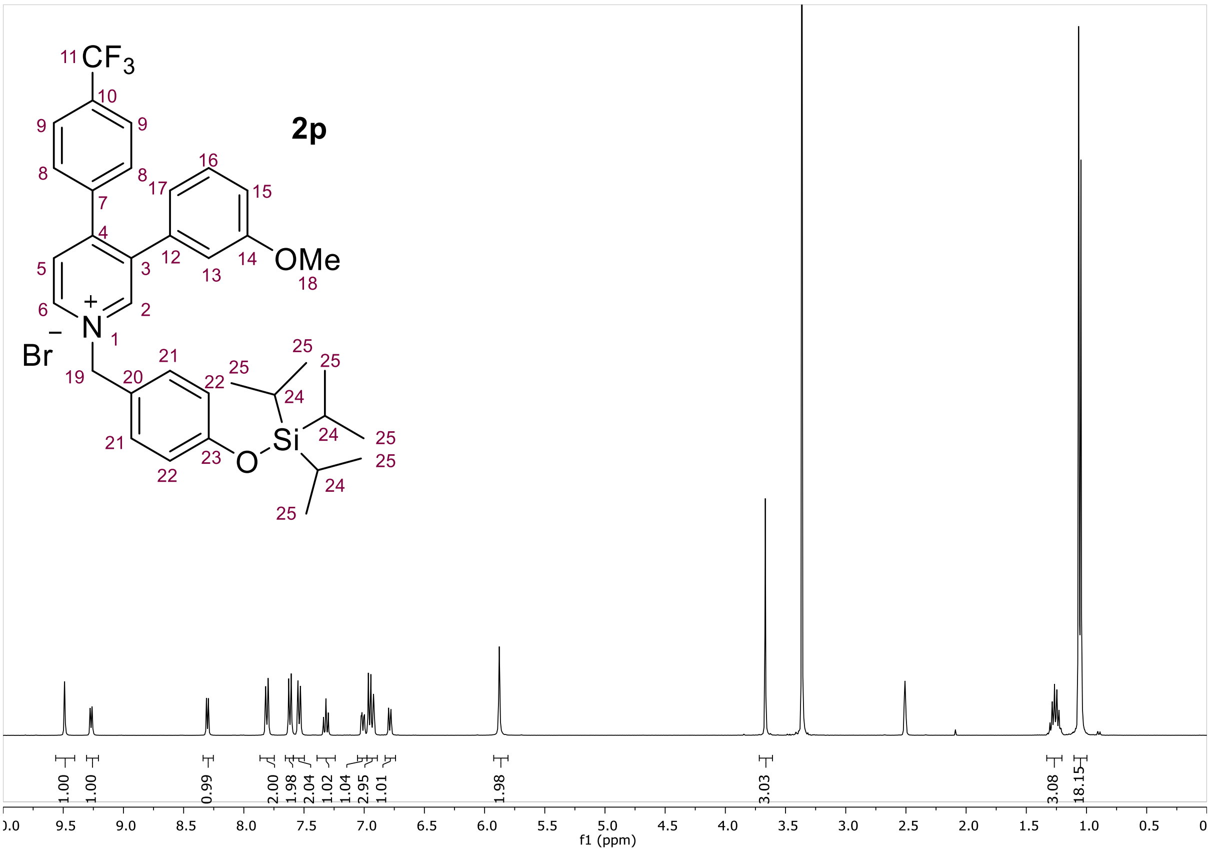
2p
CF3
OMe
Br
N
Si
O
1.00
1.00
0.99
2.00
1.98
2.04
1.02
1.04
2.95
1.01
1.98
3.03
3.08
18.15
f1 (ppm)

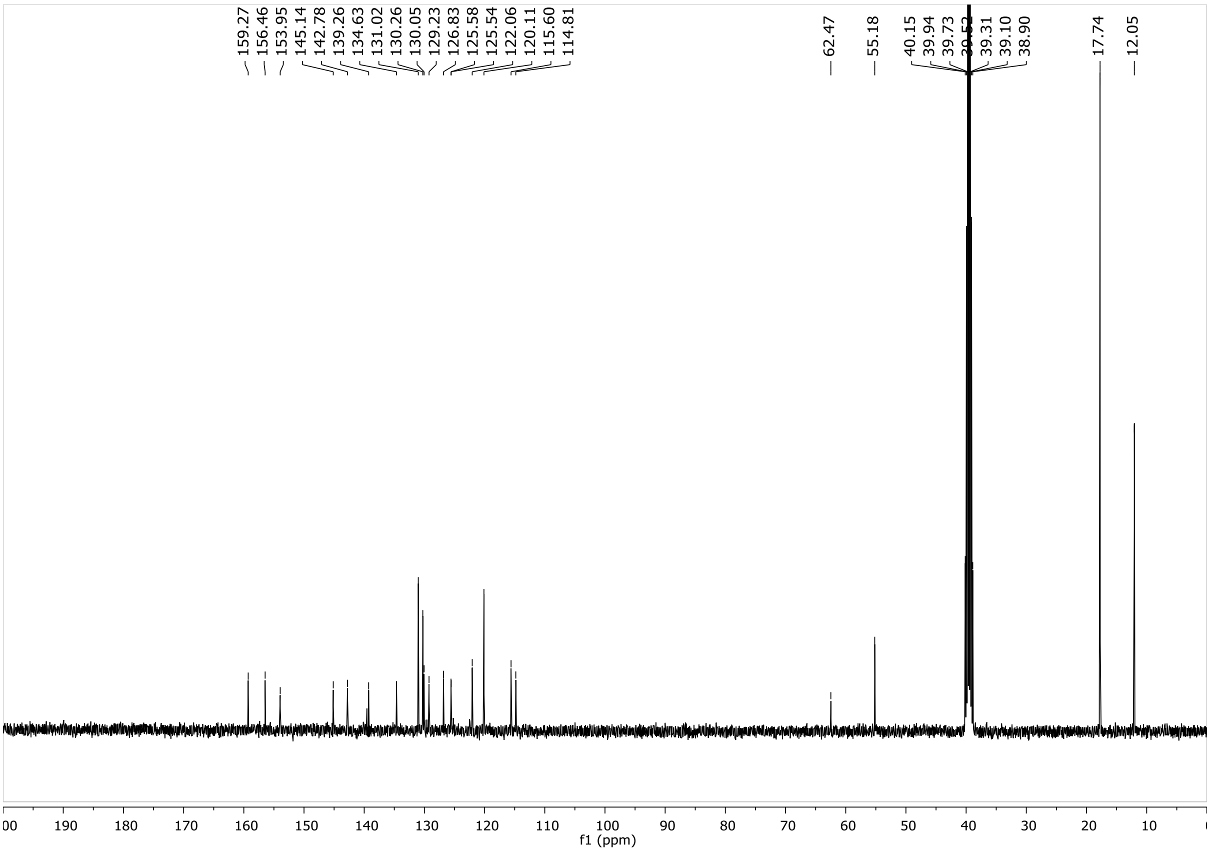
159.27
156.46
153.95
145.14
142.78
139.26
134.63
131.02
130.26
130.05
129.23
126.83
125.58
125.54
122.06
120.11
115.60
114.81
62.47
55.18
40.15
39.94
39.73
39.31
39.10
38.90
17.74
12.05
f1 (ppm)

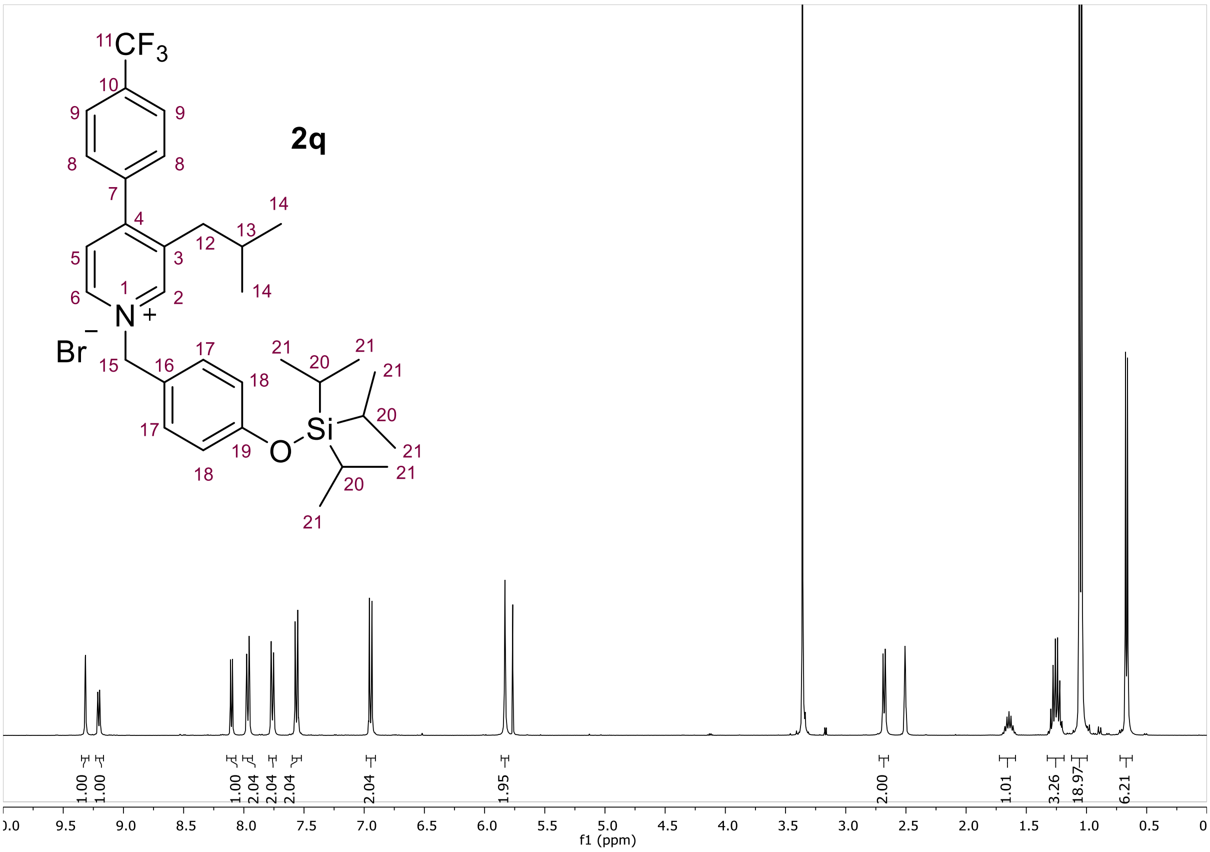
2q
Br
15
16
17
18
19
O
Si
20
21
11CF3
1.00
1.00
2.04
2.04
2.04
2.04
1.95
2.00
1.01
3.26
18.97
6.21
f1 (ppm)

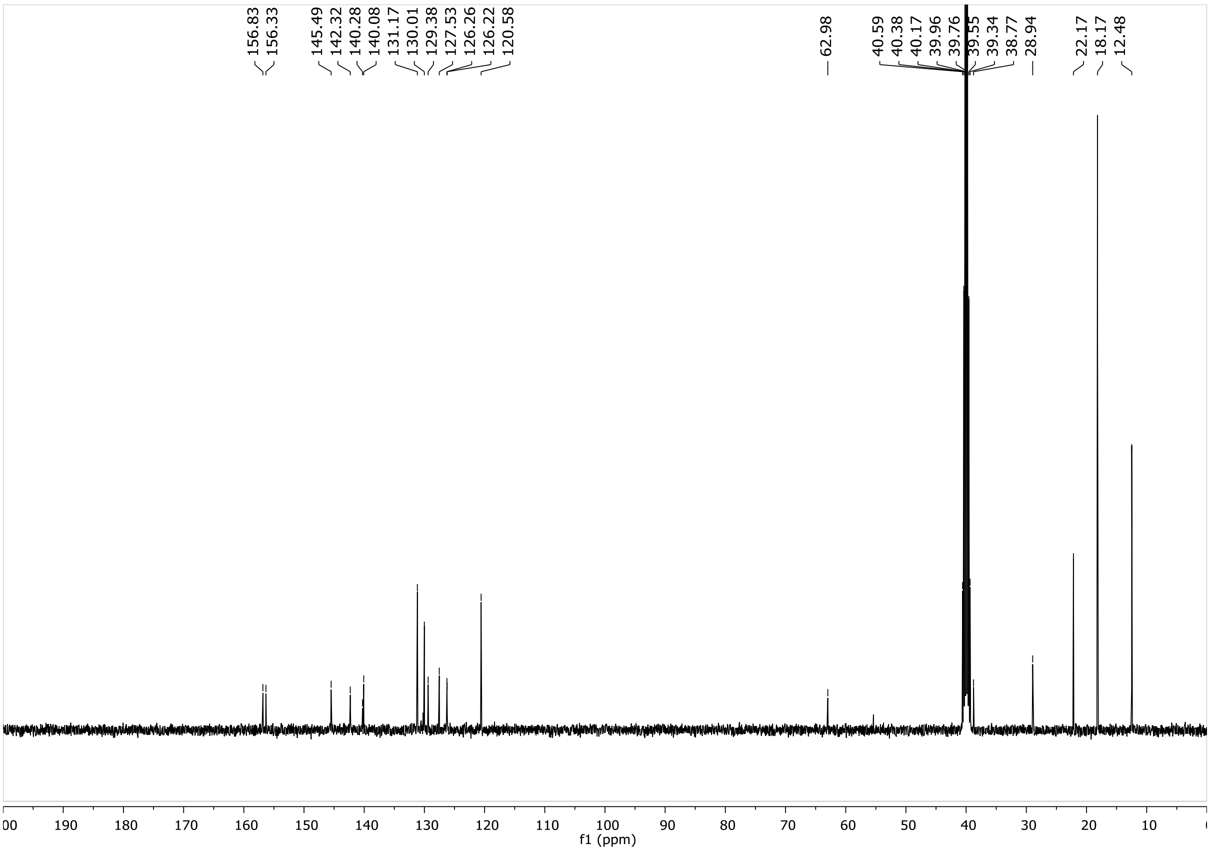
156.83
156.33
145.49
142.32
140.28
140.08
131.17
130.01
129.38
127.53
126.26
126.22
120.58
62.98
40.59
40.38
40.17
39.96
39.76
39.55
39.34
38.77
28.94
22.17
18.17
12.48
f1 (ppm)

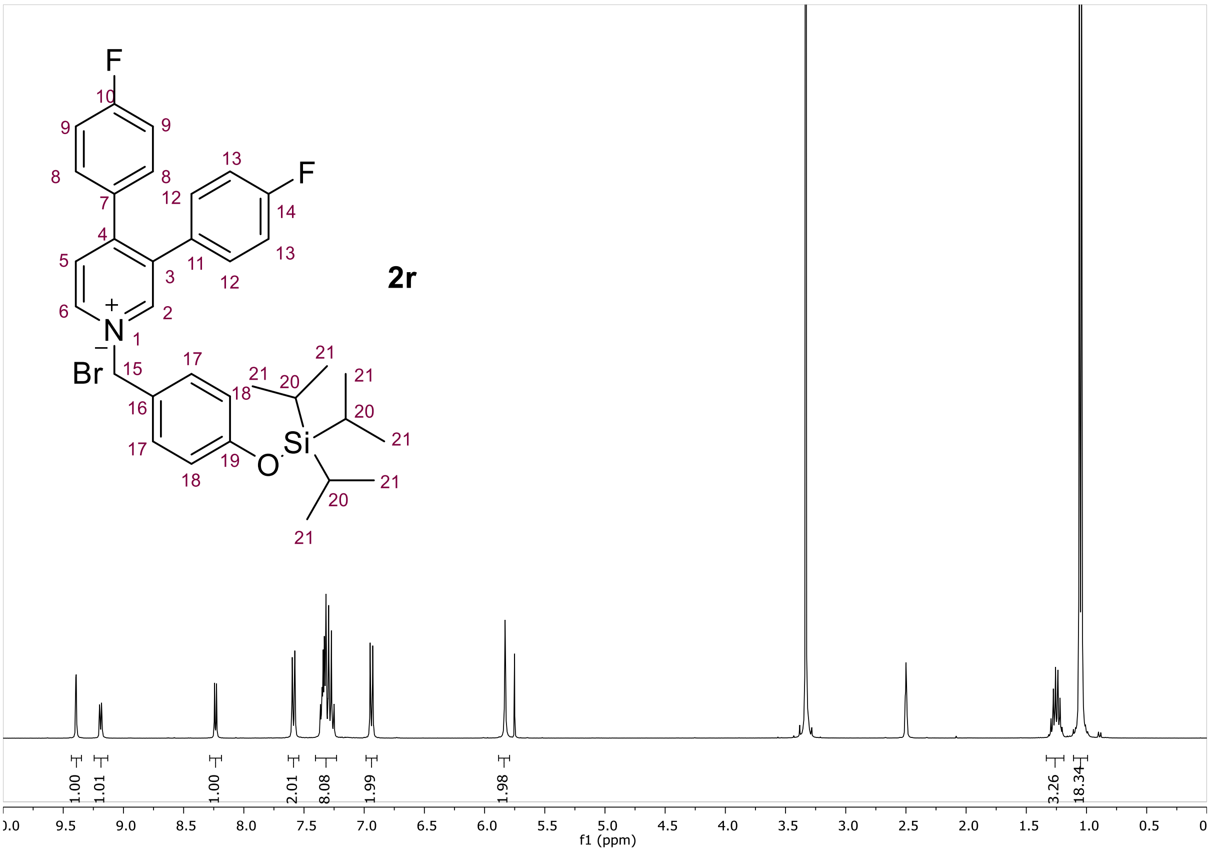
2r
Br
1.00
1.01
1.00
2.01
8.08
1.99
1.98
3.26
18.34
f1 (ppm)

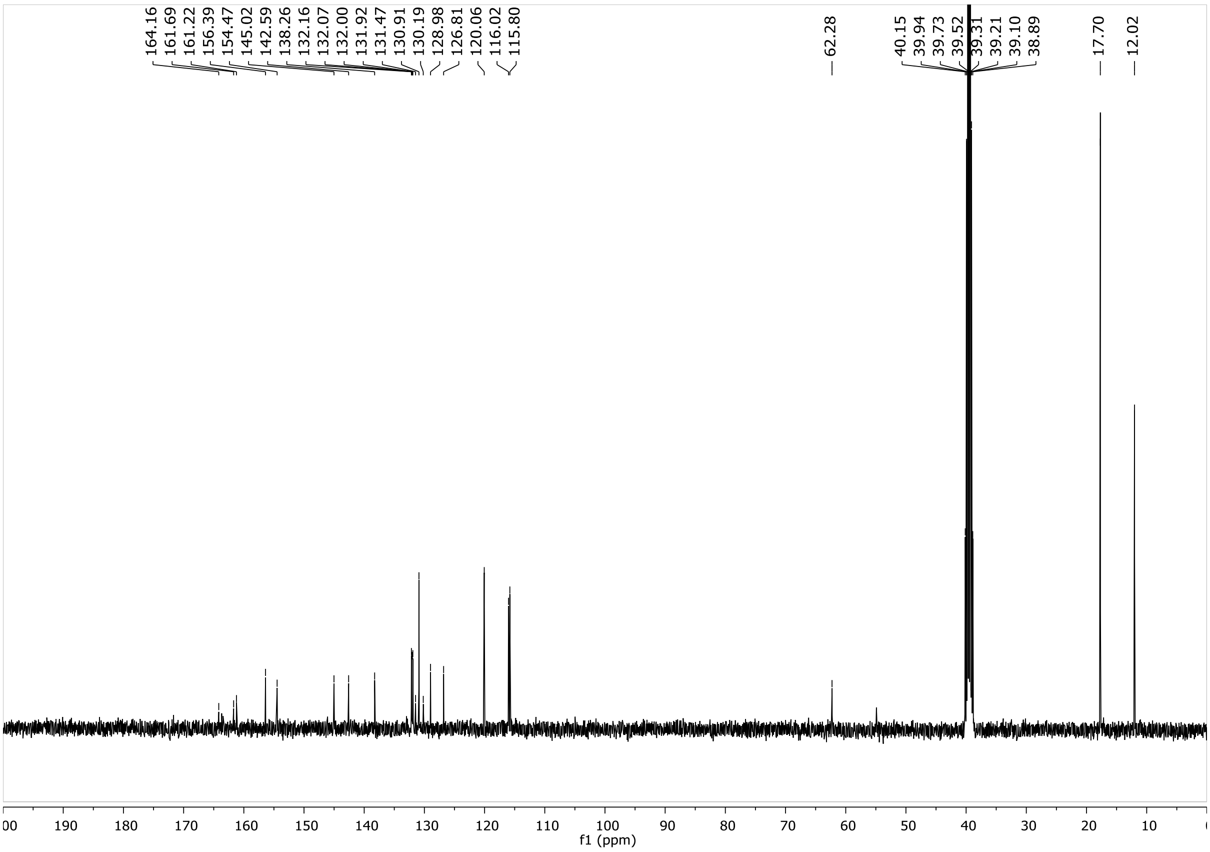
164.16
161.69
161.22
156.39
154.47
145.02
142.59
138.26
132.16
132.07
132.00
131.92
131.47
130.91
130.19
128.98
126.81
120.06
116.02
115.80
62.28
40.15
39.94
39.73
39.52
39.31
39.21
39.10
38.89
17.70
12.02
f1 (ppm)

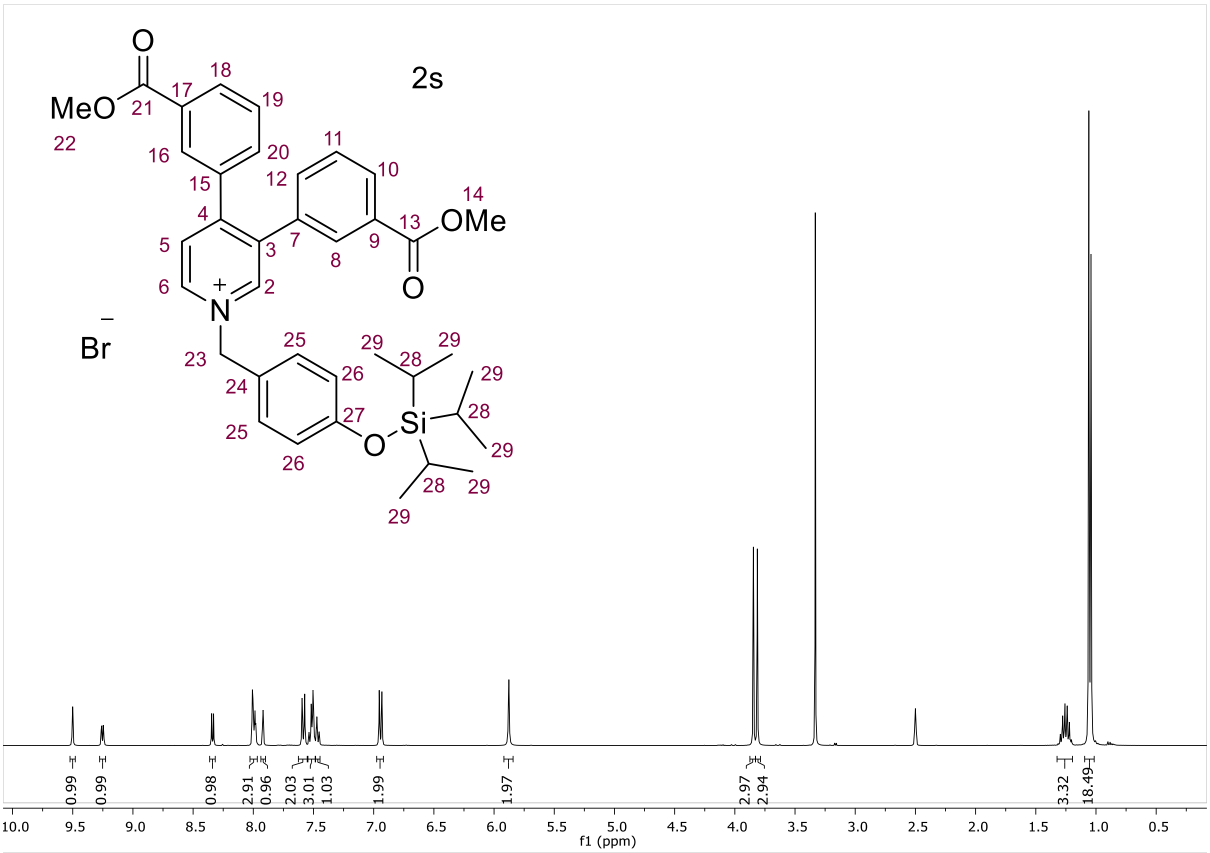
2s
MeO
OMe
Br
Si
0.99
0.99
0.98
2.91
0.96
2.03
3.01
1.03
1.99
1.97
2.97
2.94
3.32
18.49
f1 (ppm)

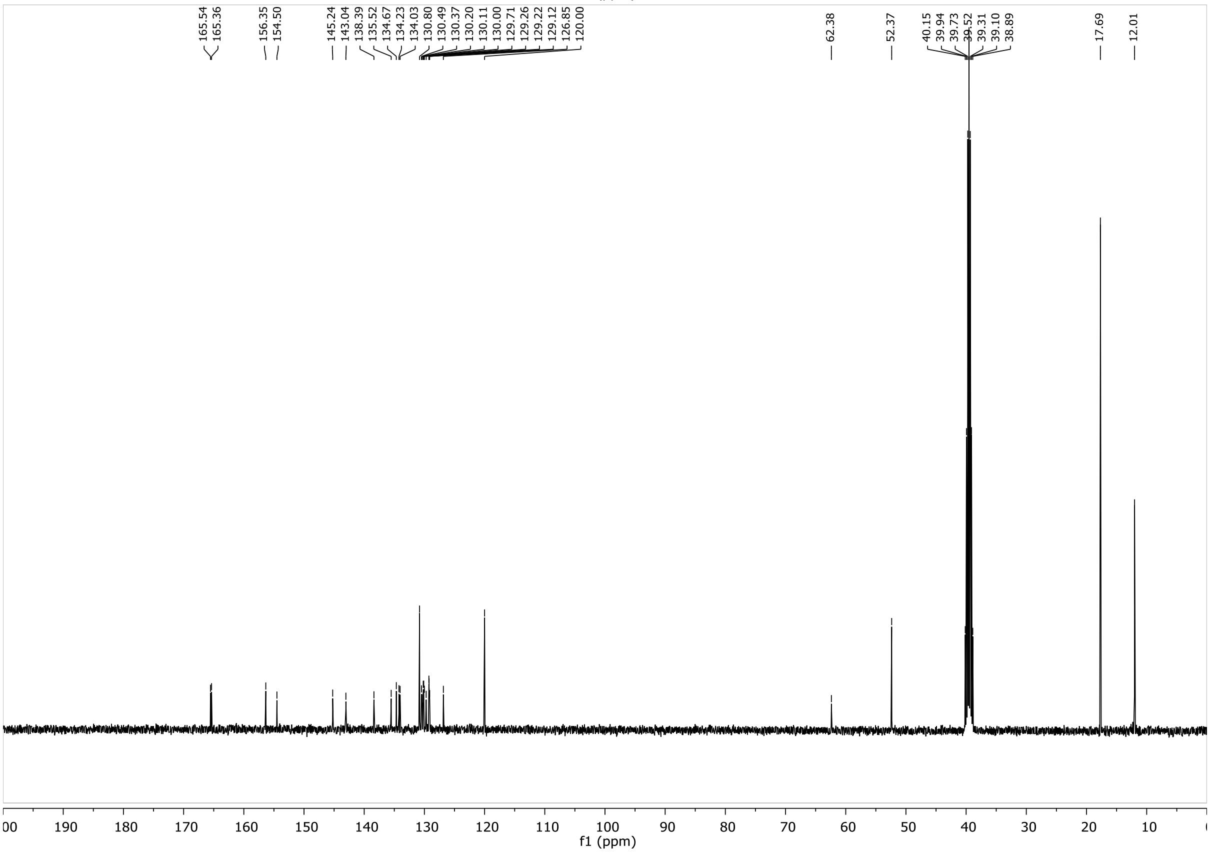
165.54
165.36
156.35
154.50
145.24
143.04
138.39
135.52
134.67
134.23
134.03
130.80
130.49
130.37
130.20
130.11
130.00
129.71
129.26
129.22
129.12
126.85
120.00
62.38
52.37
40.15
39.94
39.73
39.52
39.31
39.10
38.89
17.69
12.01
f1 (ppm)

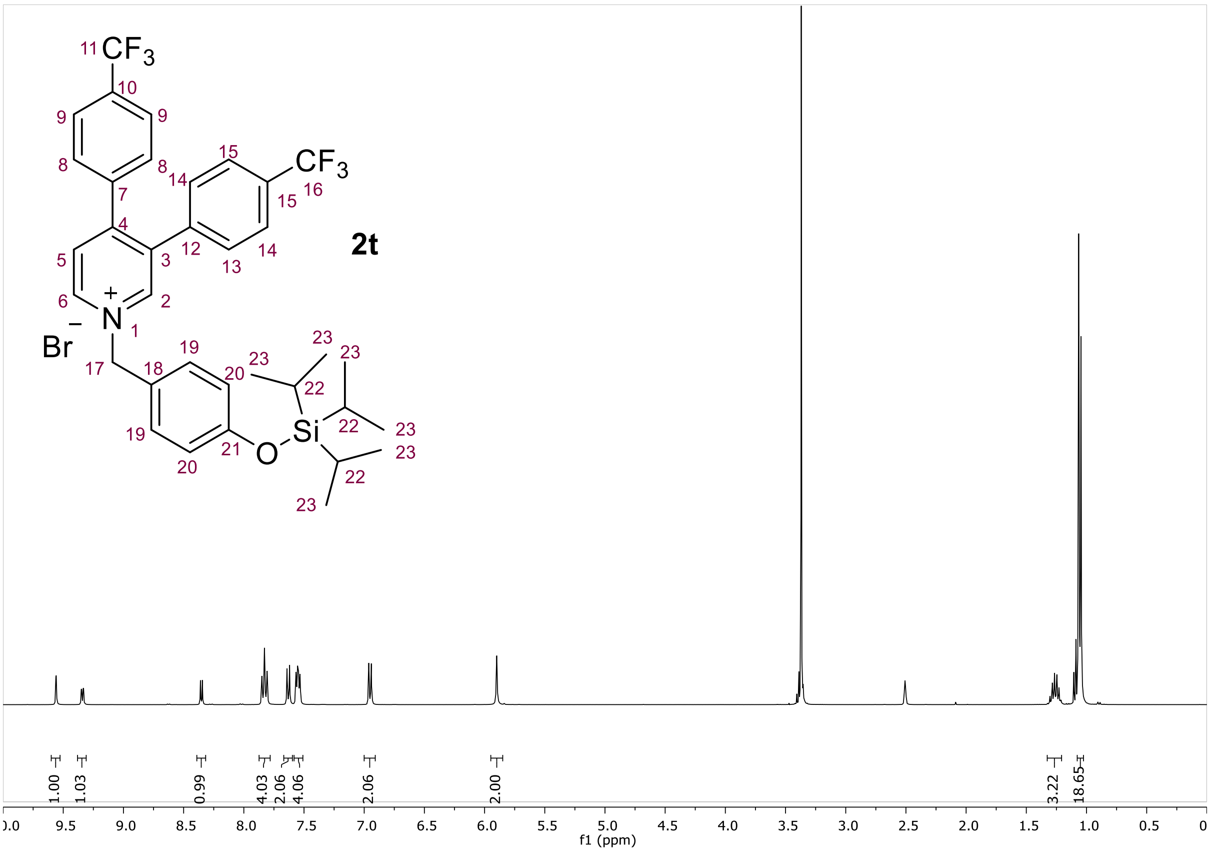

CF3
Br
N
Si
O
2t
1.00
1.03
0.99
4.03
2.06
4.06
2.06
2.00
3.22
18.65
f1 (ppm)

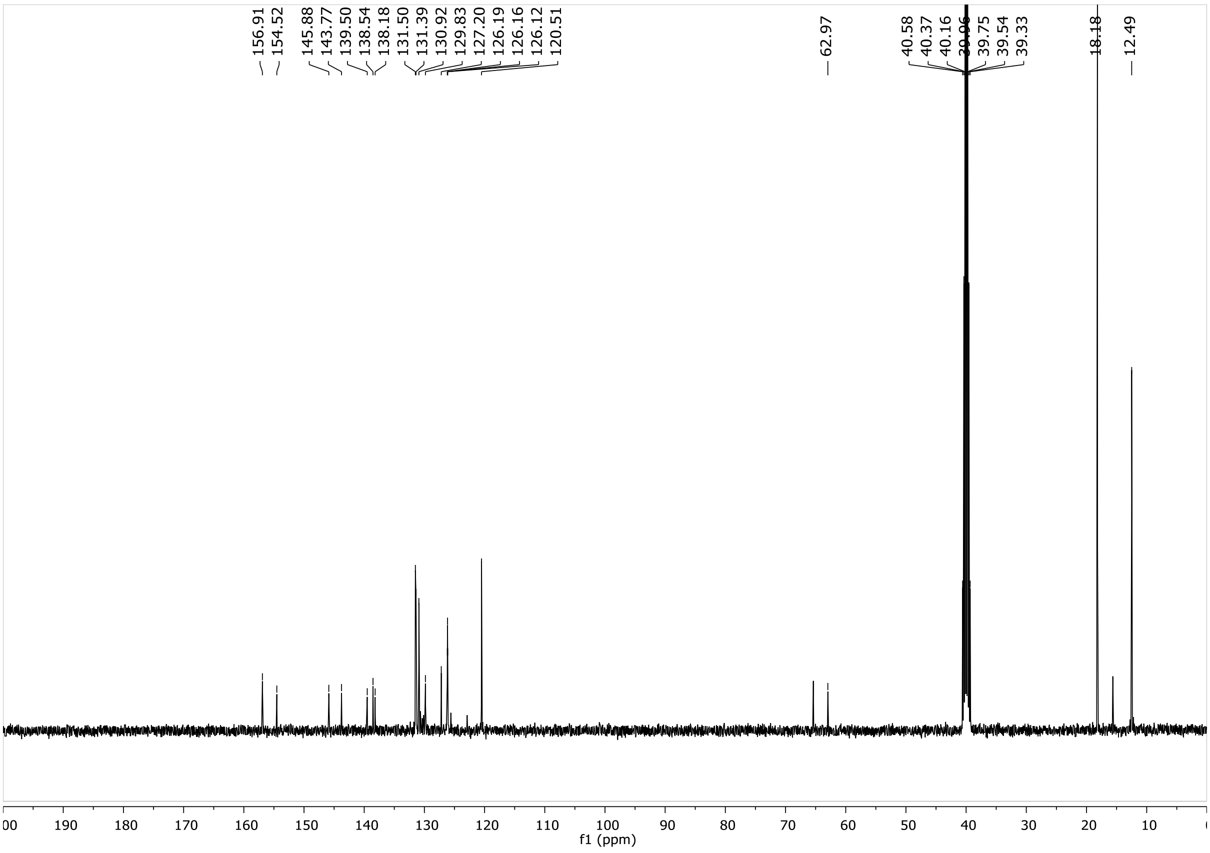

156.91
154.52
145.88
143.77
139.50
138.54
138.18
131.50
131.39
130.92
129.83
127.20
126.19
126.16
126.12
120.51
62.97
40.58
40.37
40.16
39.96
39.75
39.54
39.33
18.18
12.49
f1 (ppm)

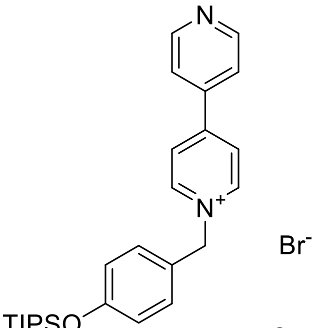
N
N+
Br-
TIPSO

2u

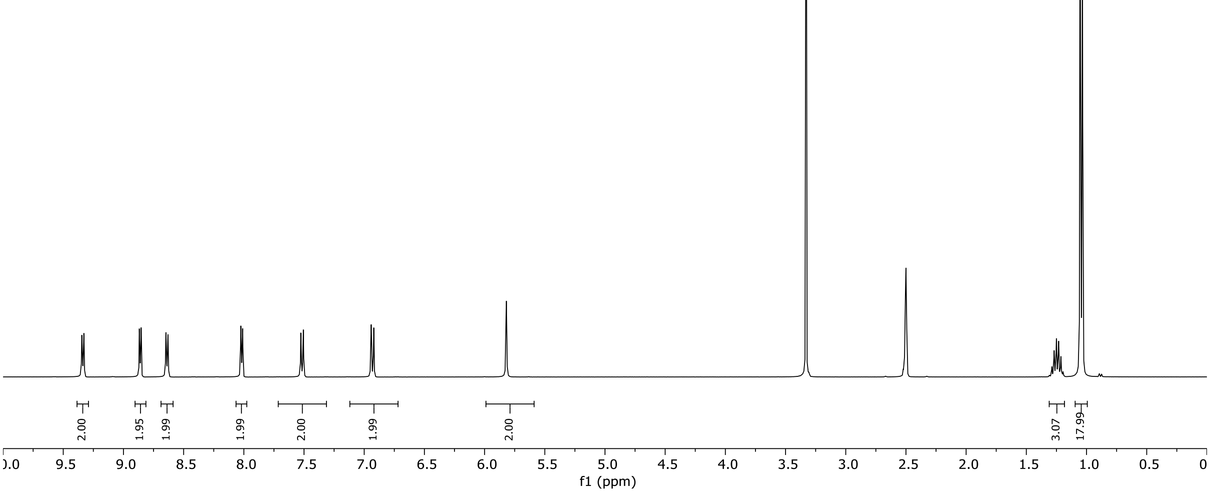
2.00
1.95
1.99
1.99
2.00
1.99
2.00
3.07
17.99
f1 (ppm)

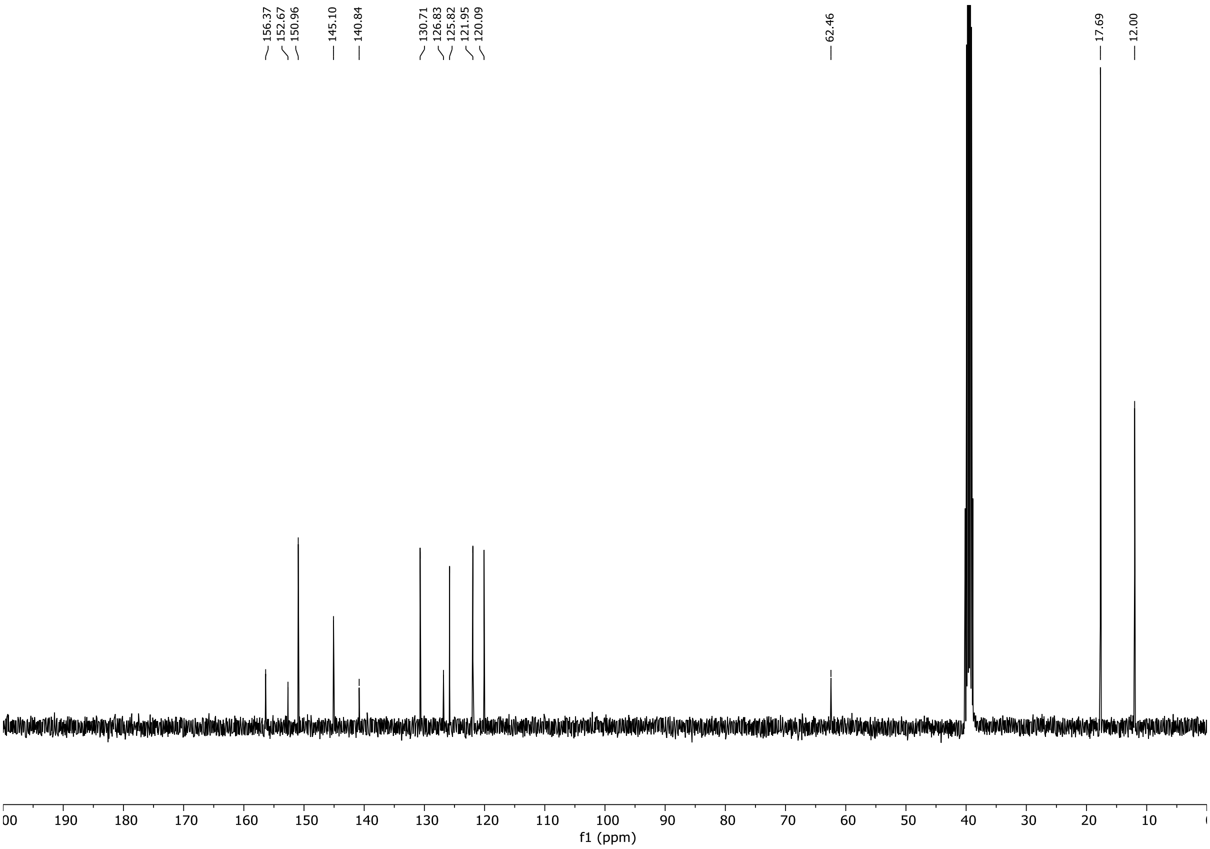
156.37
152.67
150.96
145.10
140.84
130.71
126.83
125.82
121.95
120.09
62.46
17.69
12.00
f1 (ppm)

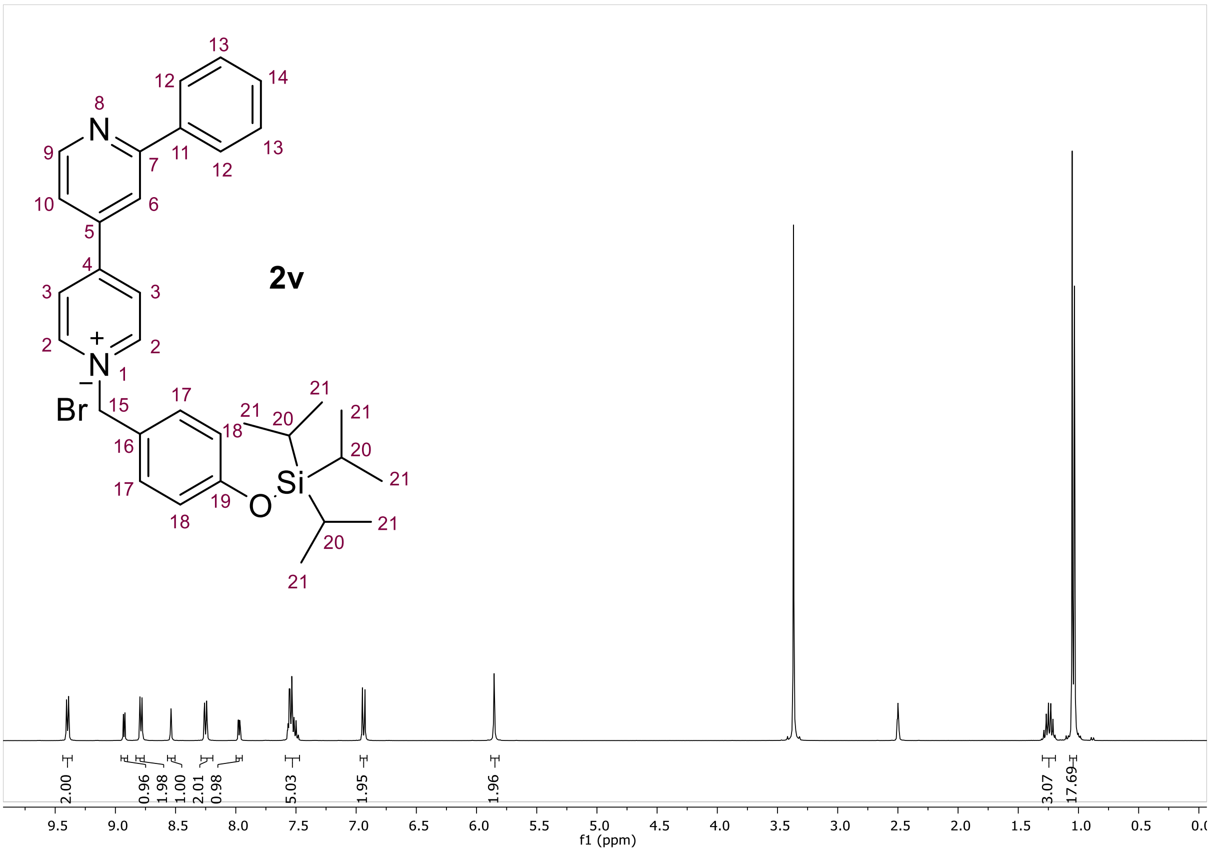
2v
Br
2.00
0.96
1.98
1.00
2.01
0.98
5.03
1.95
1.96
3.07
17.69
9.5 9.0 8.5 8.0 7.5 7.0 6.5 6.0 5.5 5.0 4.5 4.0 3.5 3.0 2.5 2.0 1.5 1.0 0.5
f1 (ppm)

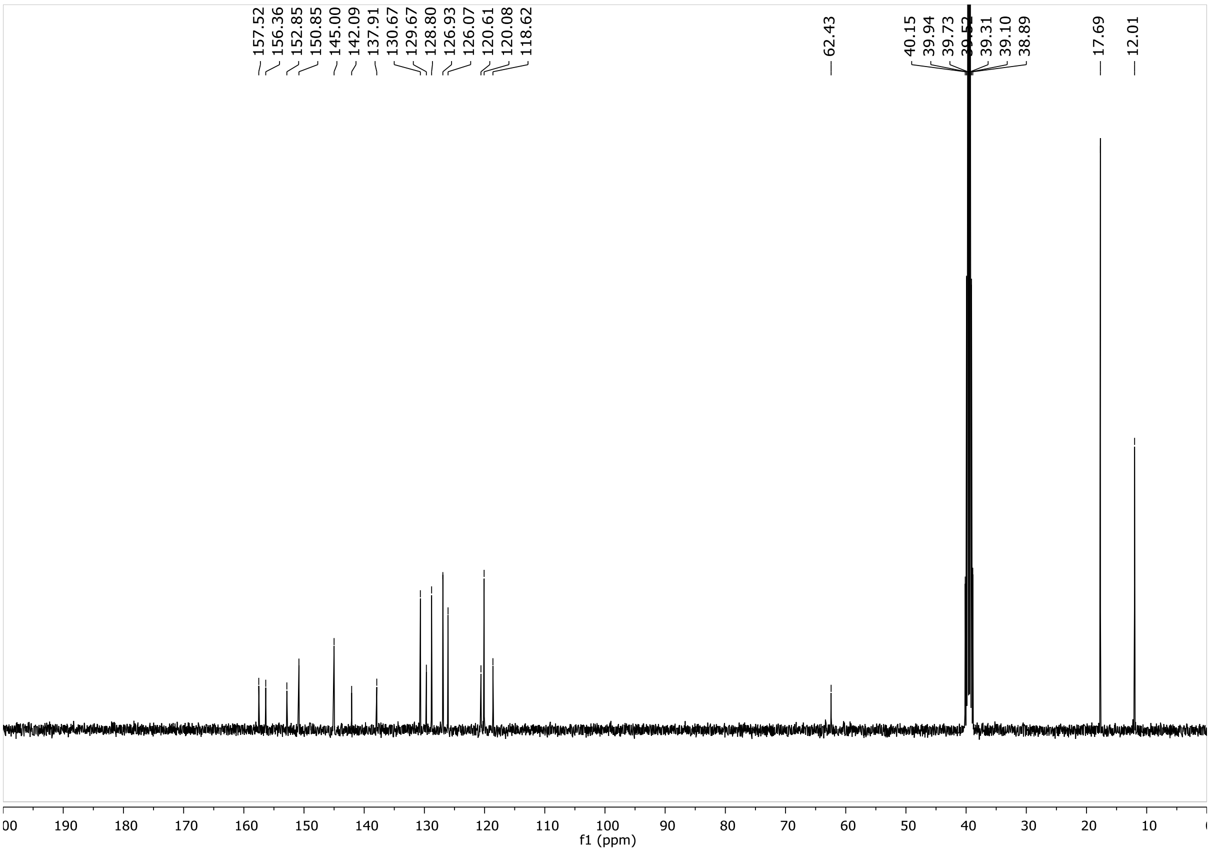
157.52
156.36
152.85
150.85
145.00
142.09
137.91
130.67
129.67
128.80
126.93
126.07
120.61
120.08
118.62
62.43
40.15
39.94
39.73
39.31
39.10
38.89
17.69
12.01
190 180 170 160 150 140 130 120 110 100 90 80 70 60 50 40 30 20 10
f1 (ppm)

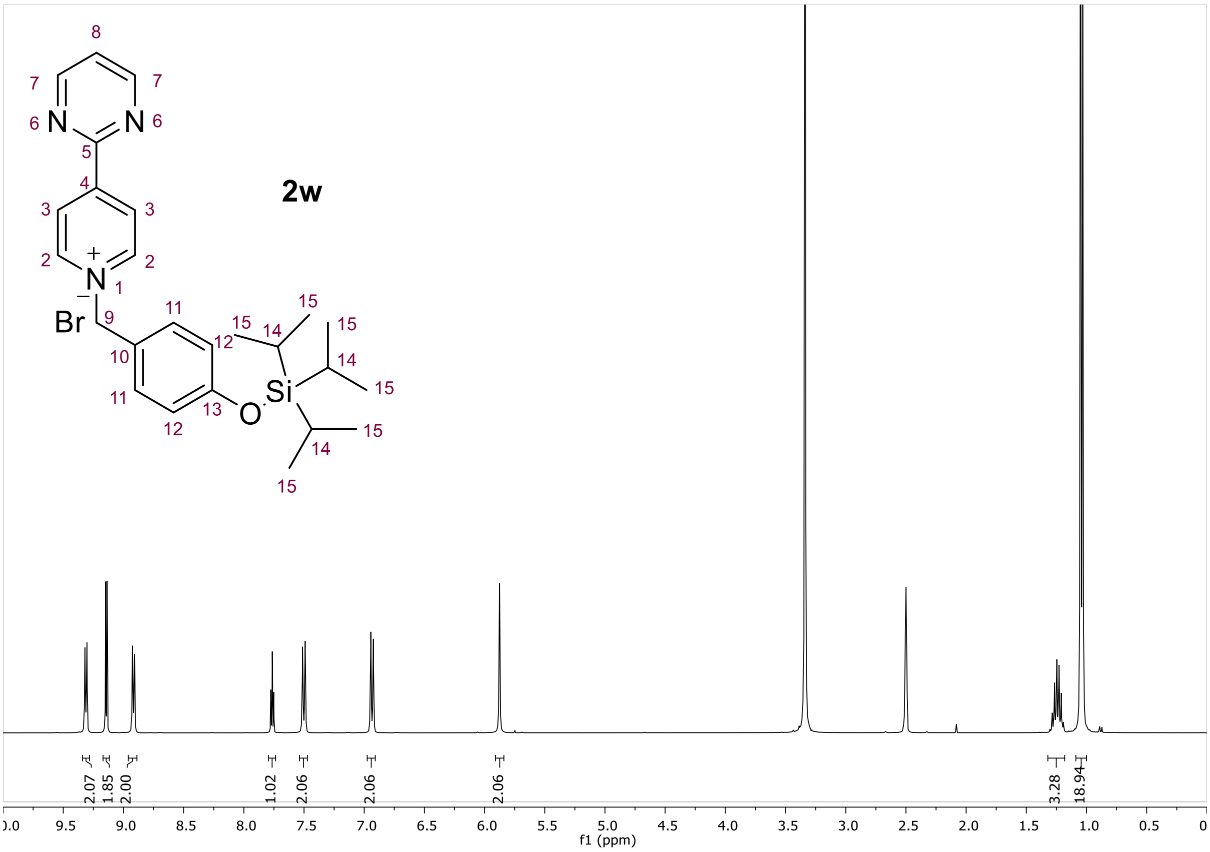
2w
Br
2.07
1.85
2.00
1.02
2.06
2.06
2.06
3.28
18.94
f1 (ppm)

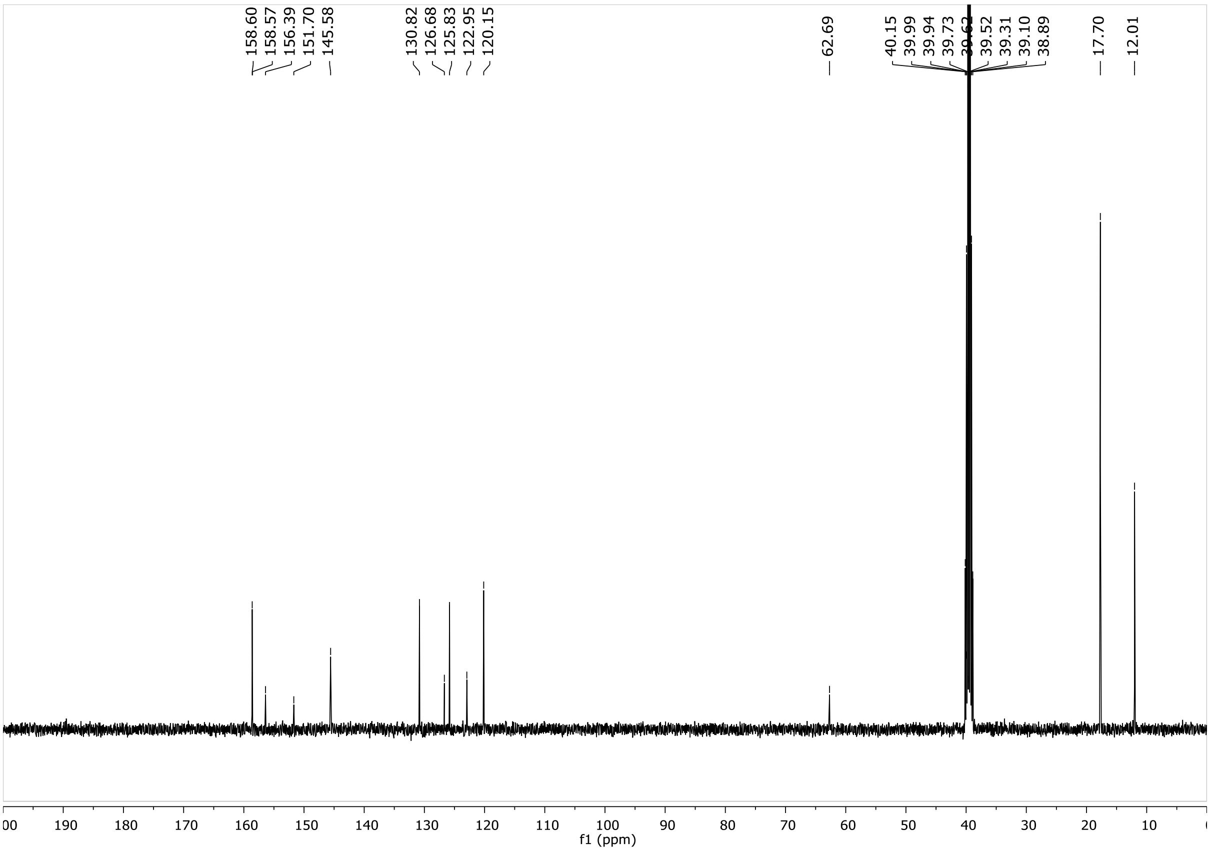
158.60
158.57
156.39
151.70
145.58
130.82
126.68
125.83
122.95
120.15
62.69
40.15
39.99
39.94
39.73
39.52
39.31
39.10
38.89
17.70
12.01
f1 (ppm)

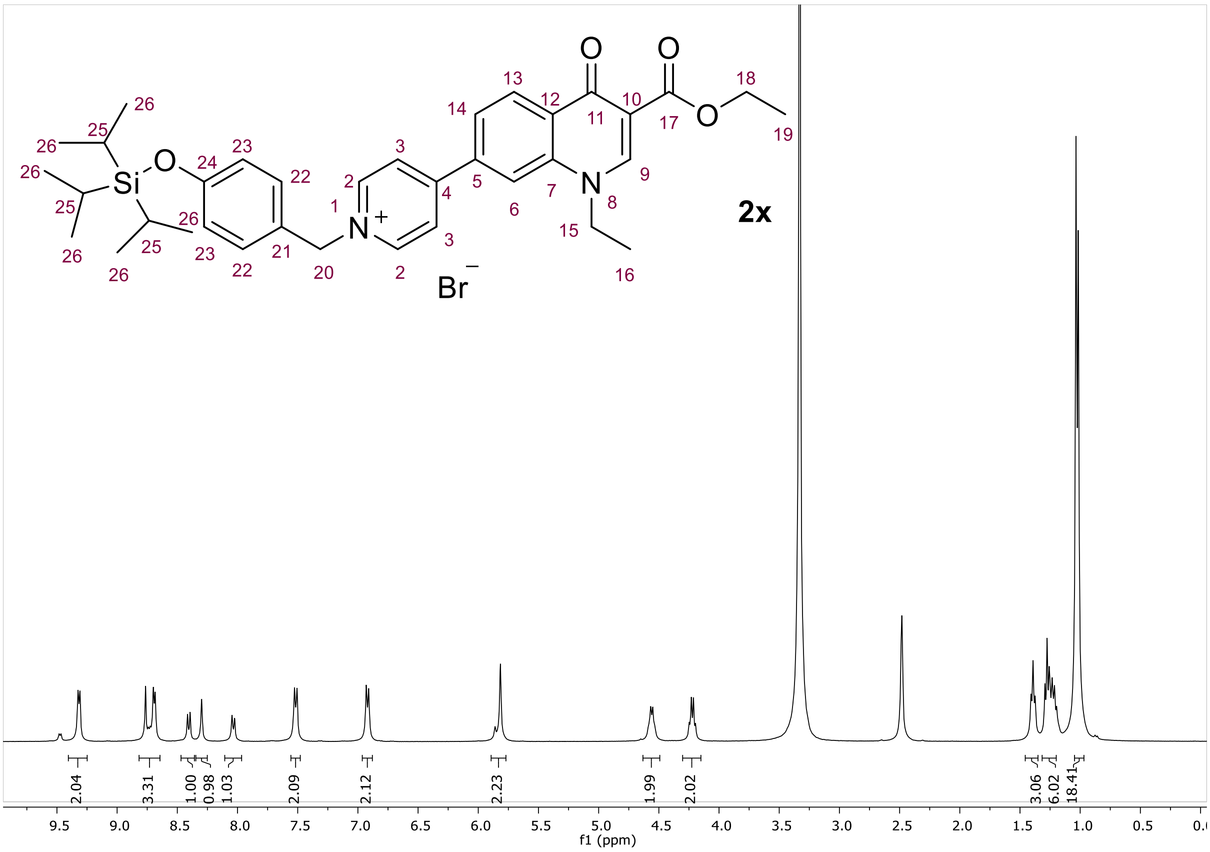
2x
Si
O
N+
Br−
2.04
3.31
1.00
0.98
1.03
2.09
2.12
2.23
1.99
2.02
3.06
6.02
18.41
f1 (ppm)
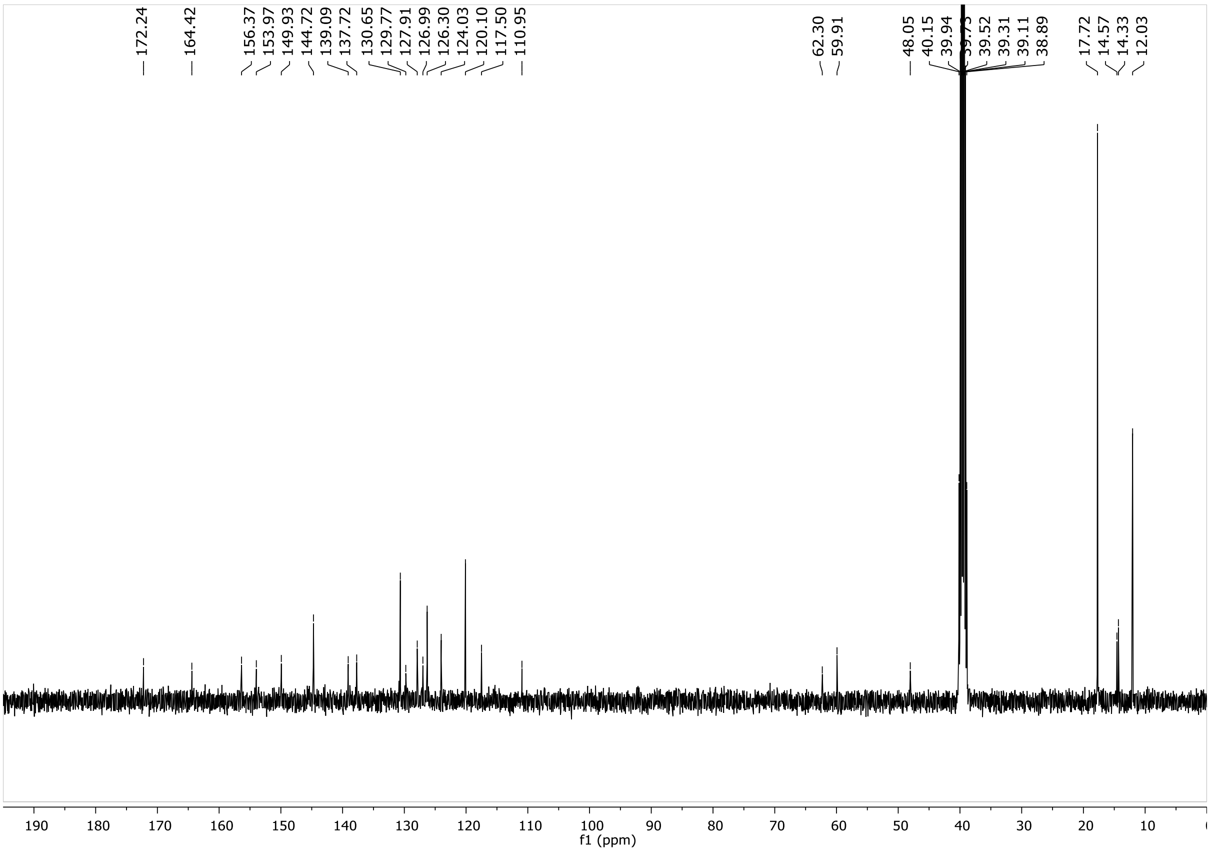
172.24
164.42
156.37
153.97
149.93
144.72
139.09
137.72
130.65
129.77
127.91
126.99
126.30
124.03
120.10
117.50
110.95
62.30
59.91
48.05
40.15
39.94
39.52
39.31
39.11
38.89
17.72
14.57
14.33
12.03
f1 (ppm)

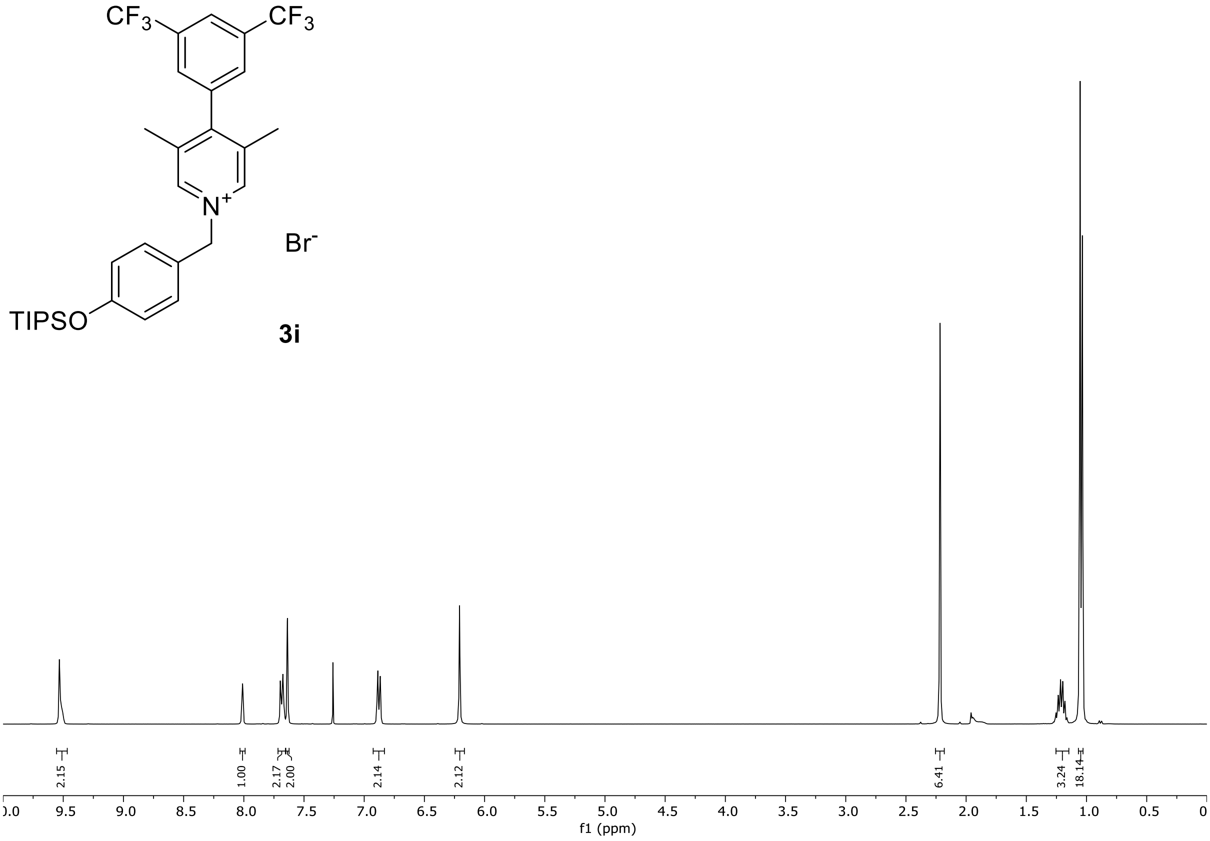
CF3
CF3
N+
Br-
TIPSO
3i
2.15
1.00
2.17
2.00
2.14
2.12
6.41
3.24
18.14
f1 (ppm)

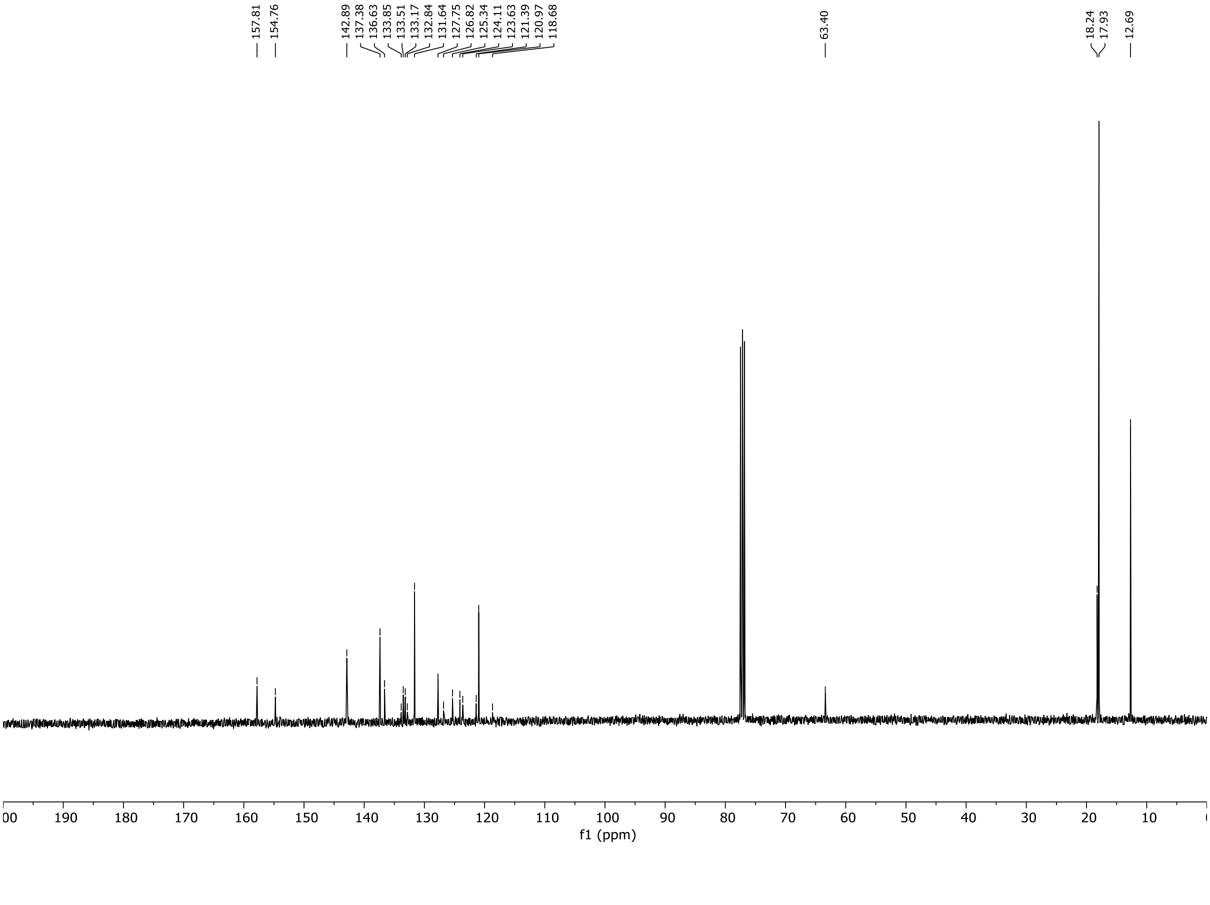
157.81
154.76
142.89
137.38
136.63
133.85
133.51
133.17
132.84
131.64
127.75
126.82
125.34
124.11
123.63
121.39
120.97
118.68
63.40
18.24
17.93
12.69
f1 (ppm)

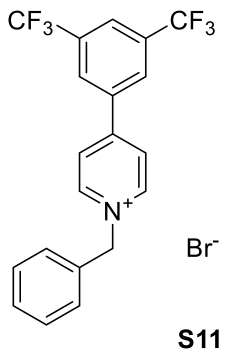
CF3
CF3
N+
Br-

S11

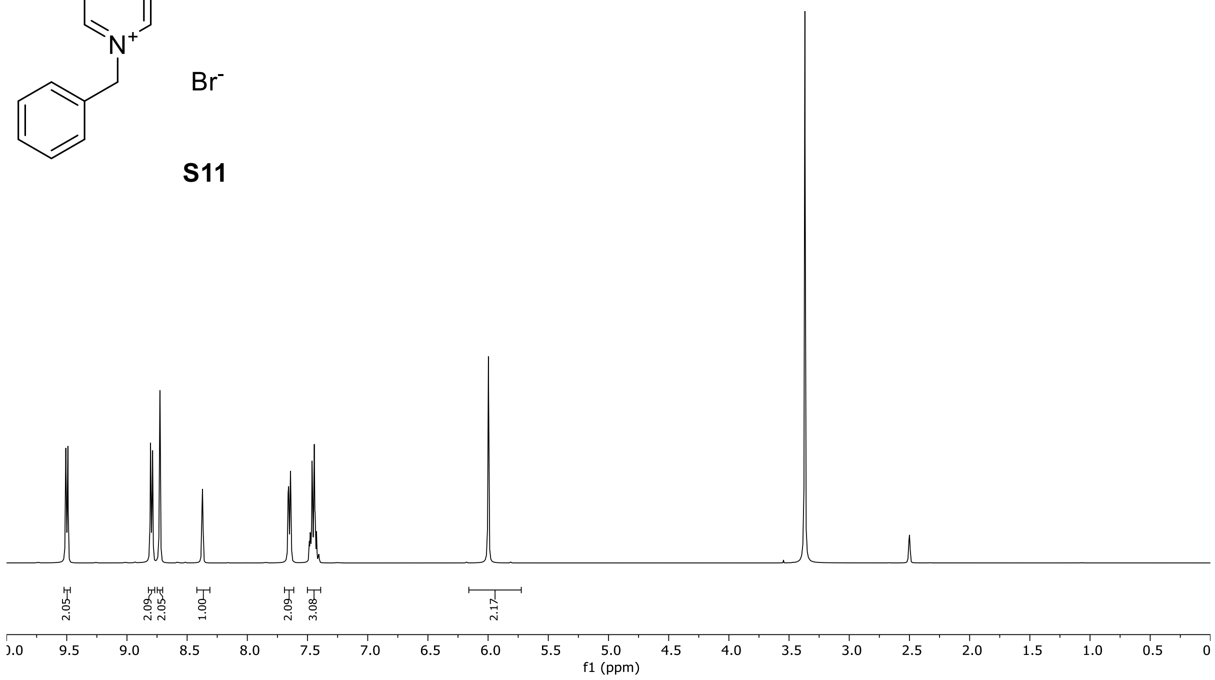
2.05
2.09
2.05
1.00
2.09
3.08
2.17
f1 (ppm)

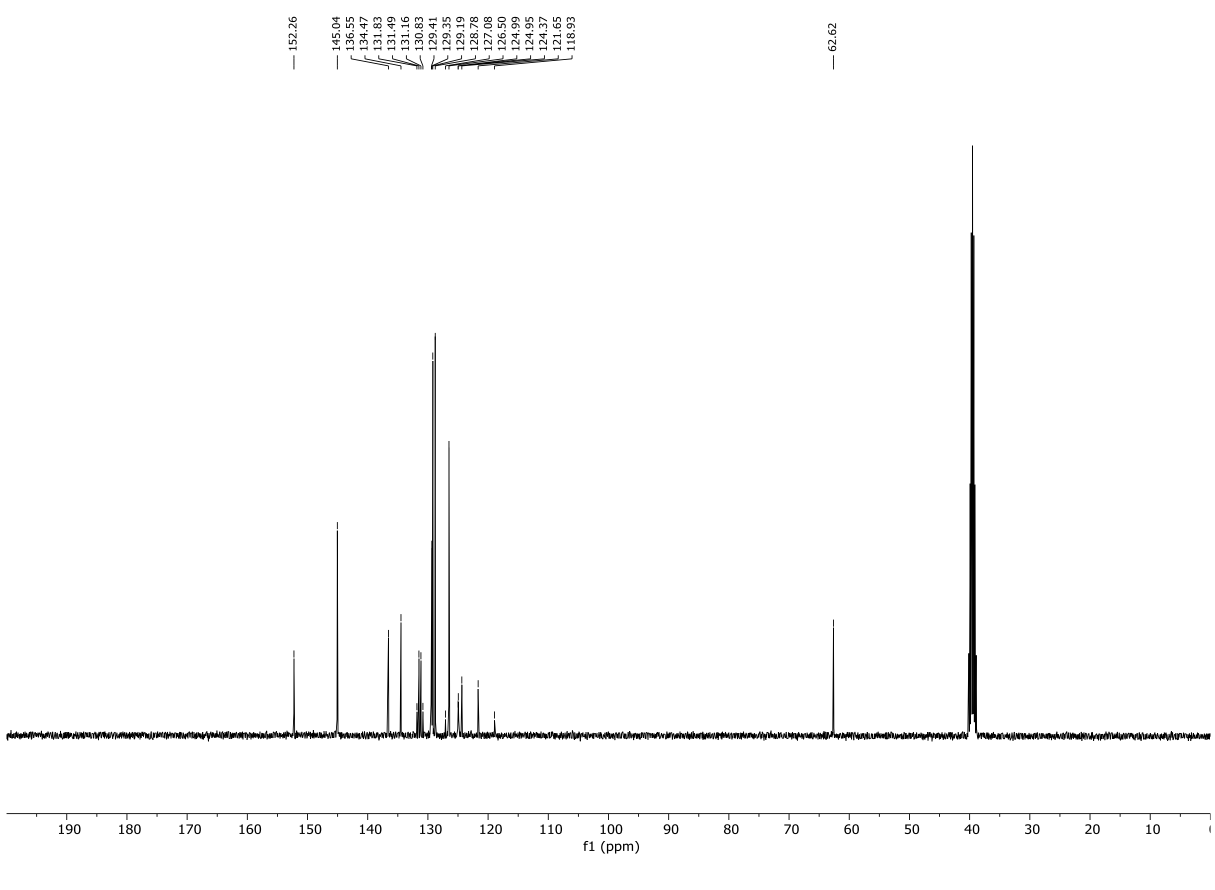
152.26
145.04
136.55
134.47
131.83
131.49
131.16
130.83
129.41
129.35
129.19
128.78
127.08
126.50
124.99
124.95
124.37
121.65
118.93
62.62
f1 (ppm)

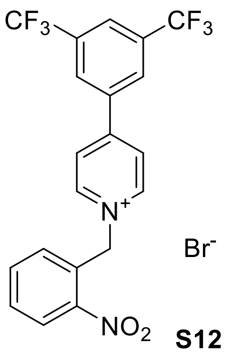
CF3
CF3
N+
Br-
NO2

S12

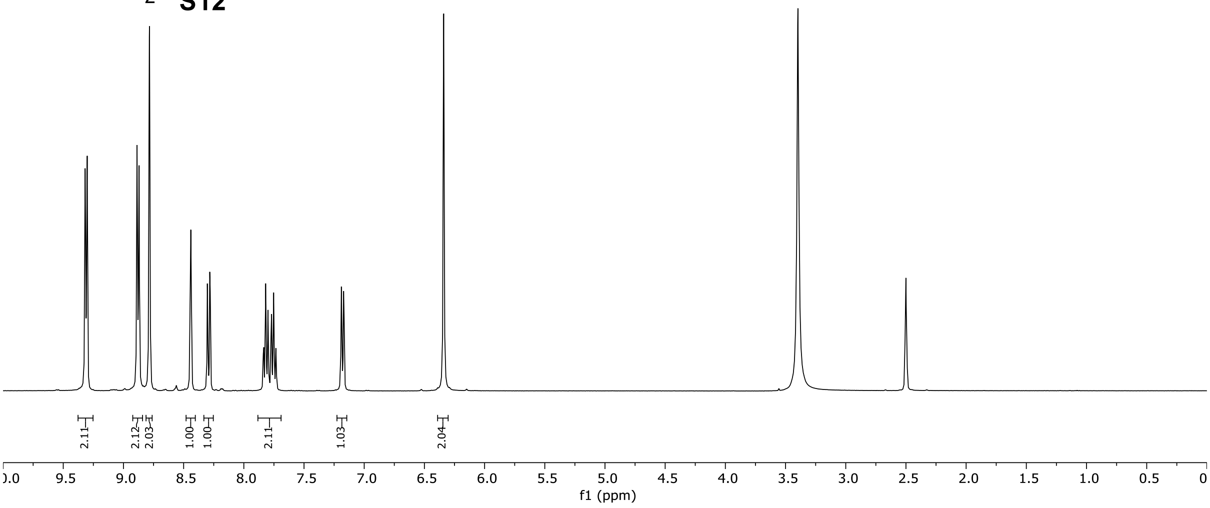
2.11
2.12
2.03
1.00
1.00
2.11
1.03
2.04
f1 (ppm)

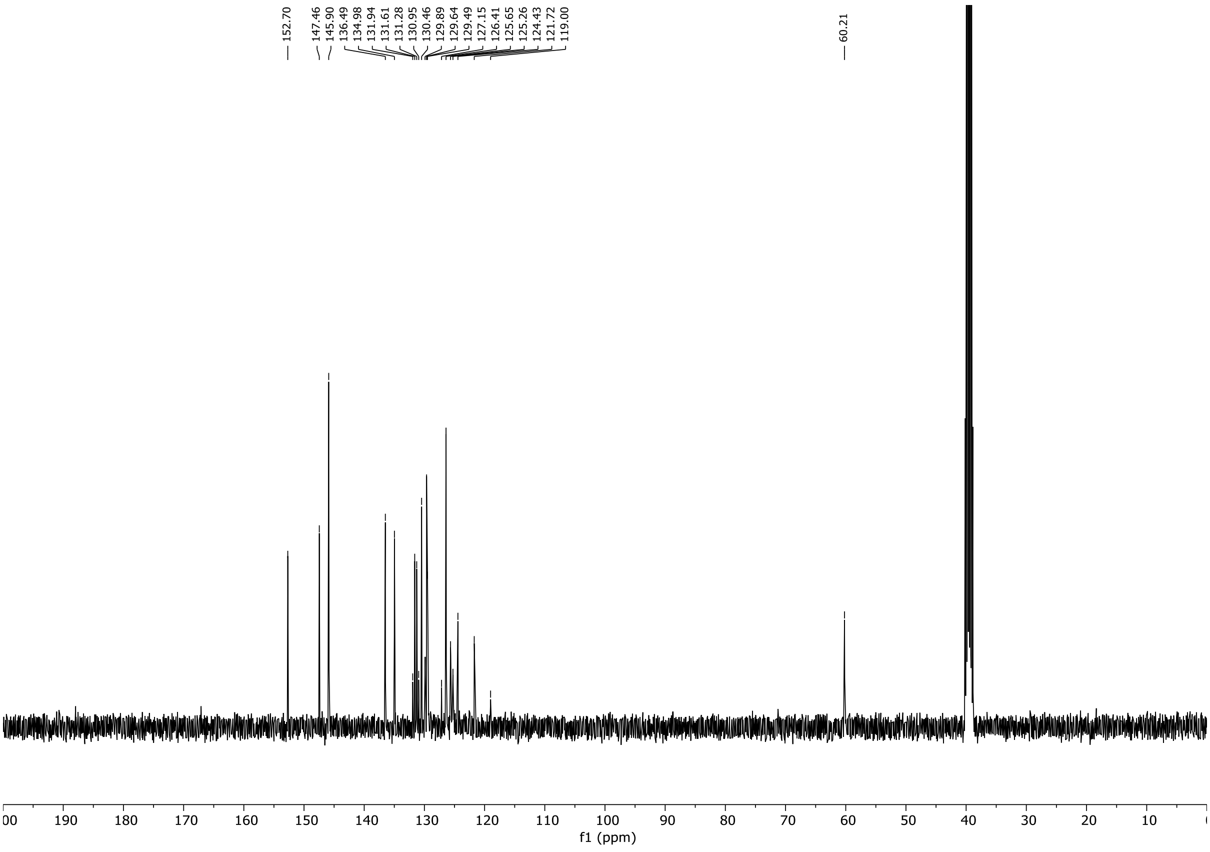
152.70
147.46
145.90
136.49
134.98
131.94
131.61
131.28
130.95
130.46
129.89
129.64
129.49
127.15
126.41
125.65
125.26
124.43
121.72
119.00
60.21
f1 (ppm)

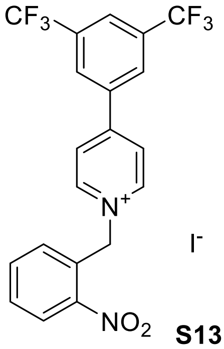

CF3
CF3
N+
I-
NO2

S13

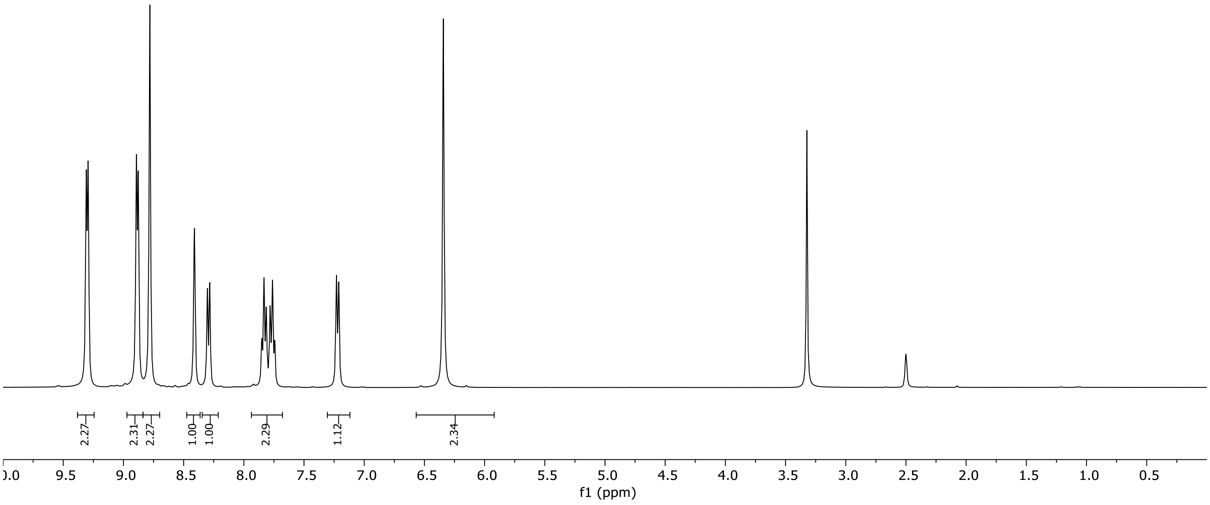

2.27
2.31
2.27
1.00
1.00
2.29
1.12
2.34
f1 (ppm)

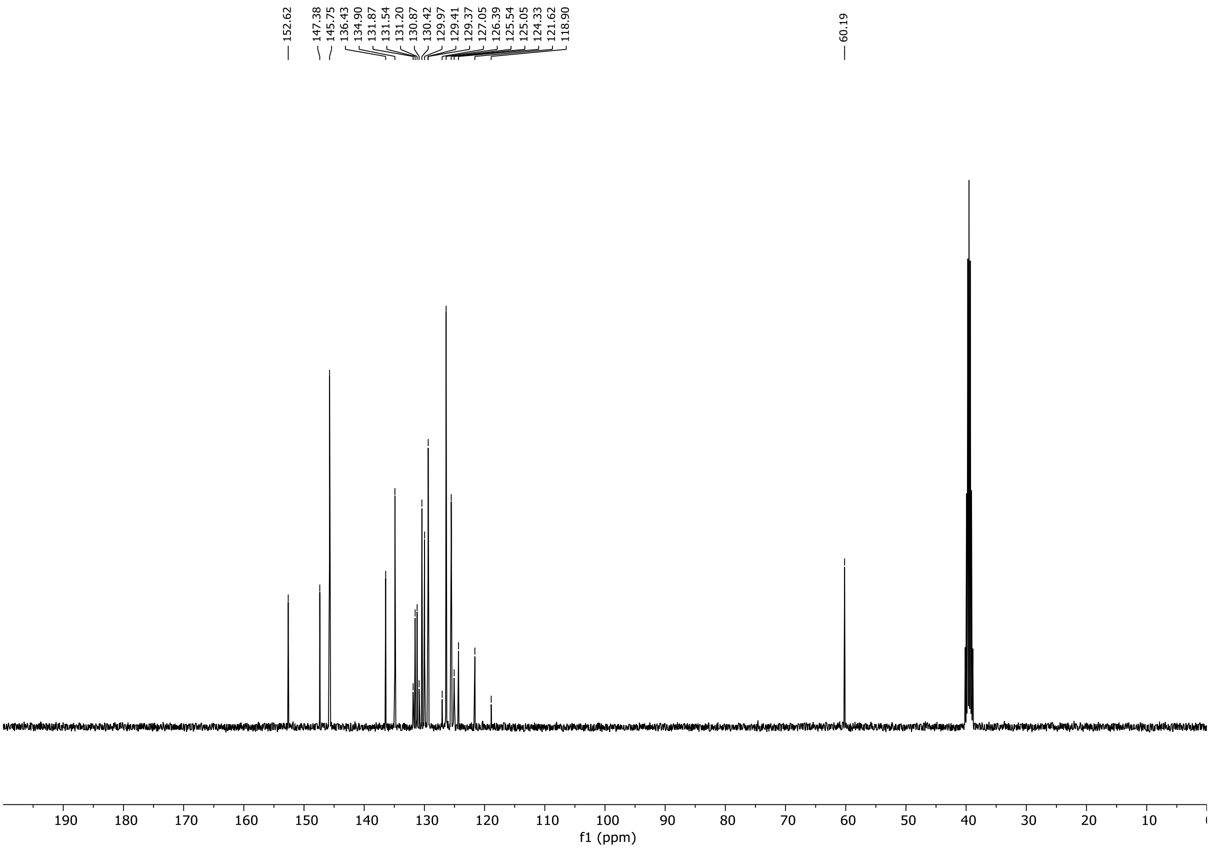

152.62
147.38
145.75
136.43
134.90
131.87
131.54
131.20
130.87
130.42
129.97
129.41
129.37
127.05
126.39
125.54
125.05
124.33
121.62
118.90
60.19
f1 (ppm)

Methylated Products

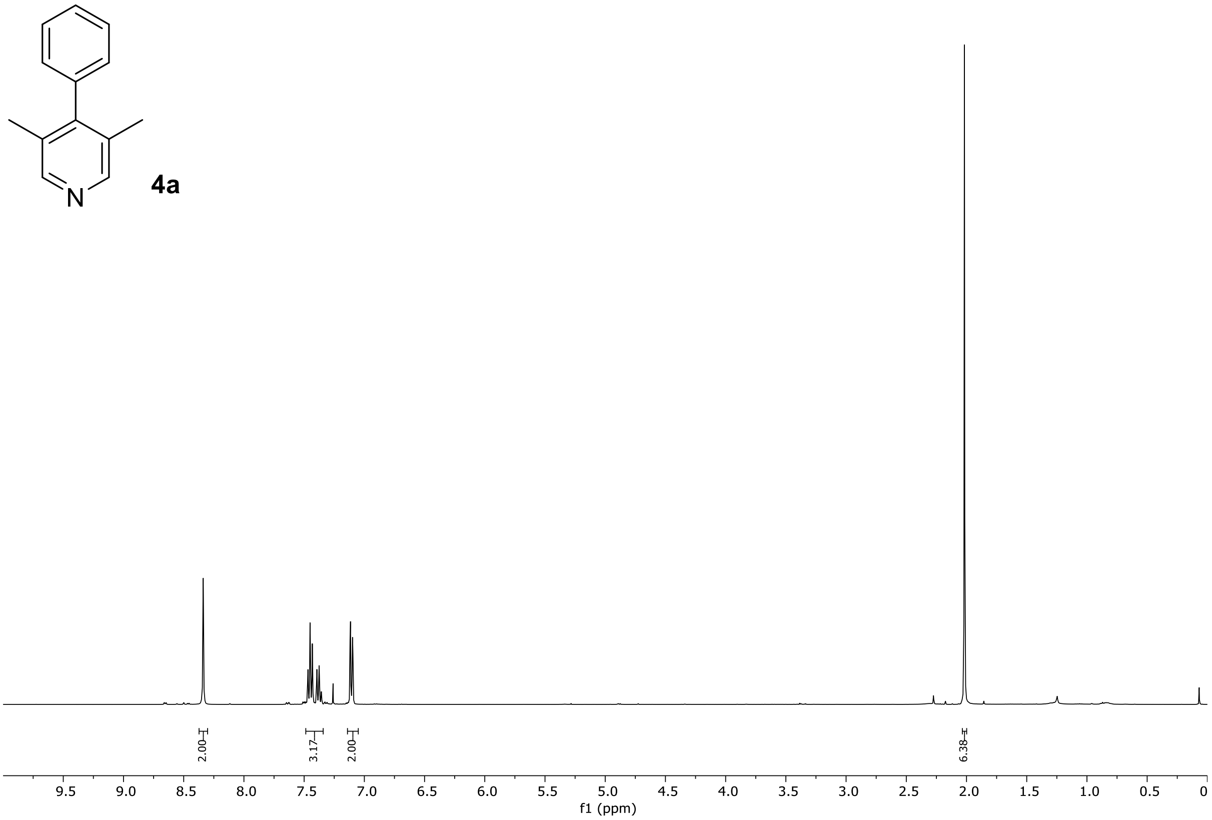

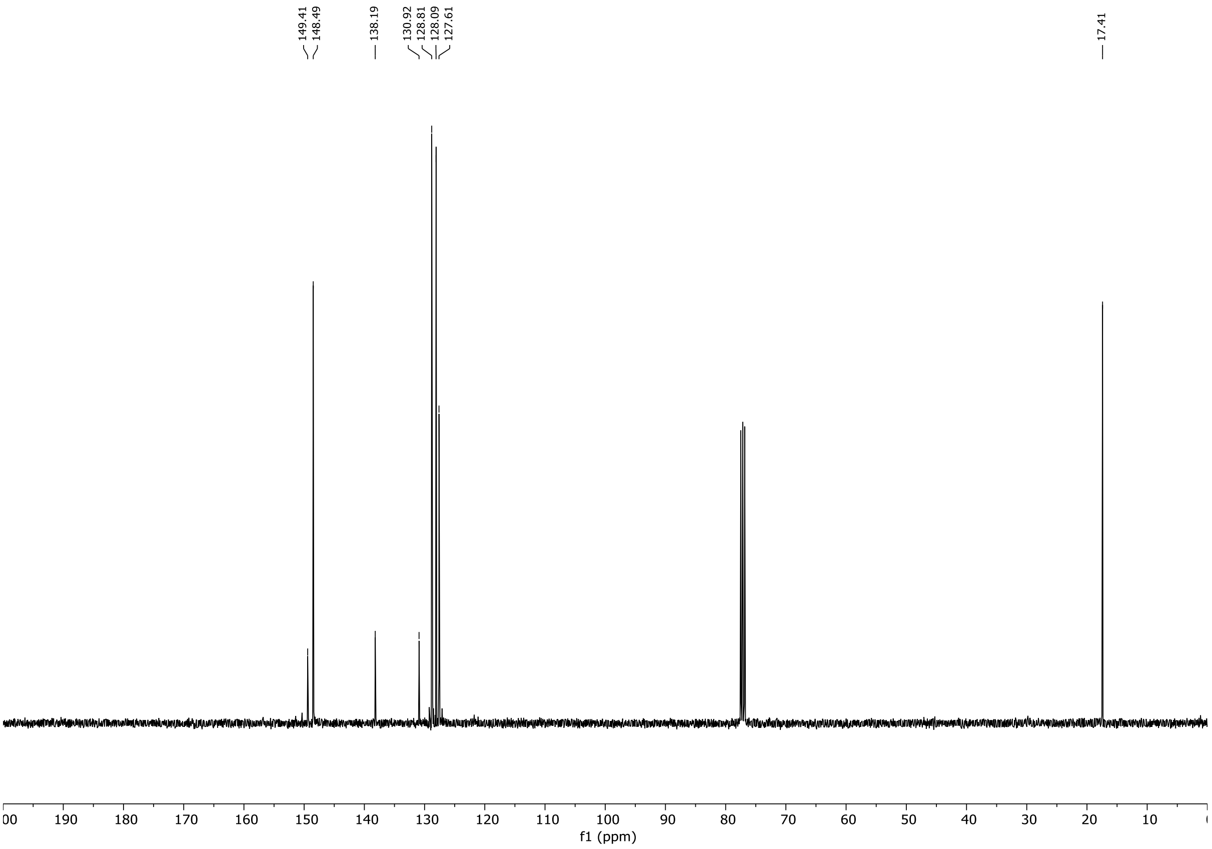

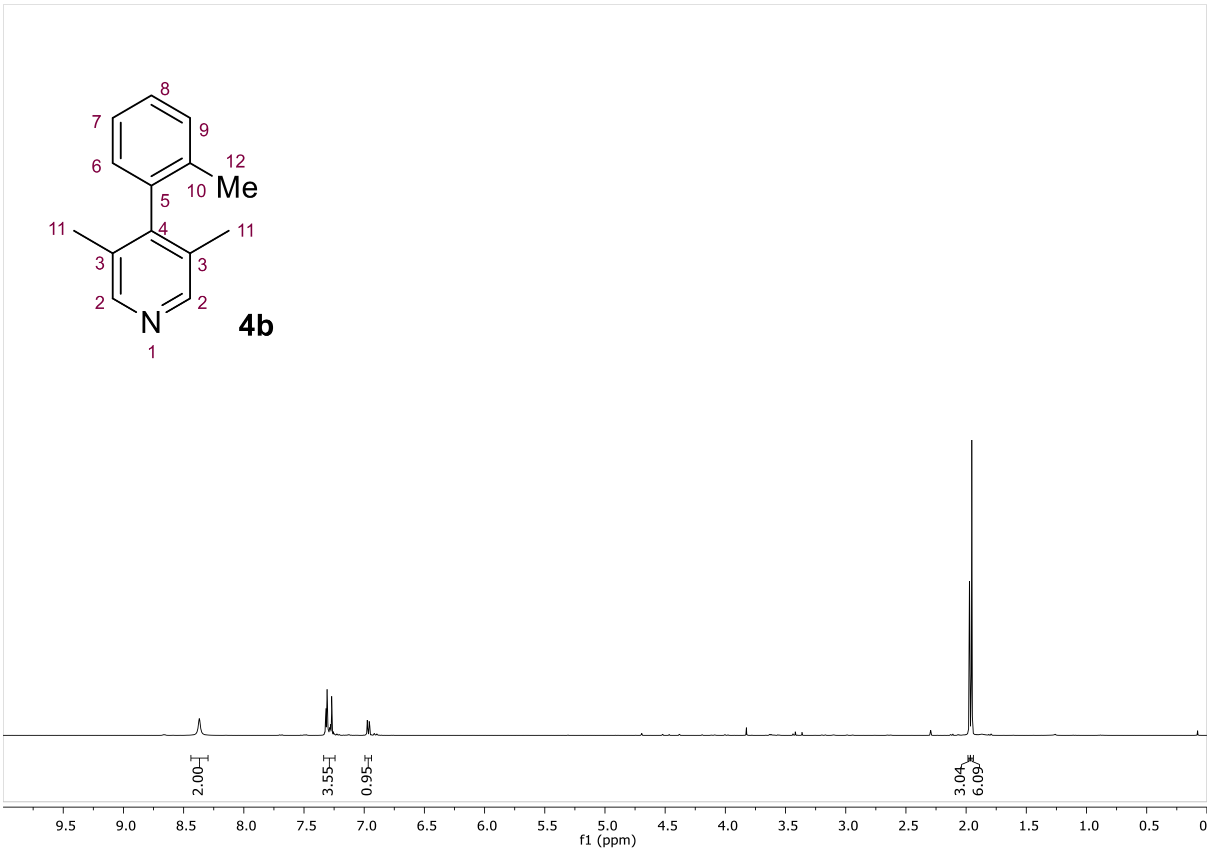
8
7
9
6
12
10 Me
5
11
4
11
3
3
2
N
2
1
4b
2.00
3.55
0.95
3.04
6.09
9.5 9.0 8.5 8.0 7.5 7.0 6.5 6.0 5.5 5.0 4.5 4.0 3.5 3.0 2.5 2.0 1.5 1.0 0.5 0
f1 (ppm)

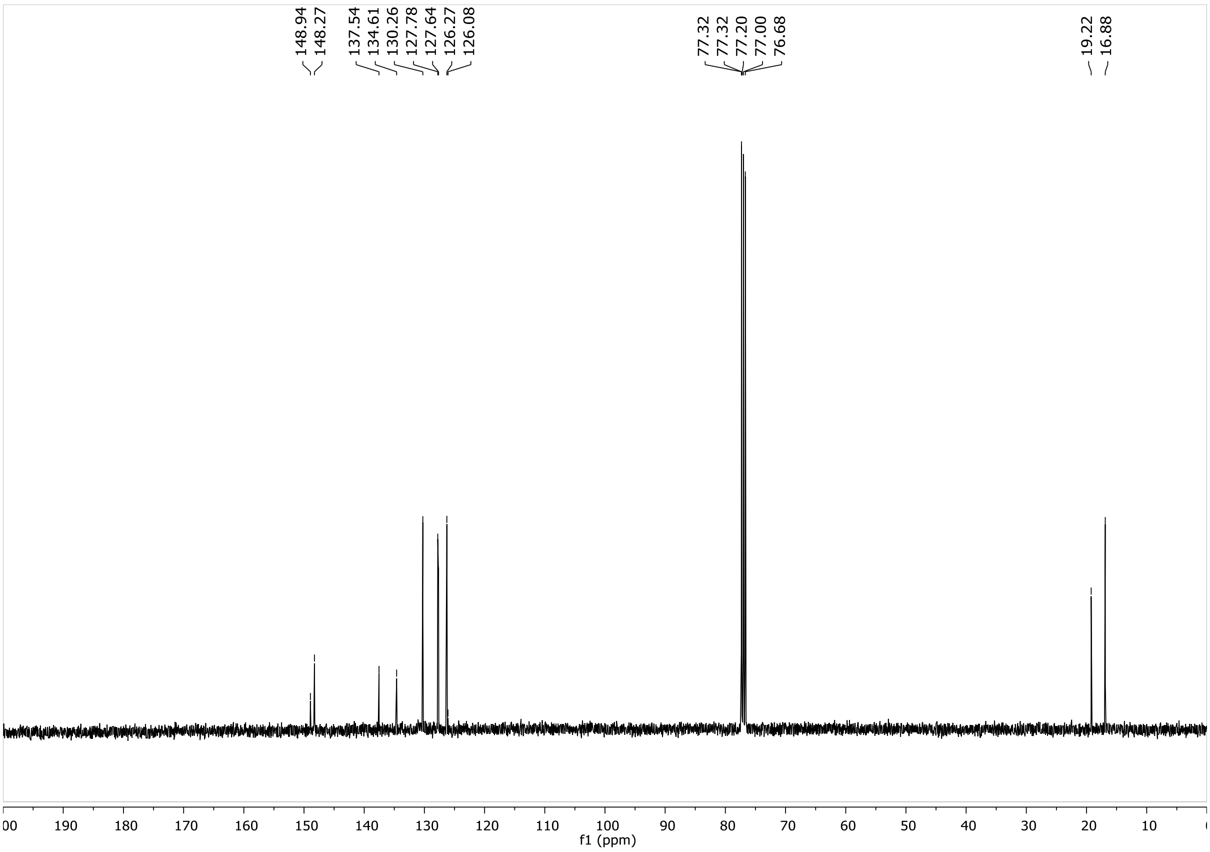
148.94
148.27
137.54
134.61
130.26
127.78
127.64
126.27
126.08
77.32
77.32
77.20
77.00
76.68
19.22
16.88
190 180 170 160 150 140 130 120 110 100 90 80 70 60 50 40 30 20 10
f1 (ppm)

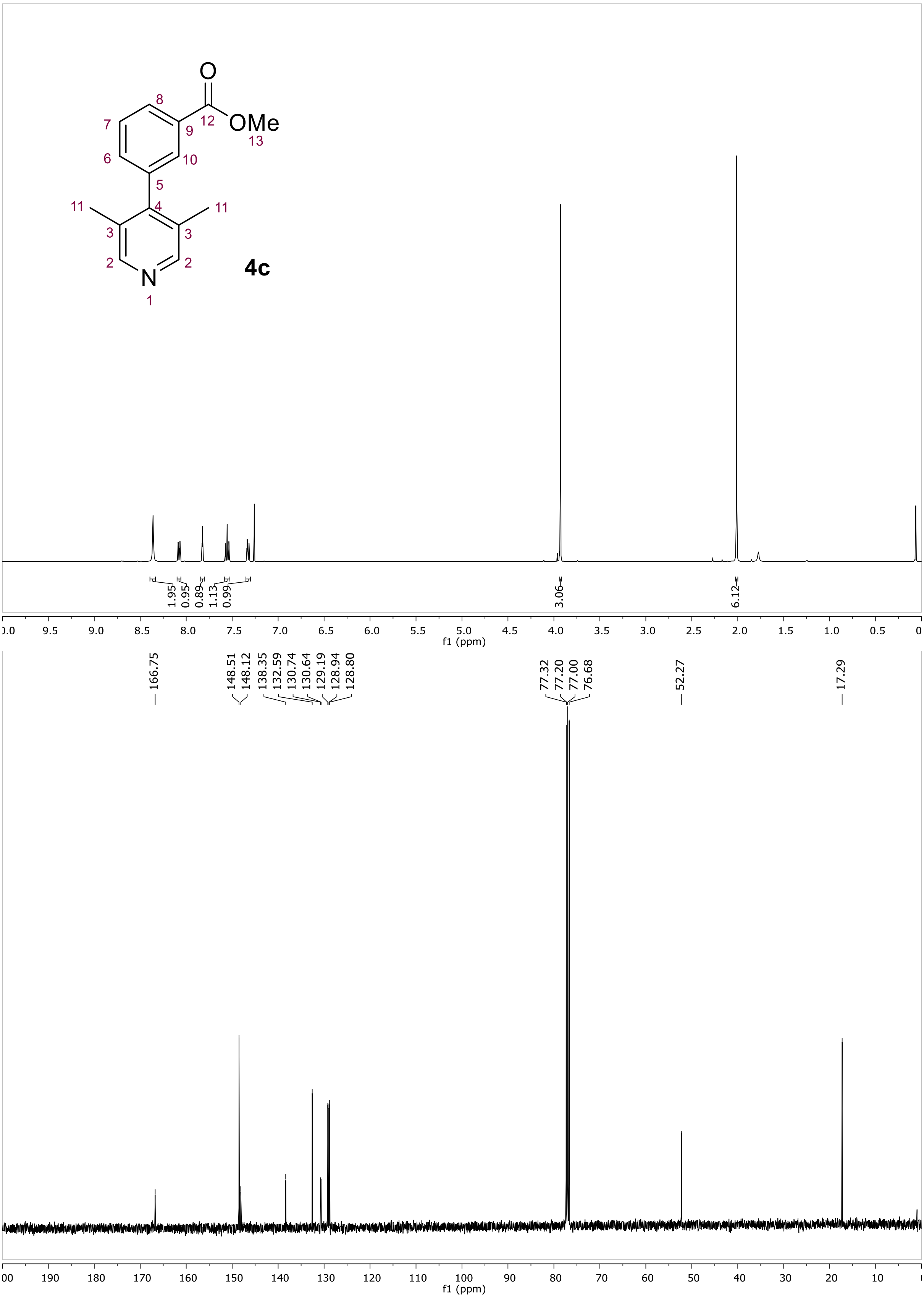
4c
OMe
f1 (ppm)
166.75
148.51
148.12
138.35
132.59
130.74
130.64
129.19
128.94
128.80
77.32
77.20
77.00
76.68
52.27
17.29

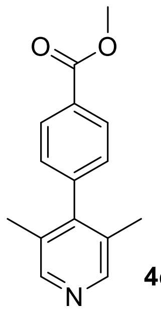

4d

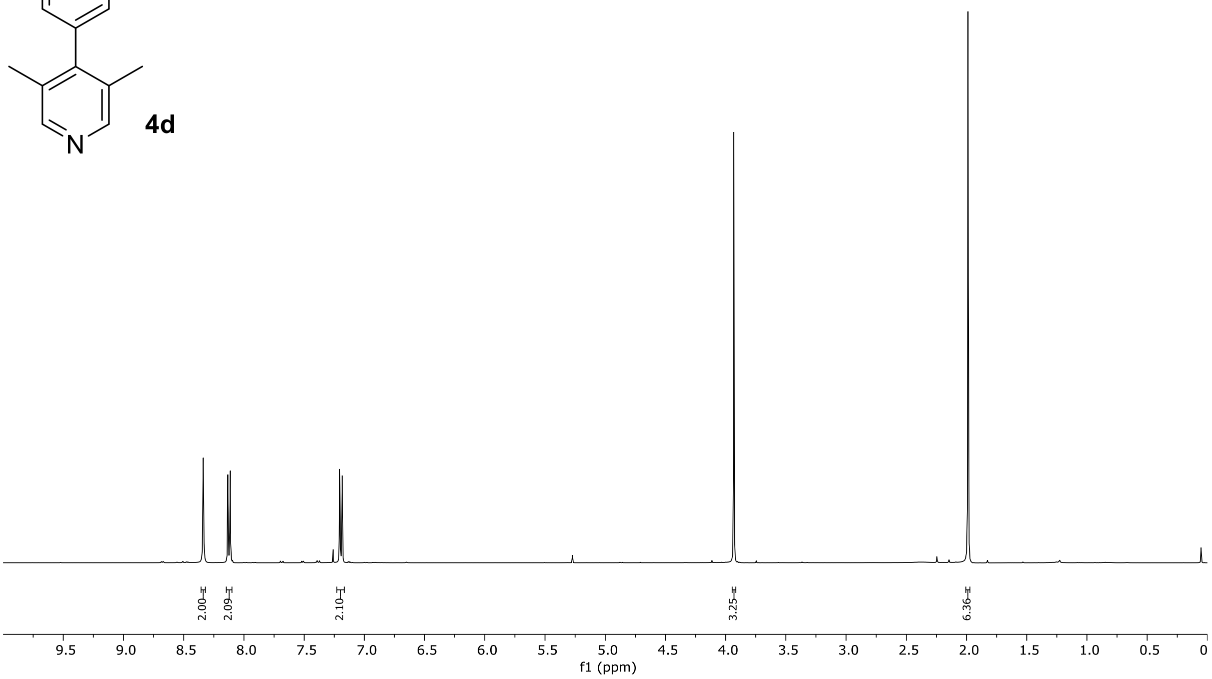
2.00
2.09
2.10
3.25
6.36
9.5
9.0
8.5
8.0
7.5
7.0
6.5
6.0
5.5
5.0
4.5
4.0
3.5
3.0
2.5
2.0
1.5
1.0
0.5
0
f1 (ppm)

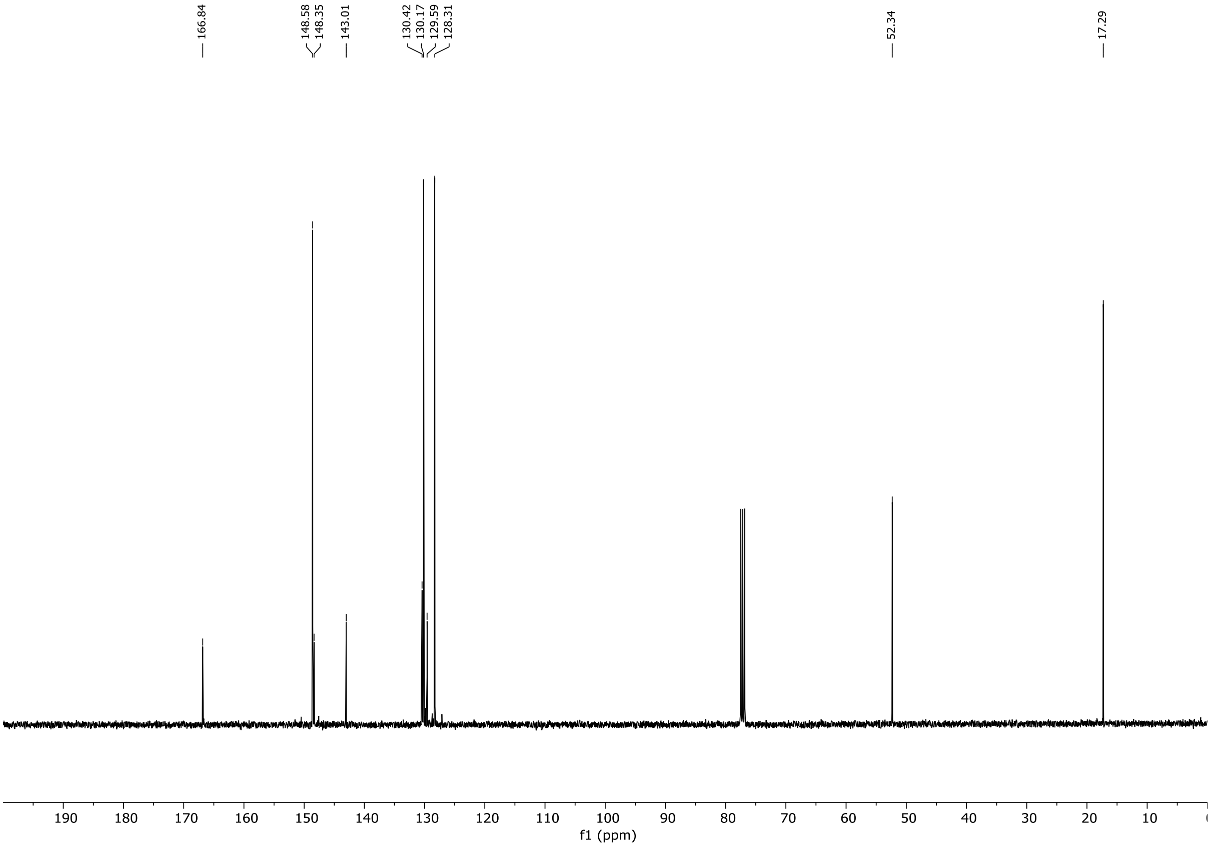
166.84
148.58
148.35
143.01
130.42
130.17
129.59
128.31
52.34
17.29
190
180
170
160
150
140
130
120
110
100
90
80
70
60
50
40
30
20
10
f1 (ppm)

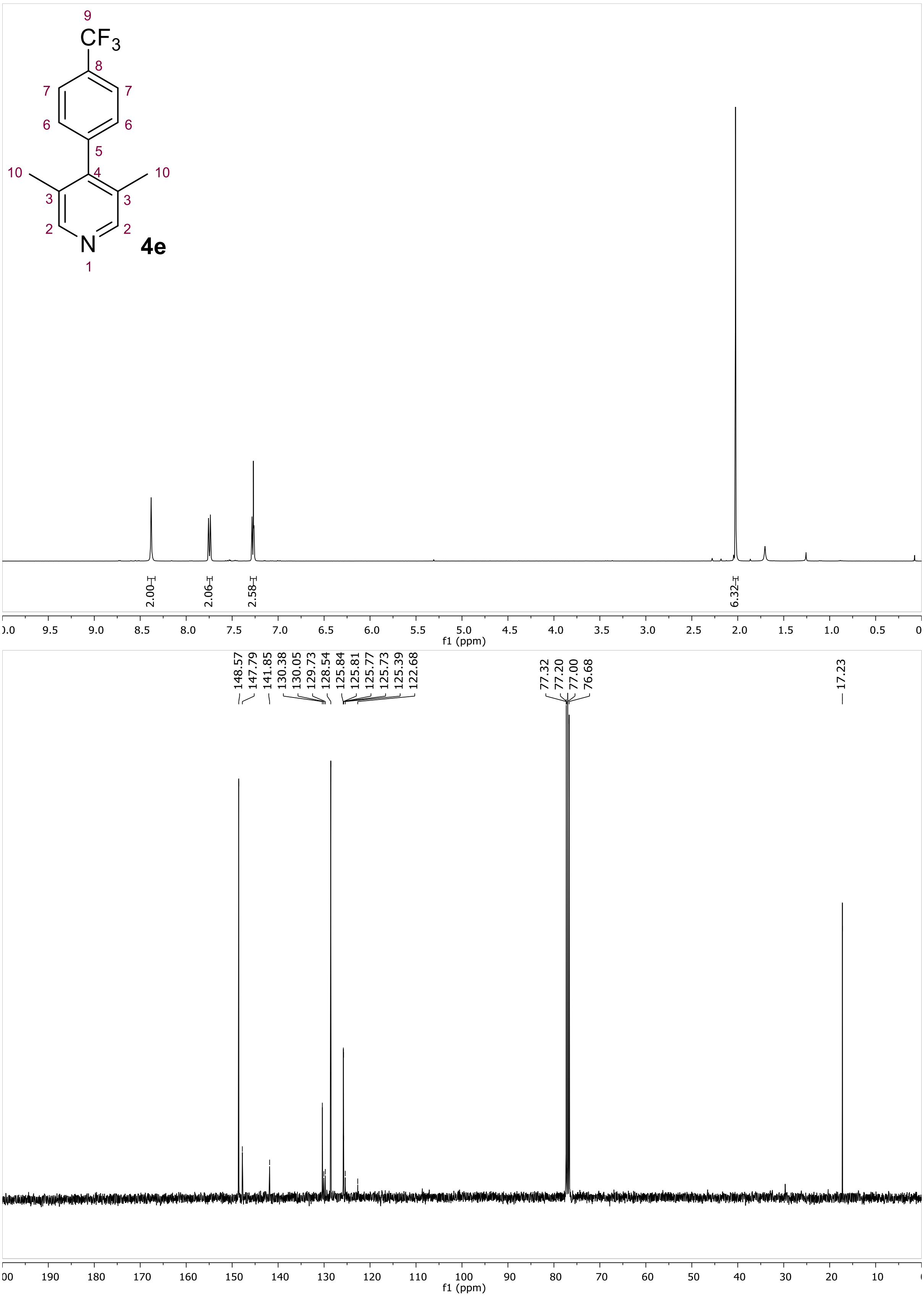

9
CF3
8
7
7
6
6
5
10
4
10
3
3
2
2
N
1
4e
2.00
2.06
2.58
6.32
f1 (ppm)
148.57
147.79
141.85
130.38
130.05
129.73
128.54
125.84
125.81
125.77
125.73
125.39
122.68
77.32
77.20
77.00
76.68
17.23
f1 (ppm)

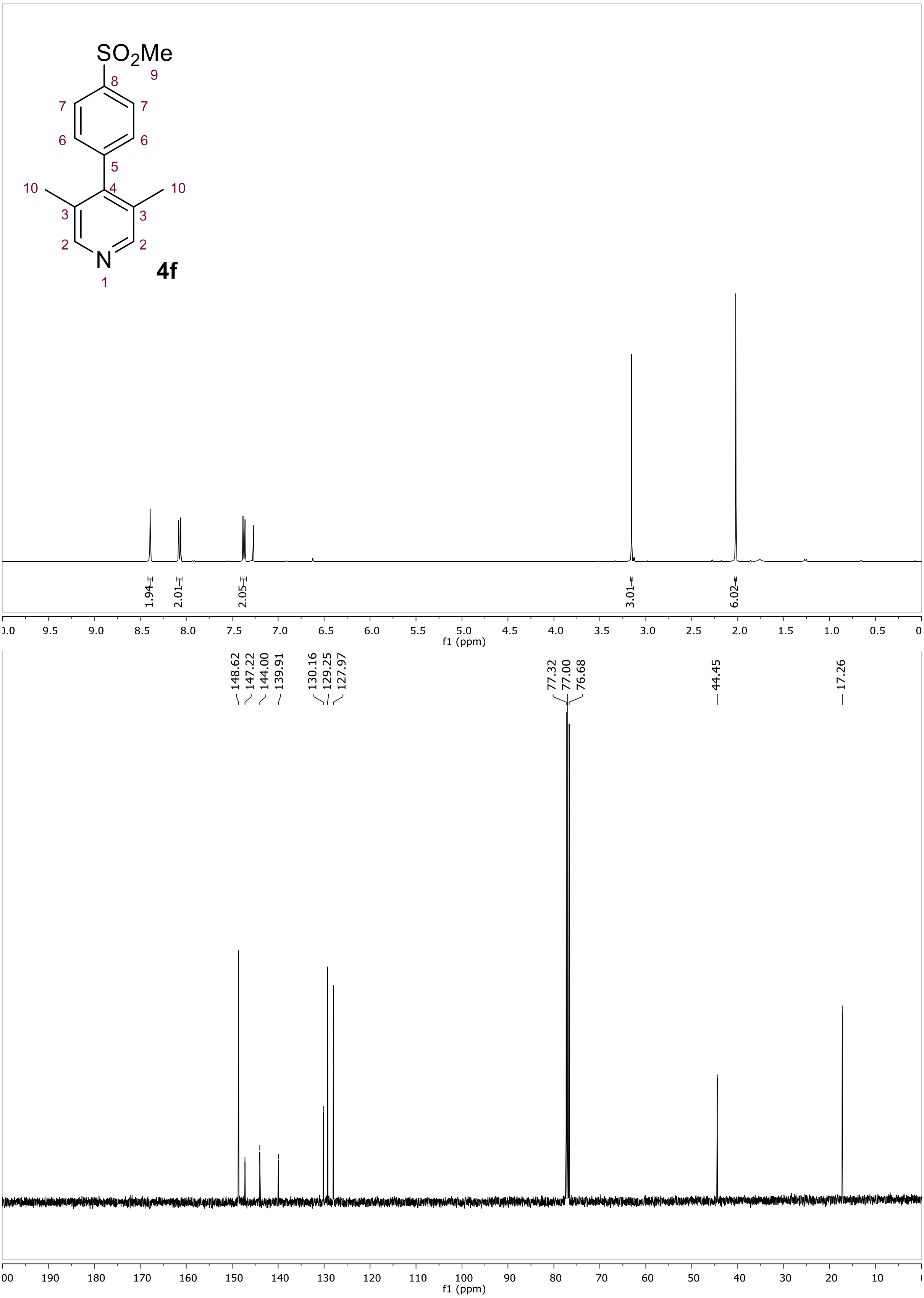
SO2Me
4f
1.94
2.01
2.05
3.01
6.02
f1 (ppm)
148.62
147.22
144.00
139.91
130.16
129.25
127.97
77.32
77.00
76.68
44.45
17.26
f1 (ppm)

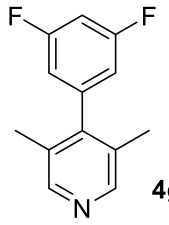
F
F
N

4g

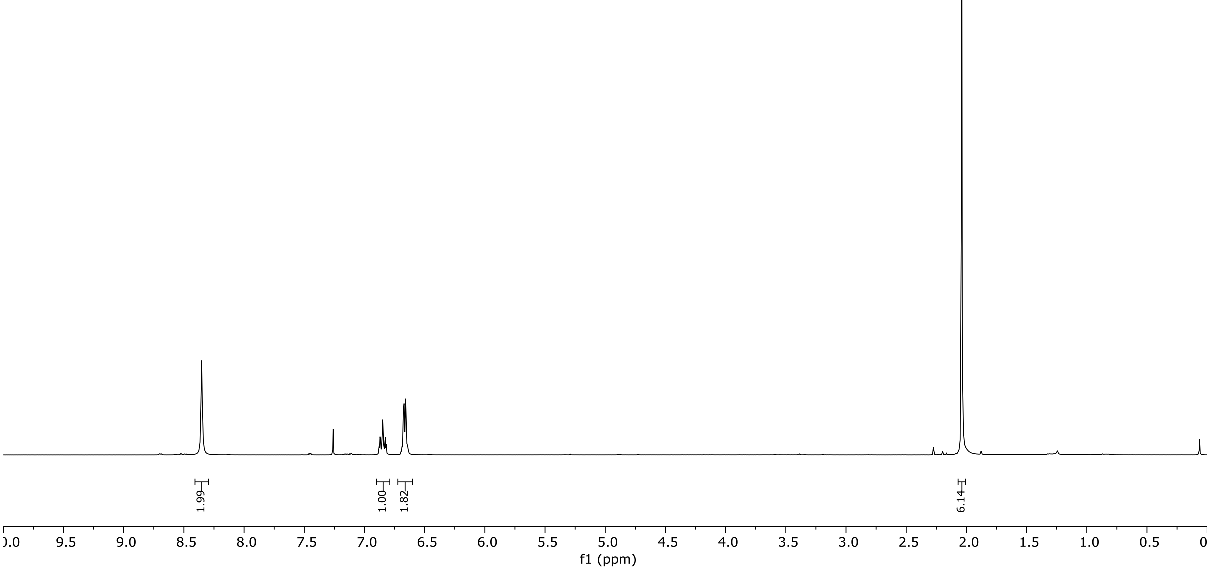
1.99
1.00
1.82
6.14
0.0 9.5 9.0 8.5 8.0 7.5 7.0 6.5 6.0 5.5 5.0 4.5 4.0 3.5 3.0 2.5 2.0 1.5 1.0 0.5 0
f1 (ppm)

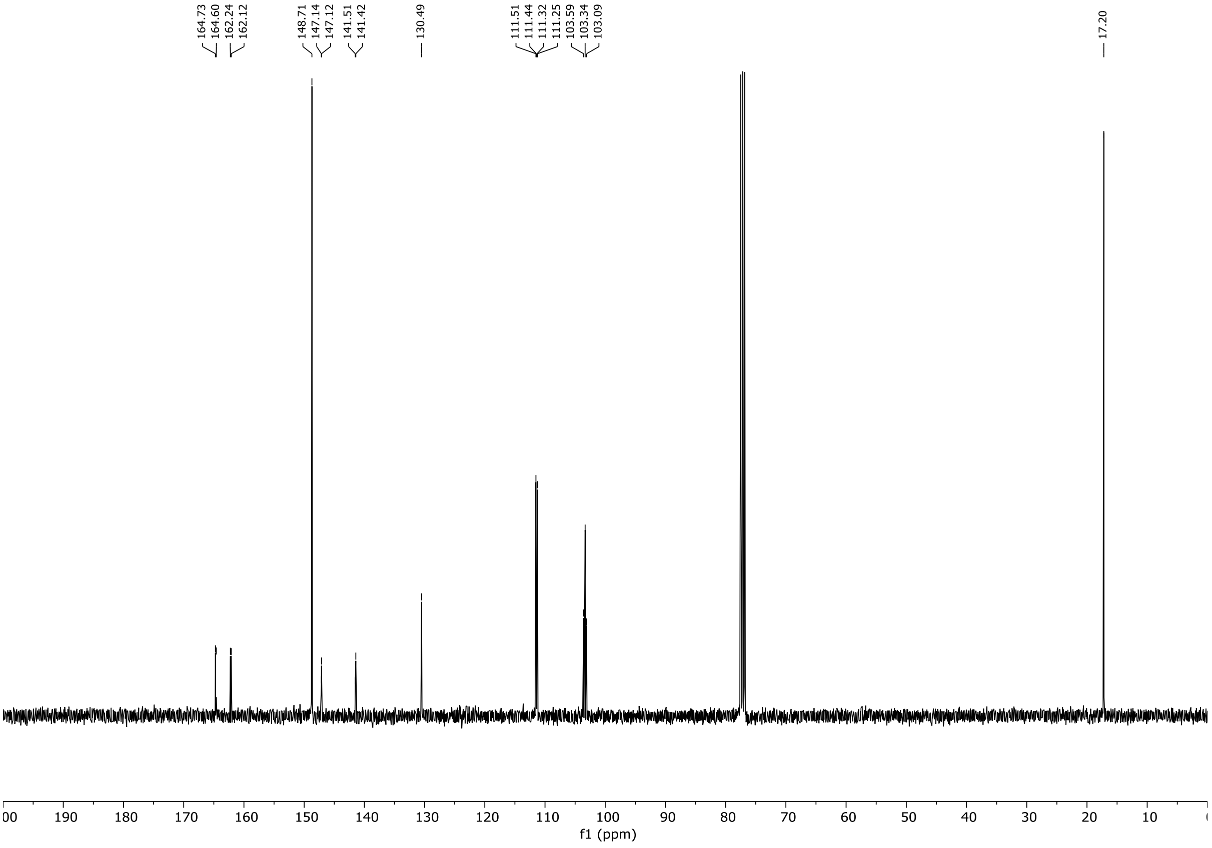
164.73
164.60
162.24
162.12
148.71
147.14
147.12
141.51
141.42
130.49
111.51
111.44
111.32
111.25
103.59
103.34
103.09
17.20
00 190 180 170 160 150 140 130 120 110 100 90 80 70 60 50 40 30 20 10
f1 (ppm)

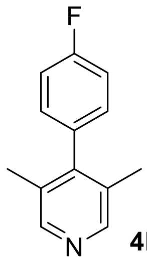
F
N

4h

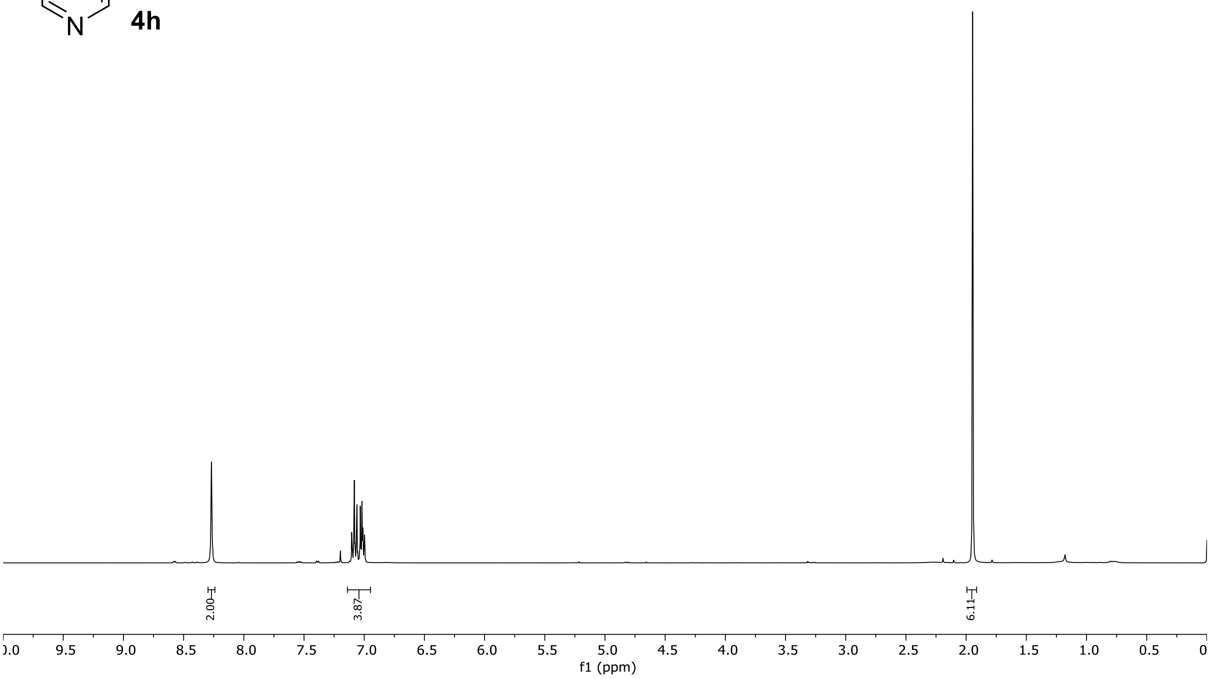
2.00
3.87
6.11
f1 (ppm)

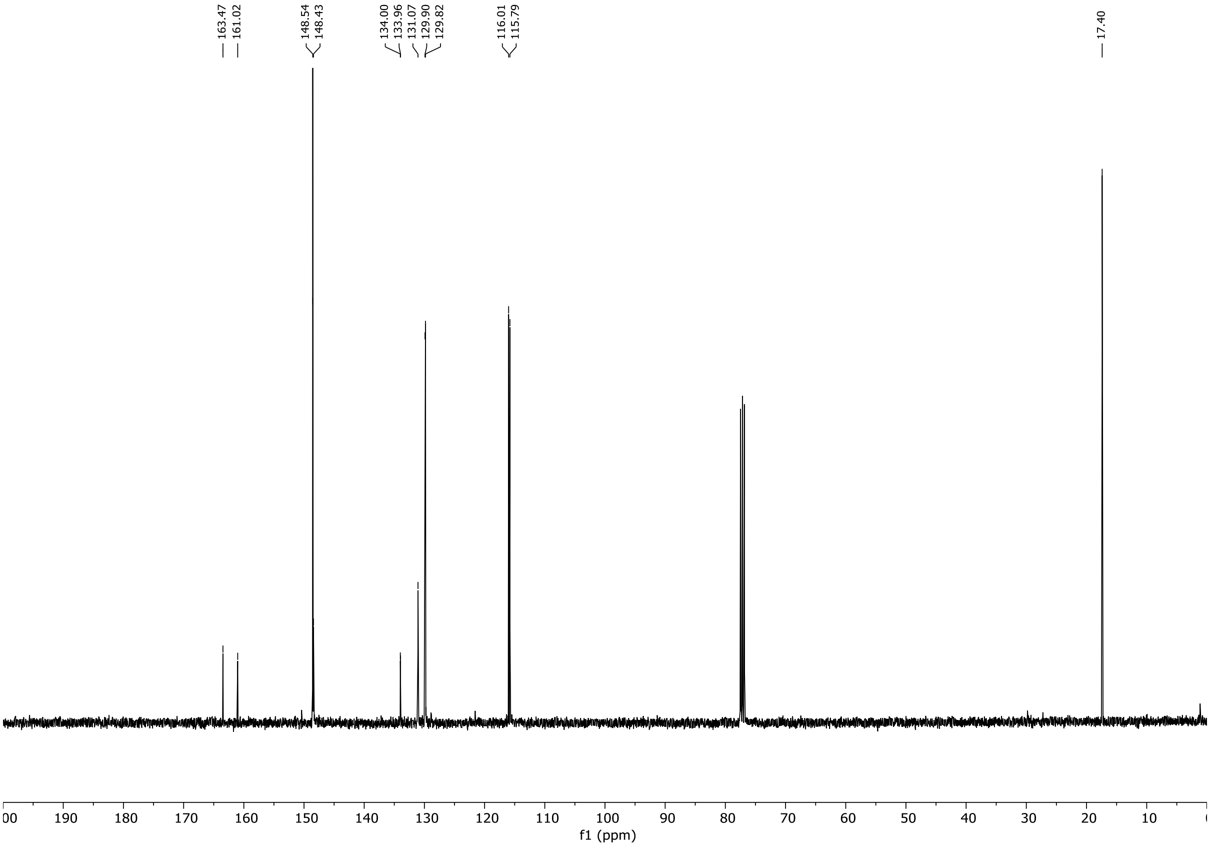
163.47
161.02
148.54
148.43
134.00
133.96
131.07
129.90
129.82
116.01
115.79
17.40
f1 (ppm)

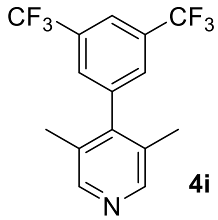
CF3
CF3
N

4i

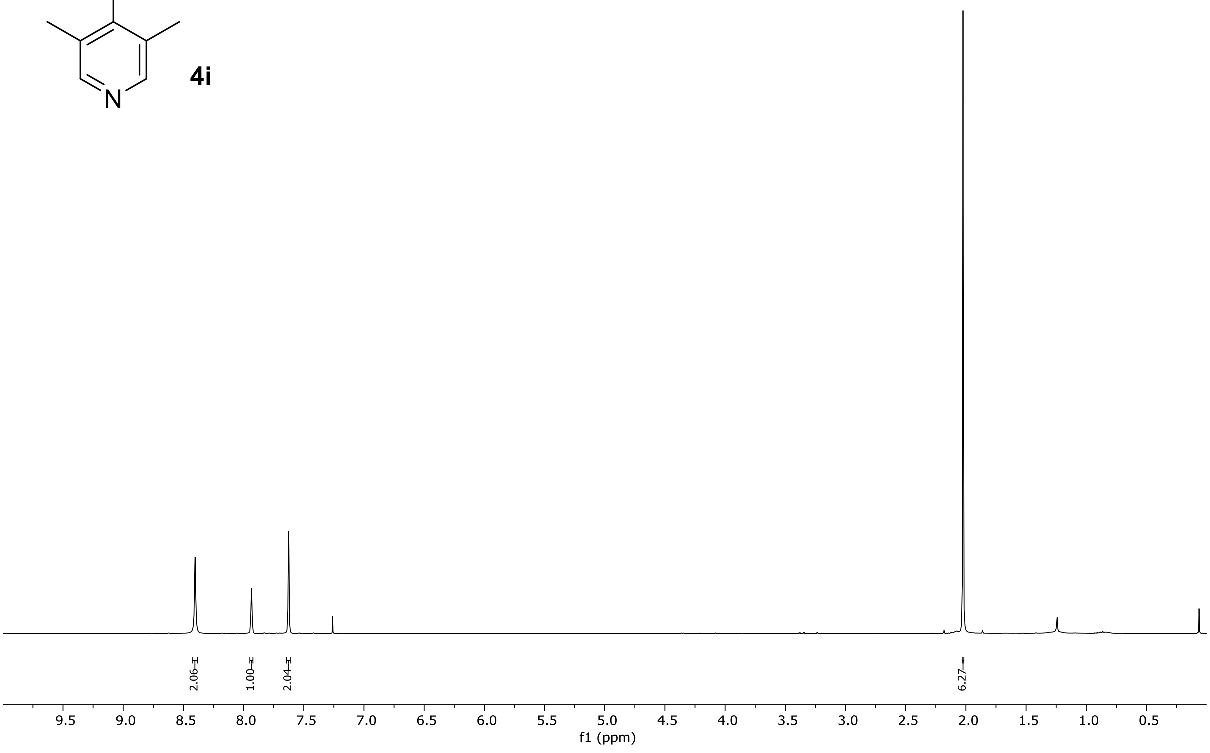
2.06
1.00
2.04
6.27
9.5 9.0 8.5 8.0 7.5 7.0 6.5 6.0 5.5 5.0 4.5 4.0 3.5 3.0 2.5 2.0 1.5 1.0 0.5
f1 (ppm)

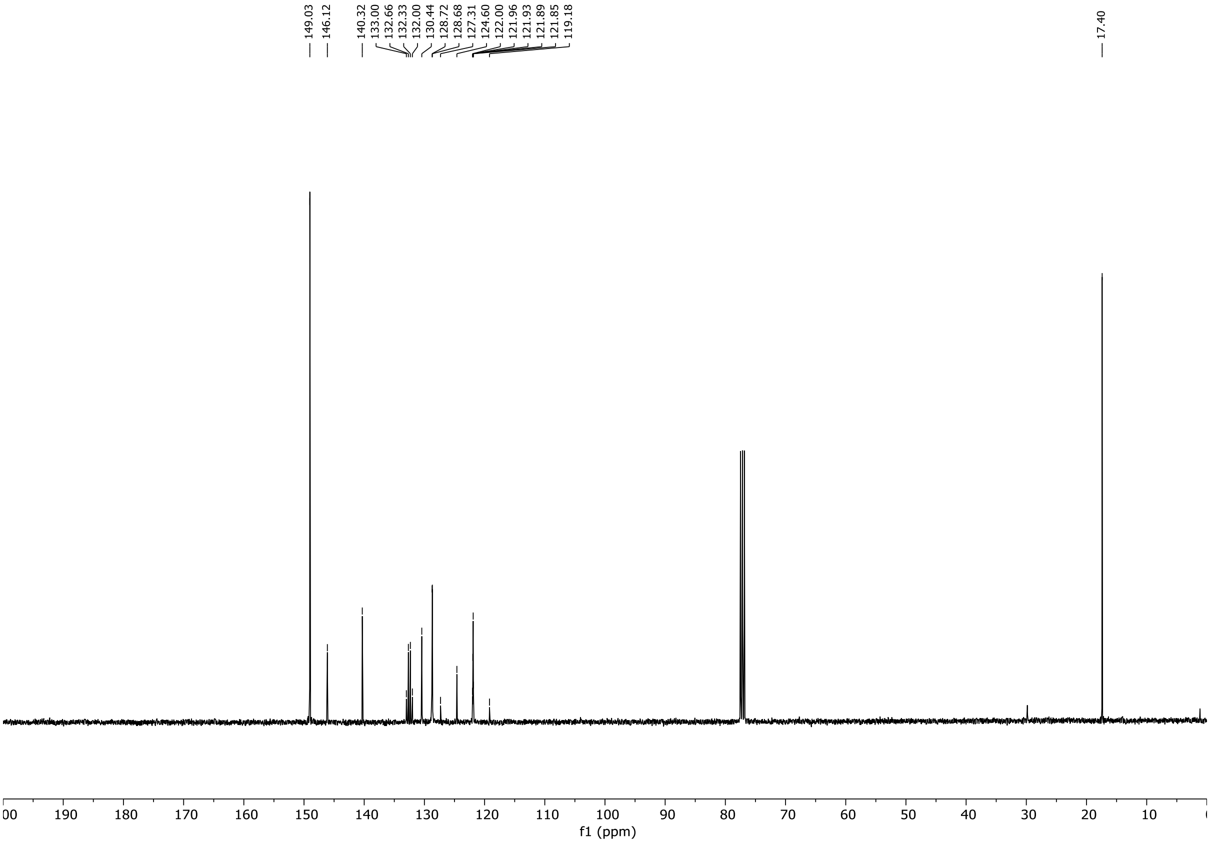
149.03
146.12
140.32
133.00
132.66
132.33
132.00
130.44
128.72
128.68
127.31
124.60
122.00
121.96
121.93
121.89
121.85
119.18
17.40
190 180 170 160 150 140 130 120 110 100 90 80 70 60 50 40 30 20 10
f1 (ppm)

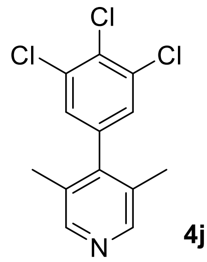
Cl
Cl
Cl
N

4j

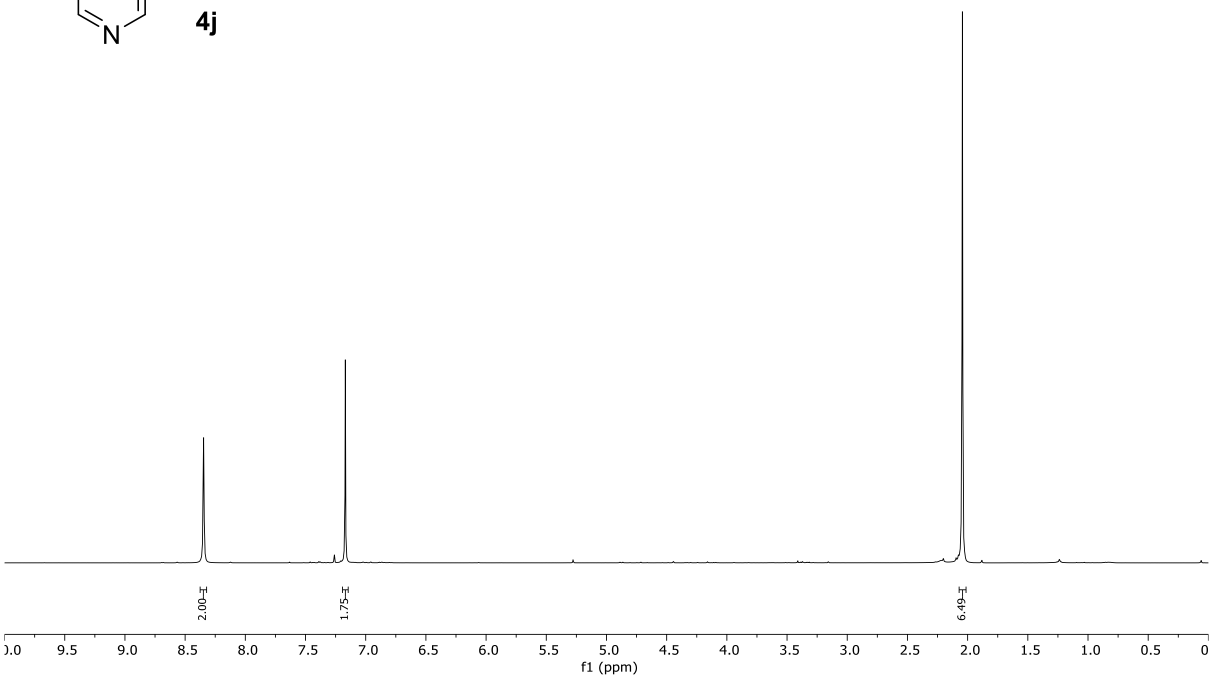
2.00
1.75
6.49
f1 (ppm)

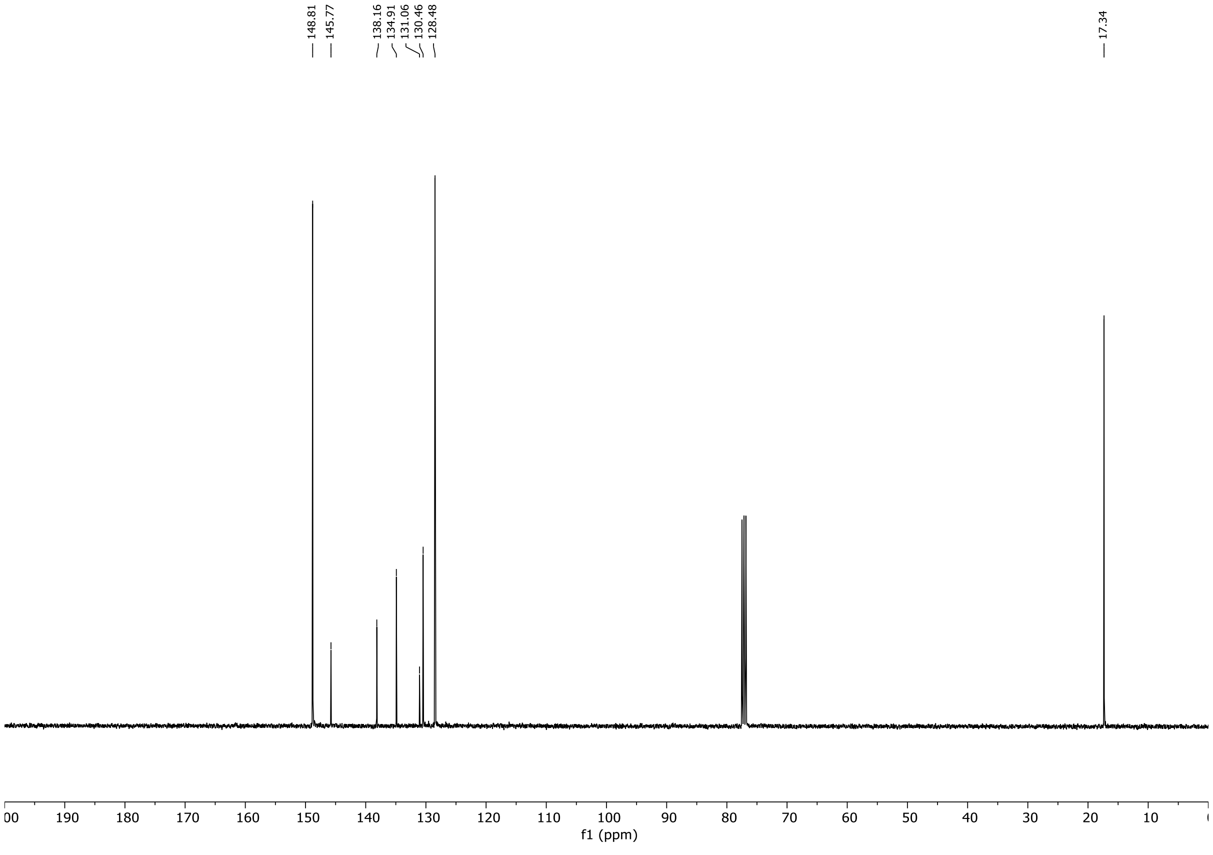
148.81
145.77
138.16
134.91
131.06
130.46
128.48
17.34
f1 (ppm)

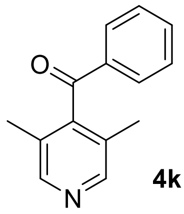
O
N

4k

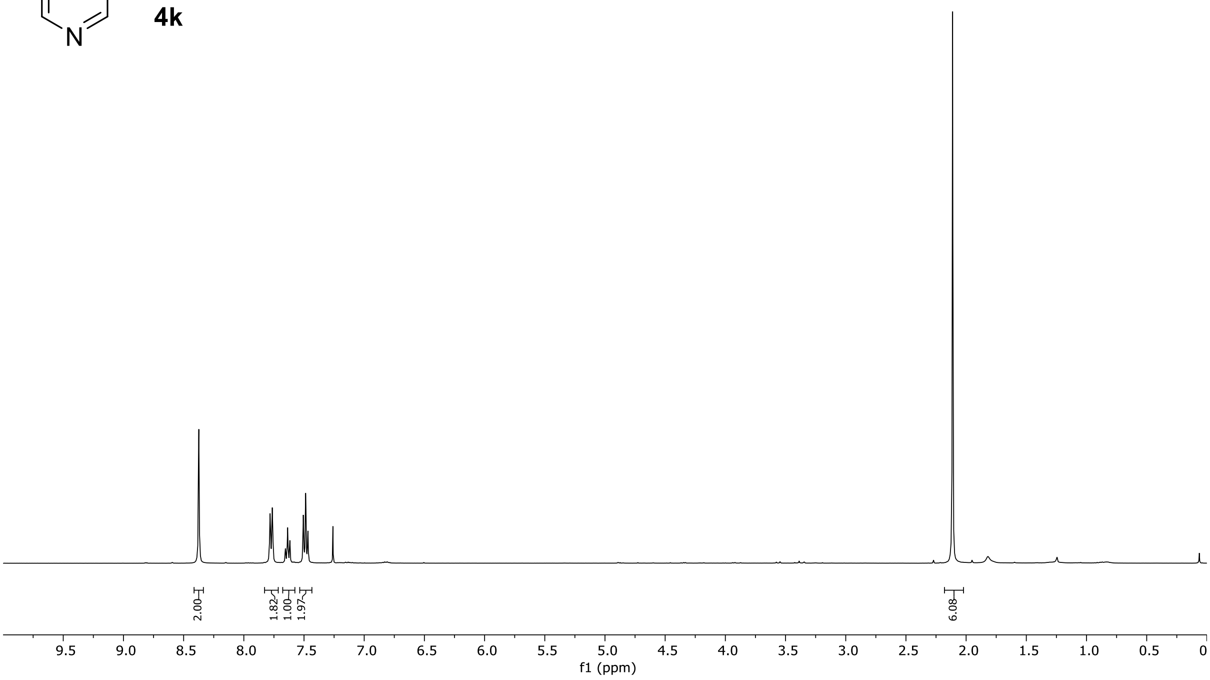
2.00
1.82
1.00
1.97
6.08
9.5 9.0 8.5 8.0 7.5 7.0 6.5 6.0 5.5 5.0 4.5 4.0 3.5 3.0 2.5 2.0 1.5 1.0 0.5 0
f1 (ppm)

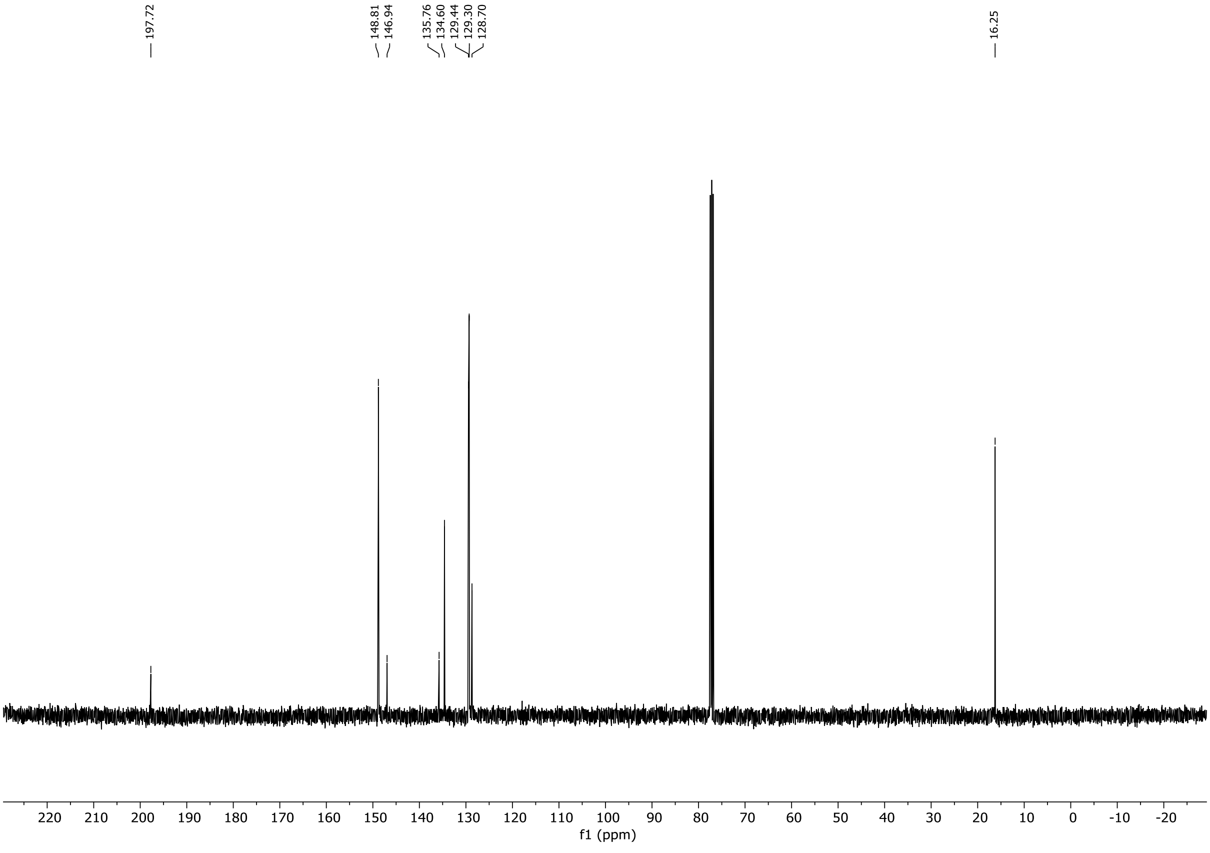
197.72
148.81
146.94
135.76
134.60
129.44
129.30
128.70
16.25
220 210 200 190 180 170 160 150 140 130 120 110 100 90 80 70 60 50 40 30 20 10 0 -10 -20
f1 (ppm)

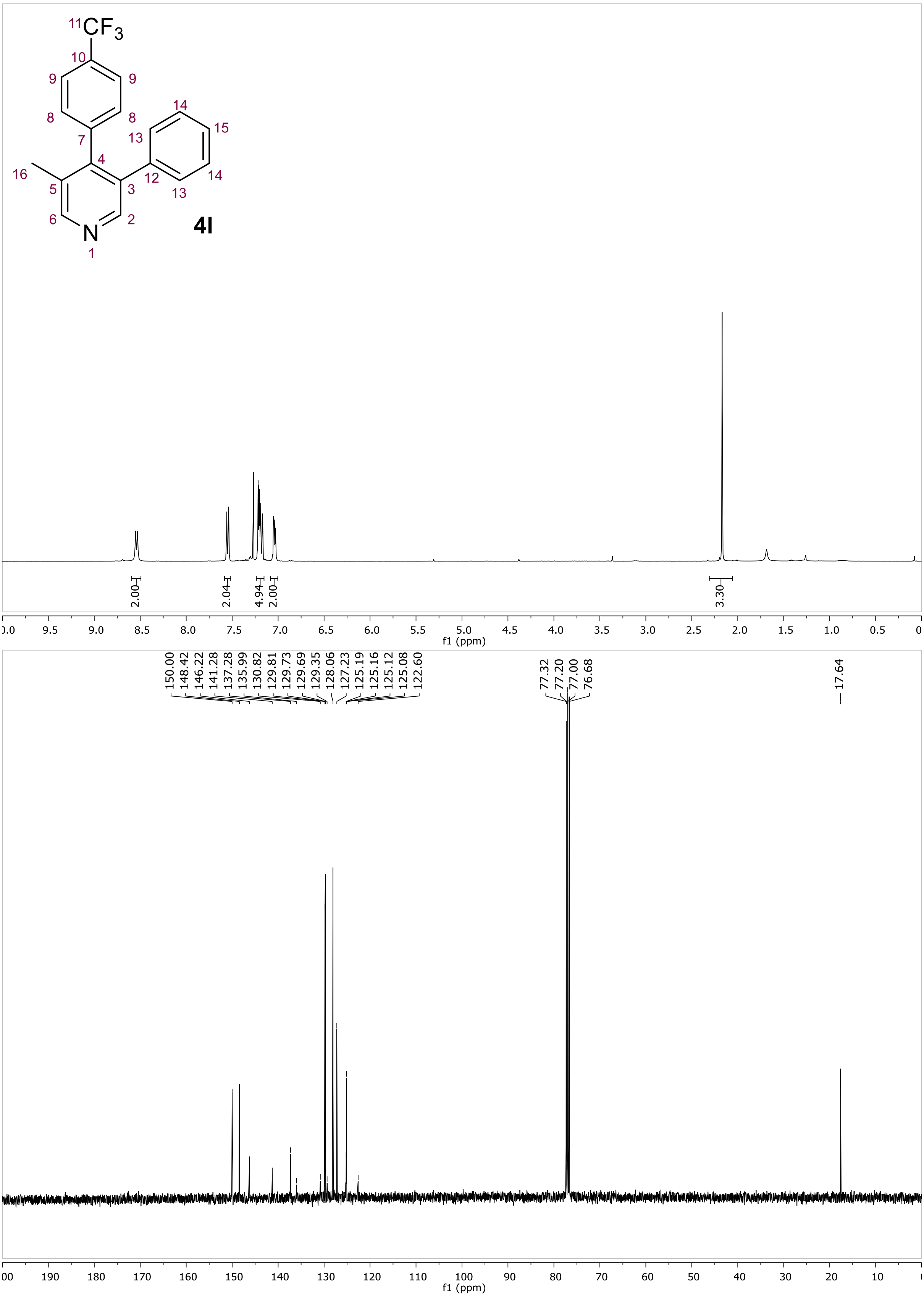

11CF3
10
9
9
8
8
7
4
16
5
3
6
N
1
2
14
15
13
12
14
13
4l
2.00
2.04
4.94
2.00
3.30
f1 (ppm)
150.00
148.42
146.22
141.28
137.28
135.99
130.82
129.81
129.73
129.69
129.35
128.06
127.23
125.19
125.16
125.12
125.08
122.60
77.32
77.20
77.00
76.68
17.64
f1 (ppm)

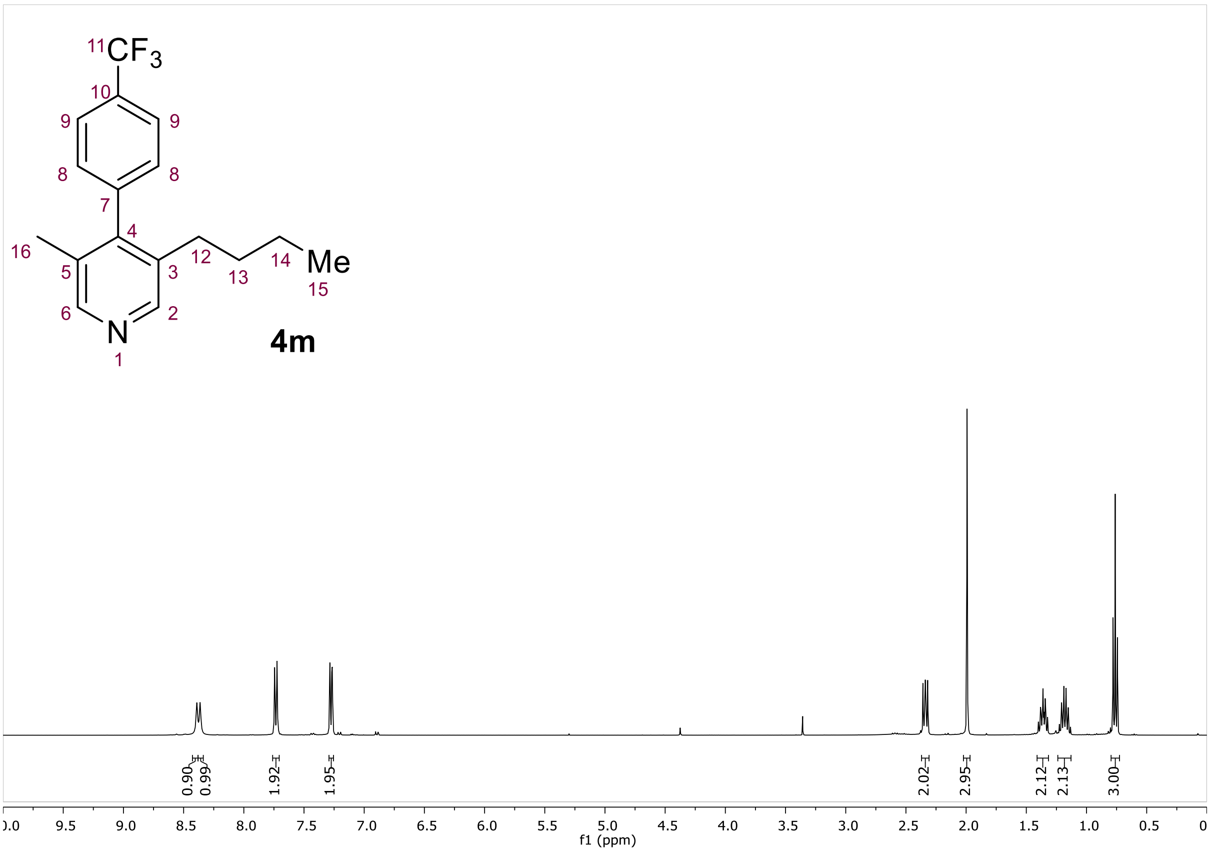
11CF3
10
9
9
8
8
7
4
16
5
3
12
13
14
Me
15
6
2
N
1
4m
0.90
0.99
1.92
1.95
2.02
2.95
2.12
2.13
3.00
9.5 9.0 8.5 8.0 7.5 7.0 6.5 6.0 5.5 5.0 4.5 4.0 3.5 3.0 2.5 2.0 1.5 1.0 0.5
f1 (ppm)

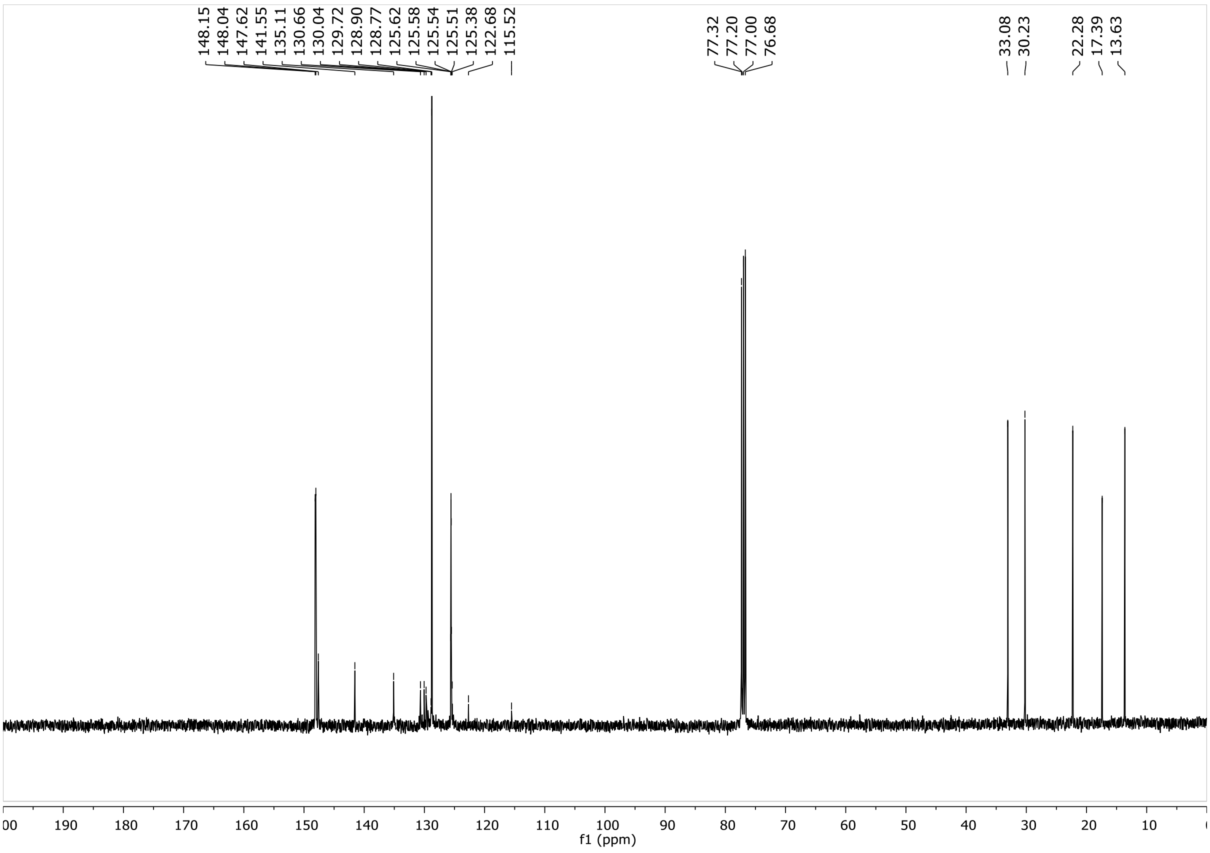
148.15
148.04
147.62
141.55
135.11
130.66
130.04
129.72
128.90
128.77
125.62
125.58
125.54
125.51
125.38
122.68
115.52
77.32
77.20
77.00
76.68
33.08
30.23
22.28
17.39
13.63
190 180 170 160 150 140 130 120 110 100 90 80 70 60 50 40 30 20 10
f1 (ppm)

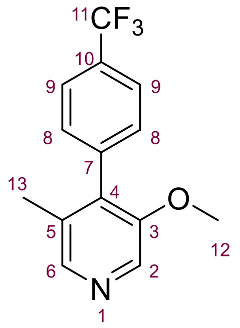

CF3
1
2
3
4
5
6
7
8
9
10
11
12
13
N
O

4n

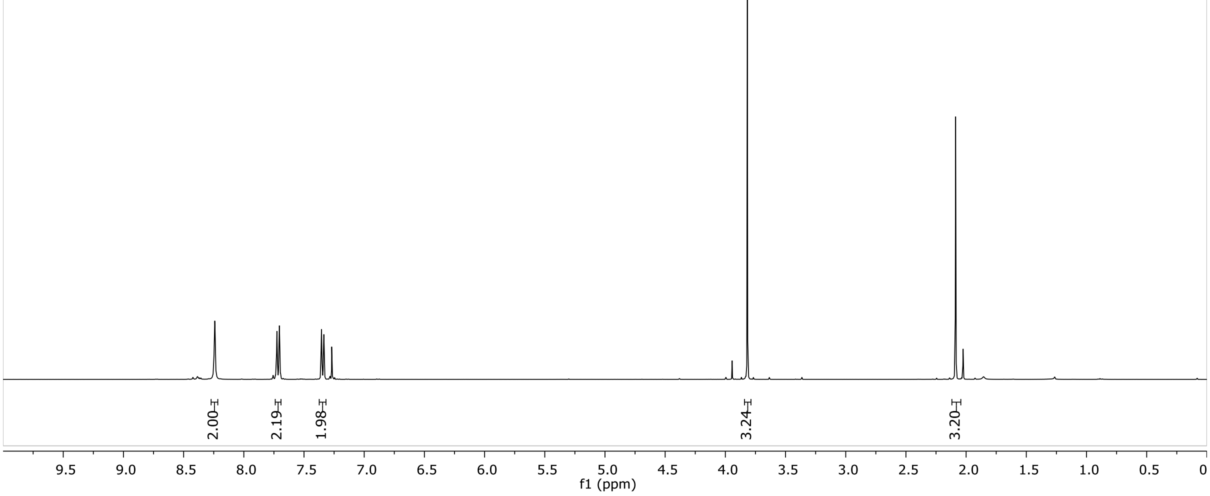

2.00
2.19
1.98
3.24
3.20
f1 (ppm)

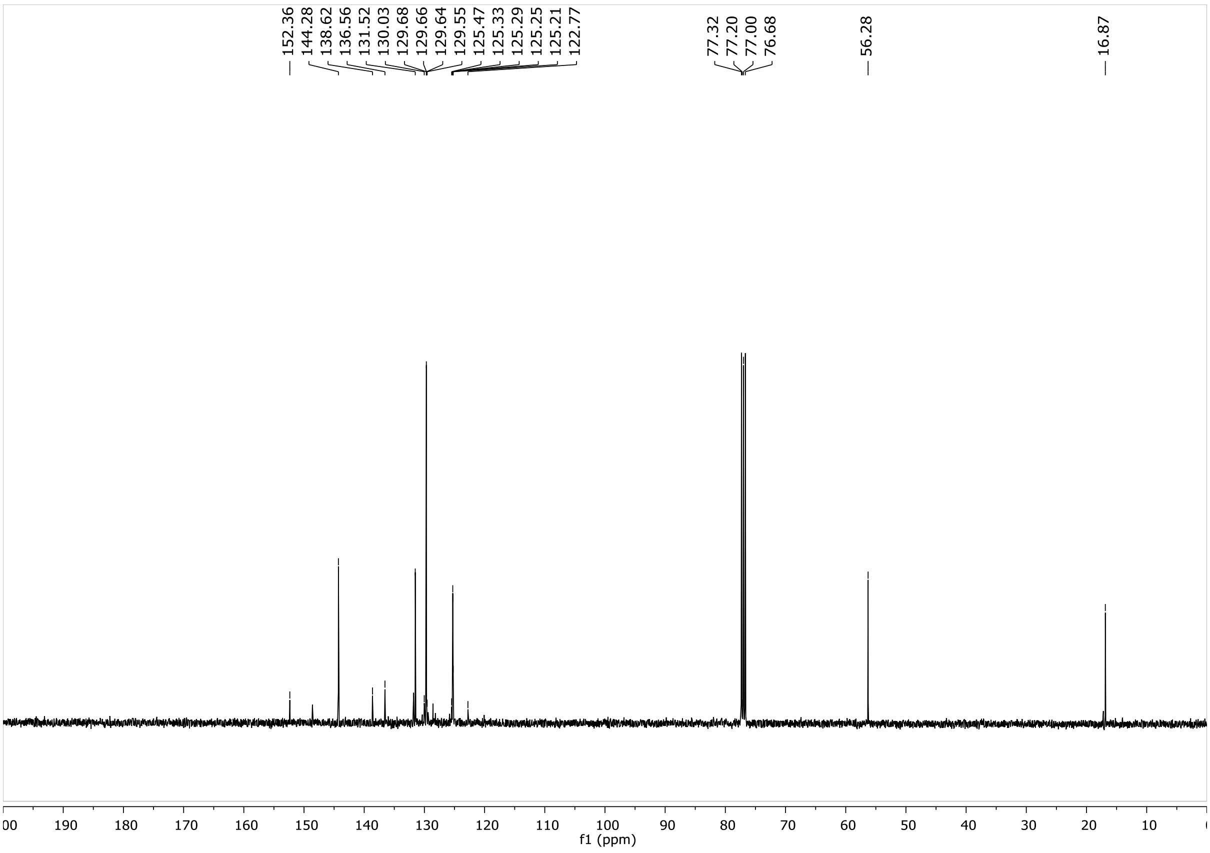

152.36
144.28
138.62
136.56
131.52
130.03
129.68
129.66
129.64
129.55
125.47
125.33
125.29
125.25
125.21
122.77
77.32
77.20
77.00
76.68
56.28
16.87
f1 (ppm)

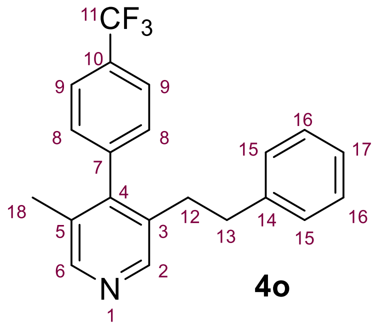

4o

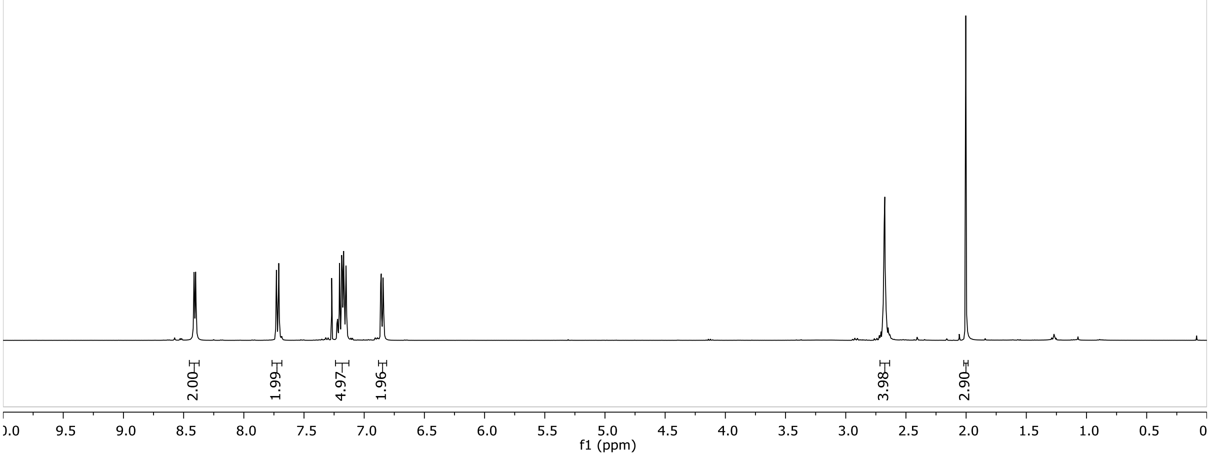

2.00
1.99
4.97
1.96
3.98
2.90
f1 (ppm)

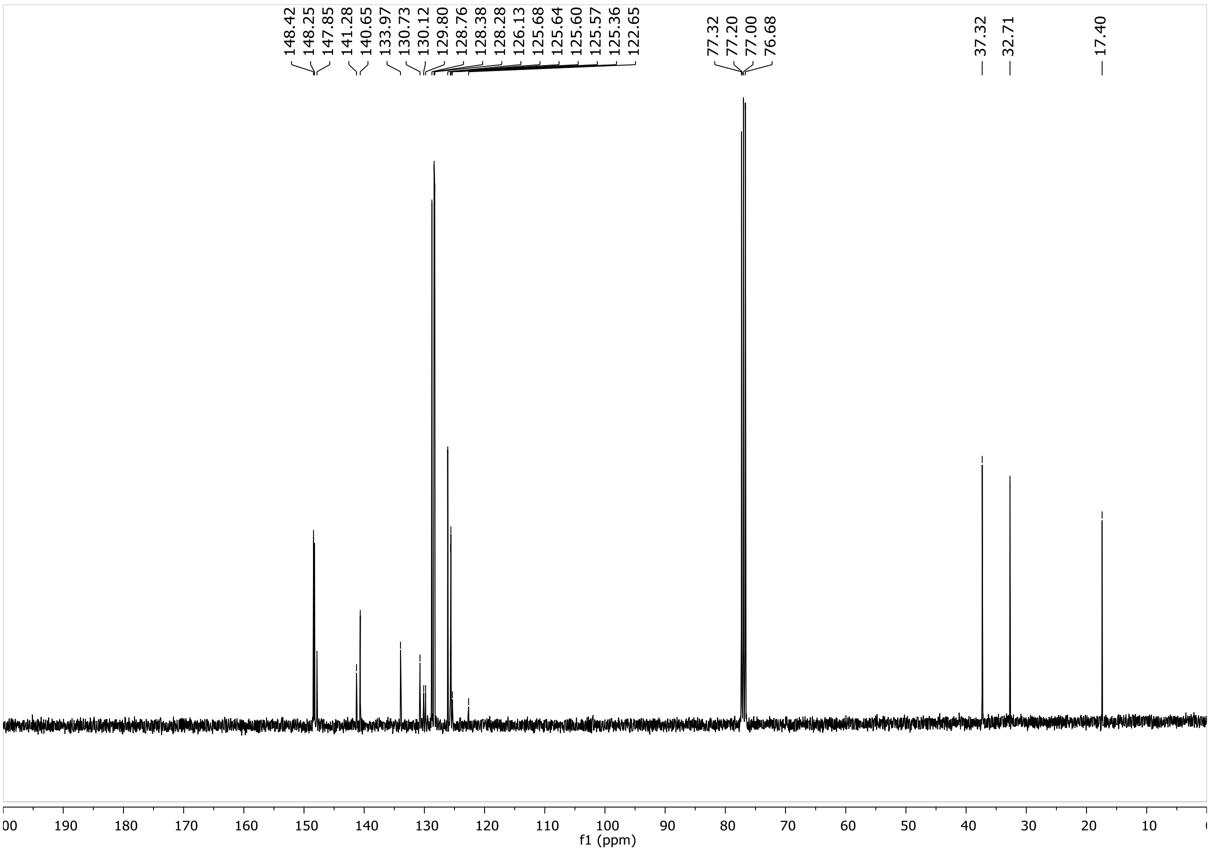

148.42
148.25
147.85
141.28
140.65
133.97
130.73
130.12
129.80
128.76
128.38
128.28
126.13
125.68
125.64
125.60
125.57
125.36
122.65
77.32
77.20
77.00
76.68
37.32
32.71
17.40
f1 (ppm)

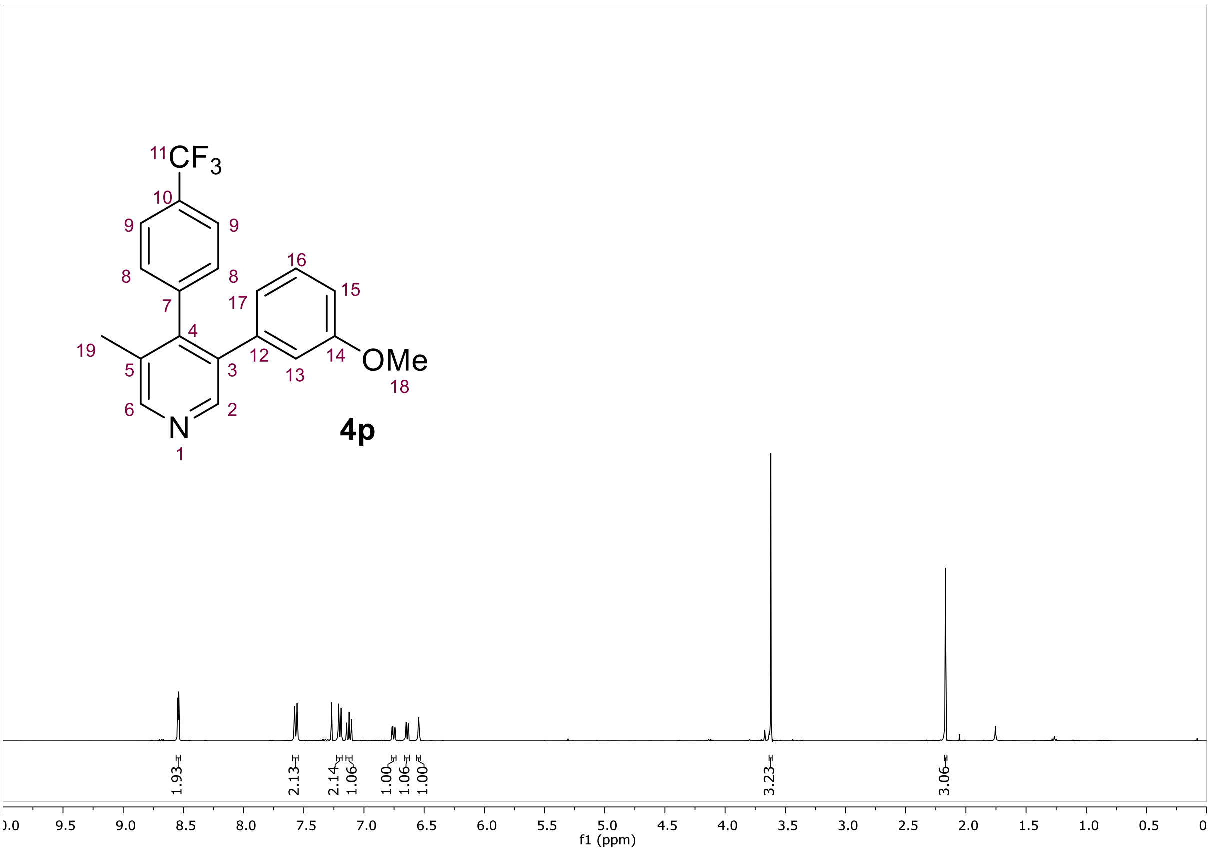
11CF3
10
9
9
8
8
7
4
16
15
17
12
14
OMe
18
19
5
3
13
6
2
N
1
4p
1.93
2.13
2.14
1.06
1.00
1.06
1.00
3.23
3.06
f1 (ppm)

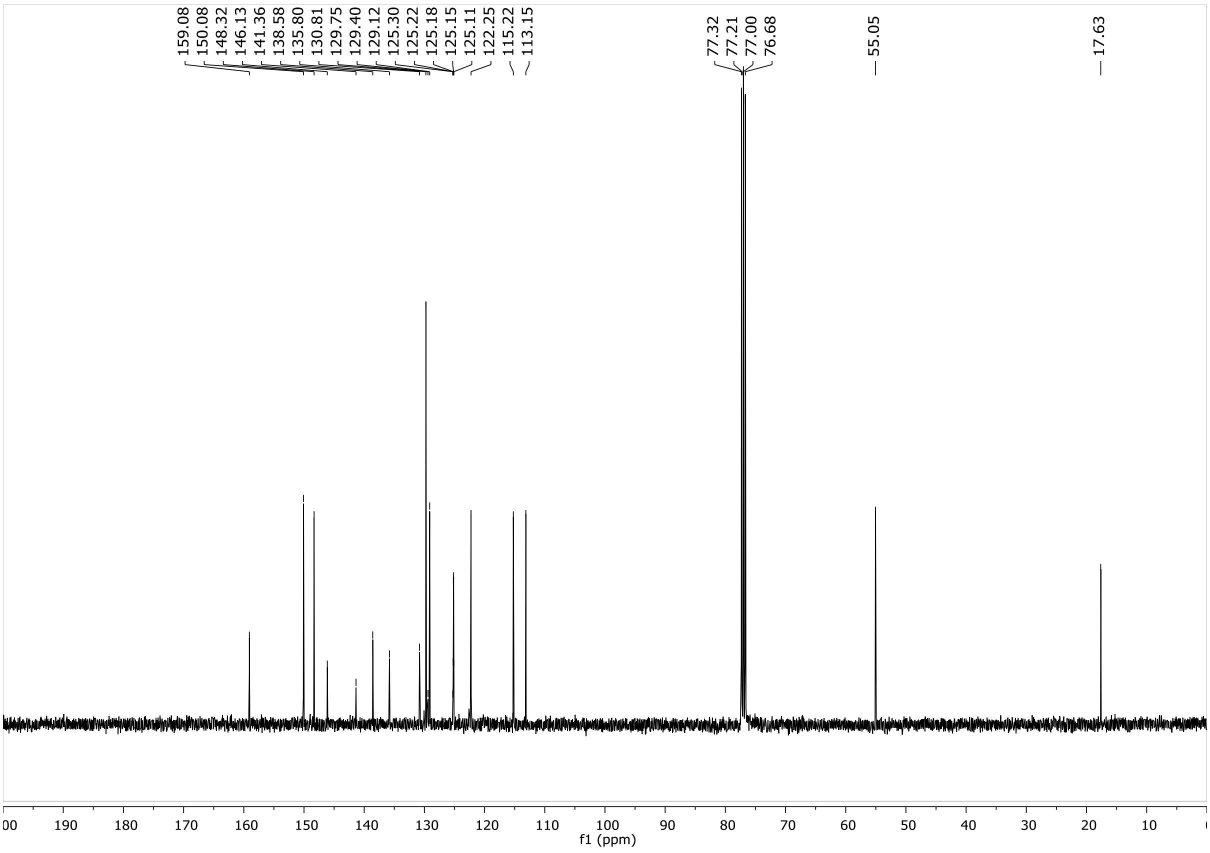
159.08
150.08
148.32
146.13
141.36
138.58
135.80
130.81
129.75
129.40
129.12
125.30
125.22
125.18
125.15
125.11
122.25
115.22
113.15
77.32
77.21
77.00
76.68
55.05
17.63
f1 (ppm)

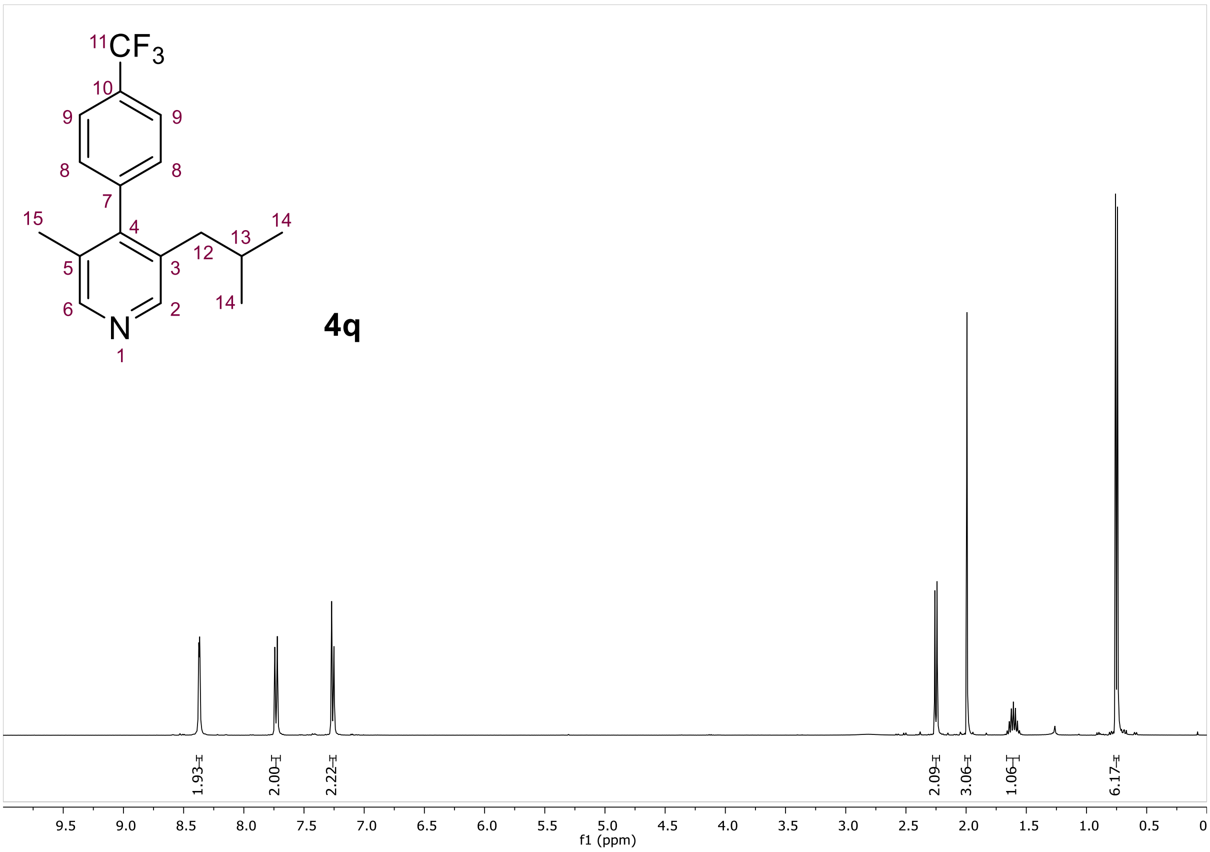

4q
1.93
2.00
2.22
2.09
3.06
1.06
6.17
9.5 9.0 8.5 8.0 7.5 7.0 6.5 6.0 5.5 5.0 4.5 4.0 3.5 3.0 2.5 2.0 1.5 1.0 0.5 0
f1 (ppm)

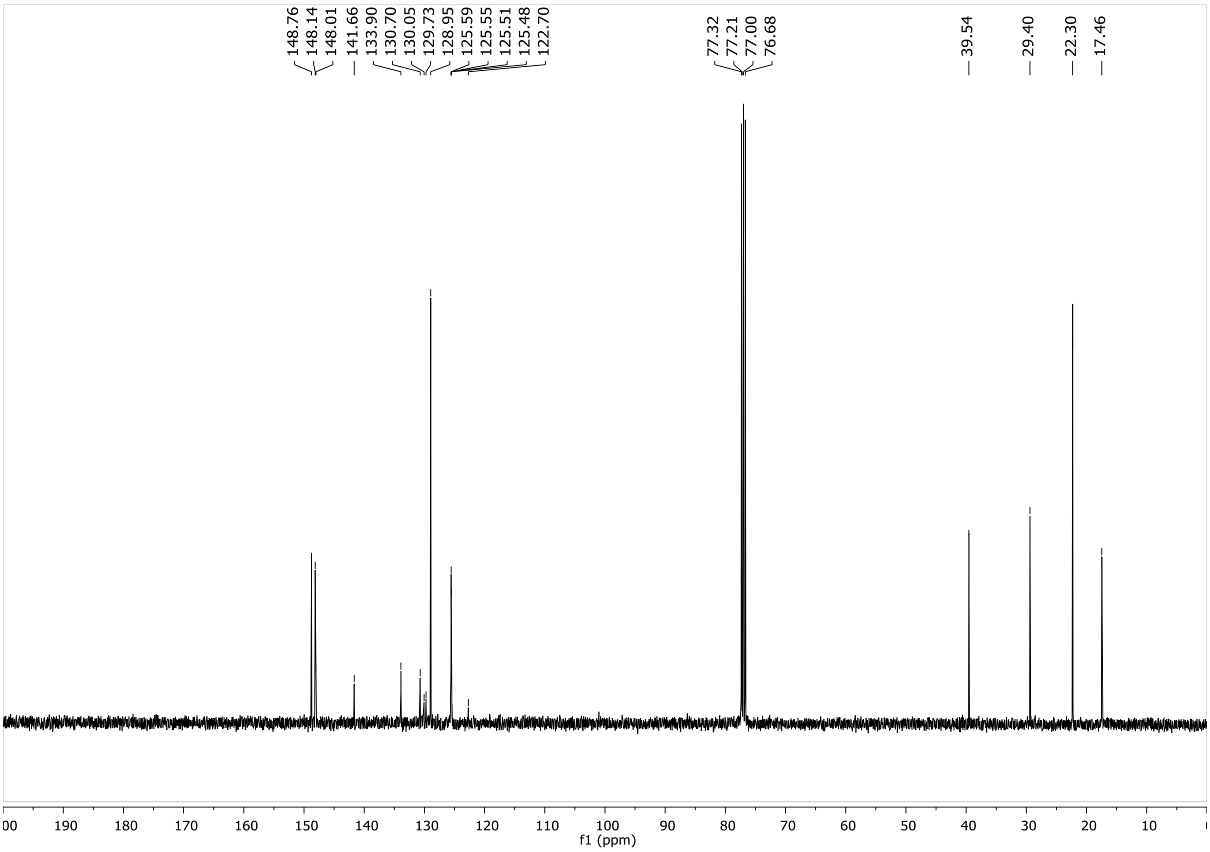

148.76
148.14
148.01
141.66
133.90
130.70
130.05
129.73
128.95
125.59
125.55
125.51
125.48
122.70
77.32
77.21
77.00
76.68
39.54
29.40
22.30
17.46
190 180 170 160 150 140 130 120 110 100 90 80 70 60 50 40 30 20 10
f1 (ppm)

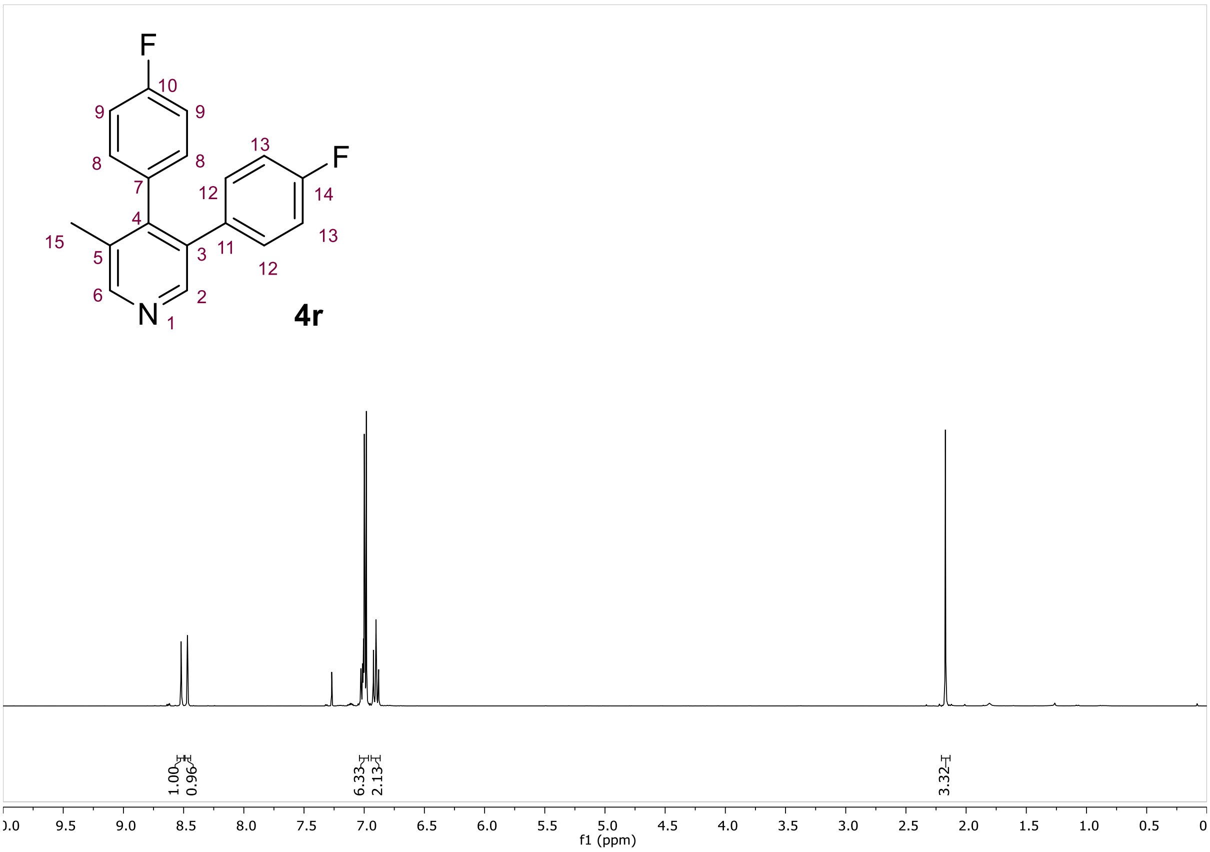

F
10
9
9
8
8
13
F
12
14
7
4
15
5
3
11
13
12
6
2
N
1
4r
1.00
0.96
6.33
2.13
3.32
f1 (ppm)

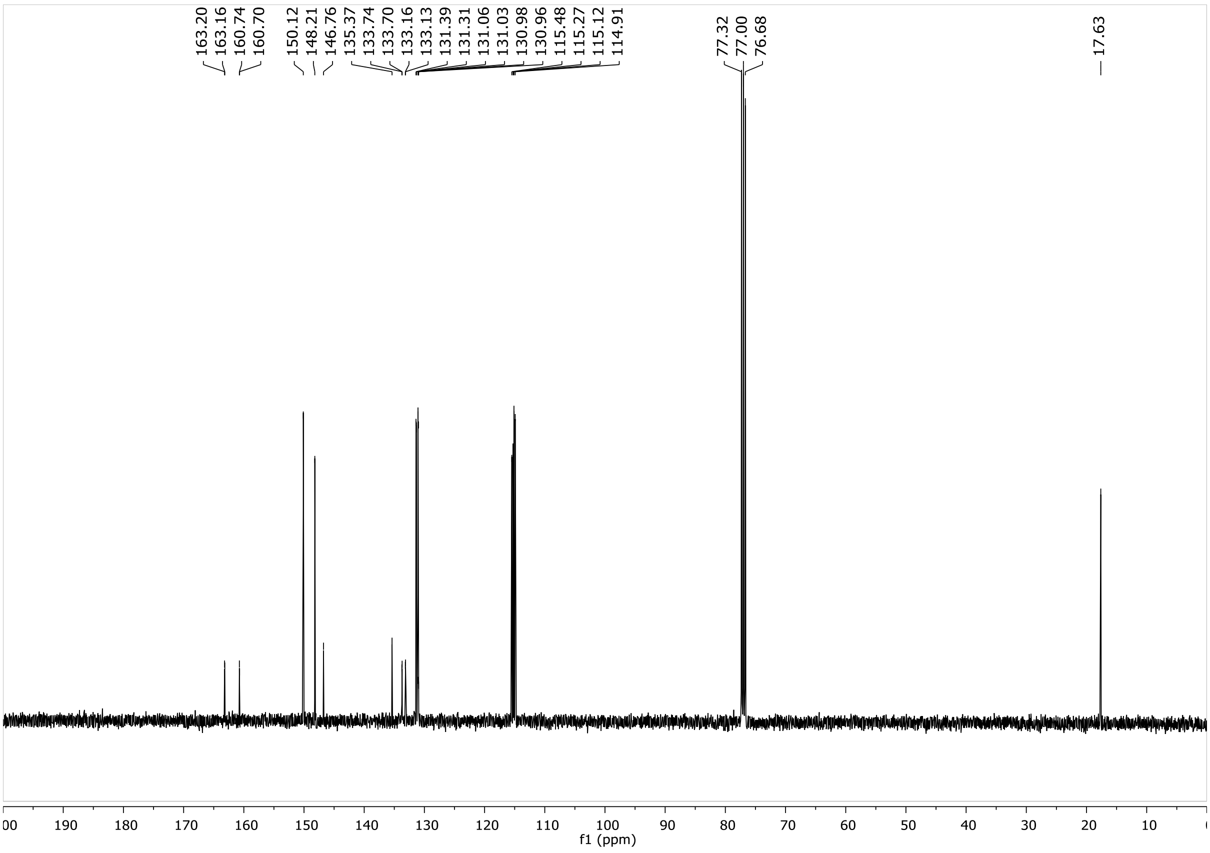

163.20
163.16
160.74
160.70
150.12
148.21
146.76
135.37
133.74
133.70
133.16
133.13
131.39
131.31
131.06
131.03
130.98
130.96
115.48
115.27
115.12
114.91
77.32
77.00
76.68
17.63
f1 (ppm)

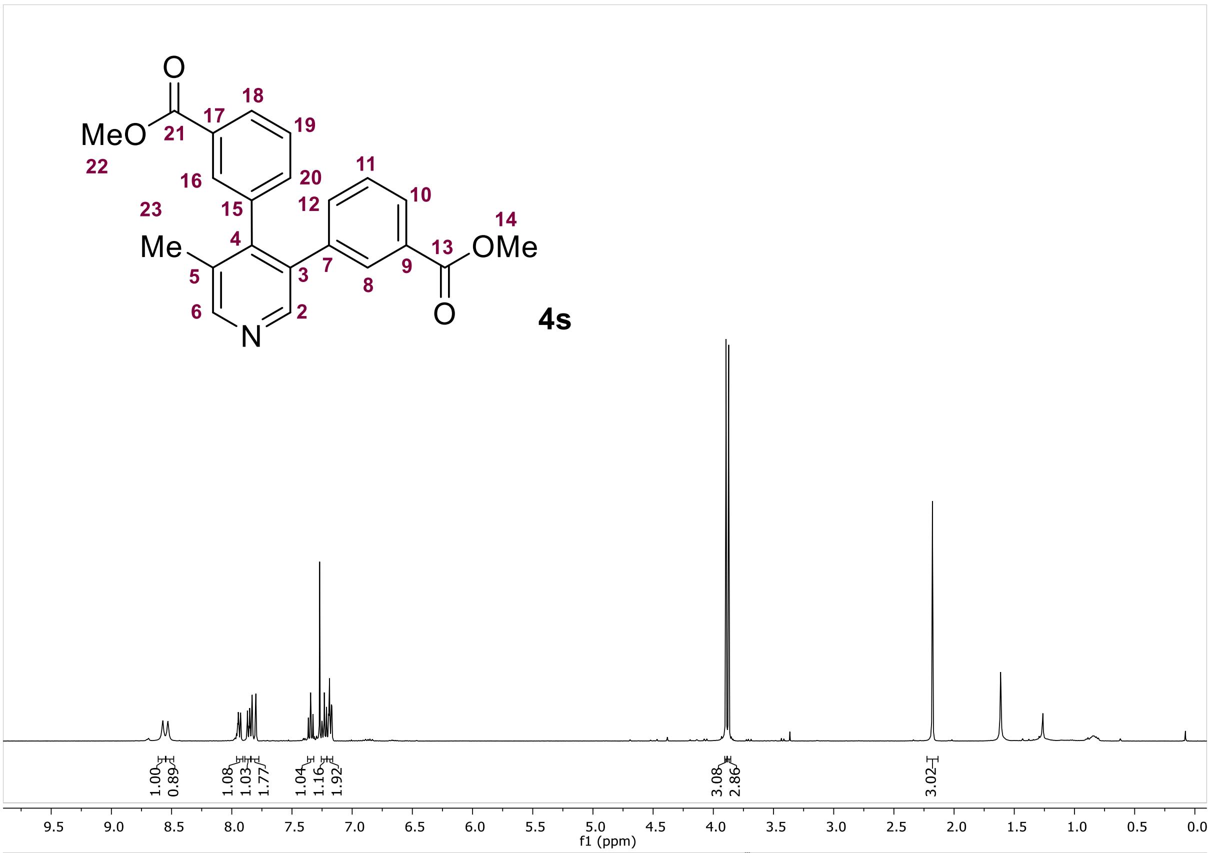
4s
MeO
OMe
Me
N
O
1.00
0.89
1.08
1.03
1.77
1.04
1.16
1.92
3.08
2.86
3.02
f1 (ppm)

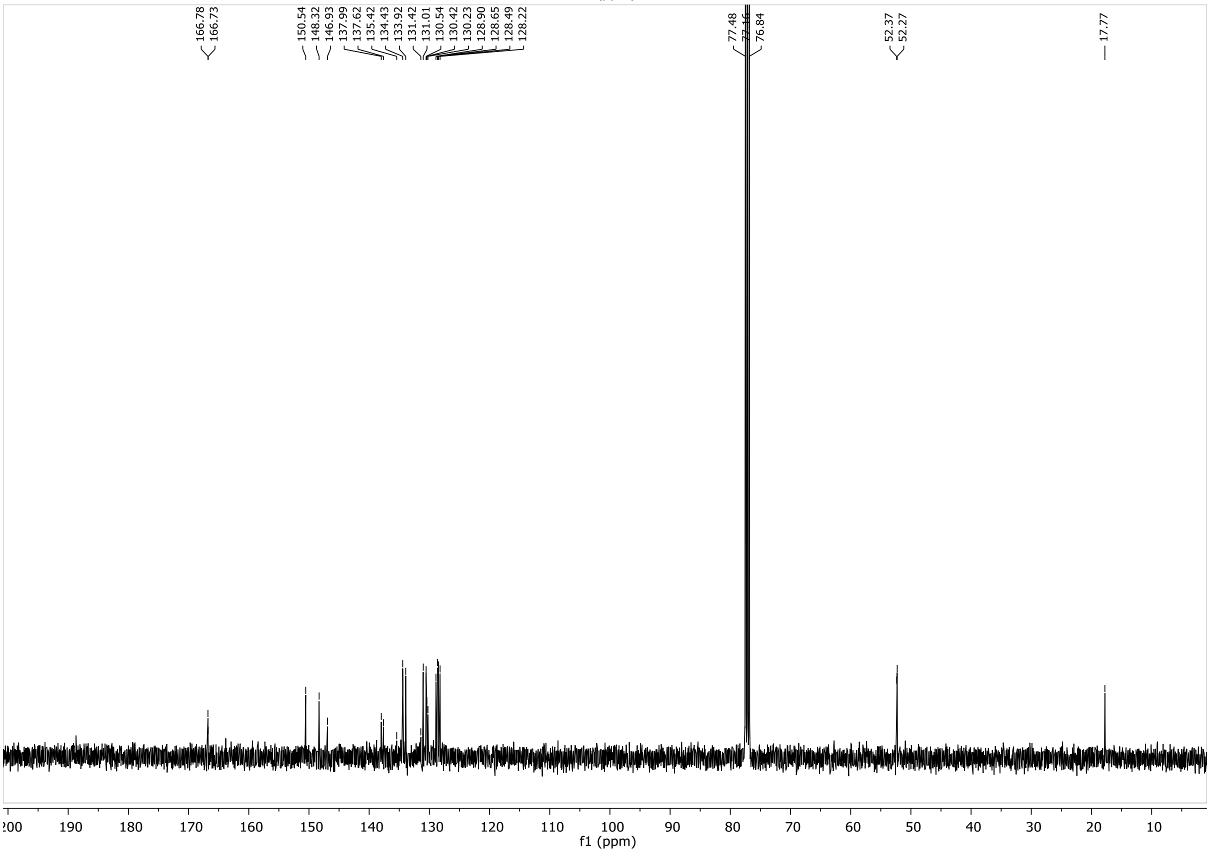
166.78
166.73
150.54
148.32
146.93
137.99
137.62
135.42
134.43
133.92
131.42
131.01
130.54
130.42
130.23
128.90
128.65
128.49
128.22
77.48
76.84
52.37
52.27
17.77
f1 (ppm)

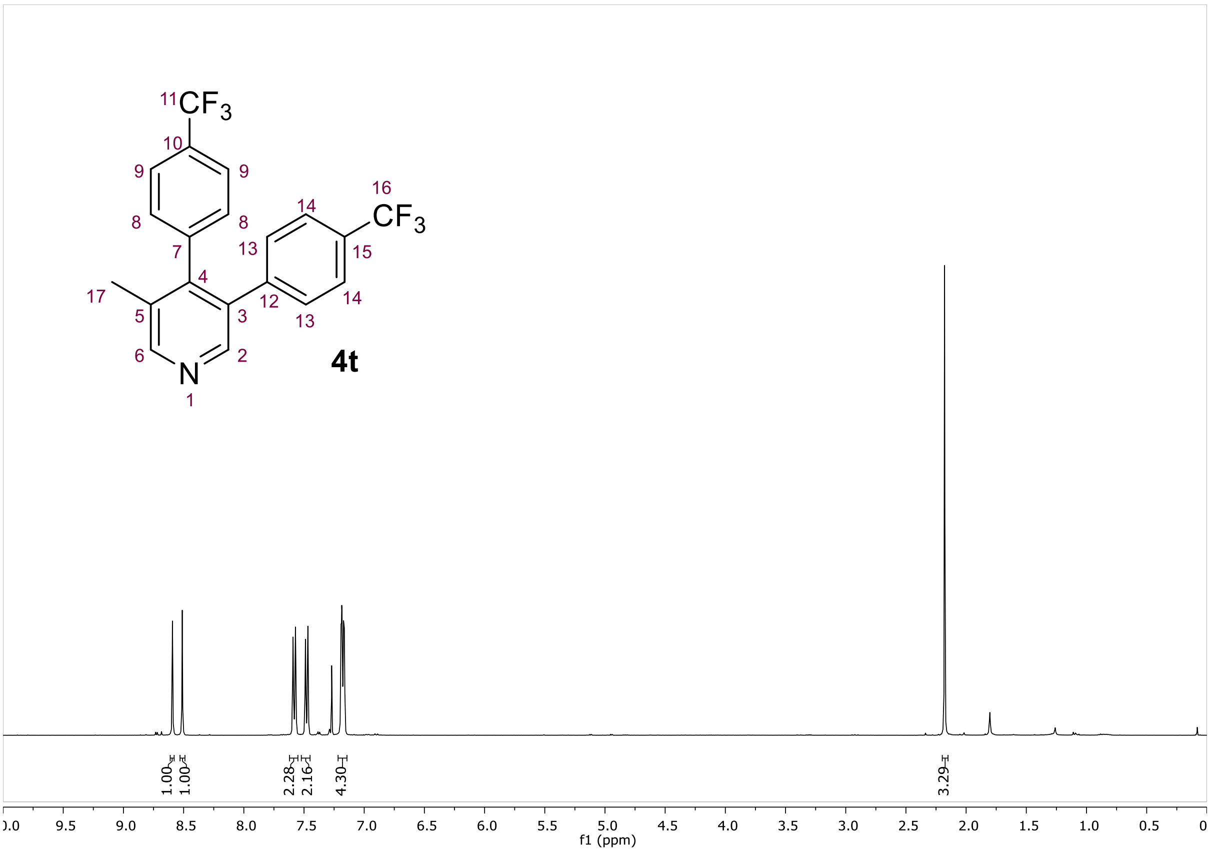
4t
1.00
1.00
2.28
2.16
4.30
3.29
f1 (ppm)

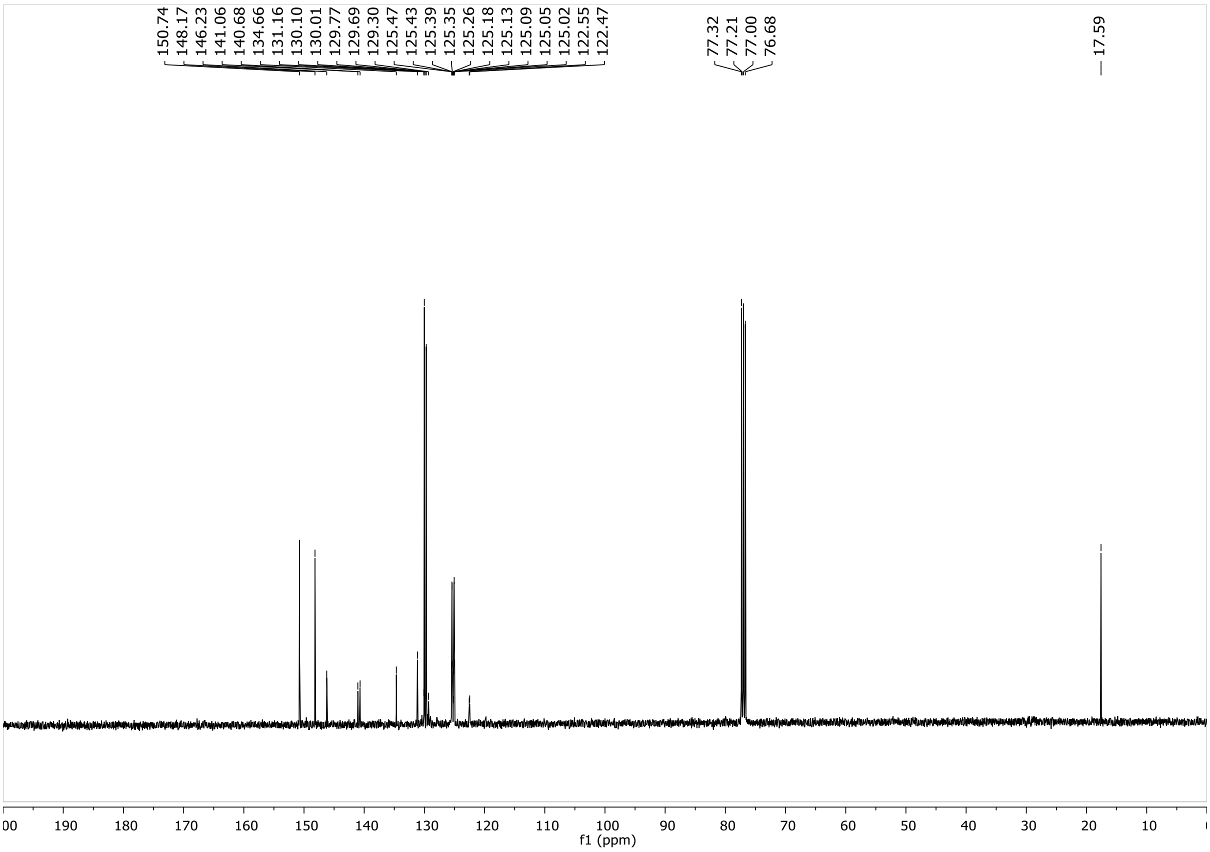
150.74
148.17
146.23
141.06
140.68
134.66
131.16
130.10
130.01
129.77
129.69
129.30
125.47
125.43
125.39
125.35
125.26
125.18
125.13
125.09
125.05
125.02
122.55
122.47
77.32
77.21
77.00
76.68
17.59
f1 (ppm)

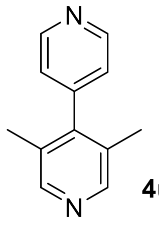

4u

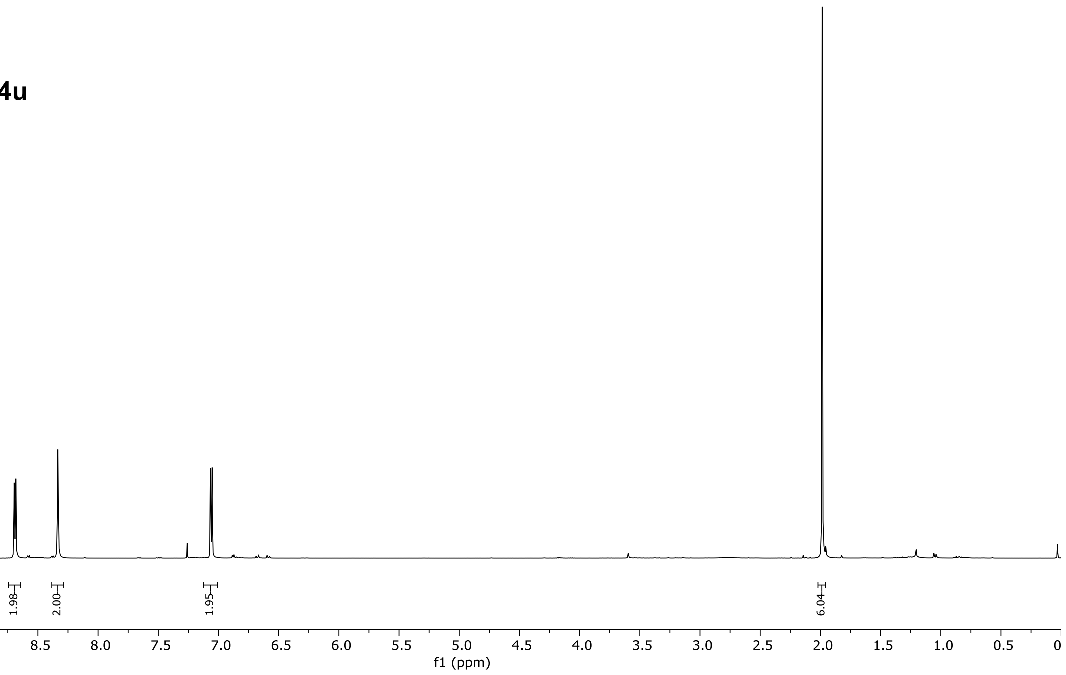

1.98
2.00
1.95
6.04
f1 (ppm)

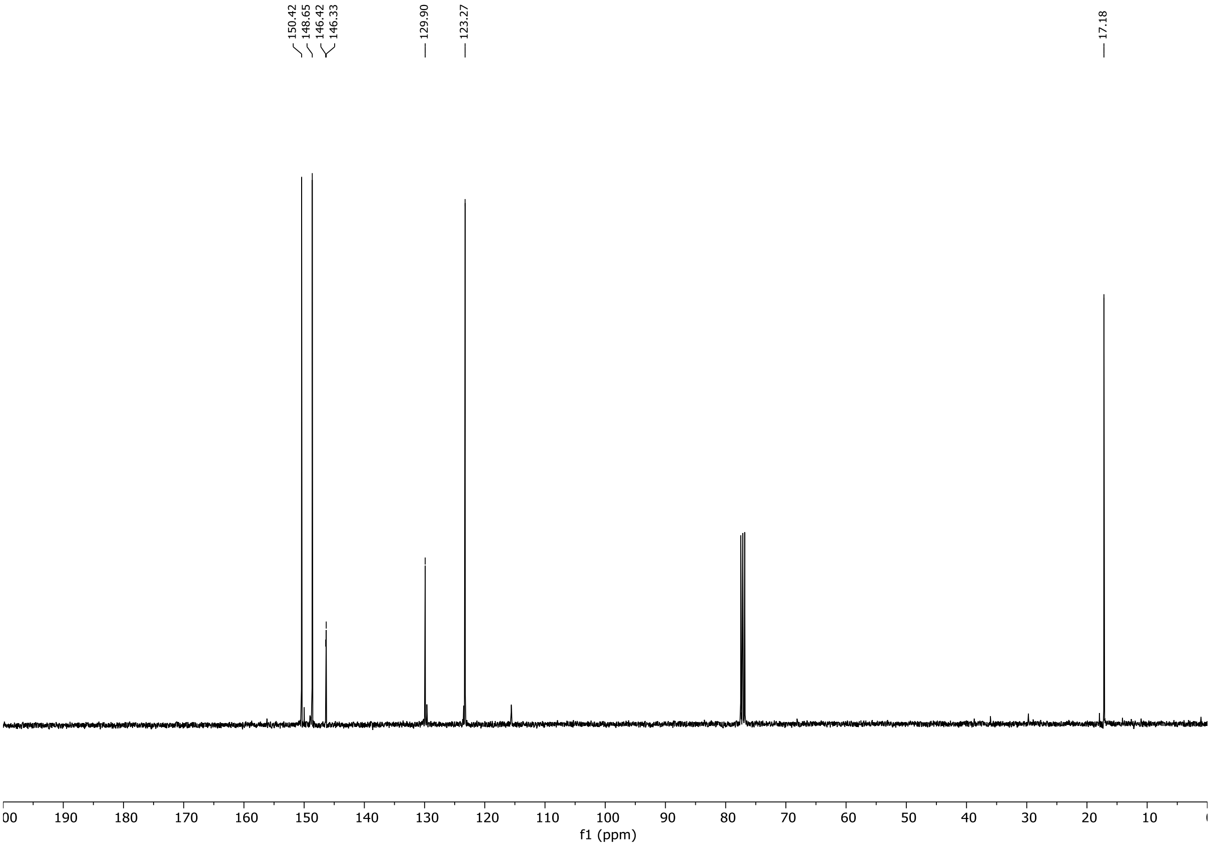

150.42
148.65
146.42
146.33
129.90
123.27
17.18
f1 (ppm)

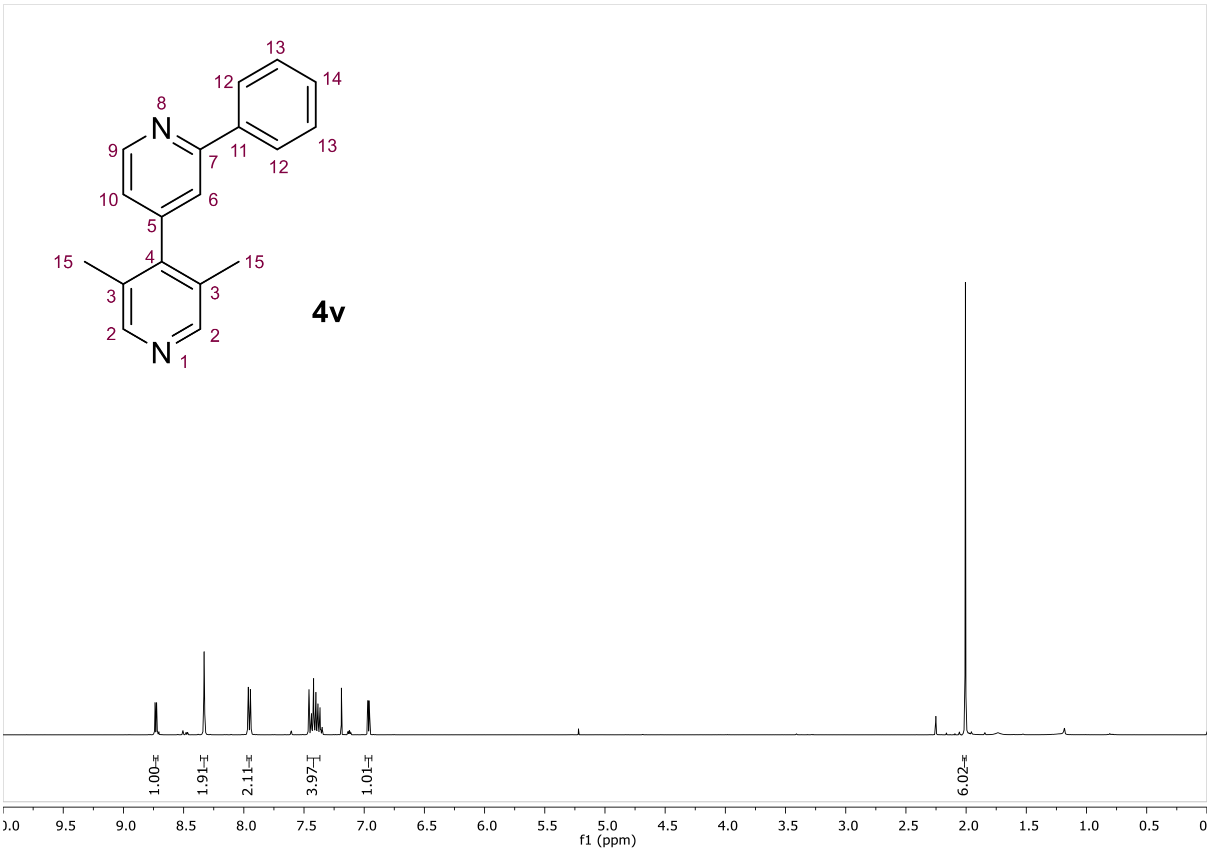
4v
13
12
14
8
N
9
11
13
7
12
10
6
5
15
4
15
3
3
2
2
N
1
1.00
1.91
2.11
3.97
1.01
6.02
f1 (ppm)

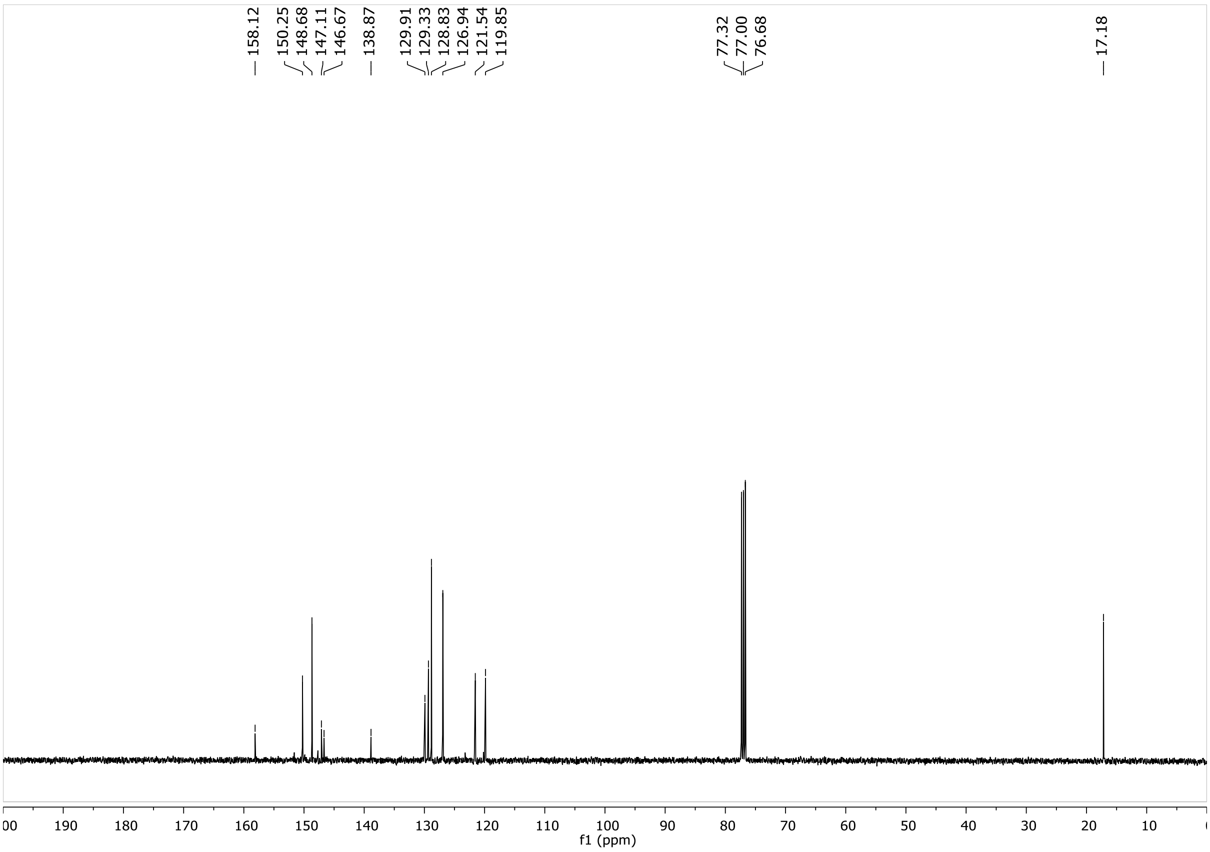
158.12
150.25
148.68
147.11
146.67
138.87
129.91
129.33
128.83
126.94
121.54
119.85
77.32
77.00
76.68
17.18
f1 (ppm)

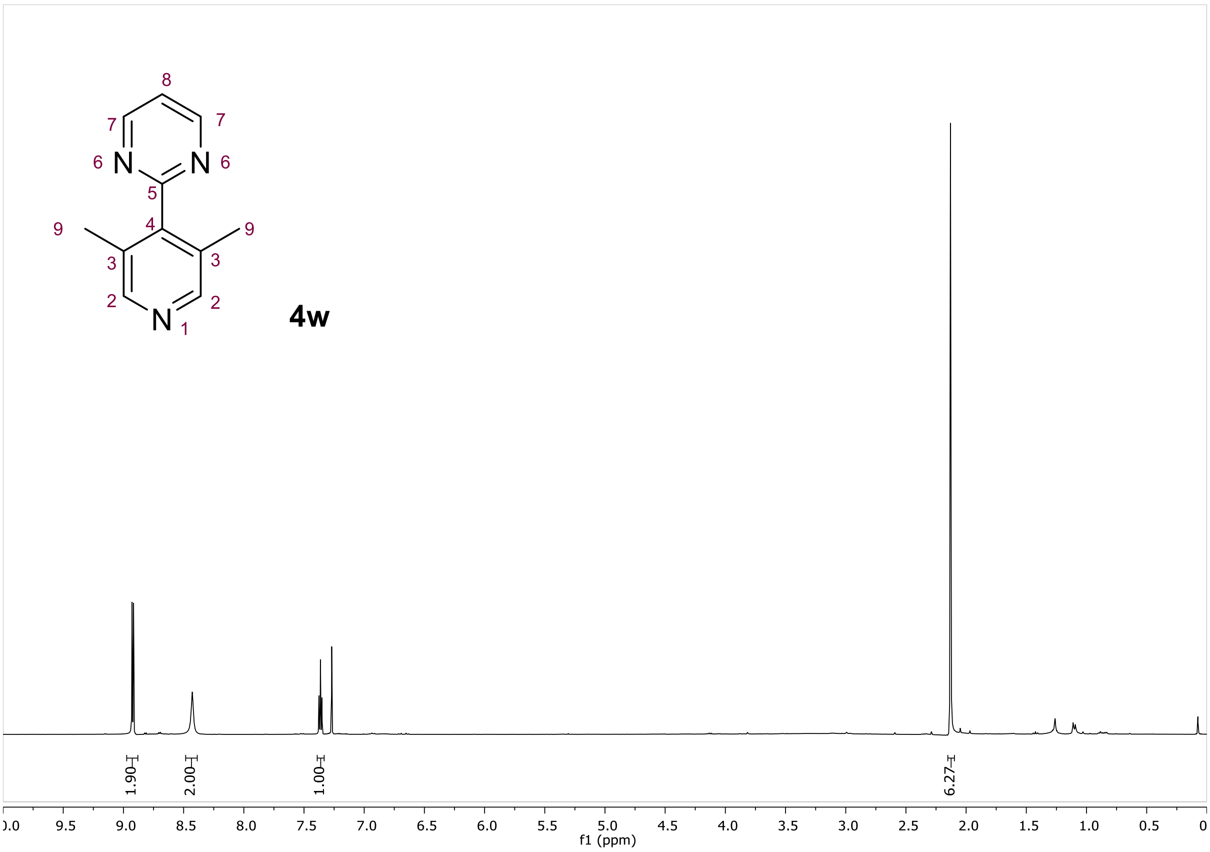
4w
1.90
2.00
1.00
6.27
f1 (ppm)

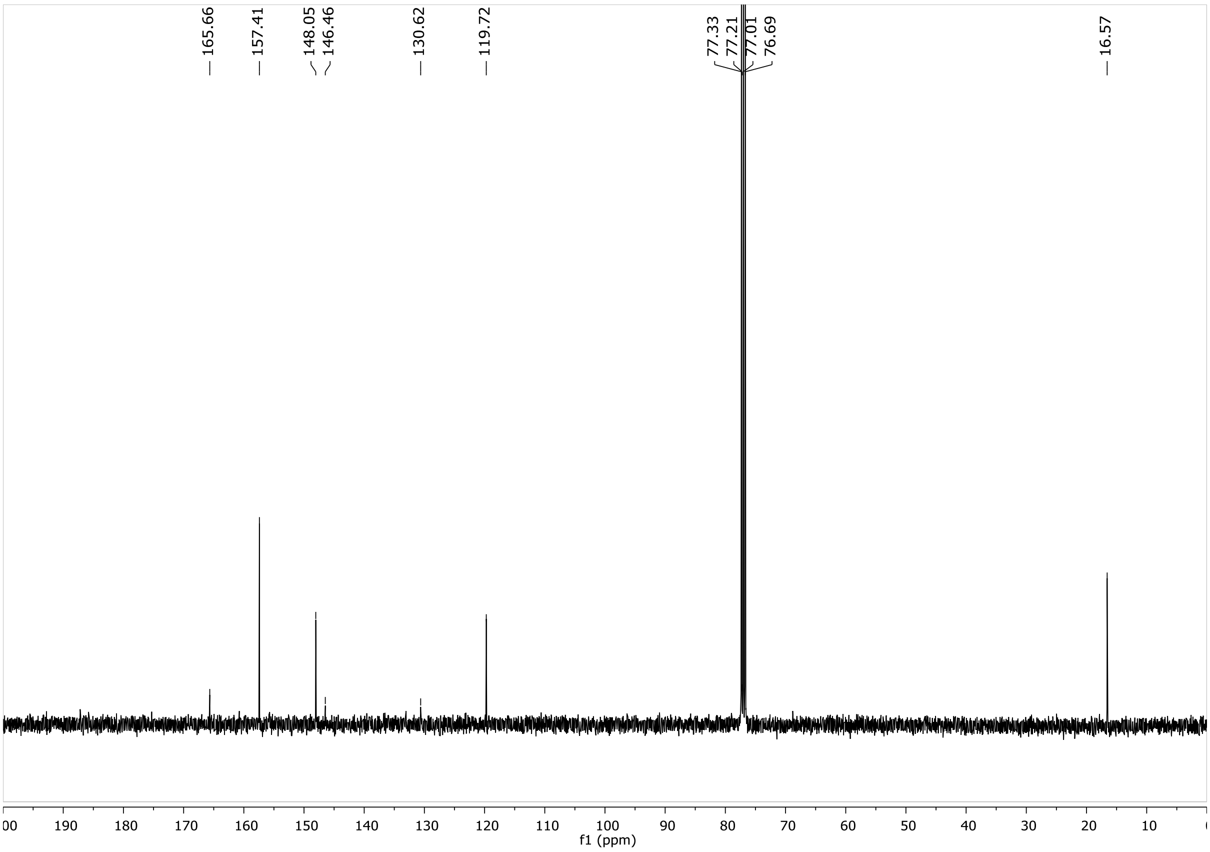
165.66
157.41
148.05
146.46
130.62
119.72
77.33
77.21
77.01
76.69
16.57
f1 (ppm)

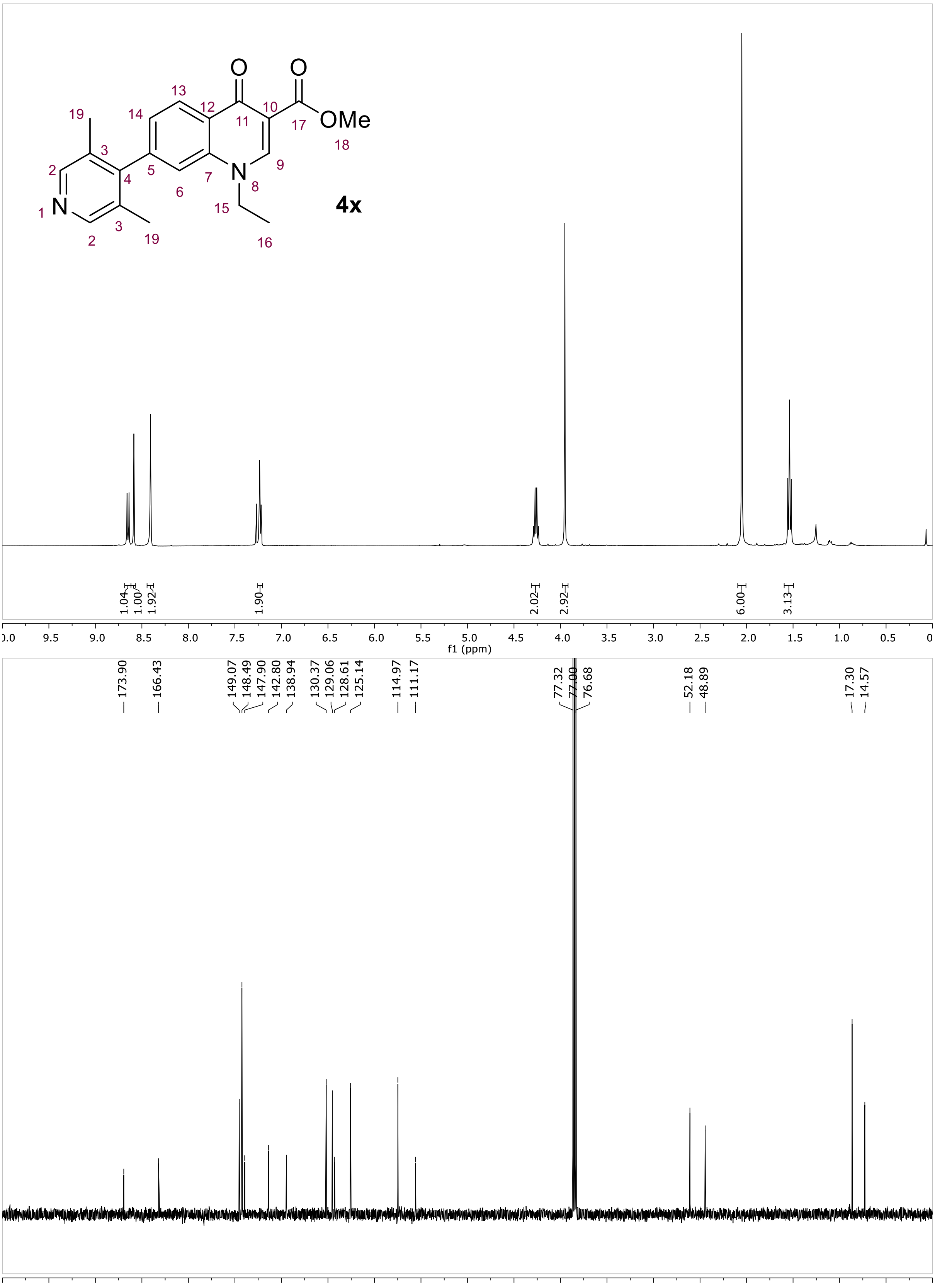
4x
OMe
1.04
1.00
1.92
1.90
2.02
2.92
6.00
3.13
f1 (ppm)
173.90
166.43
149.07
148.49
147.90
142.80
138.94
130.37
129.06
128.61
125.14
114.97
111.17
77.32
77.00
76.68
52.18
48.89
17.30
14.57
f1 (ppm)

Mechanistic Experiments

The following experiments were undertaken in order to better understand the mechanism:

1) Substrate **2i** was subjected to the standard conditions for the reaction in the absence of rhodium which led to no product formation and complete recovery of pyridine **1i**.

Experiment 1

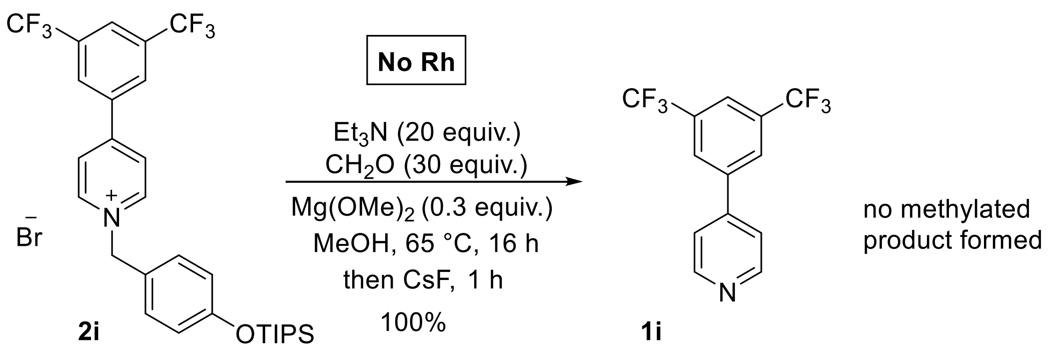

2) Substrate **2i** was subjected to the standard conditions for the reaction in the absence of paraformaldehyde which led to no product formation and high recovery of pyridine **1i**.

Experiment 2

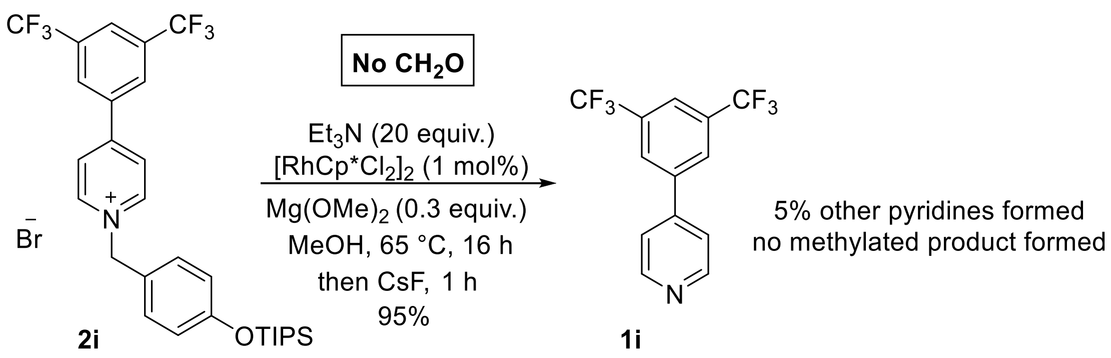

These experiments indicate that both rhodium and paraformaldehyde are required for methylated product formation.

Deuterium Labelling Studies

The doubly methylated product **3i** was prepared and re-subjected to the methylation reaction conditions using deuterated formaldehyde.

Experiment 3

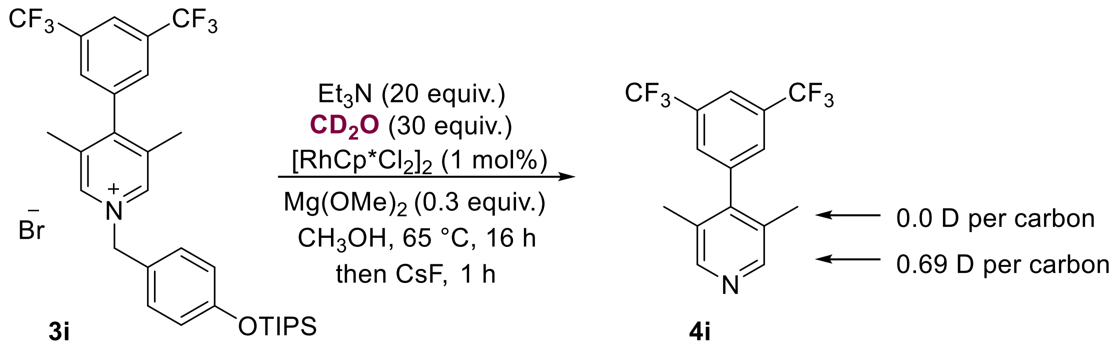

The ^{19}F NMR spectrum of the crude reaction reveals about 30 % degradation to by-products. The ^{1}H NMR and the ^{2}H NMR spectra of the isolated pure product **4i** reveal 0.69 deuterium incorporated at each C-2 position and no deuterium at the C-3 methyl position.

These findings suggest the following:

- Formaldehyde is oxidised thus forming a rhodium hydride species. This rhodium hydride species can add reversibly at the C-2 position of the pyridinium salt.
- The methyl at the C-3 position is installed irreversibly.
- Extended reaction times will lead to product degradation.

The product **3i** was also subjected to basic conditions in the presence of CH_3OD and deuteration was observed at the C-2 and C-3 methyl positions; this indicated both these positions are exchanging with a protic solvent, presumably via a deprotonation/reprotonation mechanism.

Experiment 4

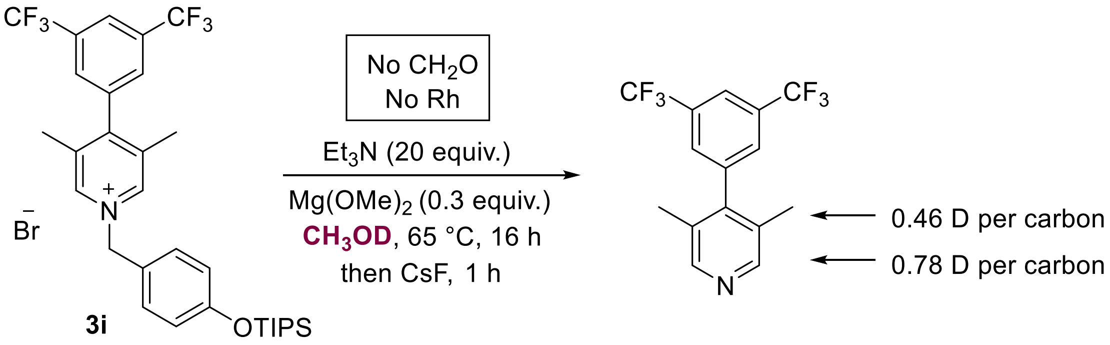

Furthermore, substrate **2i** was subjected to the reaction conditions using different permutations of deuterated methanol and paraformaldehyde and gave differing levels of deuterium incorporations.

Experiment 5

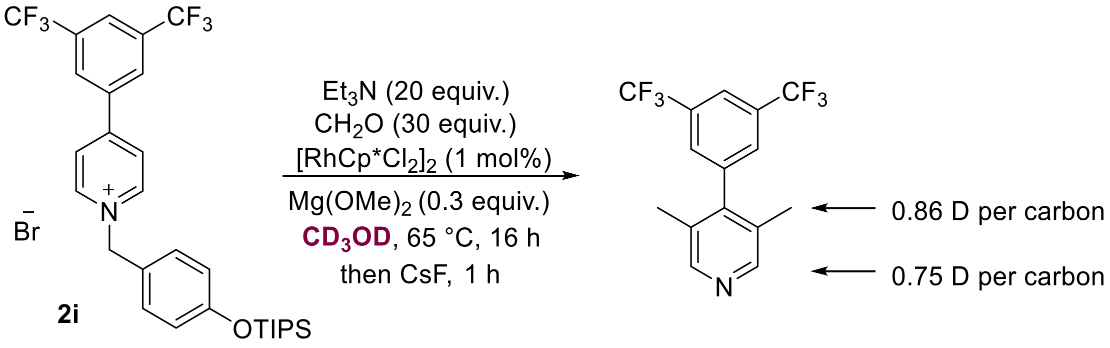

Experiment 6

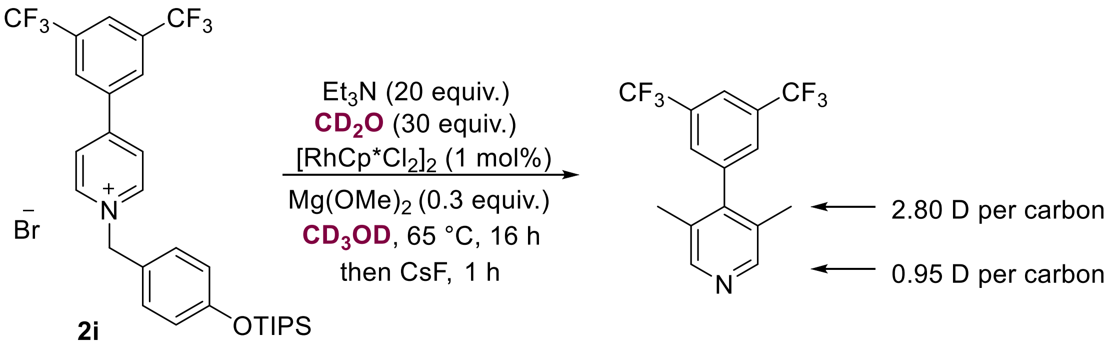

Experiment 7

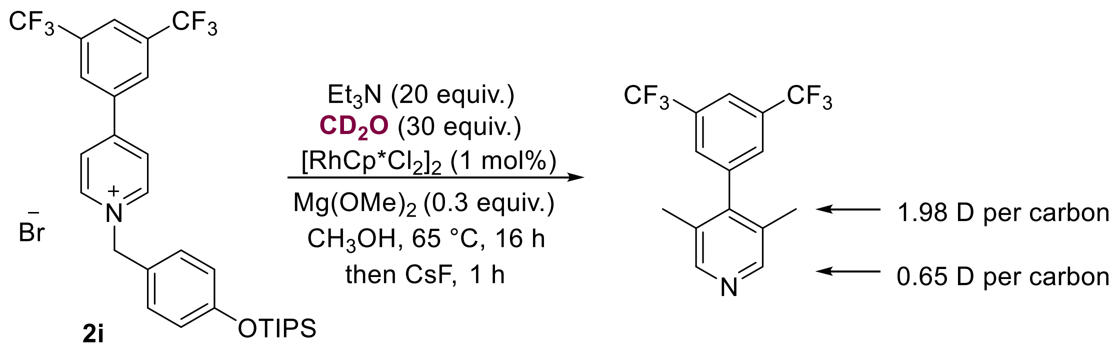

Experiment 8

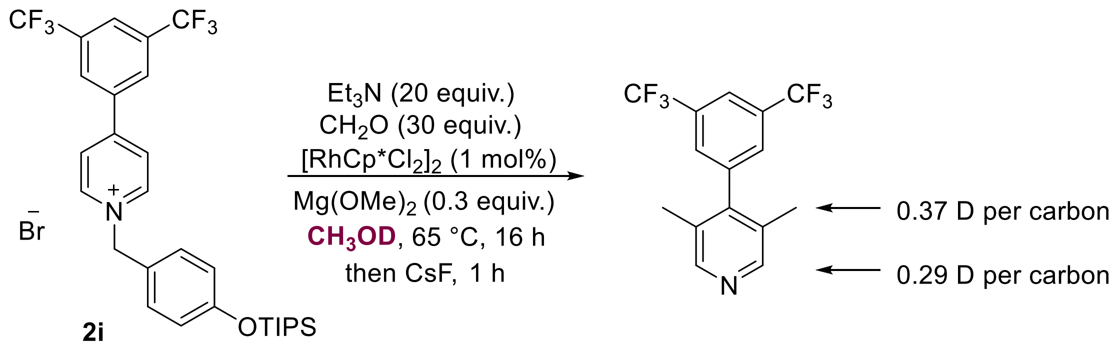

Experiment 9

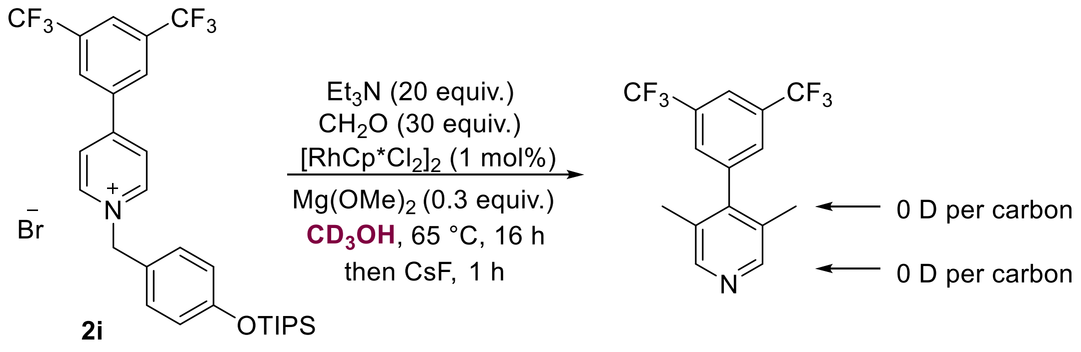

Experiment 10

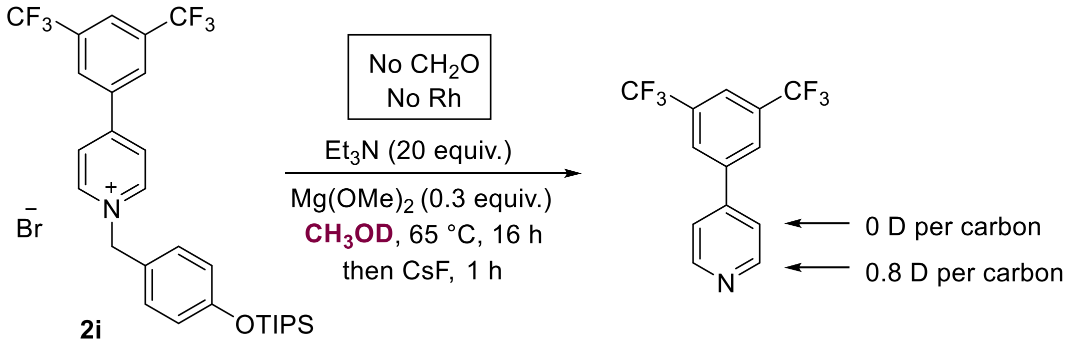

Experiment 11

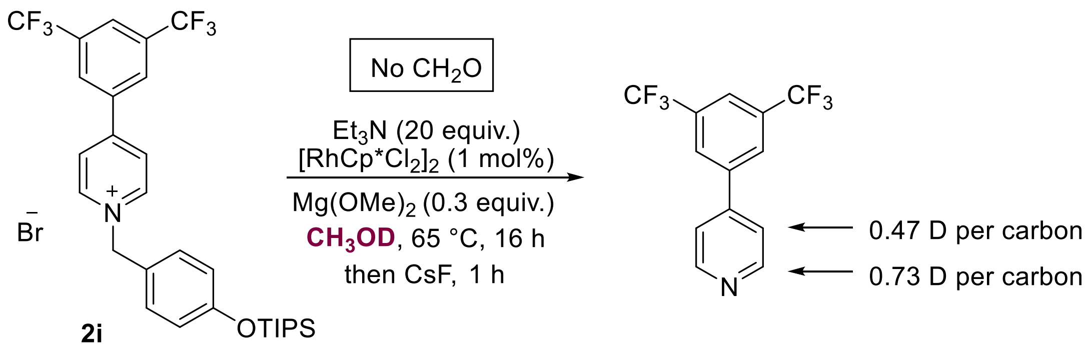

Experiment 12

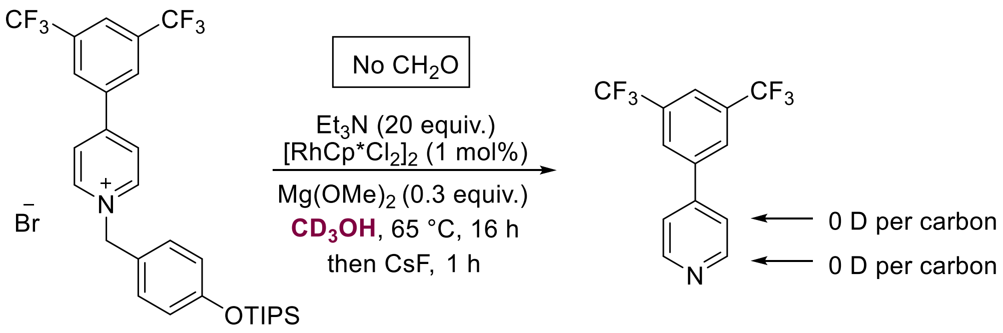

These studies lead to the following conclusions:

1) Rhodium and paraformaldehyde are needed for product formation (Exp 1/2)
2) Rhodium oxidises both paraformaldehyde and methanol in the reaction (Exp 10/11). In experiment 11, D incorporation at C-3 derives from reversible Rh-H addition to C-2 followed by reversible reaction of the enamine with the protic solvent at C-3. The Rh-H itself can only derive from CH_3OD oxidation because there is no formaldehyde present.
3) The newly installed methyl groups primarily derive from the paraformaldehyde (Exp 6-8)
4) The substrates **2i** and product **3i** are deprotonated and exchange with the solvent at both C-2 and C-3 methyl positions (Exp 4).

Experiment 3

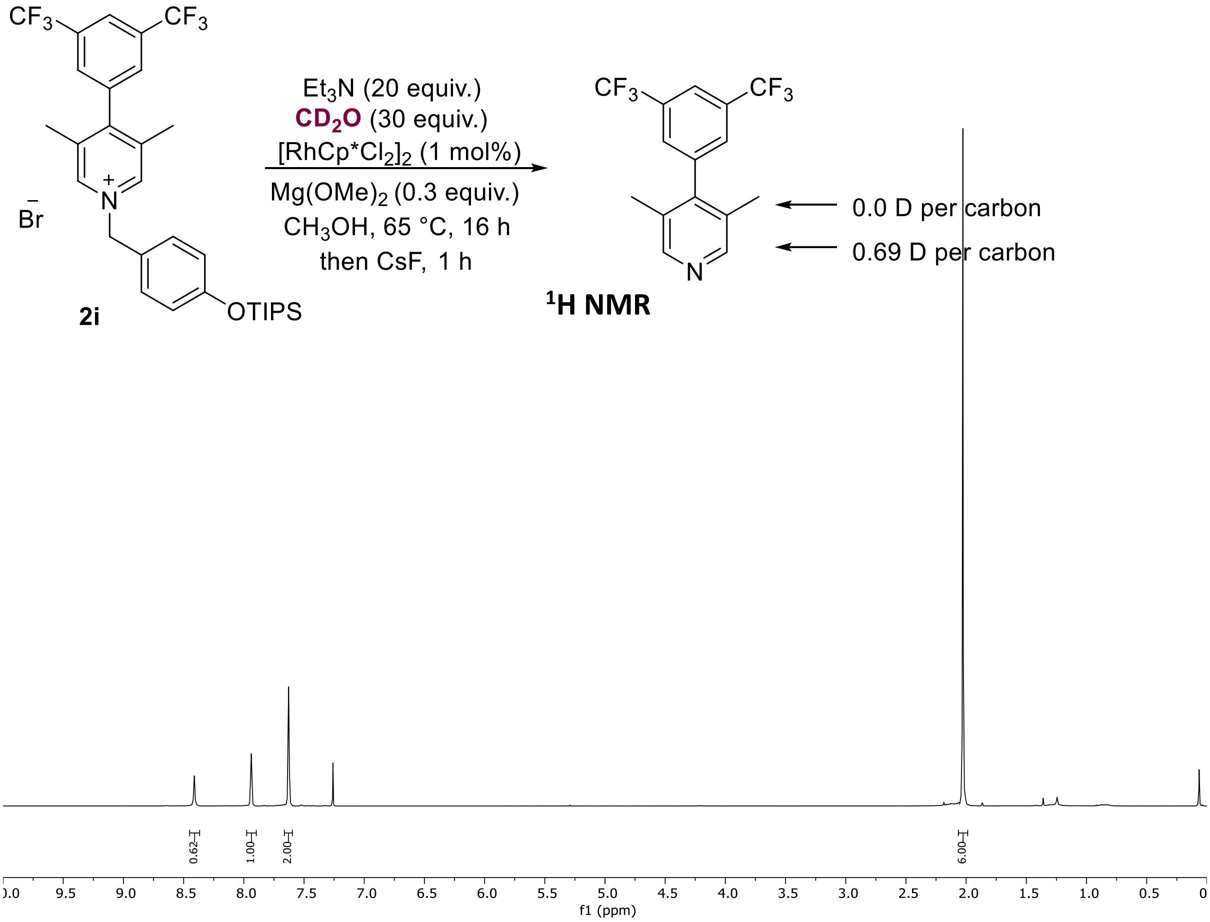

^{2}H NMR

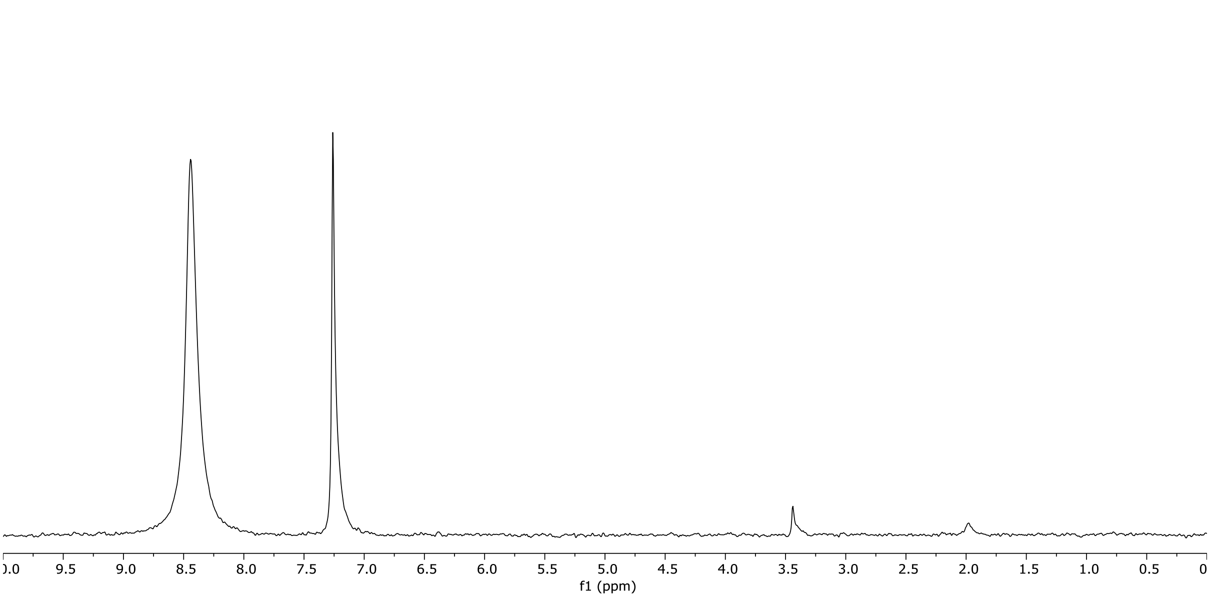

Experiment 4

^{1}H NMR

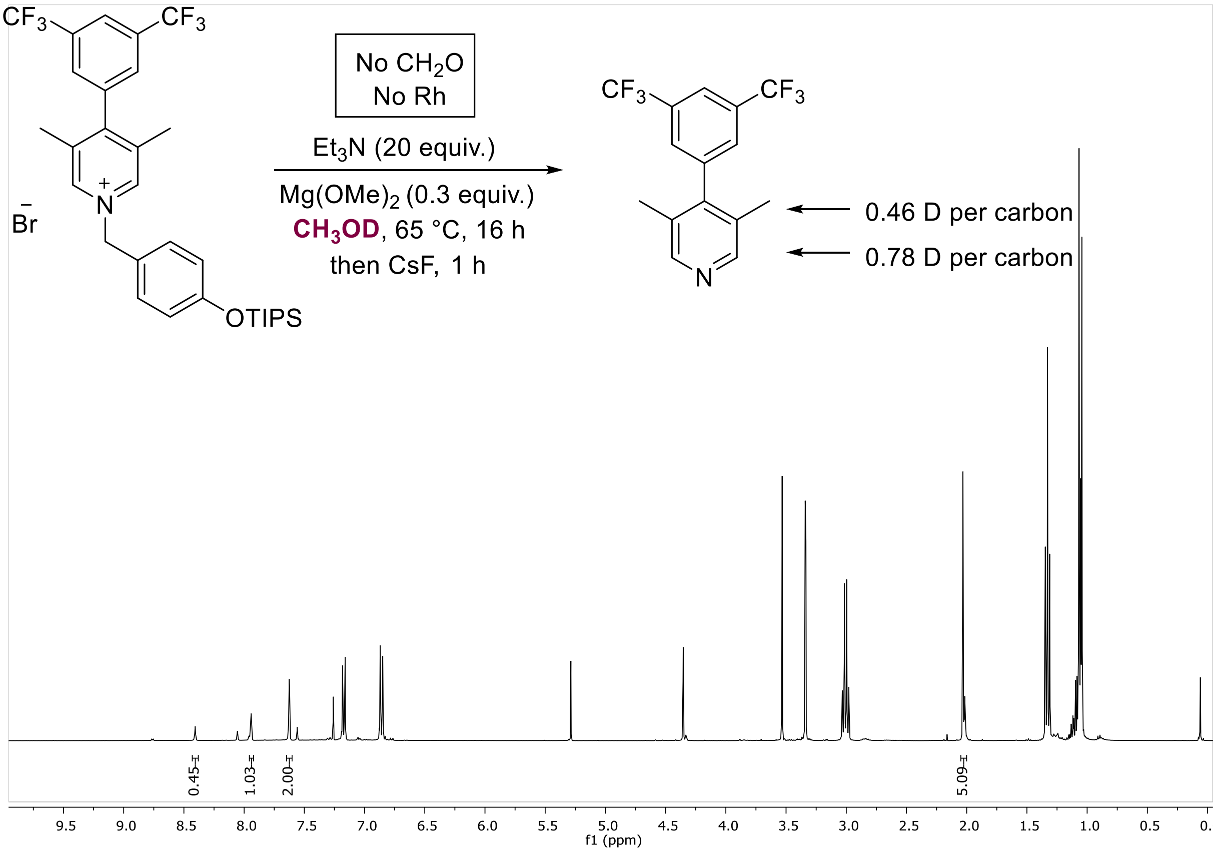

^{2}H NMR

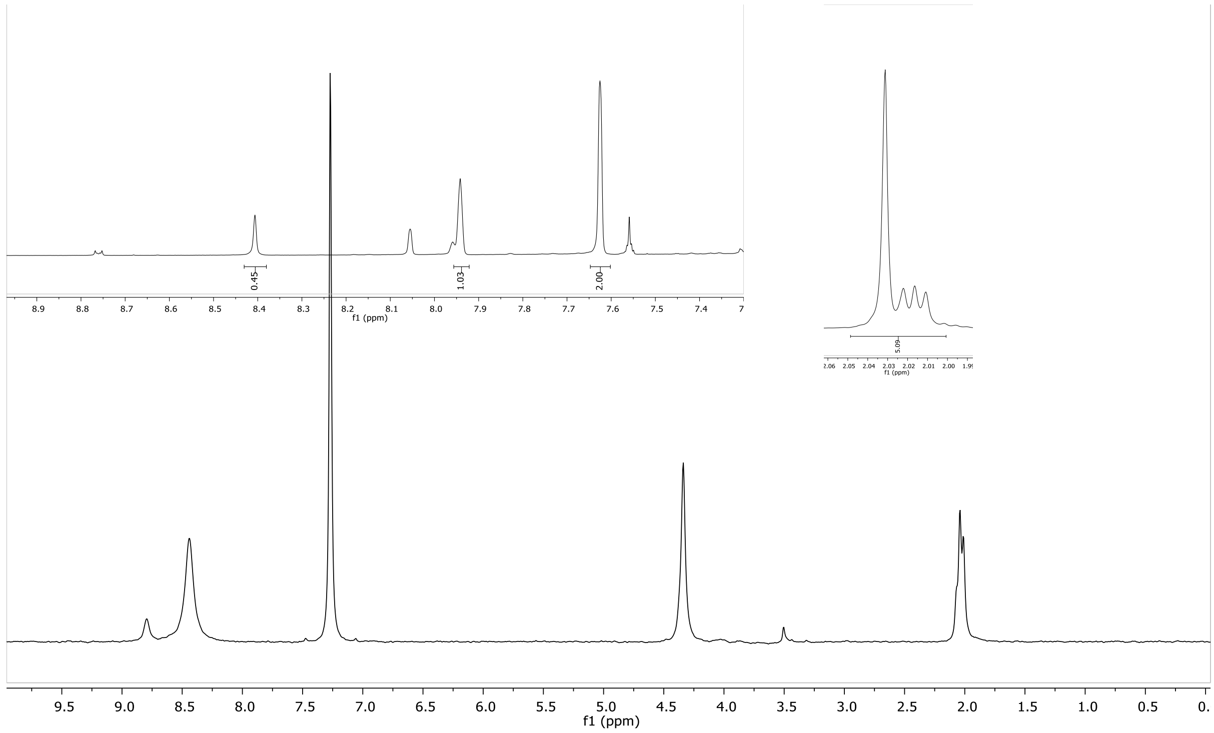

Experiment 5

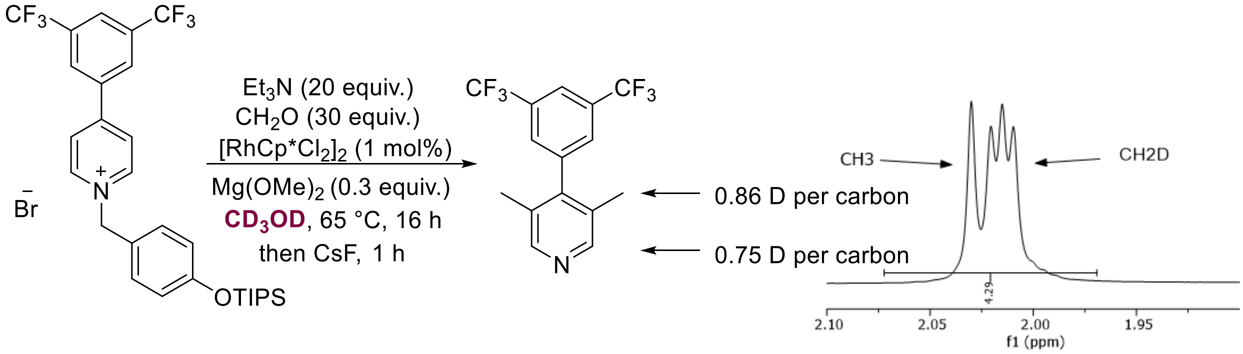

^{1}H NMR

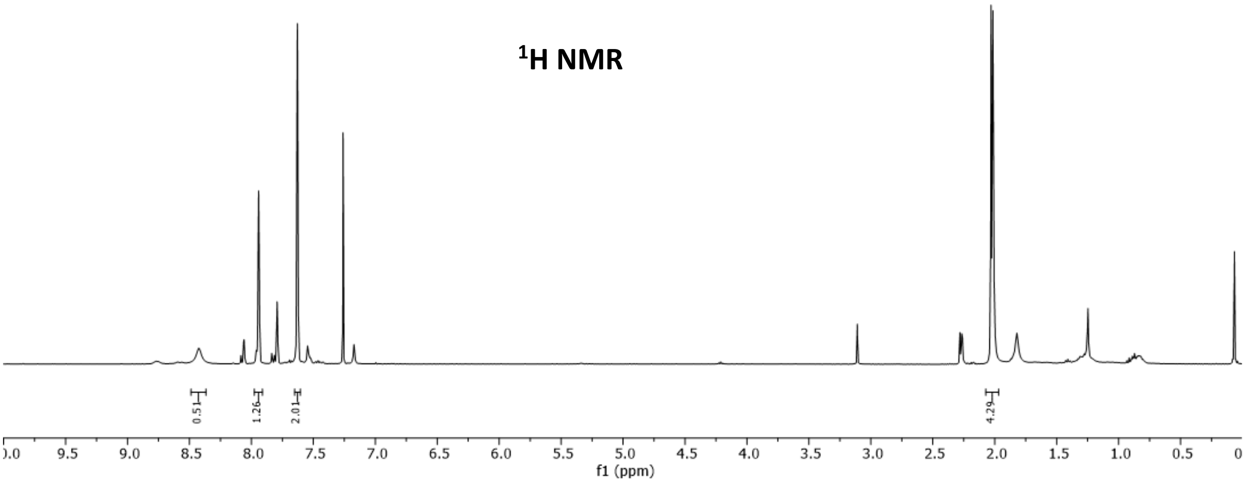

^{2}H NMR

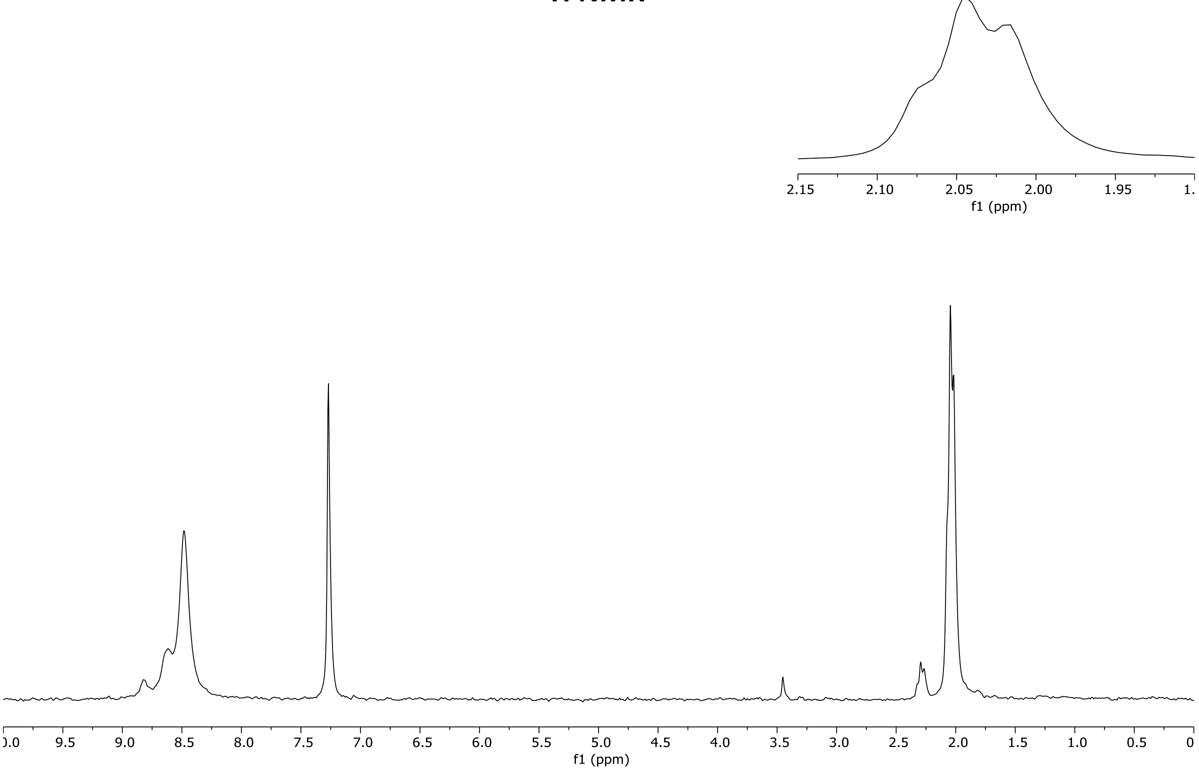

Experiment 6

^{1}H NMR

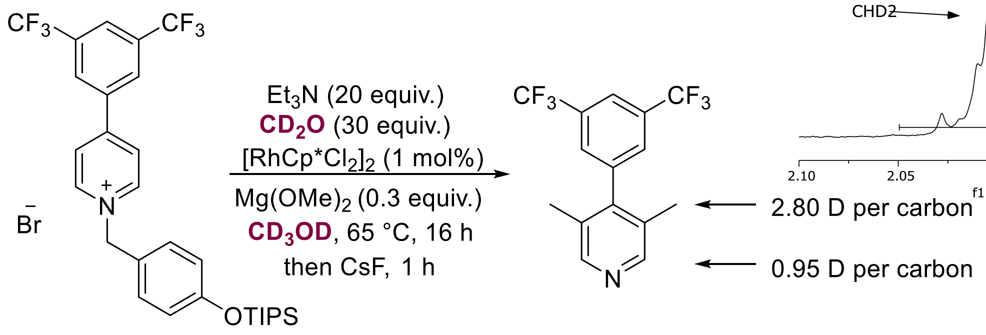

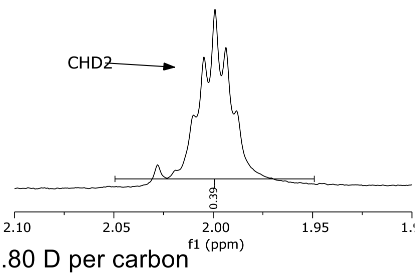

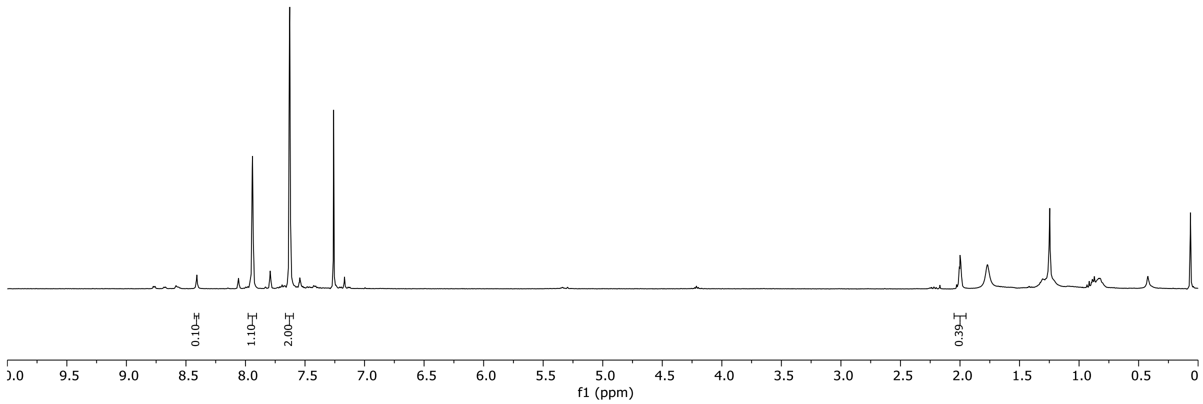

^{2}H NMR

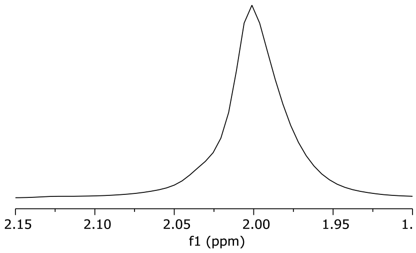

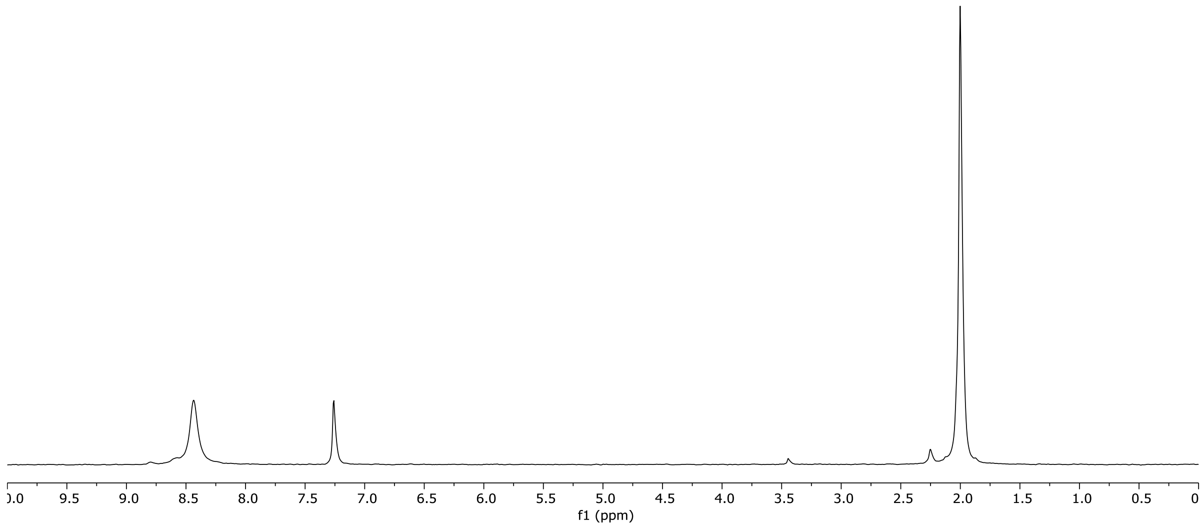

Experiment 7

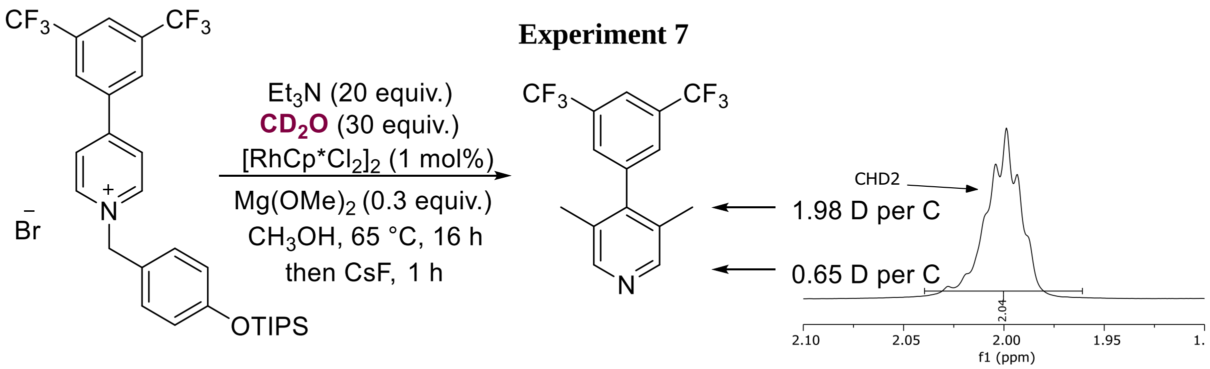

^{1}H NMR

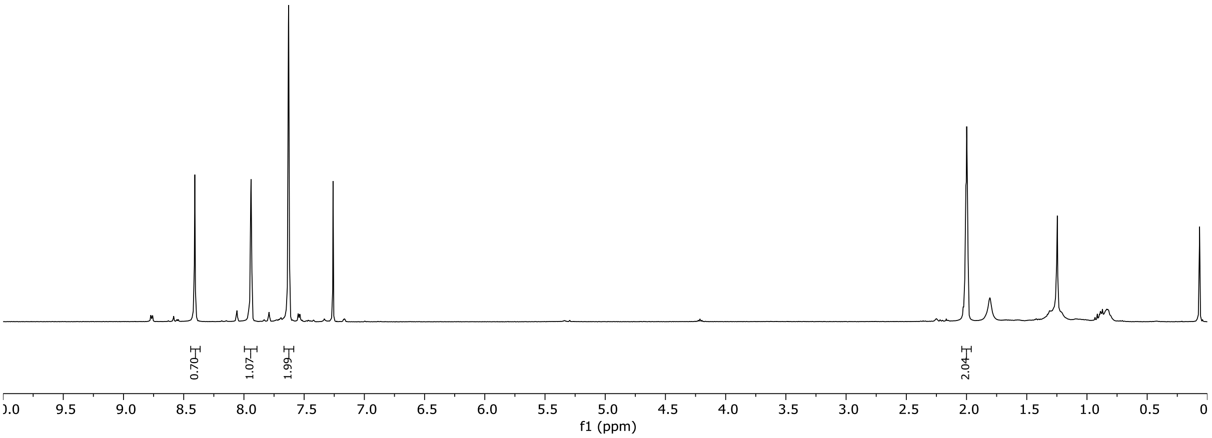

^{2}H NMR

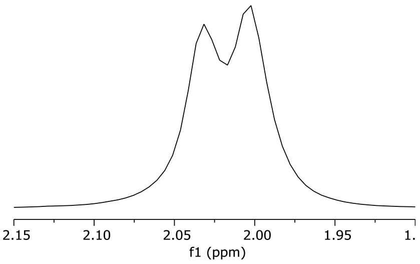

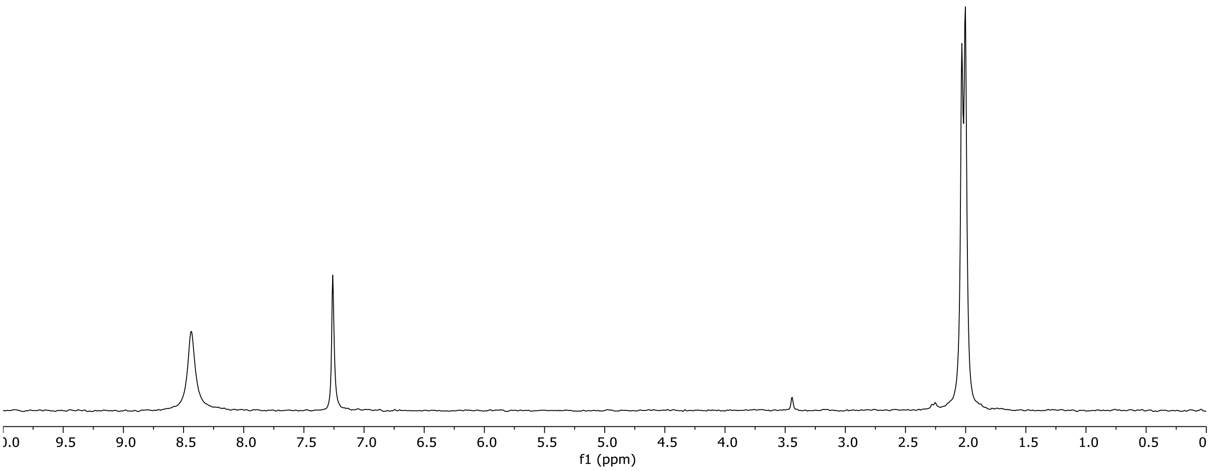

Experiment 8

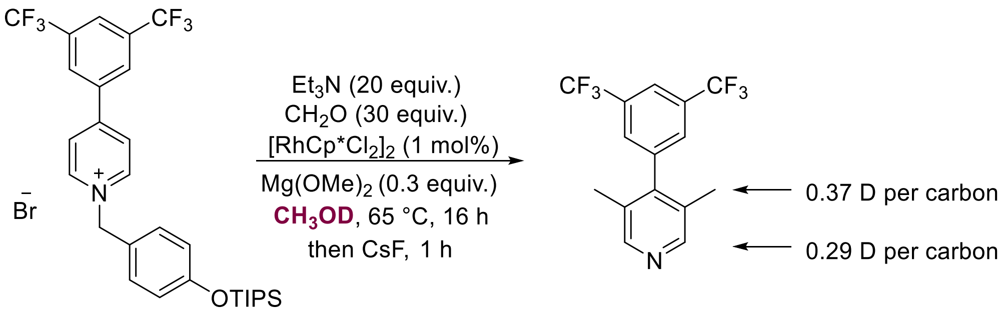

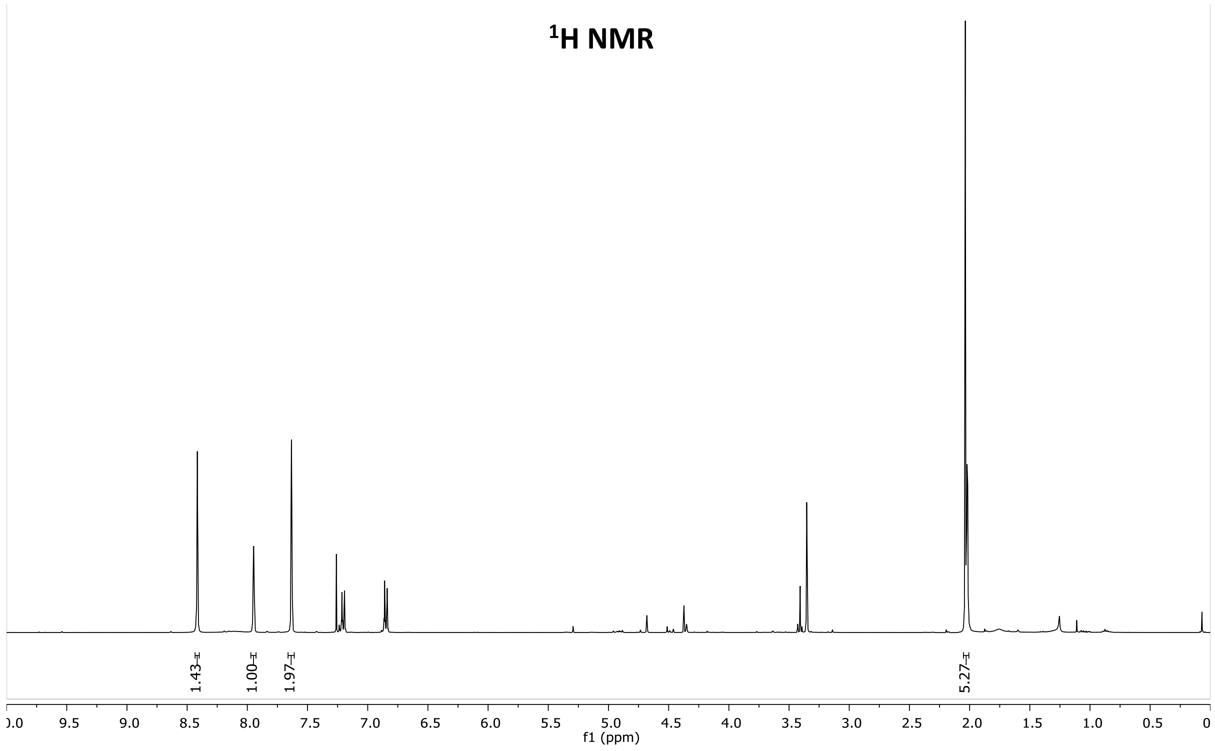

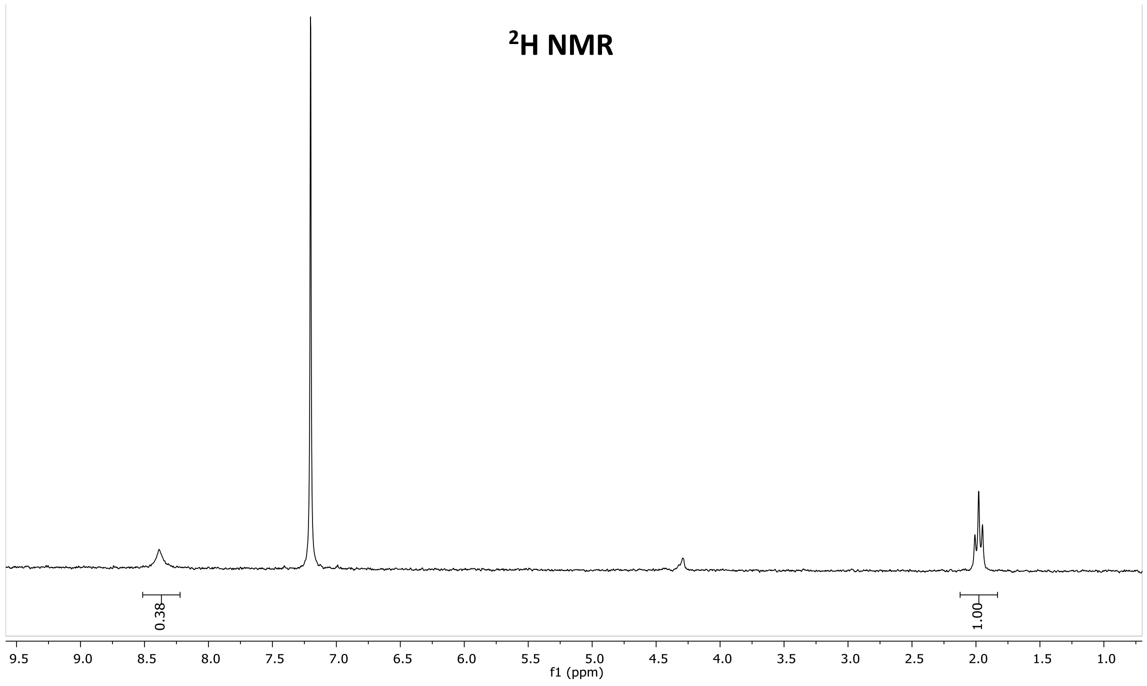

Experiment 9

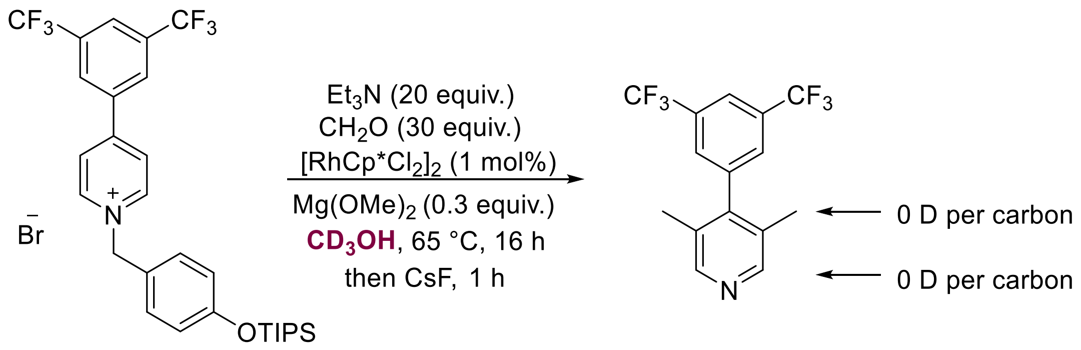

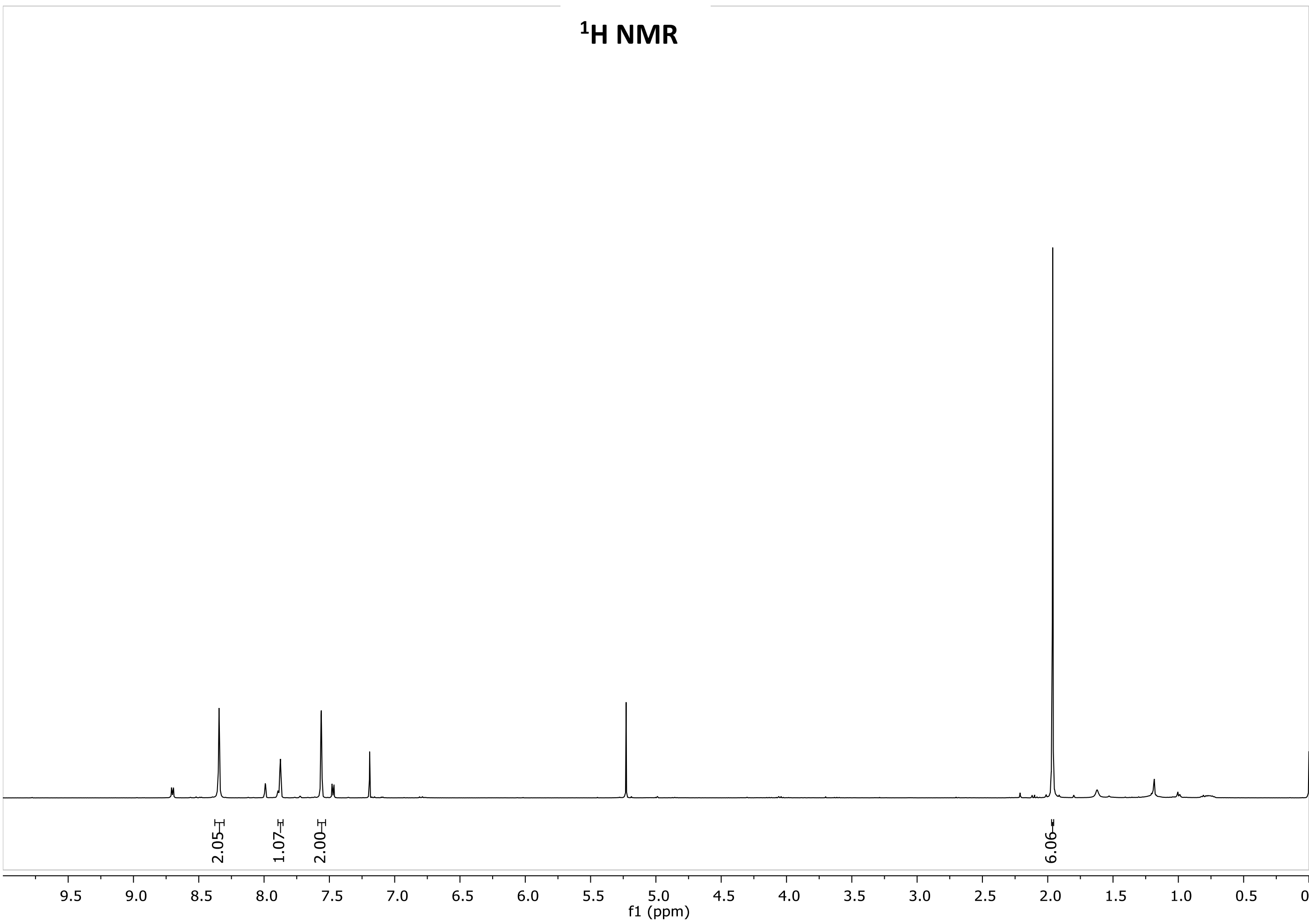

Experiment 10

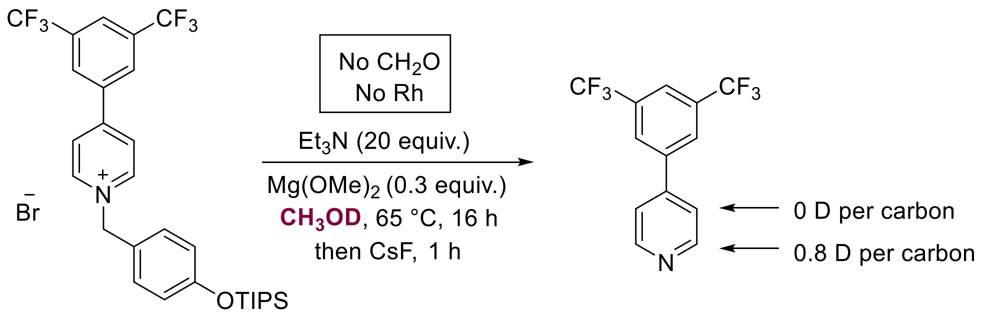

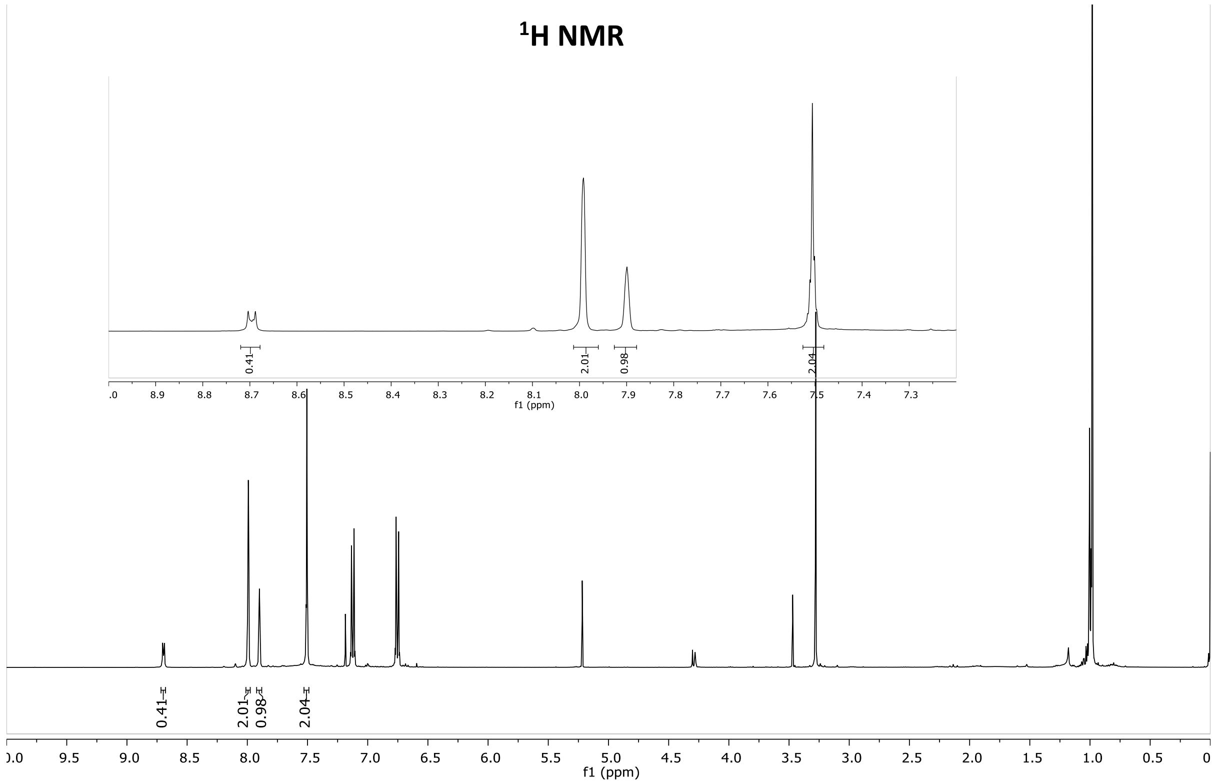

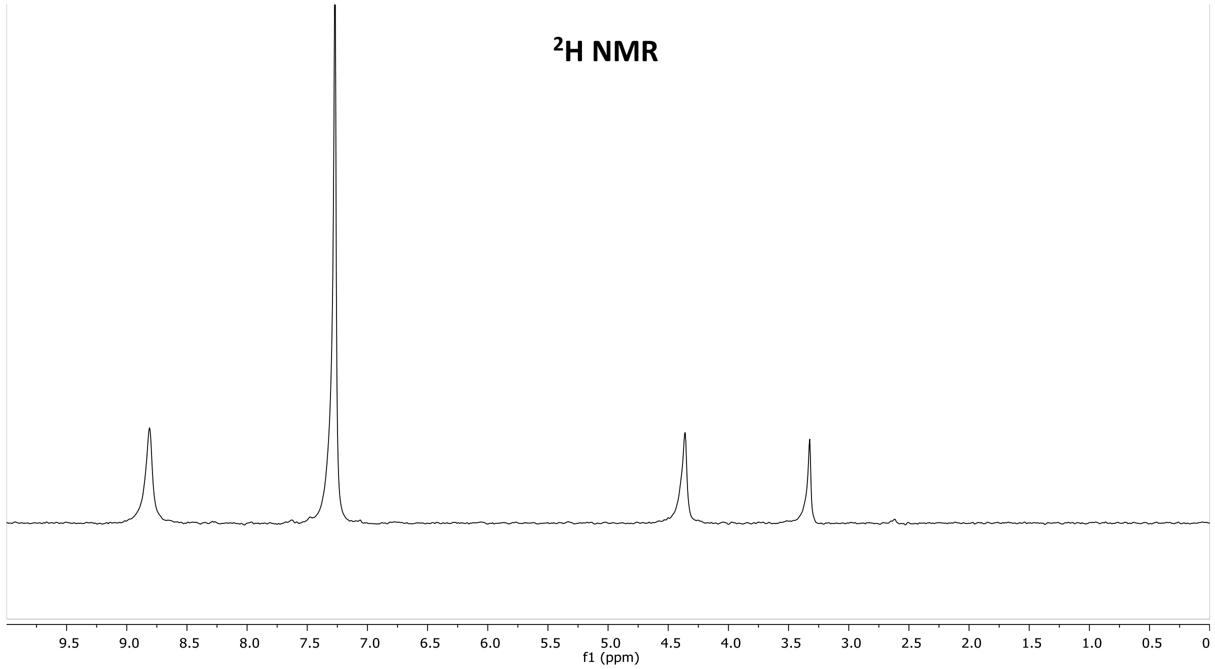

Experiment 11

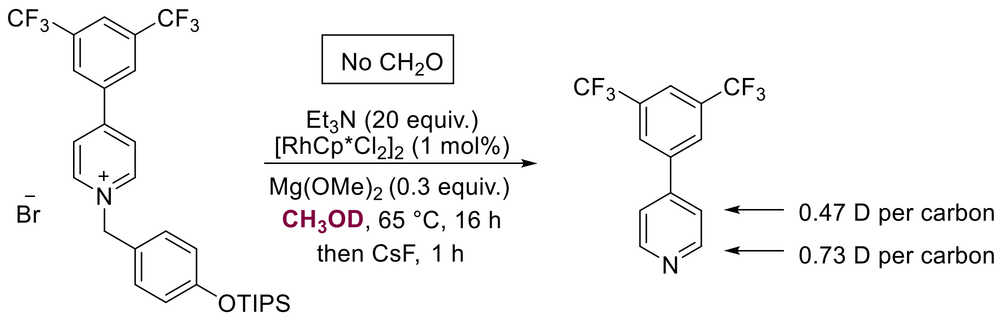

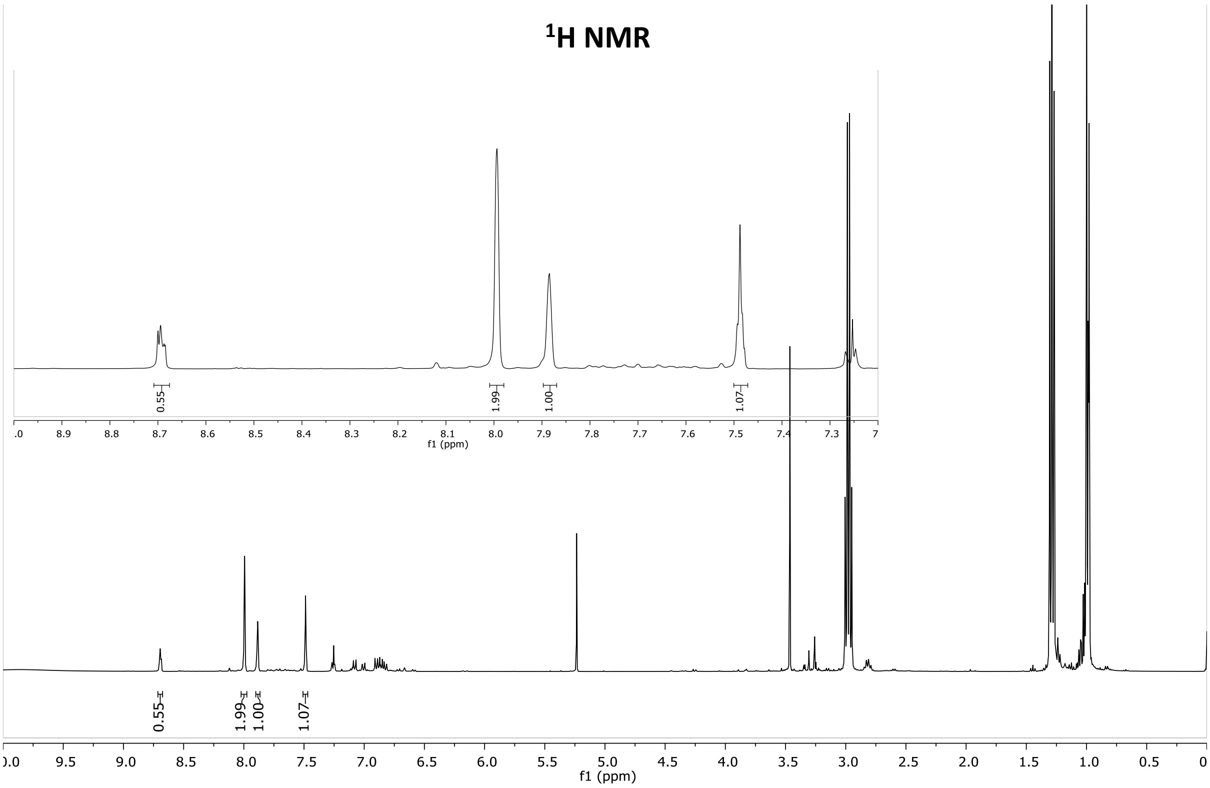

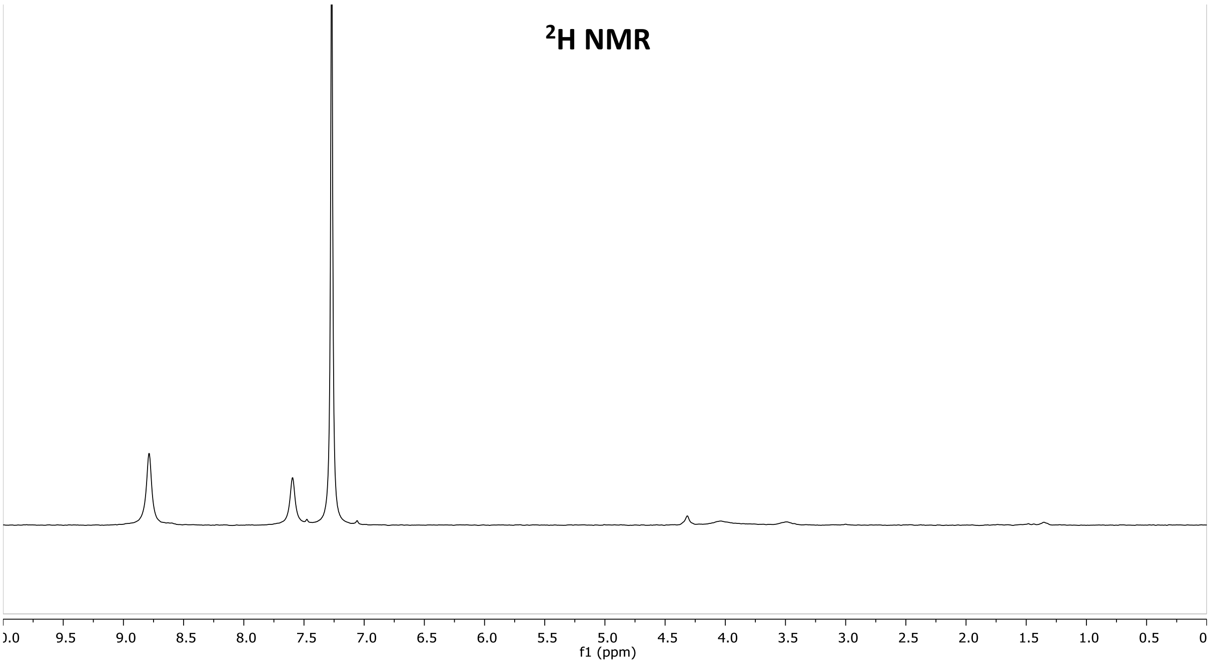

Experiment 12

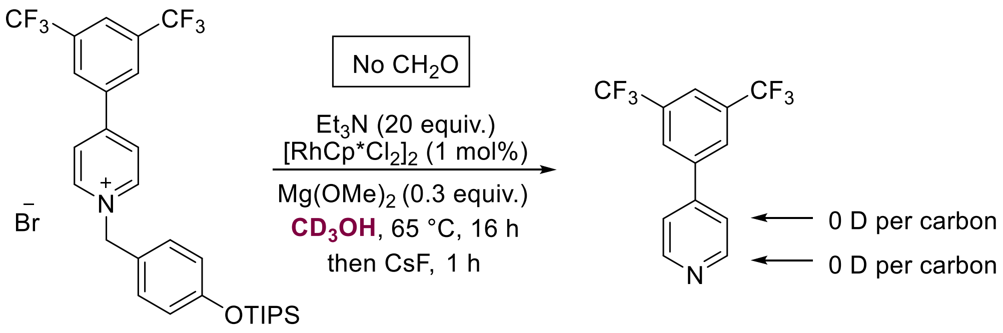

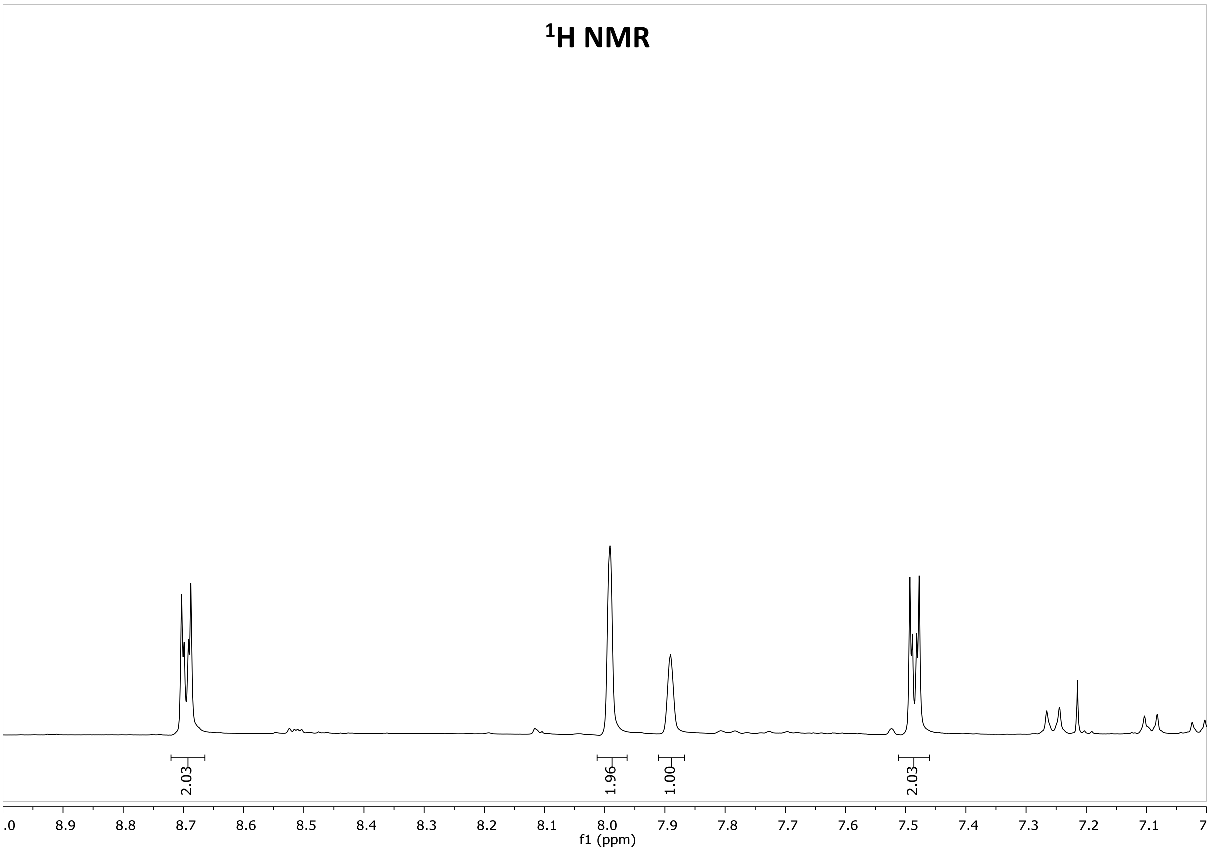

Supplement: SC-011-D0SC02759F-s001 [file SC-011-D0SC02759F-s001.pdf]
